# Author Correction: Sociality predicts orangutan vocal phenotype

**DOI:** 10.1038/s41559-025-02954-7

**Published:** 2025-12-18

**Authors:** Adriano R. Lameira, Guillermo Santamaría-Bonfil, Deborah Galeone, Marco Gamba, Madeleine E. Hardus, Cheryl D. Knott, Helen Morrogh-Bernard, Matthew G. Nowak, Gail Campbell-Smith, Serge A. Wich

**Affiliations:** 1 Department of Psychology, University of Warwick, Coventry, UK; 2 School of Psychology and Neuroscience, University of St Andrews, St Andrews, UK; 3 Instituto Nacional de Electricidad y Energías Limpias, Gerencia de Tecnologías de la Información, Cuernavaca, México; 4 Department of Life Sciences and Systems Biology, University of Torino, Turin, Italy; 5 Independent researcher, Warwick, UK; 6 Department of Anthropology, Boston University, Boston, MA USA; 7 Borneo Nature Foundation, Palangka Raya, Indonesia; 8 College of Life and Environmental Sciences, University of Exeter, Penryn, UK; 9 The PanEco Foundation—Sumatran Orangutan Conservation Programme, Berg am Irchel, Switzerland; 10 Department of Anthropology, Southern Illinois University, Carbondale, IL USA; 11 Yayasan Inisiasi Alam Rehabilitasi Indonesia, International Animal Rescue, Ketapang, Indonesia; 12 School of Natural Sciences and Psychology, Liverpool John Moores University, Liverpool, UK; 13 Faculty of Science, University of Amsterdam, Amsterdam, Netherlands

**Keywords:** Evolutionary theory, Evolutionary ecology, Human behaviour, Animal behaviour, Social behaviour

Correction to: *Nature Ecology & Evolution* 10.1038/s41559-022-01689-z, published online 21 March 2022.

After publication of the article, an error was identified in the data entry of the *maximum frequency* parameter for the Suaq orangutan population. Recalculation of entropy measures and reanalysis of the mixed models revealed that, for maximum frequency, the previously reported effect of sociality is no longer statistically supported (Emergence and self-organization: *F* = 0.321, *P* = 0.573; Complexity: *F* = 0.009, *P* = 0.927). The original results for *duration* remain unchanged and continue to show a significant effect of sociality. While the loss of statistical support for one parameter is regrettable, the revised findings are scientifically meaningful. They align with recent findings in chimpanzees^1^, showing that control over vocal parameters such as frequency and duration may operate independently. This suggests that social influences on vocal phenotypes may target specific acoustic features, and that specific populations may deploy features of vocal novelty in culturally localized ways.

The corrected analysis further reaffirms key methodological points raised in our original paper. In entropy-based analyses of behavioural novelty, low-probability events—sometimes mischaracterized as ‘outliers’—are not statistical noise but the core phenomena of interest. Their removal would bias entropy estimates and undermine the capacity to detect innovation. Given the nature of our study—multi-year, multi-site, and focused on a critically endangered species—each data point represents an irreplaceable behavioural observation. Removing such points without clear justification raises ethical concerns, including violation of IUCN data integrity guidelines and FAIR/TRUST data stewardship principles^2,3^. Our approach illustrates how ethical and methodological rigour must go hand-in-hand when working with vulnerable wild populations.

For the calculation of entropy values from continuous acoustic data, equal-width binning at the individual level remains a necessary and appropriate step^4^. Our binning approach was selected to capture vocal originality at the level of individual phenotypes—what we term “vocal personalities”—in response to social input. Other binning choices, such as a global binning, would be unable to distinguish between individual differences and novelty; benchmarking individuals against each other would be an analysis of group conformity, not of individual originality or vocal personality.

The Supplementary Information accompanying this amendment includes the original, uncorrected article for comparison (changes have been made to the Results and discussion, Methods, Table 1 and Fig. 2). Supplementary Data 3–5 have also been corrected and are available alongside the original article.

The authors would like to thank Peng-Fei Fan and Zi-Di Wang for intially bringing the issue to their attention.

## Supplementary information

Original, uncorrected article
