# Supplementary material for: Sociality predicts orangutan vocal phenotype
Source: Nat Ecol Evol. 2022 Mar 21;6(5):644–52. doi: 10.1038/s41559-022-01689-z (PMC9085614; doi:10.1038/s41559-022-01689-z)
Supplement: Supplementary file 1 — Reporting Summary [file 41559_2022_1689_MOESM1_ESM.pdf]

nature portfolio

Corresponding author(s): Adriano R. Lameira

Last updated by author(s): Jan 27, 2022

Reporting Summary

Nature Portfolio wishes to improve the reproducibility of the work that we publish. This form provides structure for consistency and transparency in reporting. For further information on Nature Portfolio policies, see our Editorial Policies and the Editorial Policy Checklist.

Statistics

For all statistical analyses, confirm that the following items are present in the figure legend, table legend, main text, or Methods section.

n/a	Confirmed	
☐	☒	The exact sample size (*n*) for each experimental group/condition, given as a discrete number and unit of measurement
☐	☒	A statement on whether measurements were taken from distinct samples or whether the same sample was measured repeatedly
☐	☒	The statistical test(s) used AND whether they are one- or two-sided<br>*Only common tests should be described solely by name; describe more complex techniques in the Methods section.*
☐	☒	A description of all covariates tested
☒	☐	A description of any assumptions or corrections, such as tests of normality and adjustment for multiple comparisons
☐	☒	A full description of the statistical parameters including central tendency (e.g. means) or other basic estimates (e.g. regression coefficient) AND variation (e.g. standard deviation) or associated estimates of uncertainty (e.g. confidence intervals)
☐	☒	For null hypothesis testing, the test statistic (e.g. *F*, *t*, *r*) with confidence intervals, effect sizes, degrees of freedom and *P* value noted<br>*Give P values as exact values whenever suitable.*
☒	☐	For Bayesian analysis, information on the choice of priors and Markov chain Monte Carlo settings
☒	☐	For hierarchical and complex designs, identification of the appropriate level for tests and full reporting of outcomes
☒	☐	Estimates of effect sizes (e.g. Cohen's *d*, Pearson's *r*), indicating how they were calculated

Our web collection on statistics for biologists contains articles on many of the points above.

Software and code

Policy information about availability of computer code

Data collection	N.a.
Data analysis	Raven interactive sound analysis software (version 1.5, Cornell Lab of Ornithology, Ithaca, New York) R 4.0 and R Studio

For manuscripts utilizing custom algorithms or software that are central to the research but not yet described in published literature, software must be made available to editors and reviewers. We strongly encourage code deposition in a community repository (e.g. GitHub). See the Nature Portfolio guidelines for submitting code & software for further information.

Data

Policy information about availability of data

All manuscripts must include a data availability statement. This statement should provide the following information, where applicable:

- Accession codes, unique identifiers, or web links for publicly available datasets
- A description of any restrictions on data availability
- For clinical datasets or third party data, please ensure that the statement adheres to our policy

All data needed to evaluate the conclusions in the paper are present in the paper and/or the Supplementary Materials. Additional data related to this paper may be requested from the authors.

Field-specific reporting

Please select the one below that is the best fit for your research. If you are not sure, read the appropriate sections before making your selection.

☐ Life sciences ☐ Behavioural & social sciences ☒ Ecological, evolutionary & environmental sciences

For a reference copy of the document with all sections, see nature.com/documents/nr-reporting-summary-flat.pdf

Ecological, evolutionary & environmental sciences study design

All studies must disclose on these points even when the disclosure is negative.

Study description	Acoustic and variation analyses of great ape vocal behaviour across wild populations
Research sample	All subjects wild individuals of genus Pongo. Nature/wild group composition across sexes and ages.
Sampling strategy	Opportunistic.
Data collection	Audio recordings by researchers.
Timing and spatial scale	Collected across 6 research sites. Minimum of 5 months at each site and a maximum of 20 months.
Data exclusions	No data excluded.
Reproducibility	N.a. No experimental protocol. Observational study only.
Randomization	Data collected according to opportunistic presence of wild individuals in the forest block associated with research station.
Blinding	N.a.

Did the study involve field work? ☒ Yes ☐ No

Field work, collection and transport

Field conditions	Low mountain rainforests and peat swamps of Borneo and Sumatra.
Location	Indonesia.
Access & import/export	N.a. No biological samples collected.
Disturbance	Groups of observers restricted to max 3 people. Max observation length of 10 days per individual with subsequent gap of 1 month without human follow/observation/data collection. Safety distance between human-ape maintained at all times, min. of 7 meters.

Reporting for specific materials, systems and methods

We require information from authors about some types of materials, experimental systems and methods used in many studies. Here, indicate whether each material, system or method listed is relevant to your study. If you are not sure if a list item applies to your research, read the appropriate section before selecting a response.

Materials & experimental systems

n/a	Involved in the study	
☒	☐	Antibodies
☒	☐	Eukaryotic cell lines
☒	☐	Palaeontology and archaeology
☐	☒	Animals and other organisms
☒	☐	Human research participants
☒	☐	Clinical data
☒	☐	Dual use research of concern

Methods

n/a	Involved in the study	
☒	☐	ChIP-seq
☒	☐	Flow cytometry
☒	☐	MRI-based neuroimaging

Animals and other organisms

Policy information about studies involving animals; ARRIVE guidelines recommended for reporting animal research

Laboratory animals	N.a.
Wild animals	No animal interaction, manipulation, transportation or substance administration were involved in the study. Observation only.
Field-collected samples	N.a.
Ethics oversight	All permissions were obtained from relevant authorities, namely, the Indonesian Ministry of Research and Technology (RISTEK), the Indonesian Ministry of Environment and Forestry (KLHK), the Directorate General of Natural Resources and Ecosystem Conservation (KSDAE), the former Directorate General of Forest Protection and Nature Conservation (PHKA), the Gunung Palung National Park (BTNGP), the Gunung Leuser National Park (TNGL), the Leuser Ecosystem Management Authority (BPKEL), the Universitas National (UNAS), the Center for International Cooperation in Sustainable Management of Tropical Peatland (CIMTROP), the University of Palangkaraya, the Tanjungpura University (UNTAN) and the Sumatran Orangutan Conservation Programme (SOCP) - PanEco Foundation.

Note that full information on the approval of the study protocol must also be provided in the manuscript.
