# Supplementary material for: Sociality predicts orangutan vocal phenotype
Source: Nat Ecol Evol. 2022 Mar 21;6(5):644–52. doi: 10.1038/s41559-022-01689-z (PMC9085614; doi:10.1038/s41559-022-01689-z)
Supplement: Supplementary file 3 — Descriptive statistics for acoustic data. [file 41559_2022_1689_MOESM3_ESM.html]

JASP 


# Results

## Grand Descriptive Statistics

| Descriptive Statistics | | | | | | | | | | | | | |
| --- | --- | --- | --- | --- | --- | --- | --- | --- | --- | --- | --- | --- | --- |
|  | | individual | | gender | | age-sex class | | context | | max freq | | duration | |
| Valid |  | 5295 |  | 5295 |  | 5295 |  | 5291 |  | 5295 |  | 5274 |  |
| Missing |  | 0 |  | 0 |  | 0 |  | 4 |  | 0 |  | 21 |  |
| Mean |  |  |  |  |  |  |  |  |  | 3752.342 |  | 0.479 |  |
| Std. Deviation |  |  |  |  |  |  |  |  |  | 1593.703 |  | 0.268 |  |
| Minimum |  |  |  |  |  |  |  |  |  | 0.300 |  | 0.038 |  |
| Maximum |  |  |  |  |  |  |  |  |  | 11800.200 |  | 7.136 |  |
|  | | | | | | | | | | | | | |
|  |  |  |  |  |  |  |  |  |  |  |  |  |  |
| --- | --- | --- | --- | --- | --- | --- | --- | --- | --- | --- | --- | --- | --- |
| *Note.*  Not all values are available for *Nominal Text* variables | | | | | | | | | | | | | |

### Frequency Tables

| Frequencies for individual | | | | | | | | | |
| --- | --- | --- | --- | --- | --- | --- | --- | --- | --- |
| individual | | Frequency | | Percent | | Valid Percent | | Cumulative Percent | |
| Alice |  | 20 |  | 0.378 |  | 0.378 |  | 0.378 |  |
| Aminah |  | 26 |  | 0.491 |  | 0.491 |  | 0.869 |  |
| Anto |  | 5 |  | 0.094 |  | 0.094 |  | 0.963 |  |
| Asny |  | 15 |  | 0.283 |  | 0.283 |  | 1.246 |  |
| Bagong |  | 101 |  | 1.907 |  | 1.907 |  | 3.154 |  |
| Bendot |  | 65 |  | 1.228 |  | 1.228 |  | 4.381 |  |
| Berani |  | 51 |  | 0.963 |  | 0.963 |  | 5.345 |  |
| Beth |  | 46 |  | 0.869 |  | 0.869 |  | 6.213 |  |
| Bibi |  | 13 |  | 0.246 |  | 0.246 |  | 6.459 |  |
| Bintang |  | 93 |  | 1.756 |  | 1.756 |  | 8.215 |  |
| Brutus |  | 183 |  | 3.456 |  | 3.456 |  | 11.671 |  |
| Chindy |  | 22 |  | 0.415 |  | 0.415 |  | 12.087 |  |
| Codet |  | 32 |  | 0.604 |  | 0.604 |  | 12.691 |  |
| Elly |  | 51 |  | 0.963 |  | 0.963 |  | 13.654 |  |
| Fajar |  | 246 |  | 4.646 |  | 4.646 |  | 18.300 |  |
| Feb |  | 4 |  | 0.076 |  | 0.076 |  | 18.376 |  |
| Female |  | 19 |  | 0.359 |  | 0.359 |  | 18.735 |  |
| Fio |  | 9 |  | 0.170 |  | 0.170 |  | 18.905 |  |
| Flanged male |  | 6 |  | 0.113 |  | 0.113 |  | 19.018 |  |
| Freddy |  | 1 |  | 0.019 |  | 0.019 |  | 19.037 |  |
| Friska |  | 1 |  | 0.019 |  | 0.019 |  | 19.056 |  |
| Fugit |  | 126 |  | 2.380 |  | 2.380 |  | 21.435 |  |
| Gangstah |  | 17 |  | 0.321 |  | 0.321 |  | 21.756 |  |
| Gordon |  | 52 |  | 0.982 |  | 0.982 |  | 22.738 |  |
| Gracia |  | 54 |  | 1.020 |  | 1.020 |  | 23.758 |  |
| Gretel |  | 36 |  | 0.680 |  | 0.680 |  | 24.438 |  |
| Henk |  | 4 |  | 0.076 |  | 0.076 |  | 24.514 |  |
| Icarus |  | 5 |  | 0.094 |  | 0.094 |  | 24.608 |  |
| Imp |  | 49 |  | 0.925 |  | 0.925 |  | 25.534 |  |
| Indah |  | 17 |  | 0.321 |  | 0.321 |  | 25.855 |  |
| Indi |  | 20 |  | 0.378 |  | 0.378 |  | 26.232 |  |
| Irma |  | 1590 |  | 30.028 |  | 30.028 |  | 56.261 |  |
| James |  | 7 |  | 0.132 |  | 0.132 |  | 56.393 |  |
| Janda Tua |  | 3 |  | 0.057 |  | 0.057 |  | 56.449 |  |
| Jinak |  | 41 |  | 0.774 |  | 0.774 |  | 57.224 |  |
| Joy |  | 2 |  | 0.038 |  | 0.038 |  | 57.262 |  |
| Juni |  | 4 |  | 0.076 |  | 0.076 |  | 57.337 |  |
| Juno |  | 14 |  | 0.264 |  | 0.264 |  | 57.602 |  |
| Kacil |  | 5 |  | 0.094 |  | 0.094 |  | 57.696 |  |
| Kan |  | 2 |  | 0.038 |  | 0.038 |  | 57.734 |  |
| Kasi |  | 159 |  | 3.003 |  | 3.003 |  | 60.737 |  |
| Kay |  | 111 |  | 2.096 |  | 2.096 |  | 62.833 |  |
| Keri |  | 6 |  | 0.113 |  | 0.113 |  | 62.946 |  |
| Keto |  | 1 |  | 0.019 |  | 0.019 |  | 62.965 |  |
| Kondor |  | 38 |  | 0.718 |  | 0.718 |  | 63.683 |  |
| Kundur |  | 42 |  | 0.793 |  | 0.793 |  | 64.476 |  |
| Madalena |  | 15 |  | 0.283 |  | 0.283 |  | 64.759 |  |
| Malé |  | 104 |  | 1.964 |  | 1.964 |  | 66.723 |  |
| Mindi |  | 17 |  | 0.321 |  | 0.321 |  | 67.044 |  |
| Ompung |  | 263 |  | 4.967 |  | 4.967 |  | 72.011 |  |
| Pensi |  | 15 |  | 0.283 |  | 0.283 |  | 72.295 |  |
| Peot |  | 8 |  | 0.151 |  | 0.151 |  | 72.446 |  |
| Prabu |  | 80 |  | 1.511 |  | 1.511 |  | 73.957 |  |
| Raffi |  | 61 |  | 1.152 |  | 1.152 |  | 75.109 |  |
| Rambo |  | 29 |  | 0.548 |  | 0.548 |  | 75.656 |  |
| Ronaldo |  | 1 |  | 0.019 |  | 0.019 |  | 75.675 |  |
| Salvador |  | 46 |  | 0.869 |  | 0.869 |  | 76.544 |  |
| Suci |  | 5 |  | 0.094 |  | 0.094 |  | 76.638 |  |
| Sultan |  | 106 |  | 2.002 |  | 2.002 |  | 78.640 |  |
| Sumi |  | 32 |  | 0.604 |  | 0.604 |  | 79.245 |  |
| Teju |  | 22 |  | 0.415 |  | 0.415 |  | 79.660 |  |
| Teresia |  | 28 |  | 0.529 |  | 0.529 |  | 80.189 |  |
| Timi |  | 3 |  | 0.057 |  | 0.057 |  | 80.246 |  |
| Tina |  | 316 |  | 5.968 |  | 5.968 |  | 86.213 |  |
| Travor |  | 1 |  | 0.019 |  | 0.019 |  | 86.232 |  |
| Umi |  | 2 |  | 0.038 |  | 0.038 |  | 86.270 |  |
| Unflm |  | 32 |  | 0.604 |  | 0.604 |  | 86.874 |  |
| Uok |  | 49 |  | 0.925 |  | 0.925 |  | 87.800 |  |
| Vulcan |  | 2 |  | 0.038 |  | 0.038 |  | 87.838 |  |
| Walimah |  | 100 |  | 1.889 |  | 1.889 |  | 89.726 |  |
| Wulan |  | 69 |  | 1.303 |  | 1.303 |  | 91.029 |  |
| XL |  | 41 |  | 0.774 |  | 0.774 |  | 91.804 |  |
| Xenix |  | 8 |  | 0.151 |  | 0.151 |  | 91.955 |  |
| Yanti |  | 414 |  | 7.819 |  | 7.819 |  | 99.773 |  |
| Zeus |  | 1 |  | 0.019 |  | 0.019 |  | 99.792 |  |
| Zorro |  | 11 |  | 0.208 |  | 0.208 |  | 100.000 |  |
| Missing |  | 0 |  | 0.000 |  |  |  |  |  |
| Total |  | 5295 |  | 100.000 |  |  |  |  |  |
|  | | | | | | | | | |

| Frequencies for gender | | | | | | | | | |
| --- | --- | --- | --- | --- | --- | --- | --- | --- | --- |
| gender | | Frequency | | Percent | | Valid Percent | | Cumulative Percent | |
| female |  | 3540 |  | 66.856 |  | 66.856 |  | 66.856 |  |
| male |  | 1755 |  | 33.144 |  | 33.144 |  | 100.000 |  |
| Missing |  | 0 |  | 0.000 |  |  |  |  |  |
| Total |  | 5295 |  | 100.000 |  |  |  |  |  |
|  | | | | | | | | | |

| Frequencies for age-sex class | | | | | | | | | |
| --- | --- | --- | --- | --- | --- | --- | --- | --- | --- |
| age-sex class | | Frequency | | Percent | | Valid Percent | | Cumulative Percent | |
| adolescent |  | 654 |  | 12.351 |  | 12.351 |  | 12.351 |  |
| female with infant |  | 2771 |  | 52.332 |  | 52.332 |  | 64.684 |  |
| flanged male |  | 1290 |  | 24.363 |  | 24.363 |  | 89.046 |  |
| infant |  | 181 |  | 3.418 |  | 3.418 |  | 92.465 |  |
| unflanged male |  | 399 |  | 7.535 |  | 7.535 |  | 100.000 |  |
| Missing |  | 0 |  | 0.000 |  |  |  |  |  |
| Total |  | 5295 |  | 100.000 |  |  |  |  |  |
|  | | | | | | | | | |

| Frequencies for context | | | | | | | | | |
| --- | --- | --- | --- | --- | --- | --- | --- | --- | --- |
| context | | Frequency | | Percent | | Valid Percent | | Cumulative Percent | |
| no apparent danger |  | 31 |  | 0.585 |  | 0.586 |  | 0.586 |  |
| towards animals |  | 97 |  | 1.832 |  | 1.833 |  | 2.419 |  |
| towards humans (non-observers) |  | 22 |  | 0.415 |  | 0.416 |  | 2.835 |  |
| towards observers |  | 4924 |  | 92.993 |  | 93.064 |  | 95.899 |  |
| towards other orangutans |  | 217 |  | 4.098 |  | 4.101 |  | 100.000 |  |
| Missing |  | 4 |  | 0.076 |  |  |  |  |  |
| Total |  | 5295 |  | 100.000 |  |  |  |  |  |
|  | | | | | | | | | |

## Descriptive Statistics, split by Population

| Descriptive Statistics | | | | | | | | | | | | | | | | | | | | | | | | | | | | | | | | | | | | | | | | | | | | | | | | | | | | | | | | | | | | | | | | | | | | | | | | | |
| --- | --- | --- | --- | --- | --- | --- | --- | --- | --- | --- | --- | --- | --- | --- | --- | --- | --- | --- | --- | --- | --- | --- | --- | --- | --- | --- | --- | --- | --- | --- | --- | --- | --- | --- | --- | --- | --- | --- | --- | --- | --- | --- | --- | --- | --- | --- | --- | --- | --- | --- | --- | --- | --- | --- | --- | --- | --- | --- | --- | --- | --- | --- | --- | --- | --- | --- | --- | --- | --- | --- | --- | --- | --- |
|  | | individual | | | | | | | | | | | | gender | | | | | | | | | | | | age-sex class | | | | | | | | | | | | context | | | | | | | | | | | | max freq | | | | | | | | | | | | duration | | | | | | | | | | | |
|  | | Gunung Palung | | Sabangau | | Sampan Getek | | Sikundur | | Suaq | | Tuanan | | Gunung Palung | | Sabangau | | Sampan Getek | | Sikundur | | Suaq | | Tuanan | | Gunung Palung | | Sabangau | | Sampan Getek | | Sikundur | | Suaq | | Tuanan | | Gunung Palung | | Sabangau | | Sampan Getek | | Sikundur | | Suaq | | Tuanan | | Gunung Palung | | Sabangau | | Sampan Getek | | Sikundur | | Suaq | | Tuanan | | Gunung Palung | | Sabangau | | Sampan Getek | | Sikundur | | Suaq | | Tuanan | |
| Valid |  | 814 |  | 257 |  | 546 |  | 2589 |  | 547 |  | 542 |  | 814 |  | 257 |  | 546 |  | 2589 |  | 547 |  | 542 |  | 814 |  | 257 |  | 546 |  | 2589 |  | 547 |  | 542 |  | 814 |  | 257 |  | 546 |  | 2589 |  | 547 |  | 538 |  | 814 |  | 257 |  | 546 |  | 2589 |  | 547 |  | 542 |  | 814 |  | 257 |  | 525 |  | 2589 |  | 547 |  | 542 |  |
| Missing |  | 0 |  | 0 |  | 0 |  | 0 |  | 0 |  | 0 |  | 0 |  | 0 |  | 0 |  | 0 |  | 0 |  | 0 |  | 0 |  | 0 |  | 0 |  | 0 |  | 0 |  | 0 |  | 0 |  | 0 |  | 0 |  | 0 |  | 0 |  | 4 |  | 0 |  | 0 |  | 0 |  | 0 |  | 0 |  | 0 |  | 0 |  | 0 |  | 21 |  | 0 |  | 0 |  | 0 |  |
| Mean |  |  |  |  |  |  |  |  |  |  |  |  |  |  |  |  |  |  |  |  |  |  |  |  |  |  |  |  |  |  |  |  |  |  |  |  |  |  |  |  |  |  |  |  |  |  |  |  |  | 3921.016 |  | 3428.832 |  | 3909.392 |  | 4501.918 |  | 139.311 |  | 3560.031 |  | 0.624 |  | 0.510 |  | 0.614 |  | 0.454 |  | 0.380 |  | 0.341 |  |
| Std. Deviation |  |  |  |  |  |  |  |  |  |  |  |  |  |  |  |  |  |  |  |  |  |  |  |  |  |  |  |  |  |  |  |  |  |  |  |  |  |  |  |  |  |  |  |  |  |  |  |  |  | 1530.885 |  | 1057.007 |  | 798.478 |  | 736.697 |  | 7.194 |  | 1234.107 |  | 0.387 |  | 0.404 |  | 0.180 |  | 0.175 |  | 0.190 |  | 0.322 |  |
| Minimum |  |  |  |  |  |  |  |  |  |  |  |  |  |  |  |  |  |  |  |  |  |  |  |  |  |  |  |  |  |  |  |  |  |  |  |  |  |  |  |  |  |  |  |  |  |  |  |  |  | 172.300 |  | 1181.600 |  | 468.800 |  | 1711.900 |  | 115.400 |  | 0.300 |  | 0.129 |  | 0.038 |  | 0.211 |  | 0.044 |  | 0.091 |  | 0.084 |  |
| Maximum |  |  |  |  |  |  |  |  |  |  |  |  |  |  |  |  |  |  |  |  |  |  |  |  |  |  |  |  |  |  |  |  |  |  |  |  |  |  |  |  |  |  |  |  |  |  |  |  |  | 11800.200 |  | 7345.500 |  | 9093.800 |  | 8516.400 |  | 164.400 |  | 10174.400 |  | 4.985 |  | 2.962 |  | 1.485 |  | 1.503 |  | 1.311 |  | 7.136 |  |
|  | | | | | | | | | | | | | | | | | | | | | | | | | | | | | | | | | | | | | | | | | | | | | | | | | | | | | | | | | | | | | | | | | | | | | | | | | |
|  |  |  |  |  |  |  |  |  |  |  |  |  |  |  |  |  |  |  |  |  |  |  |  |  |  |  |  |  |  |  |  |  |  |  |  |  |  |  |  |  |  |  |  |  |  |  |  |  |  |  |  |  |  |  |  |  |  |  |  |  |  |  |  |  |  |  |  |  |  |  |  |  |  |
| --- | --- | --- | --- | --- | --- | --- | --- | --- | --- | --- | --- | --- | --- | --- | --- | --- | --- | --- | --- | --- | --- | --- | --- | --- | --- | --- | --- | --- | --- | --- | --- | --- | --- | --- | --- | --- | --- | --- | --- | --- | --- | --- | --- | --- | --- | --- | --- | --- | --- | --- | --- | --- | --- | --- | --- | --- | --- | --- | --- | --- | --- | --- | --- | --- | --- | --- | --- | --- | --- | --- | --- | --- | --- |
| *Note.*  Not all values are available for *Nominal Text* variables | | | | | | | | | | | | | | | | | | | | | | | | | | | | | | | | | | | | | | | | | | | | | | | | | | | | | | | | | | | | | | | | | | | | | | | | | |

### Frequency Tables

| Frequencies for individual | | | | | | | | | | | |
| --- | --- | --- | --- | --- | --- | --- | --- | --- | --- | --- | --- |
| population | | individual | | Frequency | | Percent | | Valid Percent | | Cumulative Percent | |
| Gunung Palung |  | Alice |  | 0 |  | 0.000 |  | 0.000 |  | 0.000 |  |
|  |  | Aminah |  | 26 |  | 3.194 |  | 3.194 |  | 3.194 |  |
|  |  | Anto |  | 0 |  | 0.000 |  | 0.000 |  | 3.194 |  |
|  |  | Asny |  | 15 |  | 1.843 |  | 1.843 |  | 5.037 |  |
|  |  | Bagong |  | 0 |  | 0.000 |  | 0.000 |  | 5.037 |  |
|  |  | Bendot |  | 0 |  | 0.000 |  | 0.000 |  | 5.037 |  |
|  |  | Berani |  | 51 |  | 6.265 |  | 6.265 |  | 11.302 |  |
|  |  | Beth |  | 46 |  | 5.651 |  | 5.651 |  | 16.953 |  |
|  |  | Bibi |  | 13 |  | 1.597 |  | 1.597 |  | 18.550 |  |
|  |  | Bintang |  | 0 |  | 0.000 |  | 0.000 |  | 18.550 |  |
|  |  | Brutus |  | 0 |  | 0.000 |  | 0.000 |  | 18.550 |  |
|  |  | Chindy |  | 0 |  | 0.000 |  | 0.000 |  | 18.550 |  |
|  |  | Codet |  | 32 |  | 3.931 |  | 3.931 |  | 22.482 |  |
|  |  | Elly |  | 0 |  | 0.000 |  | 0.000 |  | 22.482 |  |
|  |  | Fajar |  | 246 |  | 30.221 |  | 30.221 |  | 52.703 |  |
|  |  | Feb |  | 0 |  | 0.000 |  | 0.000 |  | 52.703 |  |
|  |  | Female |  | 0 |  | 0.000 |  | 0.000 |  | 52.703 |  |
|  |  | Fio |  | 0 |  | 0.000 |  | 0.000 |  | 52.703 |  |
|  |  | Flanged male |  | 0 |  | 0.000 |  | 0.000 |  | 52.703 |  |
|  |  | Freddy |  | 0 |  | 0.000 |  | 0.000 |  | 52.703 |  |
|  |  | Friska |  | 0 |  | 0.000 |  | 0.000 |  | 52.703 |  |
|  |  | Fugit |  | 0 |  | 0.000 |  | 0.000 |  | 52.703 |  |
|  |  | Gangstah |  | 0 |  | 0.000 |  | 0.000 |  | 52.703 |  |
|  |  | Gordon |  | 52 |  | 6.388 |  | 6.388 |  | 59.091 |  |
|  |  | Gracia |  | 0 |  | 0.000 |  | 0.000 |  | 59.091 |  |
|  |  | Gretel |  | 0 |  | 0.000 |  | 0.000 |  | 59.091 |  |
|  |  | Henk |  | 0 |  | 0.000 |  | 0.000 |  | 59.091 |  |
|  |  | Icarus |  | 0 |  | 0.000 |  | 0.000 |  | 59.091 |  |
|  |  | Imp |  | 0 |  | 0.000 |  | 0.000 |  | 59.091 |  |
|  |  | Indah |  | 17 |  | 2.088 |  | 2.088 |  | 61.179 |  |
|  |  | Indi |  | 20 |  | 2.457 |  | 2.457 |  | 63.636 |  |
|  |  | Irma |  | 0 |  | 0.000 |  | 0.000 |  | 63.636 |  |
|  |  | James |  | 0 |  | 0.000 |  | 0.000 |  | 63.636 |  |
|  |  | Janda Tua |  | 3 |  | 0.369 |  | 0.369 |  | 64.005 |  |
|  |  | Jinak |  | 0 |  | 0.000 |  | 0.000 |  | 64.005 |  |
|  |  | Joy |  | 0 |  | 0.000 |  | 0.000 |  | 64.005 |  |
|  |  | Juni |  | 0 |  | 0.000 |  | 0.000 |  | 64.005 |  |
|  |  | Juno |  | 0 |  | 0.000 |  | 0.000 |  | 64.005 |  |
|  |  | Kacil |  | 0 |  | 0.000 |  | 0.000 |  | 64.005 |  |
|  |  | Kan |  | 2 |  | 0.246 |  | 0.246 |  | 64.251 |  |
|  |  | Kasi |  | 0 |  | 0.000 |  | 0.000 |  | 64.251 |  |
|  |  | Kay |  | 0 |  | 0.000 |  | 0.000 |  | 64.251 |  |
|  |  | Keri |  | 0 |  | 0.000 |  | 0.000 |  | 64.251 |  |
|  |  | Keto |  | 0 |  | 0.000 |  | 0.000 |  | 64.251 |  |
|  |  | Kondor |  | 0 |  | 0.000 |  | 0.000 |  | 64.251 |  |
|  |  | Kundur |  | 0 |  | 0.000 |  | 0.000 |  | 64.251 |  |
|  |  | Madalena |  | 0 |  | 0.000 |  | 0.000 |  | 64.251 |  |
|  |  | Malé |  | 0 |  | 0.000 |  | 0.000 |  | 64.251 |  |
|  |  | Mindi |  | 0 |  | 0.000 |  | 0.000 |  | 64.251 |  |
|  |  | Ompung |  | 0 |  | 0.000 |  | 0.000 |  | 64.251 |  |
|  |  | Pensi |  | 0 |  | 0.000 |  | 0.000 |  | 64.251 |  |
|  |  | Peot |  | 8 |  | 0.983 |  | 0.983 |  | 65.233 |  |
|  |  | Prabu |  | 80 |  | 9.828 |  | 9.828 |  | 75.061 |  |
|  |  | Raffi |  | 0 |  | 0.000 |  | 0.000 |  | 75.061 |  |
|  |  | Rambo |  | 0 |  | 0.000 |  | 0.000 |  | 75.061 |  |
|  |  | Ronaldo |  | 0 |  | 0.000 |  | 0.000 |  | 75.061 |  |
|  |  | Salvador |  | 0 |  | 0.000 |  | 0.000 |  | 75.061 |  |
|  |  | Suci |  | 0 |  | 0.000 |  | 0.000 |  | 75.061 |  |
|  |  | Sultan |  | 0 |  | 0.000 |  | 0.000 |  | 75.061 |  |
|  |  | Sumi |  | 0 |  | 0.000 |  | 0.000 |  | 75.061 |  |
|  |  | Teju |  | 0 |  | 0.000 |  | 0.000 |  | 75.061 |  |
|  |  | Teresia |  | 0 |  | 0.000 |  | 0.000 |  | 75.061 |  |
|  |  | Timi |  | 0 |  | 0.000 |  | 0.000 |  | 75.061 |  |
|  |  | Tina |  | 0 |  | 0.000 |  | 0.000 |  | 75.061 |  |
|  |  | Travor |  | 0 |  | 0.000 |  | 0.000 |  | 75.061 |  |
|  |  | Umi |  | 2 |  | 0.246 |  | 0.246 |  | 75.307 |  |
|  |  | Unflm |  | 0 |  | 0.000 |  | 0.000 |  | 75.307 |  |
|  |  | Uok |  | 49 |  | 6.020 |  | 6.020 |  | 81.327 |  |
|  |  | Vulcan |  | 0 |  | 0.000 |  | 0.000 |  | 81.327 |  |
|  |  | Walimah |  | 100 |  | 12.285 |  | 12.285 |  | 93.612 |  |
|  |  | Wulan |  | 0 |  | 0.000 |  | 0.000 |  | 93.612 |  |
|  |  | XL |  | 41 |  | 5.037 |  | 5.037 |  | 98.649 |  |
|  |  | Xenix |  | 0 |  | 0.000 |  | 0.000 |  | 98.649 |  |
|  |  | Yanti |  | 0 |  | 0.000 |  | 0.000 |  | 98.649 |  |
|  |  | Zeus |  | 0 |  | 0.000 |  | 0.000 |  | 98.649 |  |
|  |  | Zorro |  | 11 |  | 1.351 |  | 1.351 |  | 100.000 |  |
|  |  | Missing |  | 0 |  | 0.000 |  |  |  |  |  |
|  |  | Total |  | 814 |  | 100.000 |  |  |  |  |  |
| Sabangau |  | Alice |  | 0 |  | 0.000 |  | 0.000 |  | 0.000 |  |
|  |  | Aminah |  | 0 |  | 0.000 |  | 0.000 |  | 0.000 |  |
|  |  | Anto |  | 0 |  | 0.000 |  | 0.000 |  | 0.000 |  |
|  |  | Asny |  | 0 |  | 0.000 |  | 0.000 |  | 0.000 |  |
|  |  | Bagong |  | 0 |  | 0.000 |  | 0.000 |  | 0.000 |  |
|  |  | Bendot |  | 0 |  | 0.000 |  | 0.000 |  | 0.000 |  |
|  |  | Berani |  | 0 |  | 0.000 |  | 0.000 |  | 0.000 |  |
|  |  | Beth |  | 0 |  | 0.000 |  | 0.000 |  | 0.000 |  |
|  |  | Bibi |  | 0 |  | 0.000 |  | 0.000 |  | 0.000 |  |
|  |  | Bintang |  | 0 |  | 0.000 |  | 0.000 |  | 0.000 |  |
|  |  | Brutus |  | 0 |  | 0.000 |  | 0.000 |  | 0.000 |  |
|  |  | Chindy |  | 0 |  | 0.000 |  | 0.000 |  | 0.000 |  |
|  |  | Codet |  | 0 |  | 0.000 |  | 0.000 |  | 0.000 |  |
|  |  | Elly |  | 0 |  | 0.000 |  | 0.000 |  | 0.000 |  |
|  |  | Fajar |  | 0 |  | 0.000 |  | 0.000 |  | 0.000 |  |
|  |  | Feb |  | 4 |  | 1.556 |  | 1.556 |  | 1.556 |  |
|  |  | Female |  | 19 |  | 7.393 |  | 7.393 |  | 8.949 |  |
|  |  | Fio |  | 9 |  | 3.502 |  | 3.502 |  | 12.451 |  |
|  |  | Flanged male |  | 0 |  | 0.000 |  | 0.000 |  | 12.451 |  |
|  |  | Freddy |  | 0 |  | 0.000 |  | 0.000 |  | 12.451 |  |
|  |  | Friska |  | 0 |  | 0.000 |  | 0.000 |  | 12.451 |  |
|  |  | Fugit |  | 0 |  | 0.000 |  | 0.000 |  | 12.451 |  |
|  |  | Gangstah |  | 0 |  | 0.000 |  | 0.000 |  | 12.451 |  |
|  |  | Gordon |  | 0 |  | 0.000 |  | 0.000 |  | 12.451 |  |
|  |  | Gracia |  | 54 |  | 21.012 |  | 21.012 |  | 33.463 |  |
|  |  | Gretel |  | 36 |  | 14.008 |  | 14.008 |  | 47.471 |  |
|  |  | Henk |  | 0 |  | 0.000 |  | 0.000 |  | 47.471 |  |
|  |  | Icarus |  | 5 |  | 1.946 |  | 1.946 |  | 49.416 |  |
|  |  | Imp |  | 0 |  | 0.000 |  | 0.000 |  | 49.416 |  |
|  |  | Indah |  | 0 |  | 0.000 |  | 0.000 |  | 49.416 |  |
|  |  | Indi |  | 0 |  | 0.000 |  | 0.000 |  | 49.416 |  |
|  |  | Irma |  | 0 |  | 0.000 |  | 0.000 |  | 49.416 |  |
|  |  | James |  | 0 |  | 0.000 |  | 0.000 |  | 49.416 |  |
|  |  | Janda Tua |  | 0 |  | 0.000 |  | 0.000 |  | 49.416 |  |
|  |  | Jinak |  | 0 |  | 0.000 |  | 0.000 |  | 49.416 |  |
|  |  | Joy |  | 2 |  | 0.778 |  | 0.778 |  | 50.195 |  |
|  |  | Juni |  | 0 |  | 0.000 |  | 0.000 |  | 50.195 |  |
|  |  | Juno |  | 14 |  | 5.447 |  | 5.447 |  | 55.642 |  |
|  |  | Kacil |  | 0 |  | 0.000 |  | 0.000 |  | 55.642 |  |
|  |  | Kan |  | 0 |  | 0.000 |  | 0.000 |  | 55.642 |  |
|  |  | Kasi |  | 0 |  | 0.000 |  | 0.000 |  | 55.642 |  |
|  |  | Kay |  | 0 |  | 0.000 |  | 0.000 |  | 55.642 |  |
|  |  | Keri |  | 0 |  | 0.000 |  | 0.000 |  | 55.642 |  |
|  |  | Keto |  | 1 |  | 0.389 |  | 0.389 |  | 56.031 |  |
|  |  | Kondor |  | 0 |  | 0.000 |  | 0.000 |  | 56.031 |  |
|  |  | Kundur |  | 0 |  | 0.000 |  | 0.000 |  | 56.031 |  |
|  |  | Madalena |  | 0 |  | 0.000 |  | 0.000 |  | 56.031 |  |
|  |  | Malé |  | 0 |  | 0.000 |  | 0.000 |  | 56.031 |  |
|  |  | Mindi |  | 0 |  | 0.000 |  | 0.000 |  | 56.031 |  |
|  |  | Ompung |  | 0 |  | 0.000 |  | 0.000 |  | 56.031 |  |
|  |  | Pensi |  | 0 |  | 0.000 |  | 0.000 |  | 56.031 |  |
|  |  | Peot |  | 0 |  | 0.000 |  | 0.000 |  | 56.031 |  |
|  |  | Prabu |  | 0 |  | 0.000 |  | 0.000 |  | 56.031 |  |
|  |  | Raffi |  | 0 |  | 0.000 |  | 0.000 |  | 56.031 |  |
|  |  | Rambo |  | 0 |  | 0.000 |  | 0.000 |  | 56.031 |  |
|  |  | Ronaldo |  | 0 |  | 0.000 |  | 0.000 |  | 56.031 |  |
|  |  | Salvador |  | 46 |  | 17.899 |  | 17.899 |  | 73.930 |  |
|  |  | Suci |  | 0 |  | 0.000 |  | 0.000 |  | 73.930 |  |
|  |  | Sultan |  | 0 |  | 0.000 |  | 0.000 |  | 73.930 |  |
|  |  | Sumi |  | 0 |  | 0.000 |  | 0.000 |  | 73.930 |  |
|  |  | Teju |  | 0 |  | 0.000 |  | 0.000 |  | 73.930 |  |
|  |  | Teresia |  | 28 |  | 10.895 |  | 10.895 |  | 84.825 |  |
|  |  | Timi |  | 3 |  | 1.167 |  | 1.167 |  | 85.992 |  |
|  |  | Tina |  | 0 |  | 0.000 |  | 0.000 |  | 85.992 |  |
|  |  | Travor |  | 1 |  | 0.389 |  | 0.389 |  | 86.381 |  |
|  |  | Umi |  | 0 |  | 0.000 |  | 0.000 |  | 86.381 |  |
|  |  | Unflm |  | 32 |  | 12.451 |  | 12.451 |  | 98.833 |  |
|  |  | Uok |  | 0 |  | 0.000 |  | 0.000 |  | 98.833 |  |
|  |  | Vulcan |  | 2 |  | 0.778 |  | 0.778 |  | 99.611 |  |
|  |  | Walimah |  | 0 |  | 0.000 |  | 0.000 |  | 99.611 |  |
|  |  | Wulan |  | 0 |  | 0.000 |  | 0.000 |  | 99.611 |  |
|  |  | XL |  | 0 |  | 0.000 |  | 0.000 |  | 99.611 |  |
|  |  | Xenix |  | 0 |  | 0.000 |  | 0.000 |  | 99.611 |  |
|  |  | Yanti |  | 0 |  | 0.000 |  | 0.000 |  | 99.611 |  |
|  |  | Zeus |  | 1 |  | 0.389 |  | 0.389 |  | 100.000 |  |
|  |  | Zorro |  | 0 |  | 0.000 |  | 0.000 |  | 100.000 |  |
|  |  | Missing |  | 0 |  | 0.000 |  |  |  |  |  |
|  |  | Total |  | 257 |  | 100.000 |  |  |  |  |  |
| Sampan Getek |  | Alice |  | 0 |  | 0.000 |  | 0.000 |  | 0.000 |  |
|  |  | Aminah |  | 0 |  | 0.000 |  | 0.000 |  | 0.000 |  |
|  |  | Anto |  | 0 |  | 0.000 |  | 0.000 |  | 0.000 |  |
|  |  | Asny |  | 0 |  | 0.000 |  | 0.000 |  | 0.000 |  |
|  |  | Bagong |  | 101 |  | 18.498 |  | 18.498 |  | 18.498 |  |
|  |  | Bendot |  | 0 |  | 0.000 |  | 0.000 |  | 18.498 |  |
|  |  | Berani |  | 0 |  | 0.000 |  | 0.000 |  | 18.498 |  |
|  |  | Beth |  | 0 |  | 0.000 |  | 0.000 |  | 18.498 |  |
|  |  | Bibi |  | 0 |  | 0.000 |  | 0.000 |  | 18.498 |  |
|  |  | Bintang |  | 93 |  | 17.033 |  | 17.033 |  | 35.531 |  |
|  |  | Brutus |  | 0 |  | 0.000 |  | 0.000 |  | 35.531 |  |
|  |  | Chindy |  | 0 |  | 0.000 |  | 0.000 |  | 35.531 |  |
|  |  | Codet |  | 0 |  | 0.000 |  | 0.000 |  | 35.531 |  |
|  |  | Elly |  | 0 |  | 0.000 |  | 0.000 |  | 35.531 |  |
|  |  | Fajar |  | 0 |  | 0.000 |  | 0.000 |  | 35.531 |  |
|  |  | Feb |  | 0 |  | 0.000 |  | 0.000 |  | 35.531 |  |
|  |  | Female |  | 0 |  | 0.000 |  | 0.000 |  | 35.531 |  |
|  |  | Fio |  | 0 |  | 0.000 |  | 0.000 |  | 35.531 |  |
|  |  | Flanged male |  | 0 |  | 0.000 |  | 0.000 |  | 35.531 |  |
|  |  | Freddy |  | 0 |  | 0.000 |  | 0.000 |  | 35.531 |  |
|  |  | Friska |  | 0 |  | 0.000 |  | 0.000 |  | 35.531 |  |
|  |  | Fugit |  | 0 |  | 0.000 |  | 0.000 |  | 35.531 |  |
|  |  | Gangstah |  | 0 |  | 0.000 |  | 0.000 |  | 35.531 |  |
|  |  | Gordon |  | 0 |  | 0.000 |  | 0.000 |  | 35.531 |  |
|  |  | Gracia |  | 0 |  | 0.000 |  | 0.000 |  | 35.531 |  |
|  |  | Gretel |  | 0 |  | 0.000 |  | 0.000 |  | 35.531 |  |
|  |  | Henk |  | 0 |  | 0.000 |  | 0.000 |  | 35.531 |  |
|  |  | Icarus |  | 0 |  | 0.000 |  | 0.000 |  | 35.531 |  |
|  |  | Imp |  | 0 |  | 0.000 |  | 0.000 |  | 35.531 |  |
|  |  | Indah |  | 0 |  | 0.000 |  | 0.000 |  | 35.531 |  |
|  |  | Indi |  | 0 |  | 0.000 |  | 0.000 |  | 35.531 |  |
|  |  | Irma |  | 0 |  | 0.000 |  | 0.000 |  | 35.531 |  |
|  |  | James |  | 0 |  | 0.000 |  | 0.000 |  | 35.531 |  |
|  |  | Janda Tua |  | 0 |  | 0.000 |  | 0.000 |  | 35.531 |  |
|  |  | Jinak |  | 0 |  | 0.000 |  | 0.000 |  | 35.531 |  |
|  |  | Joy |  | 0 |  | 0.000 |  | 0.000 |  | 35.531 |  |
|  |  | Juni |  | 0 |  | 0.000 |  | 0.000 |  | 35.531 |  |
|  |  | Juno |  | 0 |  | 0.000 |  | 0.000 |  | 35.531 |  |
|  |  | Kacil |  | 5 |  | 0.916 |  | 0.916 |  | 36.447 |  |
|  |  | Kan |  | 0 |  | 0.000 |  | 0.000 |  | 36.447 |  |
|  |  | Kasi |  | 159 |  | 29.121 |  | 29.121 |  | 65.568 |  |
|  |  | Kay |  | 0 |  | 0.000 |  | 0.000 |  | 65.568 |  |
|  |  | Keri |  | 0 |  | 0.000 |  | 0.000 |  | 65.568 |  |
|  |  | Keto |  | 0 |  | 0.000 |  | 0.000 |  | 65.568 |  |
|  |  | Kondor |  | 0 |  | 0.000 |  | 0.000 |  | 65.568 |  |
|  |  | Kundur |  | 0 |  | 0.000 |  | 0.000 |  | 65.568 |  |
|  |  | Madalena |  | 0 |  | 0.000 |  | 0.000 |  | 65.568 |  |
|  |  | Malé |  | 104 |  | 19.048 |  | 19.048 |  | 84.615 |  |
|  |  | Mindi |  | 0 |  | 0.000 |  | 0.000 |  | 84.615 |  |
|  |  | Ompung |  | 0 |  | 0.000 |  | 0.000 |  | 84.615 |  |
|  |  | Pensi |  | 15 |  | 2.747 |  | 2.747 |  | 87.363 |  |
|  |  | Peot |  | 0 |  | 0.000 |  | 0.000 |  | 87.363 |  |
|  |  | Prabu |  | 0 |  | 0.000 |  | 0.000 |  | 87.363 |  |
|  |  | Raffi |  | 0 |  | 0.000 |  | 0.000 |  | 87.363 |  |
|  |  | Rambo |  | 0 |  | 0.000 |  | 0.000 |  | 87.363 |  |
|  |  | Ronaldo |  | 0 |  | 0.000 |  | 0.000 |  | 87.363 |  |
|  |  | Salvador |  | 0 |  | 0.000 |  | 0.000 |  | 87.363 |  |
|  |  | Suci |  | 0 |  | 0.000 |  | 0.000 |  | 87.363 |  |
|  |  | Sultan |  | 0 |  | 0.000 |  | 0.000 |  | 87.363 |  |
|  |  | Sumi |  | 0 |  | 0.000 |  | 0.000 |  | 87.363 |  |
|  |  | Teju |  | 0 |  | 0.000 |  | 0.000 |  | 87.363 |  |
|  |  | Teresia |  | 0 |  | 0.000 |  | 0.000 |  | 87.363 |  |
|  |  | Timi |  | 0 |  | 0.000 |  | 0.000 |  | 87.363 |  |
|  |  | Tina |  | 0 |  | 0.000 |  | 0.000 |  | 87.363 |  |
|  |  | Travor |  | 0 |  | 0.000 |  | 0.000 |  | 87.363 |  |
|  |  | Umi |  | 0 |  | 0.000 |  | 0.000 |  | 87.363 |  |
|  |  | Unflm |  | 0 |  | 0.000 |  | 0.000 |  | 87.363 |  |
|  |  | Uok |  | 0 |  | 0.000 |  | 0.000 |  | 87.363 |  |
|  |  | Vulcan |  | 0 |  | 0.000 |  | 0.000 |  | 87.363 |  |
|  |  | Walimah |  | 0 |  | 0.000 |  | 0.000 |  | 87.363 |  |
|  |  | Wulan |  | 69 |  | 12.637 |  | 12.637 |  | 100.000 |  |
|  |  | XL |  | 0 |  | 0.000 |  | 0.000 |  | 100.000 |  |
|  |  | Xenix |  | 0 |  | 0.000 |  | 0.000 |  | 100.000 |  |
|  |  | Yanti |  | 0 |  | 0.000 |  | 0.000 |  | 100.000 |  |
|  |  | Zeus |  | 0 |  | 0.000 |  | 0.000 |  | 100.000 |  |
|  |  | Zorro |  | 0 |  | 0.000 |  | 0.000 |  | 100.000 |  |
|  |  | Missing |  | 0 |  | 0.000 |  |  |  |  |  |
|  |  | Total |  | 546 |  | 100.000 |  |  |  |  |  |
| Sikundur |  | Alice |  | 0 |  | 0.000 |  | 0.000 |  | 0.000 |  |
|  |  | Aminah |  | 0 |  | 0.000 |  | 0.000 |  | 0.000 |  |
|  |  | Anto |  | 5 |  | 0.193 |  | 0.193 |  | 0.193 |  |
|  |  | Asny |  | 0 |  | 0.000 |  | 0.000 |  | 0.193 |  |
|  |  | Bagong |  | 0 |  | 0.000 |  | 0.000 |  | 0.193 |  |
|  |  | Bendot |  | 65 |  | 2.511 |  | 2.511 |  | 2.704 |  |
|  |  | Berani |  | 0 |  | 0.000 |  | 0.000 |  | 2.704 |  |
|  |  | Beth |  | 0 |  | 0.000 |  | 0.000 |  | 2.704 |  |
|  |  | Bibi |  | 0 |  | 0.000 |  | 0.000 |  | 2.704 |  |
|  |  | Bintang |  | 0 |  | 0.000 |  | 0.000 |  | 2.704 |  |
|  |  | Brutus |  | 183 |  | 7.068 |  | 7.068 |  | 9.772 |  |
|  |  | Chindy |  | 0 |  | 0.000 |  | 0.000 |  | 9.772 |  |
|  |  | Codet |  | 0 |  | 0.000 |  | 0.000 |  | 9.772 |  |
|  |  | Elly |  | 0 |  | 0.000 |  | 0.000 |  | 9.772 |  |
|  |  | Fajar |  | 0 |  | 0.000 |  | 0.000 |  | 9.772 |  |
|  |  | Feb |  | 0 |  | 0.000 |  | 0.000 |  | 9.772 |  |
|  |  | Female |  | 0 |  | 0.000 |  | 0.000 |  | 9.772 |  |
|  |  | Fio |  | 0 |  | 0.000 |  | 0.000 |  | 9.772 |  |
|  |  | Flanged male |  | 0 |  | 0.000 |  | 0.000 |  | 9.772 |  |
|  |  | Freddy |  | 0 |  | 0.000 |  | 0.000 |  | 9.772 |  |
|  |  | Friska |  | 0 |  | 0.000 |  | 0.000 |  | 9.772 |  |
|  |  | Fugit |  | 0 |  | 0.000 |  | 0.000 |  | 9.772 |  |
|  |  | Gangstah |  | 0 |  | 0.000 |  | 0.000 |  | 9.772 |  |
|  |  | Gordon |  | 0 |  | 0.000 |  | 0.000 |  | 9.772 |  |
|  |  | Gracia |  | 0 |  | 0.000 |  | 0.000 |  | 9.772 |  |
|  |  | Gretel |  | 0 |  | 0.000 |  | 0.000 |  | 9.772 |  |
|  |  | Henk |  | 0 |  | 0.000 |  | 0.000 |  | 9.772 |  |
|  |  | Icarus |  | 0 |  | 0.000 |  | 0.000 |  | 9.772 |  |
|  |  | Imp |  | 0 |  | 0.000 |  | 0.000 |  | 9.772 |  |
|  |  | Indah |  | 0 |  | 0.000 |  | 0.000 |  | 9.772 |  |
|  |  | Indi |  | 0 |  | 0.000 |  | 0.000 |  | 9.772 |  |
|  |  | Irma |  | 1590 |  | 61.414 |  | 61.414 |  | 71.186 |  |
|  |  | James |  | 7 |  | 0.270 |  | 0.270 |  | 71.456 |  |
|  |  | Janda Tua |  | 0 |  | 0.000 |  | 0.000 |  | 71.456 |  |
|  |  | Jinak |  | 0 |  | 0.000 |  | 0.000 |  | 71.456 |  |
|  |  | Joy |  | 0 |  | 0.000 |  | 0.000 |  | 71.456 |  |
|  |  | Juni |  | 0 |  | 0.000 |  | 0.000 |  | 71.456 |  |
|  |  | Juno |  | 0 |  | 0.000 |  | 0.000 |  | 71.456 |  |
|  |  | Kacil |  | 0 |  | 0.000 |  | 0.000 |  | 71.456 |  |
|  |  | Kan |  | 0 |  | 0.000 |  | 0.000 |  | 71.456 |  |
|  |  | Kasi |  | 0 |  | 0.000 |  | 0.000 |  | 71.456 |  |
|  |  | Kay |  | 0 |  | 0.000 |  | 0.000 |  | 71.456 |  |
|  |  | Keri |  | 0 |  | 0.000 |  | 0.000 |  | 71.456 |  |
|  |  | Keto |  | 0 |  | 0.000 |  | 0.000 |  | 71.456 |  |
|  |  | Kondor |  | 0 |  | 0.000 |  | 0.000 |  | 71.456 |  |
|  |  | Kundur |  | 42 |  | 1.622 |  | 1.622 |  | 73.078 |  |
|  |  | Madalena |  | 15 |  | 0.579 |  | 0.579 |  | 73.658 |  |
|  |  | Malé |  | 0 |  | 0.000 |  | 0.000 |  | 73.658 |  |
|  |  | Mindi |  | 0 |  | 0.000 |  | 0.000 |  | 73.658 |  |
|  |  | Ompung |  | 263 |  | 10.158 |  | 10.158 |  | 83.816 |  |
|  |  | Pensi |  | 0 |  | 0.000 |  | 0.000 |  | 83.816 |  |
|  |  | Peot |  | 0 |  | 0.000 |  | 0.000 |  | 83.816 |  |
|  |  | Prabu |  | 0 |  | 0.000 |  | 0.000 |  | 83.816 |  |
|  |  | Raffi |  | 0 |  | 0.000 |  | 0.000 |  | 83.816 |  |
|  |  | Rambo |  | 0 |  | 0.000 |  | 0.000 |  | 83.816 |  |
|  |  | Ronaldo |  | 0 |  | 0.000 |  | 0.000 |  | 83.816 |  |
|  |  | Salvador |  | 0 |  | 0.000 |  | 0.000 |  | 83.816 |  |
|  |  | Suci |  | 5 |  | 0.193 |  | 0.193 |  | 84.009 |  |
|  |  | Sultan |  | 0 |  | 0.000 |  | 0.000 |  | 84.009 |  |
|  |  | Sumi |  | 0 |  | 0.000 |  | 0.000 |  | 84.009 |  |
|  |  | Teju |  | 0 |  | 0.000 |  | 0.000 |  | 84.009 |  |
|  |  | Teresia |  | 0 |  | 0.000 |  | 0.000 |  | 84.009 |  |
|  |  | Timi |  | 0 |  | 0.000 |  | 0.000 |  | 84.009 |  |
|  |  | Tina |  | 0 |  | 0.000 |  | 0.000 |  | 84.009 |  |
|  |  | Travor |  | 0 |  | 0.000 |  | 0.000 |  | 84.009 |  |
|  |  | Umi |  | 0 |  | 0.000 |  | 0.000 |  | 84.009 |  |
|  |  | Unflm |  | 0 |  | 0.000 |  | 0.000 |  | 84.009 |  |
|  |  | Uok |  | 0 |  | 0.000 |  | 0.000 |  | 84.009 |  |
|  |  | Vulcan |  | 0 |  | 0.000 |  | 0.000 |  | 84.009 |  |
|  |  | Walimah |  | 0 |  | 0.000 |  | 0.000 |  | 84.009 |  |
|  |  | Wulan |  | 0 |  | 0.000 |  | 0.000 |  | 84.009 |  |
|  |  | XL |  | 0 |  | 0.000 |  | 0.000 |  | 84.009 |  |
|  |  | Xenix |  | 0 |  | 0.000 |  | 0.000 |  | 84.009 |  |
|  |  | Yanti |  | 414 |  | 15.991 |  | 15.991 |  | 100.000 |  |
|  |  | Zeus |  | 0 |  | 0.000 |  | 0.000 |  | 100.000 |  |
|  |  | Zorro |  | 0 |  | 0.000 |  | 0.000 |  | 100.000 |  |
|  |  | Missing |  | 0 |  | 0.000 |  |  |  |  |  |
|  |  | Total |  | 2589 |  | 100.000 |  |  |  |  |  |
| Suaq |  | Alice |  | 20 |  | 3.656 |  | 3.656 |  | 3.656 |  |
|  |  | Aminah |  | 0 |  | 0.000 |  | 0.000 |  | 3.656 |  |
|  |  | Anto |  | 0 |  | 0.000 |  | 0.000 |  | 3.656 |  |
|  |  | Asny |  | 0 |  | 0.000 |  | 0.000 |  | 3.656 |  |
|  |  | Bagong |  | 0 |  | 0.000 |  | 0.000 |  | 3.656 |  |
|  |  | Bendot |  | 0 |  | 0.000 |  | 0.000 |  | 3.656 |  |
|  |  | Berani |  | 0 |  | 0.000 |  | 0.000 |  | 3.656 |  |
|  |  | Beth |  | 0 |  | 0.000 |  | 0.000 |  | 3.656 |  |
|  |  | Bibi |  | 0 |  | 0.000 |  | 0.000 |  | 3.656 |  |
|  |  | Bintang |  | 0 |  | 0.000 |  | 0.000 |  | 3.656 |  |
|  |  | Brutus |  | 0 |  | 0.000 |  | 0.000 |  | 3.656 |  |
|  |  | Chindy |  | 22 |  | 4.022 |  | 4.022 |  | 7.678 |  |
|  |  | Codet |  | 0 |  | 0.000 |  | 0.000 |  | 7.678 |  |
|  |  | Elly |  | 51 |  | 9.324 |  | 9.324 |  | 17.002 |  |
|  |  | Fajar |  | 0 |  | 0.000 |  | 0.000 |  | 17.002 |  |
|  |  | Feb |  | 0 |  | 0.000 |  | 0.000 |  | 17.002 |  |
|  |  | Female |  | 0 |  | 0.000 |  | 0.000 |  | 17.002 |  |
|  |  | Fio |  | 0 |  | 0.000 |  | 0.000 |  | 17.002 |  |
|  |  | Flanged male |  | 0 |  | 0.000 |  | 0.000 |  | 17.002 |  |
|  |  | Freddy |  | 1 |  | 0.183 |  | 0.183 |  | 17.185 |  |
|  |  | Friska |  | 1 |  | 0.183 |  | 0.183 |  | 17.367 |  |
|  |  | Fugit |  | 0 |  | 0.000 |  | 0.000 |  | 17.367 |  |
|  |  | Gangstah |  | 17 |  | 3.108 |  | 3.108 |  | 20.475 |  |
|  |  | Gordon |  | 0 |  | 0.000 |  | 0.000 |  | 20.475 |  |
|  |  | Gracia |  | 0 |  | 0.000 |  | 0.000 |  | 20.475 |  |
|  |  | Gretel |  | 0 |  | 0.000 |  | 0.000 |  | 20.475 |  |
|  |  | Henk |  | 0 |  | 0.000 |  | 0.000 |  | 20.475 |  |
|  |  | Icarus |  | 0 |  | 0.000 |  | 0.000 |  | 20.475 |  |
|  |  | Imp |  | 49 |  | 8.958 |  | 8.958 |  | 29.433 |  |
|  |  | Indah |  | 0 |  | 0.000 |  | 0.000 |  | 29.433 |  |
|  |  | Indi |  | 0 |  | 0.000 |  | 0.000 |  | 29.433 |  |
|  |  | Irma |  | 0 |  | 0.000 |  | 0.000 |  | 29.433 |  |
|  |  | James |  | 0 |  | 0.000 |  | 0.000 |  | 29.433 |  |
|  |  | Janda Tua |  | 0 |  | 0.000 |  | 0.000 |  | 29.433 |  |
|  |  | Jinak |  | 0 |  | 0.000 |  | 0.000 |  | 29.433 |  |
|  |  | Joy |  | 0 |  | 0.000 |  | 0.000 |  | 29.433 |  |
|  |  | Juni |  | 0 |  | 0.000 |  | 0.000 |  | 29.433 |  |
|  |  | Juno |  | 0 |  | 0.000 |  | 0.000 |  | 29.433 |  |
|  |  | Kacil |  | 0 |  | 0.000 |  | 0.000 |  | 29.433 |  |
|  |  | Kan |  | 0 |  | 0.000 |  | 0.000 |  | 29.433 |  |
|  |  | Kasi |  | 0 |  | 0.000 |  | 0.000 |  | 29.433 |  |
|  |  | Kay |  | 0 |  | 0.000 |  | 0.000 |  | 29.433 |  |
|  |  | Keri |  | 0 |  | 0.000 |  | 0.000 |  | 29.433 |  |
|  |  | Keto |  | 0 |  | 0.000 |  | 0.000 |  | 29.433 |  |
|  |  | Kondor |  | 0 |  | 0.000 |  | 0.000 |  | 29.433 |  |
|  |  | Kundur |  | 0 |  | 0.000 |  | 0.000 |  | 29.433 |  |
|  |  | Madalena |  | 0 |  | 0.000 |  | 0.000 |  | 29.433 |  |
|  |  | Malé |  | 0 |  | 0.000 |  | 0.000 |  | 29.433 |  |
|  |  | Mindi |  | 0 |  | 0.000 |  | 0.000 |  | 29.433 |  |
|  |  | Ompung |  | 0 |  | 0.000 |  | 0.000 |  | 29.433 |  |
|  |  | Pensi |  | 0 |  | 0.000 |  | 0.000 |  | 29.433 |  |
|  |  | Peot |  | 0 |  | 0.000 |  | 0.000 |  | 29.433 |  |
|  |  | Prabu |  | 0 |  | 0.000 |  | 0.000 |  | 29.433 |  |
|  |  | Raffi |  | 61 |  | 11.152 |  | 11.152 |  | 40.585 |  |
|  |  | Rambo |  | 0 |  | 0.000 |  | 0.000 |  | 40.585 |  |
|  |  | Ronaldo |  | 1 |  | 0.183 |  | 0.183 |  | 40.768 |  |
|  |  | Salvador |  | 0 |  | 0.000 |  | 0.000 |  | 40.768 |  |
|  |  | Suci |  | 0 |  | 0.000 |  | 0.000 |  | 40.768 |  |
|  |  | Sultan |  | 0 |  | 0.000 |  | 0.000 |  | 40.768 |  |
|  |  | Sumi |  | 0 |  | 0.000 |  | 0.000 |  | 40.768 |  |
|  |  | Teju |  | 0 |  | 0.000 |  | 0.000 |  | 40.768 |  |
|  |  | Teresia |  | 0 |  | 0.000 |  | 0.000 |  | 40.768 |  |
|  |  | Timi |  | 0 |  | 0.000 |  | 0.000 |  | 40.768 |  |
|  |  | Tina |  | 316 |  | 57.770 |  | 57.770 |  | 98.537 |  |
|  |  | Travor |  | 0 |  | 0.000 |  | 0.000 |  | 98.537 |  |
|  |  | Umi |  | 0 |  | 0.000 |  | 0.000 |  | 98.537 |  |
|  |  | Unflm |  | 0 |  | 0.000 |  | 0.000 |  | 98.537 |  |
|  |  | Uok |  | 0 |  | 0.000 |  | 0.000 |  | 98.537 |  |
|  |  | Vulcan |  | 0 |  | 0.000 |  | 0.000 |  | 98.537 |  |
|  |  | Walimah |  | 0 |  | 0.000 |  | 0.000 |  | 98.537 |  |
|  |  | Wulan |  | 0 |  | 0.000 |  | 0.000 |  | 98.537 |  |
|  |  | XL |  | 0 |  | 0.000 |  | 0.000 |  | 98.537 |  |
|  |  | Xenix |  | 8 |  | 1.463 |  | 1.463 |  | 100.000 |  |
|  |  | Yanti |  | 0 |  | 0.000 |  | 0.000 |  | 100.000 |  |
|  |  | Zeus |  | 0 |  | 0.000 |  | 0.000 |  | 100.000 |  |
|  |  | Zorro |  | 0 |  | 0.000 |  | 0.000 |  | 100.000 |  |
|  |  | Missing |  | 0 |  | 0.000 |  |  |  |  |  |
|  |  | Total |  | 547 |  | 100.000 |  |  |  |  |  |
| Tuanan |  | Alice |  | 0 |  | 0.000 |  | 0.000 |  | 0.000 |  |
|  |  | Aminah |  | 0 |  | 0.000 |  | 0.000 |  | 0.000 |  |
|  |  | Anto |  | 0 |  | 0.000 |  | 0.000 |  | 0.000 |  |
|  |  | Asny |  | 0 |  | 0.000 |  | 0.000 |  | 0.000 |  |
|  |  | Bagong |  | 0 |  | 0.000 |  | 0.000 |  | 0.000 |  |
|  |  | Bendot |  | 0 |  | 0.000 |  | 0.000 |  | 0.000 |  |
|  |  | Berani |  | 0 |  | 0.000 |  | 0.000 |  | 0.000 |  |
|  |  | Beth |  | 0 |  | 0.000 |  | 0.000 |  | 0.000 |  |
|  |  | Bibi |  | 0 |  | 0.000 |  | 0.000 |  | 0.000 |  |
|  |  | Bintang |  | 0 |  | 0.000 |  | 0.000 |  | 0.000 |  |
|  |  | Brutus |  | 0 |  | 0.000 |  | 0.000 |  | 0.000 |  |
|  |  | Chindy |  | 0 |  | 0.000 |  | 0.000 |  | 0.000 |  |
|  |  | Codet |  | 0 |  | 0.000 |  | 0.000 |  | 0.000 |  |
|  |  | Elly |  | 0 |  | 0.000 |  | 0.000 |  | 0.000 |  |
|  |  | Fajar |  | 0 |  | 0.000 |  | 0.000 |  | 0.000 |  |
|  |  | Feb |  | 0 |  | 0.000 |  | 0.000 |  | 0.000 |  |
|  |  | Female |  | 0 |  | 0.000 |  | 0.000 |  | 0.000 |  |
|  |  | Fio |  | 0 |  | 0.000 |  | 0.000 |  | 0.000 |  |
|  |  | Flanged male |  | 6 |  | 1.107 |  | 1.107 |  | 1.107 |  |
|  |  | Freddy |  | 0 |  | 0.000 |  | 0.000 |  | 1.107 |  |
|  |  | Friska |  | 0 |  | 0.000 |  | 0.000 |  | 1.107 |  |
|  |  | Fugit |  | 126 |  | 23.247 |  | 23.247 |  | 24.354 |  |
|  |  | Gangstah |  | 0 |  | 0.000 |  | 0.000 |  | 24.354 |  |
|  |  | Gordon |  | 0 |  | 0.000 |  | 0.000 |  | 24.354 |  |
|  |  | Gracia |  | 0 |  | 0.000 |  | 0.000 |  | 24.354 |  |
|  |  | Gretel |  | 0 |  | 0.000 |  | 0.000 |  | 24.354 |  |
|  |  | Henk |  | 4 |  | 0.738 |  | 0.738 |  | 25.092 |  |
|  |  | Icarus |  | 0 |  | 0.000 |  | 0.000 |  | 25.092 |  |
|  |  | Imp |  | 0 |  | 0.000 |  | 0.000 |  | 25.092 |  |
|  |  | Indah |  | 0 |  | 0.000 |  | 0.000 |  | 25.092 |  |
|  |  | Indi |  | 0 |  | 0.000 |  | 0.000 |  | 25.092 |  |
|  |  | Irma |  | 0 |  | 0.000 |  | 0.000 |  | 25.092 |  |
|  |  | James |  | 0 |  | 0.000 |  | 0.000 |  | 25.092 |  |
|  |  | Janda Tua |  | 0 |  | 0.000 |  | 0.000 |  | 25.092 |  |
|  |  | Jinak |  | 41 |  | 7.565 |  | 7.565 |  | 32.657 |  |
|  |  | Joy |  | 0 |  | 0.000 |  | 0.000 |  | 32.657 |  |
|  |  | Juni |  | 4 |  | 0.738 |  | 0.738 |  | 33.395 |  |
|  |  | Juno |  | 0 |  | 0.000 |  | 0.000 |  | 33.395 |  |
|  |  | Kacil |  | 0 |  | 0.000 |  | 0.000 |  | 33.395 |  |
|  |  | Kan |  | 0 |  | 0.000 |  | 0.000 |  | 33.395 |  |
|  |  | Kasi |  | 0 |  | 0.000 |  | 0.000 |  | 33.395 |  |
|  |  | Kay |  | 111 |  | 20.480 |  | 20.480 |  | 53.875 |  |
|  |  | Keri |  | 6 |  | 1.107 |  | 1.107 |  | 54.982 |  |
|  |  | Keto |  | 0 |  | 0.000 |  | 0.000 |  | 54.982 |  |
|  |  | Kondor |  | 38 |  | 7.011 |  | 7.011 |  | 61.993 |  |
|  |  | Kundur |  | 0 |  | 0.000 |  | 0.000 |  | 61.993 |  |
|  |  | Madalena |  | 0 |  | 0.000 |  | 0.000 |  | 61.993 |  |
|  |  | Malé |  | 0 |  | 0.000 |  | 0.000 |  | 61.993 |  |
|  |  | Mindi |  | 17 |  | 3.137 |  | 3.137 |  | 65.129 |  |
|  |  | Ompung |  | 0 |  | 0.000 |  | 0.000 |  | 65.129 |  |
|  |  | Pensi |  | 0 |  | 0.000 |  | 0.000 |  | 65.129 |  |
|  |  | Peot |  | 0 |  | 0.000 |  | 0.000 |  | 65.129 |  |
|  |  | Prabu |  | 0 |  | 0.000 |  | 0.000 |  | 65.129 |  |
|  |  | Raffi |  | 0 |  | 0.000 |  | 0.000 |  | 65.129 |  |
|  |  | Rambo |  | 29 |  | 5.351 |  | 5.351 |  | 70.480 |  |
|  |  | Ronaldo |  | 0 |  | 0.000 |  | 0.000 |  | 70.480 |  |
|  |  | Salvador |  | 0 |  | 0.000 |  | 0.000 |  | 70.480 |  |
|  |  | Suci |  | 0 |  | 0.000 |  | 0.000 |  | 70.480 |  |
|  |  | Sultan |  | 106 |  | 19.557 |  | 19.557 |  | 90.037 |  |
|  |  | Sumi |  | 32 |  | 5.904 |  | 5.904 |  | 95.941 |  |
|  |  | Teju |  | 22 |  | 4.059 |  | 4.059 |  | 100.000 |  |
|  |  | Teresia |  | 0 |  | 0.000 |  | 0.000 |  | 100.000 |  |
|  |  | Timi |  | 0 |  | 0.000 |  | 0.000 |  | 100.000 |  |
|  |  | Tina |  | 0 |  | 0.000 |  | 0.000 |  | 100.000 |  |
|  |  | Travor |  | 0 |  | 0.000 |  | 0.000 |  | 100.000 |  |
|  |  | Umi |  | 0 |  | 0.000 |  | 0.000 |  | 100.000 |  |
|  |  | Unflm |  | 0 |  | 0.000 |  | 0.000 |  | 100.000 |  |
|  |  | Uok |  | 0 |  | 0.000 |  | 0.000 |  | 100.000 |  |
|  |  | Vulcan |  | 0 |  | 0.000 |  | 0.000 |  | 100.000 |  |
|  |  | Walimah |  | 0 |  | 0.000 |  | 0.000 |  | 100.000 |  |
|  |  | Wulan |  | 0 |  | 0.000 |  | 0.000 |  | 100.000 |  |
|  |  | XL |  | 0 |  | 0.000 |  | 0.000 |  | 100.000 |  |
|  |  | Xenix |  | 0 |  | 0.000 |  | 0.000 |  | 100.000 |  |
|  |  | Yanti |  | 0 |  | 0.000 |  | 0.000 |  | 100.000 |  |
|  |  | Zeus |  | 0 |  | 0.000 |  | 0.000 |  | 100.000 |  |
|  |  | Zorro |  | 0 |  | 0.000 |  | 0.000 |  | 100.000 |  |
|  |  | Missing |  | 0 |  | 0.000 |  |  |  |  |  |
|  |  | Total |  | 542 |  | 100.000 |  |  |  |  |  |
|  | | | | | | | | | | | |

| Frequencies for gender | | | | | | | | | | | |
| --- | --- | --- | --- | --- | --- | --- | --- | --- | --- | --- | --- |
| population | | gender | | Frequency | | Percent | | Valid Percent | | Cumulative Percent | |
| Gunung Palung |  | female |  | 301 |  | 36.978 |  | 36.978 |  | 36.978 |  |
|  |  | male |  | 513 |  | 63.022 |  | 63.022 |  | 100.000 |  |
|  |  | Missing |  | 0 |  | 0.000 |  |  |  |  |  |
|  |  | Total |  | 814 |  | 100.000 |  |  |  |  |  |
| Sabangau |  | female |  | 160 |  | 62.257 |  | 62.257 |  | 62.257 |  |
|  |  | male |  | 97 |  | 37.743 |  | 37.743 |  | 100.000 |  |
|  |  | Missing |  | 0 |  | 0.000 |  |  |  |  |  |
|  |  | Total |  | 257 |  | 100.000 |  |  |  |  |  |
| Sampan Getek |  | female |  | 445 |  | 81.502 |  | 81.502 |  | 81.502 |  |
|  |  | male |  | 101 |  | 18.498 |  | 18.498 |  | 100.000 |  |
|  |  | Missing |  | 0 |  | 0.000 |  |  |  |  |  |
|  |  | Total |  | 546 |  | 100.000 |  |  |  |  |  |
| Sikundur |  | female |  | 2024 |  | 78.177 |  | 78.177 |  | 78.177 |  |
|  |  | male |  | 565 |  | 21.823 |  | 21.823 |  | 100.000 |  |
|  |  | Missing |  | 0 |  | 0.000 |  |  |  |  |  |
|  |  | Total |  | 2589 |  | 100.000 |  |  |  |  |  |
| Suaq |  | female |  | 471 |  | 86.106 |  | 86.106 |  | 86.106 |  |
|  |  | male |  | 76 |  | 13.894 |  | 13.894 |  | 100.000 |  |
|  |  | Missing |  | 0 |  | 0.000 |  |  |  |  |  |
|  |  | Total |  | 547 |  | 100.000 |  |  |  |  |  |
| Tuanan |  | female |  | 139 |  | 25.646 |  | 25.646 |  | 25.646 |  |
|  |  | male |  | 403 |  | 74.354 |  | 74.354 |  | 100.000 |  |
|  |  | Missing |  | 0 |  | 0.000 |  |  |  |  |  |
|  |  | Total |  | 542 |  | 100.000 |  |  |  |  |  |
|  | | | | | | | | | | | |

| Frequencies for age-sex class | | | | | | | | | | | |
| --- | --- | --- | --- | --- | --- | --- | --- | --- | --- | --- | --- |
| population | | age-sex class | | Frequency | | Percent | | Valid Percent | | Cumulative Percent | |
| Gunung Palung |  | adolescent |  | 134 |  | 16.462 |  | 16.462 |  | 16.462 |  |
|  |  | female with infant |  | 99 |  | 12.162 |  | 12.162 |  | 28.624 |  |
|  |  | flanged male |  | 399 |  | 49.017 |  | 49.017 |  | 77.641 |  |
|  |  | infant |  | 119 |  | 14.619 |  | 14.619 |  | 92.260 |  |
|  |  | unflanged male |  | 63 |  | 7.740 |  | 7.740 |  | 100.000 |  |
|  |  | Missing |  | 0 |  | 0.000 |  |  |  |  |  |
|  |  | Total |  | 814 |  | 100.000 |  |  |  |  |  |
| Sabangau |  | adolescent |  | 58 |  | 22.568 |  | 22.568 |  | 22.568 |  |
|  |  | female with infant |  | 100 |  | 38.911 |  | 38.911 |  | 61.479 |  |
|  |  | flanged male |  | 49 |  | 19.066 |  | 19.066 |  | 80.545 |  |
|  |  | infant |  | 17 |  | 6.615 |  | 6.615 |  | 87.160 |  |
|  |  | unflanged male |  | 33 |  | 12.840 |  | 12.840 |  | 100.000 |  |
|  |  | Missing |  | 0 |  | 0.000 |  |  |  |  |  |
|  |  | Total |  | 257 |  | 100.000 |  |  |  |  |  |
| Sampan Getek |  | adolescent |  | 69 |  | 12.637 |  | 12.637 |  | 12.637 |  |
|  |  | female with infant |  | 371 |  | 67.949 |  | 67.949 |  | 80.586 |  |
|  |  | flanged male |  | 101 |  | 18.498 |  | 18.498 |  | 99.084 |  |
|  |  | infant |  | 5 |  | 0.916 |  | 0.916 |  | 100.000 |  |
|  |  | unflanged male |  | 0 |  | 0.000 |  | 0.000 |  | 100.000 |  |
|  |  | Missing |  | 0 |  | 0.000 |  |  |  |  |  |
|  |  | Total |  | 546 |  | 100.000 |  |  |  |  |  |
| Sikundur |  | adolescent |  | 0 |  | 0.000 |  | 0.000 |  | 0.000 |  |
|  |  | female with infant |  | 2023 |  | 78.138 |  | 78.138 |  | 78.138 |  |
|  |  | flanged male |  | 329 |  | 12.708 |  | 12.708 |  | 90.846 |  |
|  |  | infant |  | 0 |  | 0.000 |  | 0.000 |  | 90.846 |  |
|  |  | unflanged male |  | 237 |  | 9.154 |  | 9.154 |  | 100.000 |  |
|  |  | Missing |  | 0 |  | 0.000 |  |  |  |  |  |
|  |  | Total |  | 2589 |  | 100.000 |  |  |  |  |  |
| Suaq |  | adolescent |  | 389 |  | 71.115 |  | 71.115 |  | 71.115 |  |
|  |  | female with infant |  | 82 |  | 14.991 |  | 14.991 |  | 86.106 |  |
|  |  | flanged male |  | 8 |  | 1.463 |  | 1.463 |  | 87.569 |  |
|  |  | infant |  | 2 |  | 0.366 |  | 0.366 |  | 87.934 |  |
|  |  | unflanged male |  | 66 |  | 12.066 |  | 12.066 |  | 100.000 |  |
|  |  | Missing |  | 0 |  | 0.000 |  |  |  |  |  |
|  |  | Total |  | 547 |  | 100.000 |  |  |  |  |  |
| Tuanan |  | adolescent |  | 4 |  | 0.738 |  | 0.738 |  | 0.738 |  |
|  |  | female with infant |  | 96 |  | 17.712 |  | 17.712 |  | 18.450 |  |
|  |  | flanged male |  | 404 |  | 74.539 |  | 74.539 |  | 92.989 |  |
|  |  | infant |  | 38 |  | 7.011 |  | 7.011 |  | 100.000 |  |
|  |  | unflanged male |  | 0 |  | 0.000 |  | 0.000 |  | 100.000 |  |
|  |  | Missing |  | 0 |  | 0.000 |  |  |  |  |  |
|  |  | Total |  | 542 |  | 100.000 |  |  |  |  |  |
|  | | | | | | | | | | | |

| Frequencies for context | | | | | | | | | | | |
| --- | --- | --- | --- | --- | --- | --- | --- | --- | --- | --- | --- |
| population | | context | | Frequency | | Percent | | Valid Percent | | Cumulative Percent | |
| Gunung Palung |  | no apparent danger |  | 31 |  | 3.808 |  | 3.808 |  | 3.808 |  |
|  |  | towards animals |  | 0 |  | 0.000 |  | 0.000 |  | 3.808 |  |
|  |  | towards humans (non-observers) |  | 0 |  | 0.000 |  | 0.000 |  | 3.808 |  |
|  |  | towards observers |  | 780 |  | 95.823 |  | 95.823 |  | 99.631 |  |
|  |  | towards other orangutans |  | 3 |  | 0.369 |  | 0.369 |  | 100.000 |  |
|  |  | Missing |  | 0 |  | 0.000 |  |  |  |  |  |
|  |  | Total |  | 814 |  | 100.000 |  |  |  |  |  |
| Sabangau |  | no apparent danger |  | 0 |  | 0.000 |  | 0.000 |  | 0.000 |  |
|  |  | towards animals |  | 7 |  | 2.724 |  | 2.724 |  | 2.724 |  |
|  |  | towards humans (non-observers) |  | 0 |  | 0.000 |  | 0.000 |  | 2.724 |  |
|  |  | towards observers |  | 184 |  | 71.595 |  | 71.595 |  | 74.319 |  |
|  |  | towards other orangutans |  | 66 |  | 25.681 |  | 25.681 |  | 100.000 |  |
|  |  | Missing |  | 0 |  | 0.000 |  |  |  |  |  |
|  |  | Total |  | 257 |  | 100.000 |  |  |  |  |  |
| Sampan Getek |  | no apparent danger |  | 0 |  | 0.000 |  | 0.000 |  | 0.000 |  |
|  |  | towards animals |  | 0 |  | 0.000 |  | 0.000 |  | 0.000 |  |
|  |  | towards humans (non-observers) |  | 0 |  | 0.000 |  | 0.000 |  | 0.000 |  |
|  |  | towards observers |  | 546 |  | 100.000 |  | 100.000 |  | 100.000 |  |
|  |  | towards other orangutans |  | 0 |  | 0.000 |  | 0.000 |  | 100.000 |  |
|  |  | Missing |  | 0 |  | 0.000 |  |  |  |  |  |
|  |  | Total |  | 546 |  | 100.000 |  |  |  |  |  |
| Sikundur |  | no apparent danger |  | 0 |  | 0.000 |  | 0.000 |  | 0.000 |  |
|  |  | towards animals |  | 35 |  | 1.352 |  | 1.352 |  | 1.352 |  |
|  |  | towards humans (non-observers) |  | 22 |  | 0.850 |  | 0.850 |  | 2.202 |  |
|  |  | towards observers |  | 2532 |  | 97.798 |  | 97.798 |  | 100.000 |  |
|  |  | towards other orangutans |  | 0 |  | 0.000 |  | 0.000 |  | 100.000 |  |
|  |  | Missing |  | 0 |  | 0.000 |  |  |  |  |  |
|  |  | Total |  | 2589 |  | 100.000 |  |  |  |  |  |
| Suaq |  | no apparent danger |  | 0 |  | 0.000 |  | 0.000 |  | 0.000 |  |
|  |  | towards animals |  | 10 |  | 1.828 |  | 1.828 |  | 1.828 |  |
|  |  | towards humans (non-observers) |  | 0 |  | 0.000 |  | 0.000 |  | 1.828 |  |
|  |  | towards observers |  | 515 |  | 94.150 |  | 94.150 |  | 95.978 |  |
|  |  | towards other orangutans |  | 22 |  | 4.022 |  | 4.022 |  | 100.000 |  |
|  |  | Missing |  | 0 |  | 0.000 |  |  |  |  |  |
|  |  | Total |  | 547 |  | 100.000 |  |  |  |  |  |
| Tuanan |  | no apparent danger |  | 0 |  | 0.000 |  | 0.000 |  | 0.000 |  |
|  |  | towards animals |  | 45 |  | 8.303 |  | 8.364 |  | 8.364 |  |
|  |  | towards humans (non-observers) |  | 0 |  | 0.000 |  | 0.000 |  | 8.364 |  |
|  |  | towards observers |  | 367 |  | 67.712 |  | 68.216 |  | 76.580 |  |
|  |  | towards other orangutans |  | 126 |  | 23.247 |  | 23.420 |  | 100.000 |  |
|  |  | Missing |  | 4 |  | 0.738 |  |  |  |  |  |
|  |  | Total |  | 542 |  | 100.000 |  |  |  |  |  |
|  | | | | | | | | | | | |

## Descriptive Statistics, split by Individual

| Descriptive Statistics | | | | | | | | | | | | | | | | | | | | | | | | | | | | | | | | | | | | | | | | | | | | | | | | | | | | | | | | | | | | | | | | | | | | | | | | | | | | | | | | | | | | | | | | | | | | | | | | | | | | | | | | | | | | | | | | | | | | | | | | | | | | | | | | | | | | | | | | | | | | | | | | | | | | | | | | | | | | | | | | | | | | | | | | | | | | | | | | | | | | | | | | | | | | | | | | | | | | | | | | | | | | | | | | | | | | | | | | | | | | | | | | | | | | | | | | | | | | | | | | | | | | | | | | | | | | | | | | | | | | | | | | | | | | | | | | | | | | | | | | | | | | | | | | | | | | | | | | | | | | | | | | | | | | | | | | | | | | | | | | | | | | | | | | | | | | | | | | | | | | | | | | | | | | | | | | | | | | | | | | | | | | | | | | | | | | | | | | | | | | | | | | | | | | | | | | | | | | | | | | | | | | | | | | | | | | | | | | | | | | | | | | | | | | | | | | | | | | | | | | | | | | | | | | | | | | | | | | | | | | | | | | | | | | | | | | | | | | | | | | | | | | | | | | | | | | | | | | | | | | | | | | | | | | | | | | | | | | | | | | | | | | | | | | | | | | | | | | | | | | | | | | | | | | | | | | | | | | | | | | | | | | | | | | | | | | | | | | | | | | | | | | | | | | | | | | | | | | | | | | | | | | | | | | | | | | | | | | | | | | | | | | | | | | | | | | | | | | | | | | | | | | | | | | | | | | | | | | | | | | | | | | | | | | | | | | | | | | | | | | | | | | | | | | | | | | | | | | | | | | | | | | | | | | | | | | | | | | | | | | | | | | | | | | | | | | | | | | | | | | | | | | | | | | | | | | | | | | | | | | | |
| --- | --- | --- | --- | --- | --- | --- | --- | --- | --- | --- | --- | --- | --- | --- | --- | --- | --- | --- | --- | --- | --- | --- | --- | --- | --- | --- | --- | --- | --- | --- | --- | --- | --- | --- | --- | --- | --- | --- | --- | --- | --- | --- | --- | --- | --- | --- | --- | --- | --- | --- | --- | --- | --- | --- | --- | --- | --- | --- | --- | --- | --- | --- | --- | --- | --- | --- | --- | --- | --- | --- | --- | --- | --- | --- | --- | --- | --- | --- | --- | --- | --- | --- | --- | --- | --- | --- | --- | --- | --- | --- | --- | --- | --- | --- | --- | --- | --- | --- | --- | --- | --- | --- | --- | --- | --- | --- | --- | --- | --- | --- | --- | --- | --- | --- | --- | --- | --- | --- | --- | --- | --- | --- | --- | --- | --- | --- | --- | --- | --- | --- | --- | --- | --- | --- | --- | --- | --- | --- | --- | --- | --- | --- | --- | --- | --- | --- | --- | --- | --- | --- | --- | --- | --- | --- | --- | --- | --- | --- | --- | --- | --- | --- | --- | --- | --- | --- | --- | --- | --- | --- | --- | --- | --- | --- | --- | --- | --- | --- | --- | --- | --- | --- | --- | --- | --- | --- | --- | --- | --- | --- | --- | --- | --- | --- | --- | --- | --- | --- | --- | --- | --- | --- | --- | --- | --- | --- | --- | --- | --- | --- | --- | --- | --- | --- | --- | --- | --- | --- | --- | --- | --- | --- | --- | --- | --- | --- | --- | --- | --- | --- | --- | --- | --- | --- | --- | --- | --- | --- | --- | --- | --- | --- | --- | --- | --- | --- | --- | --- | --- | --- | --- | --- | --- | --- | --- | --- | --- | --- | --- | --- | --- | --- | --- | --- | --- | --- | --- | --- | --- | --- | --- | --- | --- | --- | --- | --- | --- | --- | --- | --- | --- | --- | --- | --- | --- | --- | --- | --- | --- | --- | --- | --- | --- | --- | --- | --- | --- | --- | --- | --- | --- | --- | --- | --- | --- | --- | --- | --- | --- | --- | --- | --- | --- | --- | --- | --- | --- | --- | --- | --- | --- | --- | --- | --- | --- | --- | --- | --- | --- | --- | --- | --- | --- | --- | --- | --- | --- | --- | --- | --- | --- | --- | --- | --- | --- | --- | --- | --- | --- | --- | --- | --- | --- | --- | --- | --- | --- | --- | --- | --- | --- | --- | --- | --- | --- | --- | --- | --- | --- | --- | --- | --- | --- | --- | --- | --- | --- | --- | --- | --- | --- | --- | --- | --- | --- | --- | --- | --- | --- | --- | --- | --- | --- | --- | --- | --- | --- | --- | --- | --- | --- | --- | --- | --- | --- | --- | --- | --- | --- | --- | --- | --- | --- | --- | --- | --- | --- | --- | --- | --- | --- | --- | --- | --- | --- | --- | --- | --- | --- | --- | --- | --- | --- | --- | --- | --- | --- | --- | --- | --- | --- | --- | --- | --- | --- | --- | --- | --- | --- | --- | --- | --- | --- | --- | --- | --- | --- | --- | --- | --- | --- | --- | --- | --- | --- | --- | --- | --- | --- | --- | --- | --- | --- | --- | --- | --- | --- | --- | --- | --- | --- | --- | --- | --- | --- | --- | --- | --- | --- | --- | --- | --- | --- | --- | --- | --- | --- | --- | --- | --- | --- | --- | --- | --- | --- | --- | --- | --- | --- | --- | --- | --- | --- | --- | --- | --- | --- | --- | --- | --- | --- | --- | --- | --- | --- | --- | --- | --- | --- | --- | --- | --- | --- | --- | --- | --- | --- | --- | --- | --- | --- | --- | --- | --- | --- | --- | --- | --- | --- | --- | --- | --- | --- | --- | --- | --- | --- | --- | --- | --- | --- | --- | --- | --- | --- | --- | --- | --- | --- | --- | --- | --- | --- | --- | --- | --- | --- | --- | --- | --- | --- | --- | --- | --- | --- | --- | --- | --- | --- | --- | --- | --- | --- | --- | --- | --- | --- | --- | --- | --- | --- | --- | --- | --- | --- | --- | --- | --- | --- | --- | --- | --- | --- | --- | --- | --- | --- | --- | --- | --- | --- | --- | --- | --- | --- | --- | --- | --- | --- | --- | --- | --- | --- | --- | --- | --- | --- | --- | --- | --- | --- | --- | --- | --- | --- | --- | --- | --- | --- | --- | --- | --- | --- | --- | --- | --- | --- | --- | --- | --- | --- | --- | --- | --- | --- | --- | --- | --- | --- | --- | --- | --- | --- | --- | --- | --- | --- | --- | --- | --- | --- | --- | --- | --- | --- | --- | --- | --- | --- | --- | --- | --- | --- | --- | --- | --- | --- | --- | --- | --- | --- | --- | --- | --- | --- | --- | --- | --- | --- | --- | --- | --- | --- | --- | --- | --- | --- | --- | --- | --- | --- | --- | --- | --- | --- | --- | --- | --- | --- | --- | --- | --- | --- | --- | --- | --- | --- | --- | --- | --- | --- | --- | --- | --- | --- | --- | --- | --- | --- | --- | --- | --- | --- | --- | --- | --- | --- | --- | --- | --- | --- |
|  | | gender | | | | | | | | | | | | | | | | | | | | | | | | | | | | | | | | | | | | | | | | | | | | | | | | | | | | | | | | | | | | | | | | | | | | | | | | | | | | | | | | | | | | | | | | | | | | | | | | | | | | | | | | | | | | | | | | | | | | | | | | | | | | | | | | | | | | | | | | | | | | | | | | | | | | | | | | age-sex class | | | | | | | | | | | | | | | | | | | | | | | | | | | | | | | | | | | | | | | | | | | | | | | | | | | | | | | | | | | | | | | | | | | | | | | | | | | | | | | | | | | | | | | | | | | | | | | | | | | | | | | | | | | | | | | | | | | | | | | | | | | | | | | | | | | | | | | | | | | | | | | | | | | | | | | | context | | | | | | | | | | | | | | | | | | | | | | | | | | | | | | | | | | | | | | | | | | | | | | | | | | | | | | | | | | | | | | | | | | | | | | | | | | | | | | | | | | | | | | | | | | | | | | | | | | | | | | | | | | | | | | | | | | | | | | | | | | | | | | | | | | | | | | | | | | | | | | | | | | | | | | | | max freq | | | | | | | | | | | | | | | | | | | | | | | | | | | | | | | | | | | | | | | | | | | | | | | | | | | | | | | | | | | | | | | | | | | | | | | | | | | | | | | | | | | | | | | | | | | | | | | | | | | | | | | | | | | | | | | | | | | | | | | | | | | | | | | | | | | | | | | | | | | | | | | | | | | | | | | | duration | | | | | | | | | | | | | | | | | | | | | | | | | | | | | | | | | | | | | | | | | | | | | | | | | | | | | | | | | | | | | | | | | | | | | | | | | | | | | | | | | | | | | | | | | | | | | | | | | | | | | | | | | | | | | | | | | | | | | | | | | | | | | | | | | | | | | | | | | | | | | | | | | | | | | | | |
|  | | Alice | | Aminah | | Anto | | Asny | | Bagong | | Bendot | | Berani | | Beth | | Bibi | | Bintang | | Brutus | | Chindy | | Codet | | Elly | | Fajar | | Feb | | Female | | Fio | | Flanged male | | Freddy | | Friska | | Fugit | | Gangstah | | Gordon | | Gracia | | Gretel | | Henk | | Icarus | | Imp | | Indah | | Indi | | Irma | | James | | Janda Tua | | Jinak | | Joy | | Juni | | Juno | | Kacil | | Kan | | Kasi | | Kay | | Keri | | Keto | | Kondor | | Kundur | | Madalena | | Malé | | Mindi | | Ompung | | Pensi | | Peot | | Prabu | | Raffi | | Rambo | | Ronaldo | | Salvador | | Suci | | Sultan | | Sumi | | Teju | | Teresia | | Timi | | Tina | | Travor | | Umi | | Unflm | | Uok | | Vulcan | | Walimah | | Wulan | | XL | | Xenix | | Yanti | | Zeus | | Zorro | | Alice | | Aminah | | Anto | | Asny | | Bagong | | Bendot | | Berani | | Beth | | Bibi | | Bintang | | Brutus | | Chindy | | Codet | | Elly | | Fajar | | Feb | | Female | | Fio | | Flanged male | | Freddy | | Friska | | Fugit | | Gangstah | | Gordon | | Gracia | | Gretel | | Henk | | Icarus | | Imp | | Indah | | Indi | | Irma | | James | | Janda Tua | | Jinak | | Joy | | Juni | | Juno | | Kacil | | Kan | | Kasi | | Kay | | Keri | | Keto | | Kondor | | Kundur | | Madalena | | Malé | | Mindi | | Ompung | | Pensi | | Peot | | Prabu | | Raffi | | Rambo | | Ronaldo | | Salvador | | Suci | | Sultan | | Sumi | | Teju | | Teresia | | Timi | | Tina | | Travor | | Umi | | Unflm | | Uok | | Vulcan | | Walimah | | Wulan | | XL | | Xenix | | Yanti | | Zeus | | Zorro | | Alice | | Aminah | | Anto | | Asny | | Bagong | | Bendot | | Berani | | Beth | | Bibi | | Bintang | | Brutus | | Chindy | | Codet | | Elly | | Fajar | | Feb | | Female | | Fio | | Flanged male | | Freddy | | Friska | | Fugit | | Gangstah | | Gordon | | Gracia | | Gretel | | Henk | | Icarus | | Imp | | Indah | | Indi | | Irma | | James | | Janda Tua | | Jinak | | Joy | | Juni | | Juno | | Kacil | | Kan | | Kasi | | Kay | | Keri | | Keto | | Kondor | | Kundur | | Madalena | | Malé | | Mindi | | Ompung | | Pensi | | Peot | | Prabu | | Raffi | | Rambo | | Ronaldo | | Salvador | | Suci | | Sultan | | Sumi | | Teju | | Teresia | | Timi | | Tina | | Travor | | Umi | | Unflm | | Uok | | Vulcan | | Walimah | | Wulan | | XL | | Xenix | | Yanti | | Zeus | | Zorro | | Alice | | Aminah | | Anto | | Asny | | Bagong | | Bendot | | Berani | | Beth | | Bibi | | Bintang | | Brutus | | Chindy | | Codet | | Elly | | Fajar | | Feb | | Female | | Fio | | Flanged male | | Freddy | | Friska | | Fugit | | Gangstah | | Gordon | | Gracia | | Gretel | | Henk | | Icarus | | Imp | | Indah | | Indi | | Irma | | James | | Janda Tua | | Jinak | | Joy | | Juni | | Juno | | Kacil | | Kan | | Kasi | | Kay | | Keri | | Keto | | Kondor | | Kundur | | Madalena | | Malé | | Mindi | | Ompung | | Pensi | | Peot | | Prabu | | Raffi | | Rambo | | Ronaldo | | Salvador | | Suci | | Sultan | | Sumi | | Teju | | Teresia | | Timi | | Tina | | Travor | | Umi | | Unflm | | Uok | | Vulcan | | Walimah | | Wulan | | XL | | Xenix | | Yanti | | Zeus | | Zorro | | Alice | | Aminah | | Anto | | Asny | | Bagong | | Bendot | | Berani | | Beth | | Bibi | | Bintang | | Brutus | | Chindy | | Codet | | Elly | | Fajar | | Feb | | Female | | Fio | | Flanged male | | Freddy | | Friska | | Fugit | | Gangstah | | Gordon | | Gracia | | Gretel | | Henk | | Icarus | | Imp | | Indah | | Indi | | Irma | | James | | Janda Tua | | Jinak | | Joy | | Juni | | Juno | | Kacil | | Kan | | Kasi | | Kay | | Keri | | Keto | | Kondor | | Kundur | | Madalena | | Malé | | Mindi | | Ompung | | Pensi | | Peot | | Prabu | | Raffi | | Rambo | | Ronaldo | | Salvador | | Suci | | Sultan | | Sumi | | Teju | | Teresia | | Timi | | Tina | | Travor | | Umi | | Unflm | | Uok | | Vulcan | | Walimah | | Wulan | | XL | | Xenix | | Yanti | | Zeus | | Zorro | |
| Valid |  | 20 |  | 26 |  | 5 |  | 15 |  | 101 |  | 65 |  | 51 |  | 46 |  | 13 |  | 93 |  | 183 |  | 22 |  | 32 |  | 51 |  | 246 |  | 4 |  | 19 |  | 9 |  | 6 |  | 1 |  | 1 |  | 126 |  | 17 |  | 52 |  | 54 |  | 36 |  | 4 |  | 5 |  | 49 |  | 17 |  | 20 |  | 1590 |  | 7 |  | 3 |  | 41 |  | 2 |  | 4 |  | 14 |  | 5 |  | 2 |  | 159 |  | 111 |  | 6 |  | 1 |  | 38 |  | 42 |  | 15 |  | 104 |  | 17 |  | 263 |  | 15 |  | 8 |  | 80 |  | 61 |  | 29 |  | 1 |  | 46 |  | 5 |  | 106 |  | 32 |  | 22 |  | 28 |  | 3 |  | 316 |  | 1 |  | 2 |  | 32 |  | 49 |  | 2 |  | 100 |  | 69 |  | 41 |  | 8 |  | 414 |  | 1 |  | 11 |  | 20 |  | 26 |  | 5 |  | 15 |  | 101 |  | 65 |  | 51 |  | 46 |  | 13 |  | 93 |  | 183 |  | 22 |  | 32 |  | 51 |  | 246 |  | 4 |  | 19 |  | 9 |  | 6 |  | 1 |  | 1 |  | 126 |  | 17 |  | 52 |  | 54 |  | 36 |  | 4 |  | 5 |  | 49 |  | 17 |  | 20 |  | 1590 |  | 7 |  | 3 |  | 41 |  | 2 |  | 4 |  | 14 |  | 5 |  | 2 |  | 159 |  | 111 |  | 6 |  | 1 |  | 38 |  | 42 |  | 15 |  | 104 |  | 17 |  | 263 |  | 15 |  | 8 |  | 80 |  | 61 |  | 29 |  | 1 |  | 46 |  | 5 |  | 106 |  | 32 |  | 22 |  | 28 |  | 3 |  | 316 |  | 1 |  | 2 |  | 32 |  | 49 |  | 2 |  | 100 |  | 69 |  | 41 |  | 8 |  | 414 |  | 1 |  | 11 |  | 20 |  | 26 |  | 5 |  | 15 |  | 101 |  | 65 |  | 51 |  | 46 |  | 13 |  | 93 |  | 183 |  | 22 |  | 32 |  | 51 |  | 246 |  | 4 |  | 19 |  | 9 |  | 6 |  | 1 |  | 1 |  | 126 |  | 17 |  | 52 |  | 54 |  | 36 |  | 4 |  | 5 |  | 49 |  | 17 |  | 20 |  | 1590 |  | 7 |  | 3 |  | 41 |  | 2 |  | 4 |  | 14 |  | 5 |  | 2 |  | 159 |  | 107 |  | 6 |  | 1 |  | 38 |  | 42 |  | 15 |  | 104 |  | 17 |  | 263 |  | 15 |  | 8 |  | 80 |  | 61 |  | 29 |  | 1 |  | 46 |  | 5 |  | 106 |  | 32 |  | 22 |  | 28 |  | 3 |  | 316 |  | 1 |  | 2 |  | 32 |  | 49 |  | 2 |  | 100 |  | 69 |  | 41 |  | 8 |  | 414 |  | 1 |  | 11 |  | 20 |  | 26 |  | 5 |  | 15 |  | 101 |  | 65 |  | 51 |  | 46 |  | 13 |  | 93 |  | 183 |  | 22 |  | 32 |  | 51 |  | 246 |  | 4 |  | 19 |  | 9 |  | 6 |  | 1 |  | 1 |  | 126 |  | 17 |  | 52 |  | 54 |  | 36 |  | 4 |  | 5 |  | 49 |  | 17 |  | 20 |  | 1590 |  | 7 |  | 3 |  | 41 |  | 2 |  | 4 |  | 14 |  | 5 |  | 2 |  | 159 |  | 111 |  | 6 |  | 1 |  | 38 |  | 42 |  | 15 |  | 104 |  | 17 |  | 263 |  | 15 |  | 8 |  | 80 |  | 61 |  | 29 |  | 1 |  | 46 |  | 5 |  | 106 |  | 32 |  | 22 |  | 28 |  | 3 |  | 316 |  | 1 |  | 2 |  | 32 |  | 49 |  | 2 |  | 100 |  | 69 |  | 41 |  | 8 |  | 414 |  | 1 |  | 11 |  | 20 |  | 26 |  | 5 |  | 15 |  | 101 |  | 65 |  | 51 |  | 46 |  | 13 |  | 92 |  | 183 |  | 22 |  | 32 |  | 51 |  | 246 |  | 4 |  | 19 |  | 9 |  | 6 |  | 1 |  | 1 |  | 126 |  | 17 |  | 52 |  | 54 |  | 36 |  | 4 |  | 5 |  | 49 |  | 17 |  | 20 |  | 1590 |  | 7 |  | 3 |  | 41 |  | 2 |  | 4 |  | 14 |  | 4 |  | 2 |  | 144 |  | 111 |  | 6 |  | 1 |  | 38 |  | 42 |  | 15 |  | 104 |  | 17 |  | 263 |  | 14 |  | 8 |  | 80 |  | 61 |  | 29 |  | 1 |  | 46 |  | 5 |  | 106 |  | 32 |  | 22 |  | 28 |  | 3 |  | 316 |  | 1 |  | 2 |  | 32 |  | 49 |  | 2 |  | 100 |  | 66 |  | 41 |  | 8 |  | 414 |  | 1 |  | 11 |  |
| Missing |  | 0 |  | 0 |  | 0 |  | 0 |  | 0 |  | 0 |  | 0 |  | 0 |  | 0 |  | 0 |  | 0 |  | 0 |  | 0 |  | 0 |  | 0 |  | 0 |  | 0 |  | 0 |  | 0 |  | 0 |  | 0 |  | 0 |  | 0 |  | 0 |  | 0 |  | 0 |  | 0 |  | 0 |  | 0 |  | 0 |  | 0 |  | 0 |  | 0 |  | 0 |  | 0 |  | 0 |  | 0 |  | 0 |  | 0 |  | 0 |  | 0 |  | 0 |  | 0 |  | 0 |  | 0 |  | 0 |  | 0 |  | 0 |  | 0 |  | 0 |  | 0 |  | 0 |  | 0 |  | 0 |  | 0 |  | 0 |  | 0 |  | 0 |  | 0 |  | 0 |  | 0 |  | 0 |  | 0 |  | 0 |  | 0 |  | 0 |  | 0 |  | 0 |  | 0 |  | 0 |  | 0 |  | 0 |  | 0 |  | 0 |  | 0 |  | 0 |  | 0 |  | 0 |  | 0 |  | 0 |  | 0 |  | 0 |  | 0 |  | 0 |  | 0 |  | 0 |  | 0 |  | 0 |  | 0 |  | 0 |  | 0 |  | 0 |  | 0 |  | 0 |  | 0 |  | 0 |  | 0 |  | 0 |  | 0 |  | 0 |  | 0 |  | 0 |  | 0 |  | 0 |  | 0 |  | 0 |  | 0 |  | 0 |  | 0 |  | 0 |  | 0 |  | 0 |  | 0 |  | 0 |  | 0 |  | 0 |  | 0 |  | 0 |  | 0 |  | 0 |  | 0 |  | 0 |  | 0 |  | 0 |  | 0 |  | 0 |  | 0 |  | 0 |  | 0 |  | 0 |  | 0 |  | 0 |  | 0 |  | 0 |  | 0 |  | 0 |  | 0 |  | 0 |  | 0 |  | 0 |  | 0 |  | 0 |  | 0 |  | 0 |  | 0 |  | 0 |  | 0 |  | 0 |  | 0 |  | 0 |  | 0 |  | 0 |  | 0 |  | 0 |  | 0 |  | 0 |  | 0 |  | 0 |  | 0 |  | 0 |  | 0 |  | 0 |  | 0 |  | 0 |  | 0 |  | 0 |  | 0 |  | 0 |  | 0 |  | 0 |  | 0 |  | 0 |  | 0 |  | 0 |  | 0 |  | 0 |  | 0 |  | 0 |  | 0 |  | 0 |  | 0 |  | 0 |  | 0 |  | 0 |  | 0 |  | 0 |  | 0 |  | 0 |  | 0 |  | 0 |  | 0 |  | 0 |  | 0 |  | 4 |  | 0 |  | 0 |  | 0 |  | 0 |  | 0 |  | 0 |  | 0 |  | 0 |  | 0 |  | 0 |  | 0 |  | 0 |  | 0 |  | 0 |  | 0 |  | 0 |  | 0 |  | 0 |  | 0 |  | 0 |  | 0 |  | 0 |  | 0 |  | 0 |  | 0 |  | 0 |  | 0 |  | 0 |  | 0 |  | 0 |  | 0 |  | 0 |  | 0 |  | 0 |  | 0 |  | 0 |  | 0 |  | 0 |  | 0 |  | 0 |  | 0 |  | 0 |  | 0 |  | 0 |  | 0 |  | 0 |  | 0 |  | 0 |  | 0 |  | 0 |  | 0 |  | 0 |  | 0 |  | 0 |  | 0 |  | 0 |  | 0 |  | 0 |  | 0 |  | 0 |  | 0 |  | 0 |  | 0 |  | 0 |  | 0 |  | 0 |  | 0 |  | 0 |  | 0 |  | 0 |  | 0 |  | 0 |  | 0 |  | 0 |  | 0 |  | 0 |  | 0 |  | 0 |  | 0 |  | 0 |  | 0 |  | 0 |  | 0 |  | 0 |  | 0 |  | 0 |  | 0 |  | 0 |  | 0 |  | 0 |  | 0 |  | 0 |  | 0 |  | 0 |  | 0 |  | 0 |  | 0 |  | 0 |  | 0 |  | 0 |  | 0 |  | 0 |  | 0 |  | 0 |  | 0 |  | 0 |  | 0 |  | 0 |  | 0 |  | 0 |  | 0 |  | 0 |  | 0 |  | 0 |  | 0 |  | 0 |  | 0 |  | 0 |  | 0 |  | 1 |  | 0 |  | 0 |  | 0 |  | 0 |  | 0 |  | 0 |  | 0 |  | 0 |  | 0 |  | 0 |  | 0 |  | 0 |  | 0 |  | 0 |  | 0 |  | 0 |  | 0 |  | 0 |  | 0 |  | 0 |  | 0 |  | 0 |  | 0 |  | 0 |  | 0 |  | 0 |  | 0 |  | 0 |  | 1 |  | 0 |  | 15 |  | 0 |  | 0 |  | 0 |  | 0 |  | 0 |  | 0 |  | 0 |  | 0 |  | 0 |  | 1 |  | 0 |  | 0 |  | 0 |  | 0 |  | 0 |  | 0 |  | 0 |  | 0 |  | 0 |  | 0 |  | 0 |  | 0 |  | 0 |  | 0 |  | 0 |  | 0 |  | 0 |  | 0 |  | 0 |  | 3 |  | 0 |  | 0 |  | 0 |  | 0 |  | 0 |  |
| Mean |  |  |  |  |  |  |  |  |  |  |  |  |  |  |  |  |  |  |  |  |  |  |  |  |  |  |  |  |  |  |  |  |  |  |  |  |  |  |  |  |  |  |  |  |  |  |  |  |  |  |  |  |  |  |  |  |  |  |  |  |  |  |  |  |  |  |  |  |  |  |  |  |  |  |  |  |  |  |  |  |  |  |  |  |  |  |  |  |  |  |  |  |  |  |  |  |  |  |  |  |  |  |  |  |  |  |  |  |  |  |  |  |  |  |  |  |  |  |  |  |  |  |  |  |  |  |  |  |  |  |  |  |  |  |  |  |  |  |  |  |  |  |  |  |  |  |  |  |  |  |  |  |  |  |  |  |  |  |  |  |  |  |  |  |  |  |  |  |  |  |  |  |  |  |  |  |  |  |  |  |  |  |  |  |  |  |  |  |  |  |  |  |  |  |  |  |  |  |  |  |  |  |  |  |  |  |  |  |  |  |  |  |  |  |  |  |  |  |  |  |  |  |  |  |  |  |  |  |  |  |  |  |  |  |  |  |  |  |  |  |  |  |  |  |  |  |  |  |  |  |  |  |  |  |  |  |  |  |  |  |  |  |  |  |  |  |  |  |  |  |  |  |  |  |  |  |  |  |  |  |  |  |  |  |  |  |  |  |  |  |  |  |  |  |  |  |  |  |  |  |  |  |  |  |  |  |  |  |  |  |  |  |  |  |  |  |  |  |  |  |  |  |  |  |  |  |  |  |  |  |  |  |  |  |  |  |  |  |  |  |  |  |  |  |  |  |  |  |  |  |  |  |  |  |  |  |  |  |  |  |  |  |  |  |  |  |  |  |  |  |  |  |  |  |  |  |  |  |  |  |  |  |  |  |  |  |  |  |  |  |  |  |  |  |  |  |  |  |  |  |  |  |  |  |  |  |  |  |  |  |  |  |  |  |  |  |  |  |  |  |  |  |  |  |  |  |  |  |  |  |  |  |  |  |  |  |  |  |  |  |  |  |  |  |  |  |  |  |  |  |  |  |  |  |  |  |  | 143.845 |  | 5346.869 |  | 4851.960 |  | 4163.087 |  | 3011.140 |  | 4413.146 |  | 6323.167 |  | 3342.315 |  | 3630.823 |  | 3202.922 |  | 4670.148 |  | 136.382 |  | 3892.119 |  | 141.431 |  | 3144.889 |  | 3569.150 |  | 3783.768 |  | 4210.056 |  | 2609.583 |  | 115.900 |  | 136.900 |  | 3727.509 |  | 140.882 |  | 3461.869 |  | 3667.122 |  | 2660.397 |  | 3404.275 |  | 5094.720 |  | 152.465 |  | 6541.029 |  | 4048.235 |  | 4720.685 |  | 4002.886 |  | 3703.700 |  | 4375.312 |  | 3457.450 |  | 3526.075 |  | 3807.386 |  | 4743.740 |  | 4078.100 |  | 4561.911 |  | 3065.496 |  | 4474.417 |  | 4468.100 |  | 5826.361 |  | 5197.379 |  | 4510.673 |  | 4091.646 |  | 3628.035 |  | 3154.214 |  | 3799.993 |  | 4048.225 |  | 3443.157 |  | 134.784 |  | 2694.255 |  | 125.900 |  | 3606.765 |  | 4549.440 |  | 2964.654 |  | 4035.278 |  | 2972.436 |  | 3202.007 |  | 4468.767 |  | 137.742 |  | 5598.600 |  | 5124.900 |  | 2813.806 |  | 5473.829 |  | 2729.300 |  | 4077.524 |  | 4361.413 |  | 3485.217 |  | 139.975 |  | 4390.216 |  | 3119.600 |  | 3656.736 |  | 0.310 |  | 0.485 |  | 0.324 |  | 0.876 |  | 0.620 |  | 0.814 |  | 0.592 |  | 0.814 |  | 0.636 |  | 0.543 |  | 0.349 |  | 0.430 |  | 0.564 |  | 0.389 |  | 0.473 |  | 0.404 |  | 0.269 |  | 0.998 |  | 0.248 |  | 0.155 |  | 0.250 |  | 0.256 |  | 0.760 |  | 0.989 |  | 0.795 |  | 0.316 |  | 0.569 |  | 0.124 |  | 0.308 |  | 0.377 |  | 0.653 |  | 0.396 |  | 0.388 |  | 0.488 |  | 0.340 |  | 0.210 |  | 0.771 |  | 0.214 |  | 0.361 |  | 0.296 |  | 0.630 |  | 0.356 |  | 0.416 |  | 0.220 |  | 0.224 |  | 0.211 |  | 0.403 |  | 0.717 |  | 0.368 |  | 0.519 |  | 0.540 |  | 0.520 |  | 0.596 |  | 0.266 |  | 0.388 |  | 0.290 |  | 0.771 |  | 0.398 |  | 0.382 |  | 0.477 |  | 0.366 |  | 0.338 |  | 0.223 |  | 0.388 |  | 0.406 |  | 0.303 |  | 0.314 |  | 0.604 |  | 0.262 |  | 0.487 |  | 0.537 |  | 1.470 |  | 0.594 |  | 0.654 |  | 0.201 |  | 0.718 |  |
| Std. Deviation |  |  |  |  |  |  |  |  |  |  |  |  |  |  |  |  |  |  |  |  |  |  |  |  |  |  |  |  |  |  |  |  |  |  |  |  |  |  |  |  |  |  |  |  |  |  |  |  |  |  |  |  |  |  |  |  |  |  |  |  |  |  |  |  |  |  |  |  |  |  |  |  |  |  |  |  |  |  |  |  |  |  |  |  |  |  |  |  |  |  |  |  |  |  |  |  |  |  |  |  |  |  |  |  |  |  |  |  |  |  |  |  |  |  |  |  |  |  |  |  |  |  |  |  |  |  |  |  |  |  |  |  |  |  |  |  |  |  |  |  |  |  |  |  |  |  |  |  |  |  |  |  |  |  |  |  |  |  |  |  |  |  |  |  |  |  |  |  |  |  |  |  |  |  |  |  |  |  |  |  |  |  |  |  |  |  |  |  |  |  |  |  |  |  |  |  |  |  |  |  |  |  |  |  |  |  |  |  |  |  |  |  |  |  |  |  |  |  |  |  |  |  |  |  |  |  |  |  |  |  |  |  |  |  |  |  |  |  |  |  |  |  |  |  |  |  |  |  |  |  |  |  |  |  |  |  |  |  |  |  |  |  |  |  |  |  |  |  |  |  |  |  |  |  |  |  |  |  |  |  |  |  |  |  |  |  |  |  |  |  |  |  |  |  |  |  |  |  |  |  |  |  |  |  |  |  |  |  |  |  |  |  |  |  |  |  |  |  |  |  |  |  |  |  |  |  |  |  |  |  |  |  |  |  |  |  |  |  |  |  |  |  |  |  |  |  |  |  |  |  |  |  |  |  |  |  |  |  |  |  |  |  |  |  |  |  |  |  |  |  |  |  |  |  |  |  |  |  |  |  |  |  |  |  |  |  |  |  |  |  |  |  |  |  |  |  |  |  |  |  |  |  |  |  |  |  |  |  |  |  |  |  |  |  |  |  |  |  |  |  |  |  |  |  |  |  |  |  |  |  |  |  |  |  |  |  |  |  |  |  |  |  |  |  |  |  |  |  |  |  |  |  |  |  |  |  |  | 5.196 |  | 777.252 |  | 151.721 |  | 1218.397 |  | 270.834 |  | 647.145 |  | 2325.913 |  | 394.756 |  | 213.444 |  | 239.650 |  | 897.790 |  | 4.965 |  | 972.636 |  | 6.737 |  | 860.651 |  | 225.172 |  | 384.627 |  | 566.429 |  | 1268.495 |  | NaN |  | NaN |  | 671.965 |  | 5.853 |  | 870.570 |  | 1221.800 |  | 455.045 |  | 279.601 |  | 2543.374 |  | 5.181 |  | 2666.194 |  | 655.977 |  | 436.570 |  | 511.679 |  | 172.300 |  | 503.140 |  | 138.946 |  | 110.896 |  | 325.527 |  | 557.008 |  | 2717.977 |  | 447.478 |  | 1192.320 |  | 639.687 |  | NaN |  | 1190.038 |  | 715.728 |  | 429.811 |  | 455.915 |  | 518.817 |  | 633.611 |  | 1262.188 |  | 159.488 |  | 1336.682 |  | 5.888 |  | 970.593 |  | NaN |  | 871.519 |  | 816.168 |  | 992.512 |  | 1178.053 |  | 1052.044 |  | 1157.797 |  | 832.306 |  | 5.297 |  | NaN |  | 304.480 |  | 829.934 |  | 1872.356 |  | 780.363 |  | 963.905 |  | 780.674 |  | 533.084 |  | 3.460 |  | 709.312 |  | NaN |  | 905.322 |  | 0.098 |  | 0.146 |  | 0.108 |  | 0.400 |  | 0.164 |  | 0.177 |  | 0.229 |  | 0.332 |  | 0.113 |  | 0.124 |  | 0.138 |  | 0.286 |  | 0.188 |  | 0.136 |  | 0.109 |  | 0.215 |  | 0.128 |  | 0.791 |  | 0.075 |  | NaN |  | NaN |  | 0.088 |  | 0.313 |  | 0.424 |  | 0.355 |  | 0.348 |  | 0.223 |  | 0.050 |  | 0.119 |  | 0.141 |  | 0.241 |  | 0.096 |  | 0.192 |  | 0.227 |  | 0.046 |  | 0.022 |  | 0.122 |  | 0.053 |  | 0.102 |  | 0.064 |  | 0.168 |  | 0.150 |  | 0.149 |  | NaN |  | 0.092 |  | 0.127 |  | 0.117 |  | 0.174 |  | 0.069 |  | 0.155 |  | 0.115 |  | 0.249 |  | 0.155 |  | 0.084 |  | 0.140 |  | NaN |  | 0.377 |  | 0.108 |  | 0.667 |  | 0.155 |  | 0.144 |  | 0.186 |  | 0.053 |  | 0.177 |  | NaN |  | 0.096 |  | 0.189 |  | 0.270 |  | 0.249 |  | 0.186 |  | 0.219 |  | 0.979 |  | 0.160 |  | 0.194 |  | NaN |  | 0.164 |  |
| Minimum |  |  |  |  |  |  |  |  |  |  |  |  |  |  |  |  |  |  |  |  |  |  |  |  |  |  |  |  |  |  |  |  |  |  |  |  |  |  |  |  |  |  |  |  |  |  |  |  |  |  |  |  |  |  |  |  |  |  |  |  |  |  |  |  |  |  |  |  |  |  |  |  |  |  |  |  |  |  |  |  |  |  |  |  |  |  |  |  |  |  |  |  |  |  |  |  |  |  |  |  |  |  |  |  |  |  |  |  |  |  |  |  |  |  |  |  |  |  |  |  |  |  |  |  |  |  |  |  |  |  |  |  |  |  |  |  |  |  |  |  |  |  |  |  |  |  |  |  |  |  |  |  |  |  |  |  |  |  |  |  |  |  |  |  |  |  |  |  |  |  |  |  |  |  |  |  |  |  |  |  |  |  |  |  |  |  |  |  |  |  |  |  |  |  |  |  |  |  |  |  |  |  |  |  |  |  |  |  |  |  |  |  |  |  |  |  |  |  |  |  |  |  |  |  |  |  |  |  |  |  |  |  |  |  |  |  |  |  |  |  |  |  |  |  |  |  |  |  |  |  |  |  |  |  |  |  |  |  |  |  |  |  |  |  |  |  |  |  |  |  |  |  |  |  |  |  |  |  |  |  |  |  |  |  |  |  |  |  |  |  |  |  |  |  |  |  |  |  |  |  |  |  |  |  |  |  |  |  |  |  |  |  |  |  |  |  |  |  |  |  |  |  |  |  |  |  |  |  |  |  |  |  |  |  |  |  |  |  |  |  |  |  |  |  |  |  |  |  |  |  |  |  |  |  |  |  |  |  |  |  |  |  |  |  |  |  |  |  |  |  |  |  |  |  |  |  |  |  |  |  |  |  |  |  |  |  |  |  |  |  |  |  |  |  |  |  |  |  |  |  |  |  |  |  |  |  |  |  |  |  |  |  |  |  |  |  |  |  |  |  |  |  |  |  |  |  |  |  |  |  |  |  |  |  |  |  |  |  |  |  |  |  |  |  |  |  |  |  |  |  |  |  |  |  |  |  |  | 133.800 |  | 4651.200 |  | 4761.500 |  | 775.200 |  | 2156.200 |  | 2831.600 |  | 430.700 |  | 2067.200 |  | 3186.900 |  | 2497.900 |  | 1717.300 |  | 127.100 |  | 2584.000 |  | 125.000 |  | 1205.900 |  | 3265.000 |  | 3222.700 |  | 3461.500 |  | 78.100 |  | 115.900 |  | 136.900 |  | 2702.400 |  | 132.100 |  | 172.300 |  | 1181.600 |  | 1959.500 |  | 2987.700 |  | 2298.700 |  | 141.300 |  | 3100.800 |  | 3014.600 |  | 1711.900 |  | 3558.400 |  | 3531.400 |  | 3563.700 |  | 3359.200 |  | 3450.700 |  | 3348.400 |  | 4218.800 |  | 2156.200 |  | 2250.000 |  | 223.400 |  | 3526.100 |  | 4468.100 |  | 3098.100 |  | 3458.800 |  | 3824.800 |  | 3187.500 |  | 2925.800 |  | 1927.200 |  | 468.800 |  | 3789.800 |  | 516.800 |  | 119.000 |  | 266.500 |  | 125.900 |  | 1251.600 |  | 3725.200 |  | 0.300 |  | 2985.000 |  | 228.800 |  | 1200.500 |  | 3914.100 |  | 115.400 |  | 5598.600 |  | 4909.600 |  | 1324.300 |  | 172.300 |  | 2177.500 |  | 258.400 |  | 2343.800 |  | 2239.500 |  | 134.300 |  | 2715.900 |  | 3119.600 |  | 2497.900 |  | 0.132 |  | 0.212 |  | 0.207 |  | 0.335 |  | 0.308 |  | 0.302 |  | 0.129 |  | 0.289 |  | 0.528 |  | 0.235 |  | 0.078 |  | 0.098 |  | 0.262 |  | 0.139 |  | 0.198 |  | 0.176 |  | 0.070 |  | 0.322 |  | 0.126 |  | 0.155 |  | 0.250 |  | 0.112 |  | 0.272 |  | 0.293 |  | 0.101 |  | 0.081 |  | 0.297 |  | 0.038 |  | 0.105 |  | 0.140 |  | 0.272 |  | 0.148 |  | 0.213 |  | 0.349 |  | 0.180 |  | 0.195 |  | 0.666 |  | 0.119 |  | 0.211 |  | 0.251 |  | 0.256 |  | 0.157 |  | 0.236 |  | 0.220 |  | 0.084 |  | 0.044 |  | 0.241 |  | 0.248 |  | 0.265 |  | 0.182 |  | 0.330 |  | 0.298 |  | 0.187 |  | 0.112 |  | 0.160 |  | 0.290 |  | 0.185 |  | 0.277 |  | 0.181 |  | 0.167 |  | 0.146 |  | 0.062 |  | 0.172 |  | 0.091 |  | 0.406 |  | 0.235 |  | 0.078 |  | 0.274 |  | 0.086 |  | 0.219 |  | 0.231 |  | 0.211 |  | 0.385 |  | 0.182 |  | 0.201 |  | 0.433 |  |
| Maximum |  |  |  |  |  |  |  |  |  |  |  |  |  |  |  |  |  |  |  |  |  |  |  |  |  |  |  |  |  |  |  |  |  |  |  |  |  |  |  |  |  |  |  |  |  |  |  |  |  |  |  |  |  |  |  |  |  |  |  |  |  |  |  |  |  |  |  |  |  |  |  |  |  |  |  |  |  |  |  |  |  |  |  |  |  |  |  |  |  |  |  |  |  |  |  |  |  |  |  |  |  |  |  |  |  |  |  |  |  |  |  |  |  |  |  |  |  |  |  |  |  |  |  |  |  |  |  |  |  |  |  |  |  |  |  |  |  |  |  |  |  |  |  |  |  |  |  |  |  |  |  |  |  |  |  |  |  |  |  |  |  |  |  |  |  |  |  |  |  |  |  |  |  |  |  |  |  |  |  |  |  |  |  |  |  |  |  |  |  |  |  |  |  |  |  |  |  |  |  |  |  |  |  |  |  |  |  |  |  |  |  |  |  |  |  |  |  |  |  |  |  |  |  |  |  |  |  |  |  |  |  |  |  |  |  |  |  |  |  |  |  |  |  |  |  |  |  |  |  |  |  |  |  |  |  |  |  |  |  |  |  |  |  |  |  |  |  |  |  |  |  |  |  |  |  |  |  |  |  |  |  |  |  |  |  |  |  |  |  |  |  |  |  |  |  |  |  |  |  |  |  |  |  |  |  |  |  |  |  |  |  |  |  |  |  |  |  |  |  |  |  |  |  |  |  |  |  |  |  |  |  |  |  |  |  |  |  |  |  |  |  |  |  |  |  |  |  |  |  |  |  |  |  |  |  |  |  |  |  |  |  |  |  |  |  |  |  |  |  |  |  |  |  |  |  |  |  |  |  |  |  |  |  |  |  |  |  |  |  |  |  |  |  |  |  |  |  |  |  |  |  |  |  |  |  |  |  |  |  |  |  |  |  |  |  |  |  |  |  |  |  |  |  |  |  |  |  |  |  |  |  |  |  |  |  |  |  |  |  |  |  |  |  |  |  |  |  |  |  |  |  |  |  |  |  |  |  | 153.100 |  | 7149.000 |  | 5122.200 |  | 5770.900 |  | 4031.200 |  | 5577.100 |  | 11800.200 |  | 4048.200 |  | 4048.200 |  | 3843.800 |  | 8516.400 |  | 144.500 |  | 6201.600 |  | 156.300 |  | 11369.500 |  | 3808.700 |  | 4898.400 |  | 4963.400 |  | 3367.300 |  | 115.900 |  | 136.900 |  | 5622.900 |  | 153.100 |  | 5168.000 |  | 6182.700 |  | 4834.200 |  | 3588.000 |  | 7116.700 |  | 164.400 |  | 11800.200 |  | 4909.600 |  | 7630.800 |  | 5063.000 |  | 3876.000 |  | 5539.400 |  | 3555.700 |  | 3690.300 |  | 4404.200 |  | 5437.500 |  | 6000.000 |  | 5625.000 |  | 5698.200 |  | 5272.900 |  | 4468.100 |  | 10174.400 |  | 7289.000 |  | 5297.200 |  | 5343.800 |  | 4804.600 |  | 4982.200 |  | 4781.200 |  | 4306.600 |  | 6115.400 |  | 146.800 |  | 5162.600 |  | 125.900 |  | 6142.300 |  | 5846.300 |  | 5905.500 |  | 9528.400 |  | 5875.900 |  | 7345.500 |  | 5425.800 |  | 154.100 |  | 5598.600 |  | 5340.200 |  | 5485.600 |  | 11111.100 |  | 3281.100 |  | 6632.200 |  | 9093.800 |  | 4909.600 |  | 145.300 |  | 7259.400 |  | 3119.600 |  | 4909.600 |  | 0.506 |  | 0.841 |  | 0.432 |  | 1.792 |  | 1.031 |  | 1.333 |  | 1.123 |  | 1.771 |  | 0.918 |  | 1.044 |  | 1.001 |  | 1.223 |  | 1.057 |  | 0.734 |  | 0.826 |  | 0.615 |  | 0.555 |  | 2.962 |  | 0.332 |  | 0.155 |  | 0.250 |  | 0.666 |  | 1.311 |  | 2.273 |  | 1.541 |  | 1.748 |  | 0.820 |  | 0.167 |  | 0.598 |  | 0.714 |  | 1.194 |  | 0.791 |  | 0.803 |  | 0.750 |  | 0.418 |  | 0.226 |  | 0.919 |  | 0.329 |  | 0.428 |  | 0.341 |  | 1.165 |  | 0.766 |  | 0.593 |  | 0.220 |  | 0.491 |  | 0.732 |  | 0.674 |  | 1.264 |  | 0.539 |  | 1.002 |  | 0.687 |  | 1.059 |  | 0.978 |  | 0.517 |  | 0.688 |  | 0.290 |  | 1.881 |  | 0.550 |  | 7.136 |  | 0.922 |  | 0.642 |  | 0.889 |  | 0.277 |  | 1.192 |  | 0.406 |  | 0.371 |  | 0.951 |  | 1.379 |  | 0.438 |  | 1.173 |  | 1.485 |  | 4.985 |  | 0.856 |  | 1.503 |  | 0.201 |  | 0.987 |  |
|  | | | | | | | | | | | | | | | | | | | | | | | | | | | | | | | | | | | | | | | | | | | | | | | | | | | | | | | | | | | | | | | | | | | | | | | | | | | | | | | | | | | | | | | | | | | | | | | | | | | | | | | | | | | | | | | | | | | | | | | | | | | | | | | | | | | | | | | | | | | | | | | | | | | | | | | | | | | | | | | | | | | | | | | | | | | | | | | | | | | | | | | | | | | | | | | | | | | | | | | | | | | | | | | | | | | | | | | | | | | | | | | | | | | | | | | | | | | | | | | | | | | | | | | | | | | | | | | | | | | | | | | | | | | | | | | | | | | | | | | | | | | | | | | | | | | | | | | | | | | | | | | | | | | | | | | | | | | | | | | | | | | | | | | | | | | | | | | | | | | | | | | | | | | | | | | | | | | | | | | | | | | | | | | | | | | | | | | | | | | | | | | | | | | | | | | | | | | | | | | | | | | | | | | | | | | | | | | | | | | | | | | | | | | | | | | | | | | | | | | | | | | | | | | | | | | | | | | | | | | | | | | | | | | | | | | | | | | | | | | | | | | | | | | | | | | | | | | | | | | | | | | | | | | | | | | | | | | | | | | | | | | | | | | | | | | | | | | | | | | | | | | | | | | | | | | | | | | | | | | | | | | | | | | | | | | | | | | | | | | | | | | | | | | | | | | | | | | | | | | | | | | | | | | | | | | | | | | | | | | | | | | | | | | | | | | | | | | | | | | | | | | | | | | | | | | | | | | | | | | | | | | | | | | | | | | | | | | | | | | | | | | | | | | | | | | | | | | | | | | | | | | | | | | | | | | | | | | | | | | | | | | | | | | | | | | | | | | | | | | | | | | | | | | | | | | | | | | | | | | | | |
|  |  |  |  |  |  |  |  |  |  |  |  |  |  |  |  |  |  |  |  |  |  |  |  |  |  |  |  |  |  |  |  |  |  |  |  |  |  |  |  |  |  |  |  |  |  |  |  |  |  |  |  |  |  |  |  |  |  |  |  |  |  |  |  |  |  |  |  |  |  |  |  |  |  |  |  |  |  |  |  |  |  |  |  |  |  |  |  |  |  |  |  |  |  |  |  |  |  |  |  |  |  |  |  |  |  |  |  |  |  |  |  |  |  |  |  |  |  |  |  |  |  |  |  |  |  |  |  |  |  |  |  |  |  |  |  |  |  |  |  |  |  |  |  |  |  |  |  |  |  |  |  |  |  |  |  |  |  |  |  |  |  |  |  |  |  |  |  |  |  |  |  |  |  |  |  |  |  |  |  |  |  |  |  |  |  |  |  |  |  |  |  |  |  |  |  |  |  |  |  |  |  |  |  |  |  |  |  |  |  |  |  |  |  |  |  |  |  |  |  |  |  |  |  |  |  |  |  |  |  |  |  |  |  |  |  |  |  |  |  |  |  |  |  |  |  |  |  |  |  |  |  |  |  |  |  |  |  |  |  |  |  |  |  |  |  |  |  |  |  |  |  |  |  |  |  |  |  |  |  |  |  |  |  |  |  |  |  |  |  |  |  |  |  |  |  |  |  |  |  |  |  |  |  |  |  |  |  |  |  |  |  |  |  |  |  |  |  |  |  |  |  |  |  |  |  |  |  |  |  |  |  |  |  |  |  |  |  |  |  |  |  |  |  |  |  |  |  |  |  |  |  |  |  |  |  |  |  |  |  |  |  |  |  |  |  |  |  |  |  |  |  |  |  |  |  |  |  |  |  |  |  |  |  |  |  |  |  |  |  |  |  |  |  |  |  |  |  |  |  |  |  |  |  |  |  |  |  |  |  |  |  |  |  |  |  |  |  |  |  |  |  |  |  |  |  |  |  |  |  |  |  |  |  |  |  |  |  |  |  |  |  |  |  |  |  |  |  |  |  |  |  |  |  |  |  |  |  |  |  |  |  |  |  |  |  |  |  |  |  |  |  |  |  |  |  |  |  |  |  |  |  |  |  |  |  |  |  |  |  |  |  |  |  |  |  |  |  |  |  |  |  |  |  |  |  |  |  |  |  |  |  |  |  |  |  |  |  |  |  |  |  |  |  |  |  |  |  |  |  |  |  |  |  |  |  |  |  |  |  |  |  |  |  |  |  |  |  |  |  |  |  |  |  |  |  |  |  |  |  |  |  |  |  |  |  |  |  |  |  |  |  |  |  |  |  |  |  |  |  |  |  |  |  |  |  |  |  |  |  |  |  |  |  |  |  |  |  |  |  |  |  |  |  |  |  |  |  |  |  |  |  |  |  |  |  |  |  |  |  |  |  |  |  |  |  |  |  |  |  |  |  |  |  |  |  |  |  |  |  |  |  |  |  |  |  |  |  |  |  |  |  |  |  |  |  |  |  |  |  |  |  |  |  |  |  |  |  |  |  |  |  |  |  |  |  |  |  |  |  |  |  |  |  |  |  |  |  |  |  |  |  |  |  |  |  |  |  |  |  |  |  |  |  |  |  |  |  |  |  |  |  |  |  |  |  |  |  |  |  |  |  |  |  |  |  |  |  |  |  |  |  |  |  |  |  |  |  |  |  |  |  |  |  |  |  |  |  |  |  |  |  |  |  |  |  |  |  |  |  |  |  |
| --- | --- | --- | --- | --- | --- | --- | --- | --- | --- | --- | --- | --- | --- | --- | --- | --- | --- | --- | --- | --- | --- | --- | --- | --- | --- | --- | --- | --- | --- | --- | --- | --- | --- | --- | --- | --- | --- | --- | --- | --- | --- | --- | --- | --- | --- | --- | --- | --- | --- | --- | --- | --- | --- | --- | --- | --- | --- | --- | --- | --- | --- | --- | --- | --- | --- | --- | --- | --- | --- | --- | --- | --- | --- | --- | --- | --- | --- | --- | --- | --- | --- | --- | --- | --- | --- | --- | --- | --- | --- | --- | --- | --- | --- | --- | --- | --- | --- | --- | --- | --- | --- | --- | --- | --- | --- | --- | --- | --- | --- | --- | --- | --- | --- | --- | --- | --- | --- | --- | --- | --- | --- | --- | --- | --- | --- | --- | --- | --- | --- | --- | --- | --- | --- | --- | --- | --- | --- | --- | --- | --- | --- | --- | --- | --- | --- | --- | --- | --- | --- | --- | --- | --- | --- | --- | --- | --- | --- | --- | --- | --- | --- | --- | --- | --- | --- | --- | --- | --- | --- | --- | --- | --- | --- | --- | --- | --- | --- | --- | --- | --- | --- | --- | --- | --- | --- | --- | --- | --- | --- | --- | --- | --- | --- | --- | --- | --- | --- | --- | --- | --- | --- | --- | --- | --- | --- | --- | --- | --- | --- | --- | --- | --- | --- | --- | --- | --- | --- | --- | --- | --- | --- | --- | --- | --- | --- | --- | --- | --- | --- | --- | --- | --- | --- | --- | --- | --- | --- | --- | --- | --- | --- | --- | --- | --- | --- | --- | --- | --- | --- | --- | --- | --- | --- | --- | --- | --- | --- | --- | --- | --- | --- | --- | --- | --- | --- | --- | --- | --- | --- | --- | --- | --- | --- | --- | --- | --- | --- | --- | --- | --- | --- | --- | --- | --- | --- | --- | --- | --- | --- | --- | --- | --- | --- | --- | --- | --- | --- | --- | --- | --- | --- | --- | --- | --- | --- | --- | --- | --- | --- | --- | --- | --- | --- | --- | --- | --- | --- | --- | --- | --- | --- | --- | --- | --- | --- | --- | --- | --- | --- | --- | --- | --- | --- | --- | --- | --- | --- | --- | --- | --- | --- | --- | --- | --- | --- | --- | --- | --- | --- | --- | --- | --- | --- | --- | --- | --- | --- | --- | --- | --- | --- | --- | --- | --- | --- | --- | --- | --- | --- | --- | --- | --- | --- | --- | --- | --- | --- | --- | --- | --- | --- | --- | --- | --- | --- | --- | --- | --- | --- | --- | --- | --- | --- | --- | --- | --- | --- | --- | --- | --- | --- | --- | --- | --- | --- | --- | --- | --- | --- | --- | --- | --- | --- | --- | --- | --- | --- | --- | --- | --- | --- | --- | --- | --- | --- | --- | --- | --- | --- | --- | --- | --- | --- | --- | --- | --- | --- | --- | --- | --- | --- | --- | --- | --- | --- | --- | --- | --- | --- | --- | --- | --- | --- | --- | --- | --- | --- | --- | --- | --- | --- | --- | --- | --- | --- | --- | --- | --- | --- | --- | --- | --- | --- | --- | --- | --- | --- | --- | --- | --- | --- | --- | --- | --- | --- | --- | --- | --- | --- | --- | --- | --- | --- | --- | --- | --- | --- | --- | --- | --- | --- | --- | --- | --- | --- | --- | --- | --- | --- | --- | --- | --- | --- | --- | --- | --- | --- | --- | --- | --- | --- | --- | --- | --- | --- | --- | --- | --- | --- | --- | --- | --- | --- | --- | --- | --- | --- | --- | --- | --- | --- | --- | --- | --- | --- | --- | --- | --- | --- | --- | --- | --- | --- | --- | --- | --- | --- | --- | --- | --- | --- | --- | --- | --- | --- | --- | --- | --- | --- | --- | --- | --- | --- | --- | --- | --- | --- | --- | --- | --- | --- | --- | --- | --- | --- | --- | --- | --- | --- | --- | --- | --- | --- | --- | --- | --- | --- | --- | --- | --- | --- | --- | --- | --- | --- | --- | --- | --- | --- | --- | --- | --- | --- | --- | --- | --- | --- | --- | --- | --- | --- | --- | --- | --- | --- | --- | --- | --- | --- | --- | --- | --- | --- | --- | --- | --- | --- | --- | --- | --- | --- | --- | --- | --- | --- | --- | --- | --- | --- | --- | --- | --- | --- | --- | --- | --- | --- | --- | --- | --- | --- | --- | --- | --- | --- | --- | --- | --- | --- | --- | --- | --- | --- | --- | --- | --- | --- | --- | --- | --- | --- | --- | --- | --- | --- | --- | --- | --- | --- | --- | --- | --- | --- | --- | --- | --- | --- | --- | --- | --- | --- | --- | --- | --- | --- | --- | --- | --- | --- | --- | --- | --- | --- | --- | --- | --- | --- | --- | --- | --- | --- | --- | --- | --- | --- | --- | --- | --- | --- | --- | --- | --- | --- | --- | --- | --- | --- | --- | --- | --- | --- | --- | --- | --- | --- | --- | --- | --- | --- | --- | --- | --- | --- | --- | --- | --- | --- | --- | --- | --- | --- |
| *Note.*  Not all values are available for *Nominal Text* variables | | | | | | | | | | | | | | | | | | | | | | | | | | | | | | | | | | | | | | | | | | | | | | | | | | | | | | | | | | | | | | | | | | | | | | | | | | | | | | | | | | | | | | | | | | | | | | | | | | | | | | | | | | | | | | | | | | | | | | | | | | | | | | | | | | | | | | | | | | | | | | | | | | | | | | | | | | | | | | | | | | | | | | | | | | | | | | | | | | | | | | | | | | | | | | | | | | | | | | | | | | | | | | | | | | | | | | | | | | | | | | | | | | | | | | | | | | | | | | | | | | | | | | | | | | | | | | | | | | | | | | | | | | | | | | | | | | | | | | | | | | | | | | | | | | | | | | | | | | | | | | | | | | | | | | | | | | | | | | | | | | | | | | | | | | | | | | | | | | | | | | | | | | | | | | | | | | | | | | | | | | | | | | | | | | | | | | | | | | | | | | | | | | | | | | | | | | | | | | | | | | | | | | | | | | | | | | | | | | | | | | | | | | | | | | | | | | | | | | | | | | | | | | | | | | | | | | | | | | | | | | | | | | | | | | | | | | | | | | | | | | | | | | | | | | | | | | | | | | | | | | | | | | | | | | | | | | | | | | | | | | | | | | | | | | | | | | | | | | | | | | | | | | | | | | | | | | | | | | | | | | | | | | | | | | | | | | | | | | | | | | | | | | | | | | | | | | | | | | | | | | | | | | | | | | | | | | | | | | | | | | | | | | | | | | | | | | | | | | | | | | | | | | | | | | | | | | | | | | | | | | | | | | | | | | | | | | | | | | | | | | | | | | | | | | | | | | | | | | | | | | | | | | | | | | | | | | | | | | | | | | | | | | | | | | | | | | | | | | | | | | | | | | | | | | | | | | | | | | | | | |

### Frequency Tables

| Frequencies for gender | | | | | | | | | | | |
| --- | --- | --- | --- | --- | --- | --- | --- | --- | --- | --- | --- |
| individual | | gender | | Frequency | | Percent | | Valid Percent | | Cumulative Percent | |
| Alice |  | female |  | 20 |  | 100.000 |  | 100.000 |  | 100.000 |  |
|  |  | male |  | 0 |  | 0.000 |  | 0.000 |  | 100.000 |  |
|  |  | Missing |  | 0 |  | 0.000 |  |  |  |  |  |
|  |  | Total |  | 20 |  | 100.000 |  |  |  |  |  |
| Aminah |  | female |  | 26 |  | 100.000 |  | 100.000 |  | 100.000 |  |
|  |  | male |  | 0 |  | 0.000 |  | 0.000 |  | 100.000 |  |
|  |  | Missing |  | 0 |  | 0.000 |  |  |  |  |  |
|  |  | Total |  | 26 |  | 100.000 |  |  |  |  |  |
| Anto |  | female |  | 0 |  | 0.000 |  | 0.000 |  | 0.000 |  |
|  |  | male |  | 5 |  | 100.000 |  | 100.000 |  | 100.000 |  |
|  |  | Missing |  | 0 |  | 0.000 |  |  |  |  |  |
|  |  | Total |  | 5 |  | 100.000 |  |  |  |  |  |
| Asny |  | female |  | 15 |  | 100.000 |  | 100.000 |  | 100.000 |  |
|  |  | male |  | 0 |  | 0.000 |  | 0.000 |  | 100.000 |  |
|  |  | Missing |  | 0 |  | 0.000 |  |  |  |  |  |
|  |  | Total |  | 15 |  | 100.000 |  |  |  |  |  |
| Bagong |  | female |  | 0 |  | 0.000 |  | 0.000 |  | 0.000 |  |
|  |  | male |  | 101 |  | 100.000 |  | 100.000 |  | 100.000 |  |
|  |  | Missing |  | 0 |  | 0.000 |  |  |  |  |  |
|  |  | Total |  | 101 |  | 100.000 |  |  |  |  |  |
| Bendot |  | female |  | 0 |  | 0.000 |  | 0.000 |  | 0.000 |  |
|  |  | male |  | 65 |  | 100.000 |  | 100.000 |  | 100.000 |  |
|  |  | Missing |  | 0 |  | 0.000 |  |  |  |  |  |
|  |  | Total |  | 65 |  | 100.000 |  |  |  |  |  |
| Berani |  | female |  | 51 |  | 100.000 |  | 100.000 |  | 100.000 |  |
|  |  | male |  | 0 |  | 0.000 |  | 0.000 |  | 100.000 |  |
|  |  | Missing |  | 0 |  | 0.000 |  |  |  |  |  |
|  |  | Total |  | 51 |  | 100.000 |  |  |  |  |  |
| Beth |  | female |  | 46 |  | 100.000 |  | 100.000 |  | 100.000 |  |
|  |  | male |  | 0 |  | 0.000 |  | 0.000 |  | 100.000 |  |
|  |  | Missing |  | 0 |  | 0.000 |  |  |  |  |  |
|  |  | Total |  | 46 |  | 100.000 |  |  |  |  |  |
| Bibi |  | female |  | 13 |  | 100.000 |  | 100.000 |  | 100.000 |  |
|  |  | male |  | 0 |  | 0.000 |  | 0.000 |  | 100.000 |  |
|  |  | Missing |  | 0 |  | 0.000 |  |  |  |  |  |
|  |  | Total |  | 13 |  | 100.000 |  |  |  |  |  |
| Bintang |  | female |  | 93 |  | 100.000 |  | 100.000 |  | 100.000 |  |
|  |  | male |  | 0 |  | 0.000 |  | 0.000 |  | 100.000 |  |
|  |  | Missing |  | 0 |  | 0.000 |  |  |  |  |  |
|  |  | Total |  | 93 |  | 100.000 |  |  |  |  |  |
| Brutus |  | female |  | 0 |  | 0.000 |  | 0.000 |  | 0.000 |  |
|  |  | male |  | 183 |  | 100.000 |  | 100.000 |  | 100.000 |  |
|  |  | Missing |  | 0 |  | 0.000 |  |  |  |  |  |
|  |  | Total |  | 183 |  | 100.000 |  |  |  |  |  |
| Chindy |  | female |  | 22 |  | 100.000 |  | 100.000 |  | 100.000 |  |
|  |  | male |  | 0 |  | 0.000 |  | 0.000 |  | 100.000 |  |
|  |  | Missing |  | 0 |  | 0.000 |  |  |  |  |  |
|  |  | Total |  | 22 |  | 100.000 |  |  |  |  |  |
| Codet |  | female |  | 0 |  | 0.000 |  | 0.000 |  | 0.000 |  |
|  |  | male |  | 32 |  | 100.000 |  | 100.000 |  | 100.000 |  |
|  |  | Missing |  | 0 |  | 0.000 |  |  |  |  |  |
|  |  | Total |  | 32 |  | 100.000 |  |  |  |  |  |
| Elly |  | female |  | 51 |  | 100.000 |  | 100.000 |  | 100.000 |  |
|  |  | male |  | 0 |  | 0.000 |  | 0.000 |  | 100.000 |  |
|  |  | Missing |  | 0 |  | 0.000 |  |  |  |  |  |
|  |  | Total |  | 51 |  | 100.000 |  |  |  |  |  |
| Fajar |  | female |  | 0 |  | 0.000 |  | 0.000 |  | 0.000 |  |
|  |  | male |  | 246 |  | 100.000 |  | 100.000 |  | 100.000 |  |
|  |  | Missing |  | 0 |  | 0.000 |  |  |  |  |  |
|  |  | Total |  | 246 |  | 100.000 |  |  |  |  |  |
| Feb |  | female |  | 4 |  | 100.000 |  | 100.000 |  | 100.000 |  |
|  |  | male |  | 0 |  | 0.000 |  | 0.000 |  | 100.000 |  |
|  |  | Missing |  | 0 |  | 0.000 |  |  |  |  |  |
|  |  | Total |  | 4 |  | 100.000 |  |  |  |  |  |
| Female |  | female |  | 19 |  | 100.000 |  | 100.000 |  | 100.000 |  |
|  |  | male |  | 0 |  | 0.000 |  | 0.000 |  | 100.000 |  |
|  |  | Missing |  | 0 |  | 0.000 |  |  |  |  |  |
|  |  | Total |  | 19 |  | 100.000 |  |  |  |  |  |
| Fio |  | female |  | 0 |  | 0.000 |  | 0.000 |  | 0.000 |  |
|  |  | male |  | 9 |  | 100.000 |  | 100.000 |  | 100.000 |  |
|  |  | Missing |  | 0 |  | 0.000 |  |  |  |  |  |
|  |  | Total |  | 9 |  | 100.000 |  |  |  |  |  |
| Flanged male |  | female |  | 0 |  | 0.000 |  | 0.000 |  | 0.000 |  |
|  |  | male |  | 6 |  | 100.000 |  | 100.000 |  | 100.000 |  |
|  |  | Missing |  | 0 |  | 0.000 |  |  |  |  |  |
|  |  | Total |  | 6 |  | 100.000 |  |  |  |  |  |
| Freddy |  | female |  | 0 |  | 0.000 |  | 0.000 |  | 0.000 |  |
|  |  | male |  | 1 |  | 100.000 |  | 100.000 |  | 100.000 |  |
|  |  | Missing |  | 0 |  | 0.000 |  |  |  |  |  |
|  |  | Total |  | 1 |  | 100.000 |  |  |  |  |  |
| Friska |  | female |  | 1 |  | 100.000 |  | 100.000 |  | 100.000 |  |
|  |  | male |  | 0 |  | 0.000 |  | 0.000 |  | 100.000 |  |
|  |  | Missing |  | 0 |  | 0.000 |  |  |  |  |  |
|  |  | Total |  | 1 |  | 100.000 |  |  |  |  |  |
| Fugit |  | female |  | 0 |  | 0.000 |  | 0.000 |  | 0.000 |  |
|  |  | male |  | 126 |  | 100.000 |  | 100.000 |  | 100.000 |  |
|  |  | Missing |  | 0 |  | 0.000 |  |  |  |  |  |
|  |  | Total |  | 126 |  | 100.000 |  |  |  |  |  |
| Gangstah |  | female |  | 0 |  | 0.000 |  | 0.000 |  | 0.000 |  |
|  |  | male |  | 17 |  | 100.000 |  | 100.000 |  | 100.000 |  |
|  |  | Missing |  | 0 |  | 0.000 |  |  |  |  |  |
|  |  | Total |  | 17 |  | 100.000 |  |  |  |  |  |
| Gordon |  | female |  | 0 |  | 0.000 |  | 0.000 |  | 0.000 |  |
|  |  | male |  | 52 |  | 100.000 |  | 100.000 |  | 100.000 |  |
|  |  | Missing |  | 0 |  | 0.000 |  |  |  |  |  |
|  |  | Total |  | 52 |  | 100.000 |  |  |  |  |  |
| Gracia |  | female |  | 54 |  | 100.000 |  | 100.000 |  | 100.000 |  |
|  |  | male |  | 0 |  | 0.000 |  | 0.000 |  | 100.000 |  |
|  |  | Missing |  | 0 |  | 0.000 |  |  |  |  |  |
|  |  | Total |  | 54 |  | 100.000 |  |  |  |  |  |
| Gretel |  | female |  | 36 |  | 100.000 |  | 100.000 |  | 100.000 |  |
|  |  | male |  | 0 |  | 0.000 |  | 0.000 |  | 100.000 |  |
|  |  | Missing |  | 0 |  | 0.000 |  |  |  |  |  |
|  |  | Total |  | 36 |  | 100.000 |  |  |  |  |  |
| Henk |  | female |  | 0 |  | 0.000 |  | 0.000 |  | 0.000 |  |
|  |  | male |  | 4 |  | 100.000 |  | 100.000 |  | 100.000 |  |
|  |  | Missing |  | 0 |  | 0.000 |  |  |  |  |  |
|  |  | Total |  | 4 |  | 100.000 |  |  |  |  |  |
| Icarus |  | female |  | 0 |  | 0.000 |  | 0.000 |  | 0.000 |  |
|  |  | male |  | 5 |  | 100.000 |  | 100.000 |  | 100.000 |  |
|  |  | Missing |  | 0 |  | 0.000 |  |  |  |  |  |
|  |  | Total |  | 5 |  | 100.000 |  |  |  |  |  |
| Imp |  | female |  | 0 |  | 0.000 |  | 0.000 |  | 0.000 |  |
|  |  | male |  | 49 |  | 100.000 |  | 100.000 |  | 100.000 |  |
|  |  | Missing |  | 0 |  | 0.000 |  |  |  |  |  |
|  |  | Total |  | 49 |  | 100.000 |  |  |  |  |  |
| Indah |  | female |  | 17 |  | 100.000 |  | 100.000 |  | 100.000 |  |
|  |  | male |  | 0 |  | 0.000 |  | 0.000 |  | 100.000 |  |
|  |  | Missing |  | 0 |  | 0.000 |  |  |  |  |  |
|  |  | Total |  | 17 |  | 100.000 |  |  |  |  |  |
| Indi |  | female |  | 20 |  | 100.000 |  | 100.000 |  | 100.000 |  |
|  |  | male |  | 0 |  | 0.000 |  | 0.000 |  | 100.000 |  |
|  |  | Missing |  | 0 |  | 0.000 |  |  |  |  |  |
|  |  | Total |  | 20 |  | 100.000 |  |  |  |  |  |
| Irma |  | female |  | 1590 |  | 100.000 |  | 100.000 |  | 100.000 |  |
|  |  | male |  | 0 |  | 0.000 |  | 0.000 |  | 100.000 |  |
|  |  | Missing |  | 0 |  | 0.000 |  |  |  |  |  |
|  |  | Total |  | 1590 |  | 100.000 |  |  |  |  |  |
| James |  | female |  | 0 |  | 0.000 |  | 0.000 |  | 0.000 |  |
|  |  | male |  | 7 |  | 100.000 |  | 100.000 |  | 100.000 |  |
|  |  | Missing |  | 0 |  | 0.000 |  |  |  |  |  |
|  |  | Total |  | 7 |  | 100.000 |  |  |  |  |  |
| Janda Tua |  | female |  | 3 |  | 100.000 |  | 100.000 |  | 100.000 |  |
|  |  | male |  | 0 |  | 0.000 |  | 0.000 |  | 100.000 |  |
|  |  | Missing |  | 0 |  | 0.000 |  |  |  |  |  |
|  |  | Total |  | 3 |  | 100.000 |  |  |  |  |  |
| Jinak |  | female |  | 41 |  | 100.000 |  | 100.000 |  | 100.000 |  |
|  |  | male |  | 0 |  | 0.000 |  | 0.000 |  | 100.000 |  |
|  |  | Missing |  | 0 |  | 0.000 |  |  |  |  |  |
|  |  | Total |  | 41 |  | 100.000 |  |  |  |  |  |
| Joy |  | female |  | 2 |  | 100.000 |  | 100.000 |  | 100.000 |  |
|  |  | male |  | 0 |  | 0.000 |  | 0.000 |  | 100.000 |  |
|  |  | Missing |  | 0 |  | 0.000 |  |  |  |  |  |
|  |  | Total |  | 2 |  | 100.000 |  |  |  |  |  |
| Juni |  | female |  | 4 |  | 100.000 |  | 100.000 |  | 100.000 |  |
|  |  | male |  | 0 |  | 0.000 |  | 0.000 |  | 100.000 |  |
|  |  | Missing |  | 0 |  | 0.000 |  |  |  |  |  |
|  |  | Total |  | 4 |  | 100.000 |  |  |  |  |  |
| Juno |  | female |  | 14 |  | 100.000 |  | 100.000 |  | 100.000 |  |
|  |  | male |  | 0 |  | 0.000 |  | 0.000 |  | 100.000 |  |
|  |  | Missing |  | 0 |  | 0.000 |  |  |  |  |  |
|  |  | Total |  | 14 |  | 100.000 |  |  |  |  |  |
| Kacil |  | female |  | 5 |  | 100.000 |  | 100.000 |  | 100.000 |  |
|  |  | male |  | 0 |  | 0.000 |  | 0.000 |  | 100.000 |  |
|  |  | Missing |  | 0 |  | 0.000 |  |  |  |  |  |
|  |  | Total |  | 5 |  | 100.000 |  |  |  |  |  |
| Kan |  | female |  | 0 |  | 0.000 |  | 0.000 |  | 0.000 |  |
|  |  | male |  | 2 |  | 100.000 |  | 100.000 |  | 100.000 |  |
|  |  | Missing |  | 0 |  | 0.000 |  |  |  |  |  |
|  |  | Total |  | 2 |  | 100.000 |  |  |  |  |  |
| Kasi |  | female |  | 159 |  | 100.000 |  | 100.000 |  | 100.000 |  |
|  |  | male |  | 0 |  | 0.000 |  | 0.000 |  | 100.000 |  |
|  |  | Missing |  | 0 |  | 0.000 |  |  |  |  |  |
|  |  | Total |  | 159 |  | 100.000 |  |  |  |  |  |
| Kay |  | female |  | 0 |  | 0.000 |  | 0.000 |  | 0.000 |  |
|  |  | male |  | 111 |  | 100.000 |  | 100.000 |  | 100.000 |  |
|  |  | Missing |  | 0 |  | 0.000 |  |  |  |  |  |
|  |  | Total |  | 111 |  | 100.000 |  |  |  |  |  |
| Keri |  | female |  | 6 |  | 100.000 |  | 100.000 |  | 100.000 |  |
|  |  | male |  | 0 |  | 0.000 |  | 0.000 |  | 100.000 |  |
|  |  | Missing |  | 0 |  | 0.000 |  |  |  |  |  |
|  |  | Total |  | 6 |  | 100.000 |  |  |  |  |  |
| Keto |  | female |  | 0 |  | 0.000 |  | 0.000 |  | 0.000 |  |
|  |  | male |  | 1 |  | 100.000 |  | 100.000 |  | 100.000 |  |
|  |  | Missing |  | 0 |  | 0.000 |  |  |  |  |  |
|  |  | Total |  | 1 |  | 100.000 |  |  |  |  |  |
| Kondor |  | female |  | 38 |  | 100.000 |  | 100.000 |  | 100.000 |  |
|  |  | male |  | 0 |  | 0.000 |  | 0.000 |  | 100.000 |  |
|  |  | Missing |  | 0 |  | 0.000 |  |  |  |  |  |
|  |  | Total |  | 38 |  | 100.000 |  |  |  |  |  |
| Kundur |  | female |  | 0 |  | 0.000 |  | 0.000 |  | 0.000 |  |
|  |  | male |  | 42 |  | 100.000 |  | 100.000 |  | 100.000 |  |
|  |  | Missing |  | 0 |  | 0.000 |  |  |  |  |  |
|  |  | Total |  | 42 |  | 100.000 |  |  |  |  |  |
| Madalena |  | female |  | 15 |  | 100.000 |  | 100.000 |  | 100.000 |  |
|  |  | male |  | 0 |  | 0.000 |  | 0.000 |  | 100.000 |  |
|  |  | Missing |  | 0 |  | 0.000 |  |  |  |  |  |
|  |  | Total |  | 15 |  | 100.000 |  |  |  |  |  |
| Malé |  | female |  | 104 |  | 100.000 |  | 100.000 |  | 100.000 |  |
|  |  | male |  | 0 |  | 0.000 |  | 0.000 |  | 100.000 |  |
|  |  | Missing |  | 0 |  | 0.000 |  |  |  |  |  |
|  |  | Total |  | 104 |  | 100.000 |  |  |  |  |  |
| Mindi |  | female |  | 17 |  | 100.000 |  | 100.000 |  | 100.000 |  |
|  |  | male |  | 0 |  | 0.000 |  | 0.000 |  | 100.000 |  |
|  |  | Missing |  | 0 |  | 0.000 |  |  |  |  |  |
|  |  | Total |  | 17 |  | 100.000 |  |  |  |  |  |
| Ompung |  | female |  | 0 |  | 0.000 |  | 0.000 |  | 0.000 |  |
|  |  | male |  | 263 |  | 100.000 |  | 100.000 |  | 100.000 |  |
|  |  | Missing |  | 0 |  | 0.000 |  |  |  |  |  |
|  |  | Total |  | 263 |  | 100.000 |  |  |  |  |  |
| Pensi |  | female |  | 15 |  | 100.000 |  | 100.000 |  | 100.000 |  |
|  |  | male |  | 0 |  | 0.000 |  | 0.000 |  | 100.000 |  |
|  |  | Missing |  | 0 |  | 0.000 |  |  |  |  |  |
|  |  | Total |  | 15 |  | 100.000 |  |  |  |  |  |
| Peot |  | female |  | 8 |  | 100.000 |  | 100.000 |  | 100.000 |  |
|  |  | male |  | 0 |  | 0.000 |  | 0.000 |  | 100.000 |  |
|  |  | Missing |  | 0 |  | 0.000 |  |  |  |  |  |
|  |  | Total |  | 8 |  | 100.000 |  |  |  |  |  |
| Prabu |  | female |  | 0 |  | 0.000 |  | 0.000 |  | 0.000 |  |
|  |  | male |  | 80 |  | 100.000 |  | 100.000 |  | 100.000 |  |
|  |  | Missing |  | 0 |  | 0.000 |  |  |  |  |  |
|  |  | Total |  | 80 |  | 100.000 |  |  |  |  |  |
| Raffi |  | female |  | 61 |  | 100.000 |  | 100.000 |  | 100.000 |  |
|  |  | male |  | 0 |  | 0.000 |  | 0.000 |  | 100.000 |  |
|  |  | Missing |  | 0 |  | 0.000 |  |  |  |  |  |
|  |  | Total |  | 61 |  | 100.000 |  |  |  |  |  |
| Rambo |  | female |  | 1 |  | 3.448 |  | 3.448 |  | 3.448 |  |
|  |  | male |  | 28 |  | 96.552 |  | 96.552 |  | 100.000 |  |
|  |  | Missing |  | 0 |  | 0.000 |  |  |  |  |  |
|  |  | Total |  | 29 |  | 100.000 |  |  |  |  |  |
| Ronaldo |  | female |  | 0 |  | 0.000 |  | 0.000 |  | 0.000 |  |
|  |  | male |  | 1 |  | 100.000 |  | 100.000 |  | 100.000 |  |
|  |  | Missing |  | 0 |  | 0.000 |  |  |  |  |  |
|  |  | Total |  | 1 |  | 100.000 |  |  |  |  |  |
| Salvador |  | female |  | 0 |  | 0.000 |  | 0.000 |  | 0.000 |  |
|  |  | male |  | 46 |  | 100.000 |  | 100.000 |  | 100.000 |  |
|  |  | Missing |  | 0 |  | 0.000 |  |  |  |  |  |
|  |  | Total |  | 46 |  | 100.000 |  |  |  |  |  |
| Suci |  | female |  | 5 |  | 100.000 |  | 100.000 |  | 100.000 |  |
|  |  | male |  | 0 |  | 0.000 |  | 0.000 |  | 100.000 |  |
|  |  | Missing |  | 0 |  | 0.000 |  |  |  |  |  |
|  |  | Total |  | 5 |  | 100.000 |  |  |  |  |  |
| Sultan |  | female |  | 0 |  | 0.000 |  | 0.000 |  | 0.000 |  |
|  |  | male |  | 106 |  | 100.000 |  | 100.000 |  | 100.000 |  |
|  |  | Missing |  | 0 |  | 0.000 |  |  |  |  |  |
|  |  | Total |  | 106 |  | 100.000 |  |  |  |  |  |
| Sumi |  | female |  | 32 |  | 100.000 |  | 100.000 |  | 100.000 |  |
|  |  | male |  | 0 |  | 0.000 |  | 0.000 |  | 100.000 |  |
|  |  | Missing |  | 0 |  | 0.000 |  |  |  |  |  |
|  |  | Total |  | 32 |  | 100.000 |  |  |  |  |  |
| Teju |  | female |  | 0 |  | 0.000 |  | 0.000 |  | 0.000 |  |
|  |  | male |  | 22 |  | 100.000 |  | 100.000 |  | 100.000 |  |
|  |  | Missing |  | 0 |  | 0.000 |  |  |  |  |  |
|  |  | Total |  | 22 |  | 100.000 |  |  |  |  |  |
| Teresia |  | female |  | 28 |  | 100.000 |  | 100.000 |  | 100.000 |  |
|  |  | male |  | 0 |  | 0.000 |  | 0.000 |  | 100.000 |  |
|  |  | Missing |  | 0 |  | 0.000 |  |  |  |  |  |
|  |  | Total |  | 28 |  | 100.000 |  |  |  |  |  |
| Timi |  | female |  | 3 |  | 100.000 |  | 100.000 |  | 100.000 |  |
|  |  | male |  | 0 |  | 0.000 |  | 0.000 |  | 100.000 |  |
|  |  | Missing |  | 0 |  | 0.000 |  |  |  |  |  |
|  |  | Total |  | 3 |  | 100.000 |  |  |  |  |  |
| Tina |  | female |  | 316 |  | 100.000 |  | 100.000 |  | 100.000 |  |
|  |  | male |  | 0 |  | 0.000 |  | 0.000 |  | 100.000 |  |
|  |  | Missing |  | 0 |  | 0.000 |  |  |  |  |  |
|  |  | Total |  | 316 |  | 100.000 |  |  |  |  |  |
| Travor |  | female |  | 0 |  | 0.000 |  | 0.000 |  | 0.000 |  |
|  |  | male |  | 1 |  | 100.000 |  | 100.000 |  | 100.000 |  |
|  |  | Missing |  | 0 |  | 0.000 |  |  |  |  |  |
|  |  | Total |  | 1 |  | 100.000 |  |  |  |  |  |
| Umi |  | female |  | 2 |  | 100.000 |  | 100.000 |  | 100.000 |  |
|  |  | male |  | 0 |  | 0.000 |  | 0.000 |  | 100.000 |  |
|  |  | Missing |  | 0 |  | 0.000 |  |  |  |  |  |
|  |  | Total |  | 2 |  | 100.000 |  |  |  |  |  |
| Unflm |  | female |  | 0 |  | 0.000 |  | 0.000 |  | 0.000 |  |
|  |  | male |  | 32 |  | 100.000 |  | 100.000 |  | 100.000 |  |
|  |  | Missing |  | 0 |  | 0.000 |  |  |  |  |  |
|  |  | Total |  | 32 |  | 100.000 |  |  |  |  |  |
| Uok |  | female |  | 0 |  | 0.000 |  | 0.000 |  | 0.000 |  |
|  |  | male |  | 49 |  | 100.000 |  | 100.000 |  | 100.000 |  |
|  |  | Missing |  | 0 |  | 0.000 |  |  |  |  |  |
|  |  | Total |  | 49 |  | 100.000 |  |  |  |  |  |
| Vulcan |  | female |  | 0 |  | 0.000 |  | 0.000 |  | 0.000 |  |
|  |  | male |  | 2 |  | 100.000 |  | 100.000 |  | 100.000 |  |
|  |  | Missing |  | 0 |  | 0.000 |  |  |  |  |  |
|  |  | Total |  | 2 |  | 100.000 |  |  |  |  |  |
| Walimah |  | female |  | 100 |  | 100.000 |  | 100.000 |  | 100.000 |  |
|  |  | male |  | 0 |  | 0.000 |  | 0.000 |  | 100.000 |  |
|  |  | Missing |  | 0 |  | 0.000 |  |  |  |  |  |
|  |  | Total |  | 100 |  | 100.000 |  |  |  |  |  |
| Wulan |  | female |  | 69 |  | 100.000 |  | 100.000 |  | 100.000 |  |
|  |  | male |  | 0 |  | 0.000 |  | 0.000 |  | 100.000 |  |
|  |  | Missing |  | 0 |  | 0.000 |  |  |  |  |  |
|  |  | Total |  | 69 |  | 100.000 |  |  |  |  |  |
| XL |  | female |  | 0 |  | 0.000 |  | 0.000 |  | 0.000 |  |
|  |  | male |  | 41 |  | 100.000 |  | 100.000 |  | 100.000 |  |
|  |  | Missing |  | 0 |  | 0.000 |  |  |  |  |  |
|  |  | Total |  | 41 |  | 100.000 |  |  |  |  |  |
| Xenix |  | female |  | 0 |  | 0.000 |  | 0.000 |  | 0.000 |  |
|  |  | male |  | 8 |  | 100.000 |  | 100.000 |  | 100.000 |  |
|  |  | Missing |  | 0 |  | 0.000 |  |  |  |  |  |
|  |  | Total |  | 8 |  | 100.000 |  |  |  |  |  |
| Yanti |  | female |  | 414 |  | 100.000 |  | 100.000 |  | 100.000 |  |
|  |  | male |  | 0 |  | 0.000 |  | 0.000 |  | 100.000 |  |
|  |  | Missing |  | 0 |  | 0.000 |  |  |  |  |  |
|  |  | Total |  | 414 |  | 100.000 |  |  |  |  |  |
| Zeus |  | female |  | 0 |  | 0.000 |  | 0.000 |  | 0.000 |  |
|  |  | male |  | 1 |  | 100.000 |  | 100.000 |  | 100.000 |  |
|  |  | Missing |  | 0 |  | 0.000 |  |  |  |  |  |
|  |  | Total |  | 1 |  | 100.000 |  |  |  |  |  |
| Zorro |  | female |  | 0 |  | 0.000 |  | 0.000 |  | 0.000 |  |
|  |  | male |  | 11 |  | 100.000 |  | 100.000 |  | 100.000 |  |
|  |  | Missing |  | 0 |  | 0.000 |  |  |  |  |  |
|  |  | Total |  | 11 |  | 100.000 |  |  |  |  |  |
|  | | | | | | | | | | | |

| Frequencies for age-sex class | | | | | | | | | | | |
| --- | --- | --- | --- | --- | --- | --- | --- | --- | --- | --- | --- |
| individual | | age-sex class | | Frequency | | Percent | | Valid Percent | | Cumulative Percent | |
| Alice |  | adolescent |  | 0 |  | 0.000 |  | 0.000 |  | 0.000 |  |
|  |  | female with infant |  | 20 |  | 100.000 |  | 100.000 |  | 100.000 |  |
|  |  | flanged male |  | 0 |  | 0.000 |  | 0.000 |  | 100.000 |  |
|  |  | infant |  | 0 |  | 0.000 |  | 0.000 |  | 100.000 |  |
|  |  | unflanged male |  | 0 |  | 0.000 |  | 0.000 |  | 100.000 |  |
|  |  | Missing |  | 0 |  | 0.000 |  |  |  |  |  |
|  |  | Total |  | 20 |  | 100.000 |  |  |  |  |  |
| Aminah |  | adolescent |  | 26 |  | 100.000 |  | 100.000 |  | 100.000 |  |
|  |  | female with infant |  | 0 |  | 0.000 |  | 0.000 |  | 100.000 |  |
|  |  | flanged male |  | 0 |  | 0.000 |  | 0.000 |  | 100.000 |  |
|  |  | infant |  | 0 |  | 0.000 |  | 0.000 |  | 100.000 |  |
|  |  | unflanged male |  | 0 |  | 0.000 |  | 0.000 |  | 100.000 |  |
|  |  | Missing |  | 0 |  | 0.000 |  |  |  |  |  |
|  |  | Total |  | 26 |  | 100.000 |  |  |  |  |  |
| Anto |  | adolescent |  | 0 |  | 0.000 |  | 0.000 |  | 0.000 |  |
|  |  | female with infant |  | 0 |  | 0.000 |  | 0.000 |  | 0.000 |  |
|  |  | flanged male |  | 0 |  | 0.000 |  | 0.000 |  | 0.000 |  |
|  |  | infant |  | 0 |  | 0.000 |  | 0.000 |  | 0.000 |  |
|  |  | unflanged male |  | 5 |  | 100.000 |  | 100.000 |  | 100.000 |  |
|  |  | Missing |  | 0 |  | 0.000 |  |  |  |  |  |
|  |  | Total |  | 5 |  | 100.000 |  |  |  |  |  |
| Asny |  | adolescent |  | 0 |  | 0.000 |  | 0.000 |  | 0.000 |  |
|  |  | female with infant |  | 15 |  | 100.000 |  | 100.000 |  | 100.000 |  |
|  |  | flanged male |  | 0 |  | 0.000 |  | 0.000 |  | 100.000 |  |
|  |  | infant |  | 0 |  | 0.000 |  | 0.000 |  | 100.000 |  |
|  |  | unflanged male |  | 0 |  | 0.000 |  | 0.000 |  | 100.000 |  |
|  |  | Missing |  | 0 |  | 0.000 |  |  |  |  |  |
|  |  | Total |  | 15 |  | 100.000 |  |  |  |  |  |
| Bagong |  | adolescent |  | 0 |  | 0.000 |  | 0.000 |  | 0.000 |  |
|  |  | female with infant |  | 0 |  | 0.000 |  | 0.000 |  | 0.000 |  |
|  |  | flanged male |  | 101 |  | 100.000 |  | 100.000 |  | 100.000 |  |
|  |  | infant |  | 0 |  | 0.000 |  | 0.000 |  | 100.000 |  |
|  |  | unflanged male |  | 0 |  | 0.000 |  | 0.000 |  | 100.000 |  |
|  |  | Missing |  | 0 |  | 0.000 |  |  |  |  |  |
|  |  | Total |  | 101 |  | 100.000 |  |  |  |  |  |
| Bendot |  | adolescent |  | 0 |  | 0.000 |  | 0.000 |  | 0.000 |  |
|  |  | female with infant |  | 0 |  | 0.000 |  | 0.000 |  | 0.000 |  |
|  |  | flanged male |  | 65 |  | 100.000 |  | 100.000 |  | 100.000 |  |
|  |  | infant |  | 0 |  | 0.000 |  | 0.000 |  | 100.000 |  |
|  |  | unflanged male |  | 0 |  | 0.000 |  | 0.000 |  | 100.000 |  |
|  |  | Missing |  | 0 |  | 0.000 |  |  |  |  |  |
|  |  | Total |  | 65 |  | 100.000 |  |  |  |  |  |
| Berani |  | adolescent |  | 0 |  | 0.000 |  | 0.000 |  | 0.000 |  |
|  |  | female with infant |  | 0 |  | 0.000 |  | 0.000 |  | 0.000 |  |
|  |  | flanged male |  | 0 |  | 0.000 |  | 0.000 |  | 0.000 |  |
|  |  | infant |  | 51 |  | 100.000 |  | 100.000 |  | 100.000 |  |
|  |  | unflanged male |  | 0 |  | 0.000 |  | 0.000 |  | 100.000 |  |
|  |  | Missing |  | 0 |  | 0.000 |  |  |  |  |  |
|  |  | Total |  | 51 |  | 100.000 |  |  |  |  |  |
| Beth |  | adolescent |  | 0 |  | 0.000 |  | 0.000 |  | 0.000 |  |
|  |  | female with infant |  | 46 |  | 100.000 |  | 100.000 |  | 100.000 |  |
|  |  | flanged male |  | 0 |  | 0.000 |  | 0.000 |  | 100.000 |  |
|  |  | infant |  | 0 |  | 0.000 |  | 0.000 |  | 100.000 |  |
|  |  | unflanged male |  | 0 |  | 0.000 |  | 0.000 |  | 100.000 |  |
|  |  | Missing |  | 0 |  | 0.000 |  |  |  |  |  |
|  |  | Total |  | 46 |  | 100.000 |  |  |  |  |  |
| Bibi |  | adolescent |  | 0 |  | 0.000 |  | 0.000 |  | 0.000 |  |
|  |  | female with infant |  | 13 |  | 100.000 |  | 100.000 |  | 100.000 |  |
|  |  | flanged male |  | 0 |  | 0.000 |  | 0.000 |  | 100.000 |  |
|  |  | infant |  | 0 |  | 0.000 |  | 0.000 |  | 100.000 |  |
|  |  | unflanged male |  | 0 |  | 0.000 |  | 0.000 |  | 100.000 |  |
|  |  | Missing |  | 0 |  | 0.000 |  |  |  |  |  |
|  |  | Total |  | 13 |  | 100.000 |  |  |  |  |  |
| Bintang |  | adolescent |  | 0 |  | 0.000 |  | 0.000 |  | 0.000 |  |
|  |  | female with infant |  | 93 |  | 100.000 |  | 100.000 |  | 100.000 |  |
|  |  | flanged male |  | 0 |  | 0.000 |  | 0.000 |  | 100.000 |  |
|  |  | infant |  | 0 |  | 0.000 |  | 0.000 |  | 100.000 |  |
|  |  | unflanged male |  | 0 |  | 0.000 |  | 0.000 |  | 100.000 |  |
|  |  | Missing |  | 0 |  | 0.000 |  |  |  |  |  |
|  |  | Total |  | 93 |  | 100.000 |  |  |  |  |  |
| Brutus |  | adolescent |  | 0 |  | 0.000 |  | 0.000 |  | 0.000 |  |
|  |  | female with infant |  | 0 |  | 0.000 |  | 0.000 |  | 0.000 |  |
|  |  | flanged male |  | 0 |  | 0.000 |  | 0.000 |  | 0.000 |  |
|  |  | infant |  | 0 |  | 0.000 |  | 0.000 |  | 0.000 |  |
|  |  | unflanged male |  | 183 |  | 100.000 |  | 100.000 |  | 100.000 |  |
|  |  | Missing |  | 0 |  | 0.000 |  |  |  |  |  |
|  |  | Total |  | 183 |  | 100.000 |  |  |  |  |  |
| Chindy |  | adolescent |  | 22 |  | 100.000 |  | 100.000 |  | 100.000 |  |
|  |  | female with infant |  | 0 |  | 0.000 |  | 0.000 |  | 100.000 |  |
|  |  | flanged male |  | 0 |  | 0.000 |  | 0.000 |  | 100.000 |  |
|  |  | infant |  | 0 |  | 0.000 |  | 0.000 |  | 100.000 |  |
|  |  | unflanged male |  | 0 |  | 0.000 |  | 0.000 |  | 100.000 |  |
|  |  | Missing |  | 0 |  | 0.000 |  |  |  |  |  |
|  |  | Total |  | 22 |  | 100.000 |  |  |  |  |  |
| Codet |  | adolescent |  | 0 |  | 0.000 |  | 0.000 |  | 0.000 |  |
|  |  | female with infant |  | 0 |  | 0.000 |  | 0.000 |  | 0.000 |  |
|  |  | flanged male |  | 32 |  | 100.000 |  | 100.000 |  | 100.000 |  |
|  |  | infant |  | 0 |  | 0.000 |  | 0.000 |  | 100.000 |  |
|  |  | unflanged male |  | 0 |  | 0.000 |  | 0.000 |  | 100.000 |  |
|  |  | Missing |  | 0 |  | 0.000 |  |  |  |  |  |
|  |  | Total |  | 32 |  | 100.000 |  |  |  |  |  |
| Elly |  | adolescent |  | 51 |  | 100.000 |  | 100.000 |  | 100.000 |  |
|  |  | female with infant |  | 0 |  | 0.000 |  | 0.000 |  | 100.000 |  |
|  |  | flanged male |  | 0 |  | 0.000 |  | 0.000 |  | 100.000 |  |
|  |  | infant |  | 0 |  | 0.000 |  | 0.000 |  | 100.000 |  |
|  |  | unflanged male |  | 0 |  | 0.000 |  | 0.000 |  | 100.000 |  |
|  |  | Missing |  | 0 |  | 0.000 |  |  |  |  |  |
|  |  | Total |  | 51 |  | 100.000 |  |  |  |  |  |
| Fajar |  | adolescent |  | 0 |  | 0.000 |  | 0.000 |  | 0.000 |  |
|  |  | female with infant |  | 0 |  | 0.000 |  | 0.000 |  | 0.000 |  |
|  |  | flanged male |  | 246 |  | 100.000 |  | 100.000 |  | 100.000 |  |
|  |  | infant |  | 0 |  | 0.000 |  | 0.000 |  | 100.000 |  |
|  |  | unflanged male |  | 0 |  | 0.000 |  | 0.000 |  | 100.000 |  |
|  |  | Missing |  | 0 |  | 0.000 |  |  |  |  |  |
|  |  | Total |  | 246 |  | 100.000 |  |  |  |  |  |
| Feb |  | adolescent |  | 0 |  | 0.000 |  | 0.000 |  | 0.000 |  |
|  |  | female with infant |  | 4 |  | 100.000 |  | 100.000 |  | 100.000 |  |
|  |  | flanged male |  | 0 |  | 0.000 |  | 0.000 |  | 100.000 |  |
|  |  | infant |  | 0 |  | 0.000 |  | 0.000 |  | 100.000 |  |
|  |  | unflanged male |  | 0 |  | 0.000 |  | 0.000 |  | 100.000 |  |
|  |  | Missing |  | 0 |  | 0.000 |  |  |  |  |  |
|  |  | Total |  | 4 |  | 100.000 |  |  |  |  |  |
| Female |  | adolescent |  | 19 |  | 100.000 |  | 100.000 |  | 100.000 |  |
|  |  | female with infant |  | 0 |  | 0.000 |  | 0.000 |  | 100.000 |  |
|  |  | flanged male |  | 0 |  | 0.000 |  | 0.000 |  | 100.000 |  |
|  |  | infant |  | 0 |  | 0.000 |  | 0.000 |  | 100.000 |  |
|  |  | unflanged male |  | 0 |  | 0.000 |  | 0.000 |  | 100.000 |  |
|  |  | Missing |  | 0 |  | 0.000 |  |  |  |  |  |
|  |  | Total |  | 19 |  | 100.000 |  |  |  |  |  |
| Fio |  | adolescent |  | 0 |  | 0.000 |  | 0.000 |  | 0.000 |  |
|  |  | female with infant |  | 0 |  | 0.000 |  | 0.000 |  | 0.000 |  |
|  |  | flanged male |  | 0 |  | 0.000 |  | 0.000 |  | 0.000 |  |
|  |  | infant |  | 9 |  | 100.000 |  | 100.000 |  | 100.000 |  |
|  |  | unflanged male |  | 0 |  | 0.000 |  | 0.000 |  | 100.000 |  |
|  |  | Missing |  | 0 |  | 0.000 |  |  |  |  |  |
|  |  | Total |  | 9 |  | 100.000 |  |  |  |  |  |
| Flanged male |  | adolescent |  | 0 |  | 0.000 |  | 0.000 |  | 0.000 |  |
|  |  | female with infant |  | 0 |  | 0.000 |  | 0.000 |  | 0.000 |  |
|  |  | flanged male |  | 6 |  | 100.000 |  | 100.000 |  | 100.000 |  |
|  |  | infant |  | 0 |  | 0.000 |  | 0.000 |  | 100.000 |  |
|  |  | unflanged male |  | 0 |  | 0.000 |  | 0.000 |  | 100.000 |  |
|  |  | Missing |  | 0 |  | 0.000 |  |  |  |  |  |
|  |  | Total |  | 6 |  | 100.000 |  |  |  |  |  |
| Freddy |  | adolescent |  | 0 |  | 0.000 |  | 0.000 |  | 0.000 |  |
|  |  | female with infant |  | 0 |  | 0.000 |  | 0.000 |  | 0.000 |  |
|  |  | flanged male |  | 0 |  | 0.000 |  | 0.000 |  | 0.000 |  |
|  |  | infant |  | 1 |  | 100.000 |  | 100.000 |  | 100.000 |  |
|  |  | unflanged male |  | 0 |  | 0.000 |  | 0.000 |  | 100.000 |  |
|  |  | Missing |  | 0 |  | 0.000 |  |  |  |  |  |
|  |  | Total |  | 1 |  | 100.000 |  |  |  |  |  |
| Friska |  | adolescent |  | 0 |  | 0.000 |  | 0.000 |  | 0.000 |  |
|  |  | female with infant |  | 1 |  | 100.000 |  | 100.000 |  | 100.000 |  |
|  |  | flanged male |  | 0 |  | 0.000 |  | 0.000 |  | 100.000 |  |
|  |  | infant |  | 0 |  | 0.000 |  | 0.000 |  | 100.000 |  |
|  |  | unflanged male |  | 0 |  | 0.000 |  | 0.000 |  | 100.000 |  |
|  |  | Missing |  | 0 |  | 0.000 |  |  |  |  |  |
|  |  | Total |  | 1 |  | 100.000 |  |  |  |  |  |
| Fugit |  | adolescent |  | 0 |  | 0.000 |  | 0.000 |  | 0.000 |  |
|  |  | female with infant |  | 0 |  | 0.000 |  | 0.000 |  | 0.000 |  |
|  |  | flanged male |  | 126 |  | 100.000 |  | 100.000 |  | 100.000 |  |
|  |  | infant |  | 0 |  | 0.000 |  | 0.000 |  | 100.000 |  |
|  |  | unflanged male |  | 0 |  | 0.000 |  | 0.000 |  | 100.000 |  |
|  |  | Missing |  | 0 |  | 0.000 |  |  |  |  |  |
|  |  | Total |  | 126 |  | 100.000 |  |  |  |  |  |
| Gangstah |  | adolescent |  | 0 |  | 0.000 |  | 0.000 |  | 0.000 |  |
|  |  | female with infant |  | 0 |  | 0.000 |  | 0.000 |  | 0.000 |  |
|  |  | flanged male |  | 0 |  | 0.000 |  | 0.000 |  | 0.000 |  |
|  |  | infant |  | 0 |  | 0.000 |  | 0.000 |  | 0.000 |  |
|  |  | unflanged male |  | 17 |  | 100.000 |  | 100.000 |  | 100.000 |  |
|  |  | Missing |  | 0 |  | 0.000 |  |  |  |  |  |
|  |  | Total |  | 17 |  | 100.000 |  |  |  |  |  |
| Gordon |  | adolescent |  | 0 |  | 0.000 |  | 0.000 |  | 0.000 |  |
|  |  | female with infant |  | 0 |  | 0.000 |  | 0.000 |  | 0.000 |  |
|  |  | flanged male |  | 0 |  | 0.000 |  | 0.000 |  | 0.000 |  |
|  |  | infant |  | 0 |  | 0.000 |  | 0.000 |  | 0.000 |  |
|  |  | unflanged male |  | 52 |  | 100.000 |  | 100.000 |  | 100.000 |  |
|  |  | Missing |  | 0 |  | 0.000 |  |  |  |  |  |
|  |  | Total |  | 52 |  | 100.000 |  |  |  |  |  |
| Gracia |  | adolescent |  | 0 |  | 0.000 |  | 0.000 |  | 0.000 |  |
|  |  | female with infant |  | 54 |  | 100.000 |  | 100.000 |  | 100.000 |  |
|  |  | flanged male |  | 0 |  | 0.000 |  | 0.000 |  | 100.000 |  |
|  |  | infant |  | 0 |  | 0.000 |  | 0.000 |  | 100.000 |  |
|  |  | unflanged male |  | 0 |  | 0.000 |  | 0.000 |  | 100.000 |  |
|  |  | Missing |  | 0 |  | 0.000 |  |  |  |  |  |
|  |  | Total |  | 54 |  | 100.000 |  |  |  |  |  |
| Gretel |  | adolescent |  | 36 |  | 100.000 |  | 100.000 |  | 100.000 |  |
|  |  | female with infant |  | 0 |  | 0.000 |  | 0.000 |  | 100.000 |  |
|  |  | flanged male |  | 0 |  | 0.000 |  | 0.000 |  | 100.000 |  |
|  |  | infant |  | 0 |  | 0.000 |  | 0.000 |  | 100.000 |  |
|  |  | unflanged male |  | 0 |  | 0.000 |  | 0.000 |  | 100.000 |  |
|  |  | Missing |  | 0 |  | 0.000 |  |  |  |  |  |
|  |  | Total |  | 36 |  | 100.000 |  |  |  |  |  |
| Henk |  | adolescent |  | 0 |  | 0.000 |  | 0.000 |  | 0.000 |  |
|  |  | female with infant |  | 0 |  | 0.000 |  | 0.000 |  | 0.000 |  |
|  |  | flanged male |  | 4 |  | 100.000 |  | 100.000 |  | 100.000 |  |
|  |  | infant |  | 0 |  | 0.000 |  | 0.000 |  | 100.000 |  |
|  |  | unflanged male |  | 0 |  | 0.000 |  | 0.000 |  | 100.000 |  |
|  |  | Missing |  | 0 |  | 0.000 |  |  |  |  |  |
|  |  | Total |  | 4 |  | 100.000 |  |  |  |  |  |
| Icarus |  | adolescent |  | 0 |  | 0.000 |  | 0.000 |  | 0.000 |  |
|  |  | female with infant |  | 0 |  | 0.000 |  | 0.000 |  | 0.000 |  |
|  |  | flanged male |  | 0 |  | 0.000 |  | 0.000 |  | 0.000 |  |
|  |  | infant |  | 5 |  | 100.000 |  | 100.000 |  | 100.000 |  |
|  |  | unflanged male |  | 0 |  | 0.000 |  | 0.000 |  | 100.000 |  |
|  |  | Missing |  | 0 |  | 0.000 |  |  |  |  |  |
|  |  | Total |  | 5 |  | 100.000 |  |  |  |  |  |
| Imp |  | adolescent |  | 0 |  | 0.000 |  | 0.000 |  | 0.000 |  |
|  |  | female with infant |  | 0 |  | 0.000 |  | 0.000 |  | 0.000 |  |
|  |  | flanged male |  | 0 |  | 0.000 |  | 0.000 |  | 0.000 |  |
|  |  | infant |  | 0 |  | 0.000 |  | 0.000 |  | 0.000 |  |
|  |  | unflanged male |  | 49 |  | 100.000 |  | 100.000 |  | 100.000 |  |
|  |  | Missing |  | 0 |  | 0.000 |  |  |  |  |  |
|  |  | Total |  | 49 |  | 100.000 |  |  |  |  |  |
| Indah |  | adolescent |  | 0 |  | 0.000 |  | 0.000 |  | 0.000 |  |
|  |  | female with infant |  | 0 |  | 0.000 |  | 0.000 |  | 0.000 |  |
|  |  | flanged male |  | 0 |  | 0.000 |  | 0.000 |  | 0.000 |  |
|  |  | infant |  | 17 |  | 100.000 |  | 100.000 |  | 100.000 |  |
|  |  | unflanged male |  | 0 |  | 0.000 |  | 0.000 |  | 100.000 |  |
|  |  | Missing |  | 0 |  | 0.000 |  |  |  |  |  |
|  |  | Total |  | 17 |  | 100.000 |  |  |  |  |  |
| Indi |  | adolescent |  | 0 |  | 0.000 |  | 0.000 |  | 0.000 |  |
|  |  | female with infant |  | 20 |  | 100.000 |  | 100.000 |  | 100.000 |  |
|  |  | flanged male |  | 0 |  | 0.000 |  | 0.000 |  | 100.000 |  |
|  |  | infant |  | 0 |  | 0.000 |  | 0.000 |  | 100.000 |  |
|  |  | unflanged male |  | 0 |  | 0.000 |  | 0.000 |  | 100.000 |  |
|  |  | Missing |  | 0 |  | 0.000 |  |  |  |  |  |
|  |  | Total |  | 20 |  | 100.000 |  |  |  |  |  |
| Irma |  | adolescent |  | 0 |  | 0.000 |  | 0.000 |  | 0.000 |  |
|  |  | female with infant |  | 1589 |  | 99.937 |  | 99.937 |  | 99.937 |  |
|  |  | flanged male |  | 1 |  | 0.063 |  | 0.063 |  | 100.000 |  |
|  |  | infant |  | 0 |  | 0.000 |  | 0.000 |  | 100.000 |  |
|  |  | unflanged male |  | 0 |  | 0.000 |  | 0.000 |  | 100.000 |  |
|  |  | Missing |  | 0 |  | 0.000 |  |  |  |  |  |
|  |  | Total |  | 1590 |  | 100.000 |  |  |  |  |  |
| James |  | adolescent |  | 0 |  | 0.000 |  | 0.000 |  | 0.000 |  |
|  |  | female with infant |  | 0 |  | 0.000 |  | 0.000 |  | 0.000 |  |
|  |  | flanged male |  | 0 |  | 0.000 |  | 0.000 |  | 0.000 |  |
|  |  | infant |  | 0 |  | 0.000 |  | 0.000 |  | 0.000 |  |
|  |  | unflanged male |  | 7 |  | 100.000 |  | 100.000 |  | 100.000 |  |
|  |  | Missing |  | 0 |  | 0.000 |  |  |  |  |  |
|  |  | Total |  | 7 |  | 100.000 |  |  |  |  |  |
| Janda Tua |  | adolescent |  | 0 |  | 0.000 |  | 0.000 |  | 0.000 |  |
|  |  | female with infant |  | 3 |  | 100.000 |  | 100.000 |  | 100.000 |  |
|  |  | flanged male |  | 0 |  | 0.000 |  | 0.000 |  | 100.000 |  |
|  |  | infant |  | 0 |  | 0.000 |  | 0.000 |  | 100.000 |  |
|  |  | unflanged male |  | 0 |  | 0.000 |  | 0.000 |  | 100.000 |  |
|  |  | Missing |  | 0 |  | 0.000 |  |  |  |  |  |
|  |  | Total |  | 3 |  | 100.000 |  |  |  |  |  |
| Jinak |  | adolescent |  | 0 |  | 0.000 |  | 0.000 |  | 0.000 |  |
|  |  | female with infant |  | 41 |  | 100.000 |  | 100.000 |  | 100.000 |  |
|  |  | flanged male |  | 0 |  | 0.000 |  | 0.000 |  | 100.000 |  |
|  |  | infant |  | 0 |  | 0.000 |  | 0.000 |  | 100.000 |  |
|  |  | unflanged male |  | 0 |  | 0.000 |  | 0.000 |  | 100.000 |  |
|  |  | Missing |  | 0 |  | 0.000 |  |  |  |  |  |
|  |  | Total |  | 41 |  | 100.000 |  |  |  |  |  |
| Joy |  | adolescent |  | 0 |  | 0.000 |  | 0.000 |  | 0.000 |  |
|  |  | female with infant |  | 0 |  | 0.000 |  | 0.000 |  | 0.000 |  |
|  |  | flanged male |  | 0 |  | 0.000 |  | 0.000 |  | 0.000 |  |
|  |  | infant |  | 2 |  | 100.000 |  | 100.000 |  | 100.000 |  |
|  |  | unflanged male |  | 0 |  | 0.000 |  | 0.000 |  | 100.000 |  |
|  |  | Missing |  | 0 |  | 0.000 |  |  |  |  |  |
|  |  | Total |  | 2 |  | 100.000 |  |  |  |  |  |
| Juni |  | adolescent |  | 4 |  | 100.000 |  | 100.000 |  | 100.000 |  |
|  |  | female with infant |  | 0 |  | 0.000 |  | 0.000 |  | 100.000 |  |
|  |  | flanged male |  | 0 |  | 0.000 |  | 0.000 |  | 100.000 |  |
|  |  | infant |  | 0 |  | 0.000 |  | 0.000 |  | 100.000 |  |
|  |  | unflanged male |  | 0 |  | 0.000 |  | 0.000 |  | 100.000 |  |
|  |  | Missing |  | 0 |  | 0.000 |  |  |  |  |  |
|  |  | Total |  | 4 |  | 100.000 |  |  |  |  |  |
| Juno |  | adolescent |  | 0 |  | 0.000 |  | 0.000 |  | 0.000 |  |
|  |  | female with infant |  | 14 |  | 100.000 |  | 100.000 |  | 100.000 |  |
|  |  | flanged male |  | 0 |  | 0.000 |  | 0.000 |  | 100.000 |  |
|  |  | infant |  | 0 |  | 0.000 |  | 0.000 |  | 100.000 |  |
|  |  | unflanged male |  | 0 |  | 0.000 |  | 0.000 |  | 100.000 |  |
|  |  | Missing |  | 0 |  | 0.000 |  |  |  |  |  |
|  |  | Total |  | 14 |  | 100.000 |  |  |  |  |  |
| Kacil |  | adolescent |  | 0 |  | 0.000 |  | 0.000 |  | 0.000 |  |
|  |  | female with infant |  | 0 |  | 0.000 |  | 0.000 |  | 0.000 |  |
|  |  | flanged male |  | 0 |  | 0.000 |  | 0.000 |  | 0.000 |  |
|  |  | infant |  | 5 |  | 100.000 |  | 100.000 |  | 100.000 |  |
|  |  | unflanged male |  | 0 |  | 0.000 |  | 0.000 |  | 100.000 |  |
|  |  | Missing |  | 0 |  | 0.000 |  |  |  |  |  |
|  |  | Total |  | 5 |  | 100.000 |  |  |  |  |  |
| Kan |  | adolescent |  | 0 |  | 0.000 |  | 0.000 |  | 0.000 |  |
|  |  | female with infant |  | 0 |  | 0.000 |  | 0.000 |  | 0.000 |  |
|  |  | flanged male |  | 0 |  | 0.000 |  | 0.000 |  | 0.000 |  |
|  |  | infant |  | 2 |  | 100.000 |  | 100.000 |  | 100.000 |  |
|  |  | unflanged male |  | 0 |  | 0.000 |  | 0.000 |  | 100.000 |  |
|  |  | Missing |  | 0 |  | 0.000 |  |  |  |  |  |
|  |  | Total |  | 2 |  | 100.000 |  |  |  |  |  |
| Kasi |  | adolescent |  | 0 |  | 0.000 |  | 0.000 |  | 0.000 |  |
|  |  | female with infant |  | 159 |  | 100.000 |  | 100.000 |  | 100.000 |  |
|  |  | flanged male |  | 0 |  | 0.000 |  | 0.000 |  | 100.000 |  |
|  |  | infant |  | 0 |  | 0.000 |  | 0.000 |  | 100.000 |  |
|  |  | unflanged male |  | 0 |  | 0.000 |  | 0.000 |  | 100.000 |  |
|  |  | Missing |  | 0 |  | 0.000 |  |  |  |  |  |
|  |  | Total |  | 159 |  | 100.000 |  |  |  |  |  |
| Kay |  | adolescent |  | 0 |  | 0.000 |  | 0.000 |  | 0.000 |  |
|  |  | female with infant |  | 0 |  | 0.000 |  | 0.000 |  | 0.000 |  |
|  |  | flanged male |  | 111 |  | 100.000 |  | 100.000 |  | 100.000 |  |
|  |  | infant |  | 0 |  | 0.000 |  | 0.000 |  | 100.000 |  |
|  |  | unflanged male |  | 0 |  | 0.000 |  | 0.000 |  | 100.000 |  |
|  |  | Missing |  | 0 |  | 0.000 |  |  |  |  |  |
|  |  | Total |  | 111 |  | 100.000 |  |  |  |  |  |
| Keri |  | adolescent |  | 0 |  | 0.000 |  | 0.000 |  | 0.000 |  |
|  |  | female with infant |  | 6 |  | 100.000 |  | 100.000 |  | 100.000 |  |
|  |  | flanged male |  | 0 |  | 0.000 |  | 0.000 |  | 100.000 |  |
|  |  | infant |  | 0 |  | 0.000 |  | 0.000 |  | 100.000 |  |
|  |  | unflanged male |  | 0 |  | 0.000 |  | 0.000 |  | 100.000 |  |
|  |  | Missing |  | 0 |  | 0.000 |  |  |  |  |  |
|  |  | Total |  | 6 |  | 100.000 |  |  |  |  |  |
| Keto |  | adolescent |  | 0 |  | 0.000 |  | 0.000 |  | 0.000 |  |
|  |  | female with infant |  | 0 |  | 0.000 |  | 0.000 |  | 0.000 |  |
|  |  | flanged male |  | 0 |  | 0.000 |  | 0.000 |  | 0.000 |  |
|  |  | infant |  | 0 |  | 0.000 |  | 0.000 |  | 0.000 |  |
|  |  | unflanged male |  | 1 |  | 100.000 |  | 100.000 |  | 100.000 |  |
|  |  | Missing |  | 0 |  | 0.000 |  |  |  |  |  |
|  |  | Total |  | 1 |  | 100.000 |  |  |  |  |  |
| Kondor |  | adolescent |  | 0 |  | 0.000 |  | 0.000 |  | 0.000 |  |
|  |  | female with infant |  | 0 |  | 0.000 |  | 0.000 |  | 0.000 |  |
|  |  | flanged male |  | 0 |  | 0.000 |  | 0.000 |  | 0.000 |  |
|  |  | infant |  | 38 |  | 100.000 |  | 100.000 |  | 100.000 |  |
|  |  | unflanged male |  | 0 |  | 0.000 |  | 0.000 |  | 100.000 |  |
|  |  | Missing |  | 0 |  | 0.000 |  |  |  |  |  |
|  |  | Total |  | 38 |  | 100.000 |  |  |  |  |  |
| Kundur |  | adolescent |  | 0 |  | 0.000 |  | 0.000 |  | 0.000 |  |
|  |  | female with infant |  | 0 |  | 0.000 |  | 0.000 |  | 0.000 |  |
|  |  | flanged male |  | 0 |  | 0.000 |  | 0.000 |  | 0.000 |  |
|  |  | infant |  | 0 |  | 0.000 |  | 0.000 |  | 0.000 |  |
|  |  | unflanged male |  | 42 |  | 100.000 |  | 100.000 |  | 100.000 |  |
|  |  | Missing |  | 0 |  | 0.000 |  |  |  |  |  |
|  |  | Total |  | 42 |  | 100.000 |  |  |  |  |  |
| Madalena |  | adolescent |  | 0 |  | 0.000 |  | 0.000 |  | 0.000 |  |
|  |  | female with infant |  | 15 |  | 100.000 |  | 100.000 |  | 100.000 |  |
|  |  | flanged male |  | 0 |  | 0.000 |  | 0.000 |  | 100.000 |  |
|  |  | infant |  | 0 |  | 0.000 |  | 0.000 |  | 100.000 |  |
|  |  | unflanged male |  | 0 |  | 0.000 |  | 0.000 |  | 100.000 |  |
|  |  | Missing |  | 0 |  | 0.000 |  |  |  |  |  |
|  |  | Total |  | 15 |  | 100.000 |  |  |  |  |  |
| Malé |  | adolescent |  | 0 |  | 0.000 |  | 0.000 |  | 0.000 |  |
|  |  | female with infant |  | 104 |  | 100.000 |  | 100.000 |  | 100.000 |  |
|  |  | flanged male |  | 0 |  | 0.000 |  | 0.000 |  | 100.000 |  |
|  |  | infant |  | 0 |  | 0.000 |  | 0.000 |  | 100.000 |  |
|  |  | unflanged male |  | 0 |  | 0.000 |  | 0.000 |  | 100.000 |  |
|  |  | Missing |  | 0 |  | 0.000 |  |  |  |  |  |
|  |  | Total |  | 104 |  | 100.000 |  |  |  |  |  |
| Mindi |  | adolescent |  | 0 |  | 0.000 |  | 0.000 |  | 0.000 |  |
|  |  | female with infant |  | 17 |  | 100.000 |  | 100.000 |  | 100.000 |  |
|  |  | flanged male |  | 0 |  | 0.000 |  | 0.000 |  | 100.000 |  |
|  |  | infant |  | 0 |  | 0.000 |  | 0.000 |  | 100.000 |  |
|  |  | unflanged male |  | 0 |  | 0.000 |  | 0.000 |  | 100.000 |  |
|  |  | Missing |  | 0 |  | 0.000 |  |  |  |  |  |
|  |  | Total |  | 17 |  | 100.000 |  |  |  |  |  |
| Ompung |  | adolescent |  | 0 |  | 0.000 |  | 0.000 |  | 0.000 |  |
|  |  | female with infant |  | 0 |  | 0.000 |  | 0.000 |  | 0.000 |  |
|  |  | flanged male |  | 263 |  | 100.000 |  | 100.000 |  | 100.000 |  |
|  |  | infant |  | 0 |  | 0.000 |  | 0.000 |  | 100.000 |  |
|  |  | unflanged male |  | 0 |  | 0.000 |  | 0.000 |  | 100.000 |  |
|  |  | Missing |  | 0 |  | 0.000 |  |  |  |  |  |
|  |  | Total |  | 263 |  | 100.000 |  |  |  |  |  |
| Pensi |  | adolescent |  | 0 |  | 0.000 |  | 0.000 |  | 0.000 |  |
|  |  | female with infant |  | 15 |  | 100.000 |  | 100.000 |  | 100.000 |  |
|  |  | flanged male |  | 0 |  | 0.000 |  | 0.000 |  | 100.000 |  |
|  |  | infant |  | 0 |  | 0.000 |  | 0.000 |  | 100.000 |  |
|  |  | unflanged male |  | 0 |  | 0.000 |  | 0.000 |  | 100.000 |  |
|  |  | Missing |  | 0 |  | 0.000 |  |  |  |  |  |
|  |  | Total |  | 15 |  | 100.000 |  |  |  |  |  |
| Peot |  | adolescent |  | 8 |  | 100.000 |  | 100.000 |  | 100.000 |  |
|  |  | female with infant |  | 0 |  | 0.000 |  | 0.000 |  | 100.000 |  |
|  |  | flanged male |  | 0 |  | 0.000 |  | 0.000 |  | 100.000 |  |
|  |  | infant |  | 0 |  | 0.000 |  | 0.000 |  | 100.000 |  |
|  |  | unflanged male |  | 0 |  | 0.000 |  | 0.000 |  | 100.000 |  |
|  |  | Missing |  | 0 |  | 0.000 |  |  |  |  |  |
|  |  | Total |  | 8 |  | 100.000 |  |  |  |  |  |
| Prabu |  | adolescent |  | 0 |  | 0.000 |  | 0.000 |  | 0.000 |  |
|  |  | female with infant |  | 0 |  | 0.000 |  | 0.000 |  | 0.000 |  |
|  |  | flanged male |  | 80 |  | 100.000 |  | 100.000 |  | 100.000 |  |
|  |  | infant |  | 0 |  | 0.000 |  | 0.000 |  | 100.000 |  |
|  |  | unflanged male |  | 0 |  | 0.000 |  | 0.000 |  | 100.000 |  |
|  |  | Missing |  | 0 |  | 0.000 |  |  |  |  |  |
|  |  | Total |  | 80 |  | 100.000 |  |  |  |  |  |
| Raffi |  | adolescent |  | 0 |  | 0.000 |  | 0.000 |  | 0.000 |  |
|  |  | female with infant |  | 61 |  | 100.000 |  | 100.000 |  | 100.000 |  |
|  |  | flanged male |  | 0 |  | 0.000 |  | 0.000 |  | 100.000 |  |
|  |  | infant |  | 0 |  | 0.000 |  | 0.000 |  | 100.000 |  |
|  |  | unflanged male |  | 0 |  | 0.000 |  | 0.000 |  | 100.000 |  |
|  |  | Missing |  | 0 |  | 0.000 |  |  |  |  |  |
|  |  | Total |  | 61 |  | 100.000 |  |  |  |  |  |
| Rambo |  | adolescent |  | 0 |  | 0.000 |  | 0.000 |  | 0.000 |  |
|  |  | female with infant |  | 0 |  | 0.000 |  | 0.000 |  | 0.000 |  |
|  |  | flanged male |  | 29 |  | 100.000 |  | 100.000 |  | 100.000 |  |
|  |  | infant |  | 0 |  | 0.000 |  | 0.000 |  | 100.000 |  |
|  |  | unflanged male |  | 0 |  | 0.000 |  | 0.000 |  | 100.000 |  |
|  |  | Missing |  | 0 |  | 0.000 |  |  |  |  |  |
|  |  | Total |  | 29 |  | 100.000 |  |  |  |  |  |
| Ronaldo |  | adolescent |  | 0 |  | 0.000 |  | 0.000 |  | 0.000 |  |
|  |  | female with infant |  | 0 |  | 0.000 |  | 0.000 |  | 0.000 |  |
|  |  | flanged male |  | 0 |  | 0.000 |  | 0.000 |  | 0.000 |  |
|  |  | infant |  | 1 |  | 100.000 |  | 100.000 |  | 100.000 |  |
|  |  | unflanged male |  | 0 |  | 0.000 |  | 0.000 |  | 100.000 |  |
|  |  | Missing |  | 0 |  | 0.000 |  |  |  |  |  |
|  |  | Total |  | 1 |  | 100.000 |  |  |  |  |  |
| Salvador |  | adolescent |  | 0 |  | 0.000 |  | 0.000 |  | 0.000 |  |
|  |  | female with infant |  | 0 |  | 0.000 |  | 0.000 |  | 0.000 |  |
|  |  | flanged male |  | 46 |  | 100.000 |  | 100.000 |  | 100.000 |  |
|  |  | infant |  | 0 |  | 0.000 |  | 0.000 |  | 100.000 |  |
|  |  | unflanged male |  | 0 |  | 0.000 |  | 0.000 |  | 100.000 |  |
|  |  | Missing |  | 0 |  | 0.000 |  |  |  |  |  |
|  |  | Total |  | 46 |  | 100.000 |  |  |  |  |  |
| Suci |  | adolescent |  | 0 |  | 0.000 |  | 0.000 |  | 0.000 |  |
|  |  | female with infant |  | 5 |  | 100.000 |  | 100.000 |  | 100.000 |  |
|  |  | flanged male |  | 0 |  | 0.000 |  | 0.000 |  | 100.000 |  |
|  |  | infant |  | 0 |  | 0.000 |  | 0.000 |  | 100.000 |  |
|  |  | unflanged male |  | 0 |  | 0.000 |  | 0.000 |  | 100.000 |  |
|  |  | Missing |  | 0 |  | 0.000 |  |  |  |  |  |
|  |  | Total |  | 5 |  | 100.000 |  |  |  |  |  |
| Sultan |  | adolescent |  | 0 |  | 0.000 |  | 0.000 |  | 0.000 |  |
|  |  | female with infant |  | 0 |  | 0.000 |  | 0.000 |  | 0.000 |  |
|  |  | flanged male |  | 106 |  | 100.000 |  | 100.000 |  | 100.000 |  |
|  |  | infant |  | 0 |  | 0.000 |  | 0.000 |  | 100.000 |  |
|  |  | unflanged male |  | 0 |  | 0.000 |  | 0.000 |  | 100.000 |  |
|  |  | Missing |  | 0 |  | 0.000 |  |  |  |  |  |
|  |  | Total |  | 106 |  | 100.000 |  |  |  |  |  |
| Sumi |  | adolescent |  | 0 |  | 0.000 |  | 0.000 |  | 0.000 |  |
|  |  | female with infant |  | 32 |  | 100.000 |  | 100.000 |  | 100.000 |  |
|  |  | flanged male |  | 0 |  | 0.000 |  | 0.000 |  | 100.000 |  |
|  |  | infant |  | 0 |  | 0.000 |  | 0.000 |  | 100.000 |  |
|  |  | unflanged male |  | 0 |  | 0.000 |  | 0.000 |  | 100.000 |  |
|  |  | Missing |  | 0 |  | 0.000 |  |  |  |  |  |
|  |  | Total |  | 32 |  | 100.000 |  |  |  |  |  |
| Teju |  | adolescent |  | 0 |  | 0.000 |  | 0.000 |  | 0.000 |  |
|  |  | female with infant |  | 0 |  | 0.000 |  | 0.000 |  | 0.000 |  |
|  |  | flanged male |  | 22 |  | 100.000 |  | 100.000 |  | 100.000 |  |
|  |  | infant |  | 0 |  | 0.000 |  | 0.000 |  | 100.000 |  |
|  |  | unflanged male |  | 0 |  | 0.000 |  | 0.000 |  | 100.000 |  |
|  |  | Missing |  | 0 |  | 0.000 |  |  |  |  |  |
|  |  | Total |  | 22 |  | 100.000 |  |  |  |  |  |
| Teresia |  | adolescent |  | 0 |  | 0.000 |  | 0.000 |  | 0.000 |  |
|  |  | female with infant |  | 28 |  | 100.000 |  | 100.000 |  | 100.000 |  |
|  |  | flanged male |  | 0 |  | 0.000 |  | 0.000 |  | 100.000 |  |
|  |  | infant |  | 0 |  | 0.000 |  | 0.000 |  | 100.000 |  |
|  |  | unflanged male |  | 0 |  | 0.000 |  | 0.000 |  | 100.000 |  |
|  |  | Missing |  | 0 |  | 0.000 |  |  |  |  |  |
|  |  | Total |  | 28 |  | 100.000 |  |  |  |  |  |
| Timi |  | adolescent |  | 3 |  | 100.000 |  | 100.000 |  | 100.000 |  |
|  |  | female with infant |  | 0 |  | 0.000 |  | 0.000 |  | 100.000 |  |
|  |  | flanged male |  | 0 |  | 0.000 |  | 0.000 |  | 100.000 |  |
|  |  | infant |  | 0 |  | 0.000 |  | 0.000 |  | 100.000 |  |
|  |  | unflanged male |  | 0 |  | 0.000 |  | 0.000 |  | 100.000 |  |
|  |  | Missing |  | 0 |  | 0.000 |  |  |  |  |  |
|  |  | Total |  | 3 |  | 100.000 |  |  |  |  |  |
| Tina |  | adolescent |  | 316 |  | 100.000 |  | 100.000 |  | 100.000 |  |
|  |  | female with infant |  | 0 |  | 0.000 |  | 0.000 |  | 100.000 |  |
|  |  | flanged male |  | 0 |  | 0.000 |  | 0.000 |  | 100.000 |  |
|  |  | infant |  | 0 |  | 0.000 |  | 0.000 |  | 100.000 |  |
|  |  | unflanged male |  | 0 |  | 0.000 |  | 0.000 |  | 100.000 |  |
|  |  | Missing |  | 0 |  | 0.000 |  |  |  |  |  |
|  |  | Total |  | 316 |  | 100.000 |  |  |  |  |  |
| Travor |  | adolescent |  | 0 |  | 0.000 |  | 0.000 |  | 0.000 |  |
|  |  | female with infant |  | 0 |  | 0.000 |  | 0.000 |  | 0.000 |  |
|  |  | flanged male |  | 0 |  | 0.000 |  | 0.000 |  | 0.000 |  |
|  |  | infant |  | 1 |  | 100.000 |  | 100.000 |  | 100.000 |  |
|  |  | unflanged male |  | 0 |  | 0.000 |  | 0.000 |  | 100.000 |  |
|  |  | Missing |  | 0 |  | 0.000 |  |  |  |  |  |
|  |  | Total |  | 1 |  | 100.000 |  |  |  |  |  |
| Umi |  | adolescent |  | 0 |  | 0.000 |  | 0.000 |  | 0.000 |  |
|  |  | female with infant |  | 2 |  | 100.000 |  | 100.000 |  | 100.000 |  |
|  |  | flanged male |  | 0 |  | 0.000 |  | 0.000 |  | 100.000 |  |
|  |  | infant |  | 0 |  | 0.000 |  | 0.000 |  | 100.000 |  |
|  |  | unflanged male |  | 0 |  | 0.000 |  | 0.000 |  | 100.000 |  |
|  |  | Missing |  | 0 |  | 0.000 |  |  |  |  |  |
|  |  | Total |  | 2 |  | 100.000 |  |  |  |  |  |
| Unflm |  | adolescent |  | 0 |  | 0.000 |  | 0.000 |  | 0.000 |  |
|  |  | female with infant |  | 0 |  | 0.000 |  | 0.000 |  | 0.000 |  |
|  |  | flanged male |  | 0 |  | 0.000 |  | 0.000 |  | 0.000 |  |
|  |  | infant |  | 0 |  | 0.000 |  | 0.000 |  | 0.000 |  |
|  |  | unflanged male |  | 32 |  | 100.000 |  | 100.000 |  | 100.000 |  |
|  |  | Missing |  | 0 |  | 0.000 |  |  |  |  |  |
|  |  | Total |  | 32 |  | 100.000 |  |  |  |  |  |
| Uok |  | adolescent |  | 0 |  | 0.000 |  | 0.000 |  | 0.000 |  |
|  |  | female with infant |  | 0 |  | 0.000 |  | 0.000 |  | 0.000 |  |
|  |  | flanged male |  | 0 |  | 0.000 |  | 0.000 |  | 0.000 |  |
|  |  | infant |  | 49 |  | 100.000 |  | 100.000 |  | 100.000 |  |
|  |  | unflanged male |  | 0 |  | 0.000 |  | 0.000 |  | 100.000 |  |
|  |  | Missing |  | 0 |  | 0.000 |  |  |  |  |  |
|  |  | Total |  | 49 |  | 100.000 |  |  |  |  |  |
| Vulcan |  | adolescent |  | 0 |  | 0.000 |  | 0.000 |  | 0.000 |  |
|  |  | female with infant |  | 0 |  | 0.000 |  | 0.000 |  | 0.000 |  |
|  |  | flanged male |  | 2 |  | 100.000 |  | 100.000 |  | 100.000 |  |
|  |  | infant |  | 0 |  | 0.000 |  | 0.000 |  | 100.000 |  |
|  |  | unflanged male |  | 0 |  | 0.000 |  | 0.000 |  | 100.000 |  |
|  |  | Missing |  | 0 |  | 0.000 |  |  |  |  |  |
|  |  | Total |  | 2 |  | 100.000 |  |  |  |  |  |
| Walimah |  | adolescent |  | 100 |  | 100.000 |  | 100.000 |  | 100.000 |  |
|  |  | female with infant |  | 0 |  | 0.000 |  | 0.000 |  | 100.000 |  |
|  |  | flanged male |  | 0 |  | 0.000 |  | 0.000 |  | 100.000 |  |
|  |  | infant |  | 0 |  | 0.000 |  | 0.000 |  | 100.000 |  |
|  |  | unflanged male |  | 0 |  | 0.000 |  | 0.000 |  | 100.000 |  |
|  |  | Missing |  | 0 |  | 0.000 |  |  |  |  |  |
|  |  | Total |  | 100 |  | 100.000 |  |  |  |  |  |
| Wulan |  | adolescent |  | 69 |  | 100.000 |  | 100.000 |  | 100.000 |  |
|  |  | female with infant |  | 0 |  | 0.000 |  | 0.000 |  | 100.000 |  |
|  |  | flanged male |  | 0 |  | 0.000 |  | 0.000 |  | 100.000 |  |
|  |  | infant |  | 0 |  | 0.000 |  | 0.000 |  | 100.000 |  |
|  |  | unflanged male |  | 0 |  | 0.000 |  | 0.000 |  | 100.000 |  |
|  |  | Missing |  | 0 |  | 0.000 |  |  |  |  |  |
|  |  | Total |  | 69 |  | 100.000 |  |  |  |  |  |
| XL |  | adolescent |  | 0 |  | 0.000 |  | 0.000 |  | 0.000 |  |
|  |  | female with infant |  | 0 |  | 0.000 |  | 0.000 |  | 0.000 |  |
|  |  | flanged male |  | 41 |  | 100.000 |  | 100.000 |  | 100.000 |  |
|  |  | infant |  | 0 |  | 0.000 |  | 0.000 |  | 100.000 |  |
|  |  | unflanged male |  | 0 |  | 0.000 |  | 0.000 |  | 100.000 |  |
|  |  | Missing |  | 0 |  | 0.000 |  |  |  |  |  |
|  |  | Total |  | 41 |  | 100.000 |  |  |  |  |  |
| Xenix |  | adolescent |  | 0 |  | 0.000 |  | 0.000 |  | 0.000 |  |
|  |  | female with infant |  | 0 |  | 0.000 |  | 0.000 |  | 0.000 |  |
|  |  | flanged male |  | 8 |  | 100.000 |  | 100.000 |  | 100.000 |  |
|  |  | infant |  | 0 |  | 0.000 |  | 0.000 |  | 100.000 |  |
|  |  | unflanged male |  | 0 |  | 0.000 |  | 0.000 |  | 100.000 |  |
|  |  | Missing |  | 0 |  | 0.000 |  |  |  |  |  |
|  |  | Total |  | 8 |  | 100.000 |  |  |  |  |  |
| Yanti |  | adolescent |  | 0 |  | 0.000 |  | 0.000 |  | 0.000 |  |
|  |  | female with infant |  | 414 |  | 100.000 |  | 100.000 |  | 100.000 |  |
|  |  | flanged male |  | 0 |  | 0.000 |  | 0.000 |  | 100.000 |  |
|  |  | infant |  | 0 |  | 0.000 |  | 0.000 |  | 100.000 |  |
|  |  | unflanged male |  | 0 |  | 0.000 |  | 0.000 |  | 100.000 |  |
|  |  | Missing |  | 0 |  | 0.000 |  |  |  |  |  |
|  |  | Total |  | 414 |  | 100.000 |  |  |  |  |  |
| Zeus |  | adolescent |  | 0 |  | 0.000 |  | 0.000 |  | 0.000 |  |
|  |  | female with infant |  | 0 |  | 0.000 |  | 0.000 |  | 0.000 |  |
|  |  | flanged male |  | 1 |  | 100.000 |  | 100.000 |  | 100.000 |  |
|  |  | infant |  | 0 |  | 0.000 |  | 0.000 |  | 100.000 |  |
|  |  | unflanged male |  | 0 |  | 0.000 |  | 0.000 |  | 100.000 |  |
|  |  | Missing |  | 0 |  | 0.000 |  |  |  |  |  |
|  |  | Total |  | 1 |  | 100.000 |  |  |  |  |  |
| Zorro |  | adolescent |  | 0 |  | 0.000 |  | 0.000 |  | 0.000 |  |
|  |  | female with infant |  | 0 |  | 0.000 |  | 0.000 |  | 0.000 |  |
|  |  | flanged male |  | 0 |  | 0.000 |  | 0.000 |  | 0.000 |  |
|  |  | infant |  | 0 |  | 0.000 |  | 0.000 |  | 0.000 |  |
|  |  | unflanged male |  | 11 |  | 100.000 |  | 100.000 |  | 100.000 |  |
|  |  | Missing |  | 0 |  | 0.000 |  |  |  |  |  |
|  |  | Total |  | 11 |  | 100.000 |  |  |  |  |  |
|  | | | | | | | | | | | |

| Frequencies for context | | | | | | | | | | | |
| --- | --- | --- | --- | --- | --- | --- | --- | --- | --- | --- | --- |
| individual | | context | | Frequency | | Percent | | Valid Percent | | Cumulative Percent | |
| Alice |  | no apparent danger |  | 0 |  | 0.000 |  | 0.000 |  | 0.000 |  |
|  |  | towards animals |  | 0 |  | 0.000 |  | 0.000 |  | 0.000 |  |
|  |  | towards humans (non-observers) |  | 0 |  | 0.000 |  | 0.000 |  | 0.000 |  |
|  |  | towards observers |  | 3 |  | 15.000 |  | 15.000 |  | 15.000 |  |
|  |  | towards other orangutans |  | 17 |  | 85.000 |  | 85.000 |  | 100.000 |  |
|  |  | Missing |  | 0 |  | 0.000 |  |  |  |  |  |
|  |  | Total |  | 20 |  | 100.000 |  |  |  |  |  |
| Aminah |  | no apparent danger |  | 0 |  | 0.000 |  | 0.000 |  | 0.000 |  |
|  |  | towards animals |  | 0 |  | 0.000 |  | 0.000 |  | 0.000 |  |
|  |  | towards humans (non-observers) |  | 0 |  | 0.000 |  | 0.000 |  | 0.000 |  |
|  |  | towards observers |  | 26 |  | 100.000 |  | 100.000 |  | 100.000 |  |
|  |  | towards other orangutans |  | 0 |  | 0.000 |  | 0.000 |  | 100.000 |  |
|  |  | Missing |  | 0 |  | 0.000 |  |  |  |  |  |
|  |  | Total |  | 26 |  | 100.000 |  |  |  |  |  |
| Anto |  | no apparent danger |  | 0 |  | 0.000 |  | 0.000 |  | 0.000 |  |
|  |  | towards animals |  | 0 |  | 0.000 |  | 0.000 |  | 0.000 |  |
|  |  | towards humans (non-observers) |  | 0 |  | 0.000 |  | 0.000 |  | 0.000 |  |
|  |  | towards observers |  | 5 |  | 100.000 |  | 100.000 |  | 100.000 |  |
|  |  | towards other orangutans |  | 0 |  | 0.000 |  | 0.000 |  | 100.000 |  |
|  |  | Missing |  | 0 |  | 0.000 |  |  |  |  |  |
|  |  | Total |  | 5 |  | 100.000 |  |  |  |  |  |
| Asny |  | no apparent danger |  | 0 |  | 0.000 |  | 0.000 |  | 0.000 |  |
|  |  | towards animals |  | 0 |  | 0.000 |  | 0.000 |  | 0.000 |  |
|  |  | towards humans (non-observers) |  | 0 |  | 0.000 |  | 0.000 |  | 0.000 |  |
|  |  | towards observers |  | 15 |  | 100.000 |  | 100.000 |  | 100.000 |  |
|  |  | towards other orangutans |  | 0 |  | 0.000 |  | 0.000 |  | 100.000 |  |
|  |  | Missing |  | 0 |  | 0.000 |  |  |  |  |  |
|  |  | Total |  | 15 |  | 100.000 |  |  |  |  |  |
| Bagong |  | no apparent danger |  | 0 |  | 0.000 |  | 0.000 |  | 0.000 |  |
|  |  | towards animals |  | 0 |  | 0.000 |  | 0.000 |  | 0.000 |  |
|  |  | towards humans (non-observers) |  | 0 |  | 0.000 |  | 0.000 |  | 0.000 |  |
|  |  | towards observers |  | 101 |  | 100.000 |  | 100.000 |  | 100.000 |  |
|  |  | towards other orangutans |  | 0 |  | 0.000 |  | 0.000 |  | 100.000 |  |
|  |  | Missing |  | 0 |  | 0.000 |  |  |  |  |  |
|  |  | Total |  | 101 |  | 100.000 |  |  |  |  |  |
| Bendot |  | no apparent danger |  | 0 |  | 0.000 |  | 0.000 |  | 0.000 |  |
|  |  | towards animals |  | 0 |  | 0.000 |  | 0.000 |  | 0.000 |  |
|  |  | towards humans (non-observers) |  | 0 |  | 0.000 |  | 0.000 |  | 0.000 |  |
|  |  | towards observers |  | 65 |  | 100.000 |  | 100.000 |  | 100.000 |  |
|  |  | towards other orangutans |  | 0 |  | 0.000 |  | 0.000 |  | 100.000 |  |
|  |  | Missing |  | 0 |  | 0.000 |  |  |  |  |  |
|  |  | Total |  | 65 |  | 100.000 |  |  |  |  |  |
| Berani |  | no apparent danger |  | 0 |  | 0.000 |  | 0.000 |  | 0.000 |  |
|  |  | towards animals |  | 0 |  | 0.000 |  | 0.000 |  | 0.000 |  |
|  |  | towards humans (non-observers) |  | 0 |  | 0.000 |  | 0.000 |  | 0.000 |  |
|  |  | towards observers |  | 51 |  | 100.000 |  | 100.000 |  | 100.000 |  |
|  |  | towards other orangutans |  | 0 |  | 0.000 |  | 0.000 |  | 100.000 |  |
|  |  | Missing |  | 0 |  | 0.000 |  |  |  |  |  |
|  |  | Total |  | 51 |  | 100.000 |  |  |  |  |  |
| Beth |  | no apparent danger |  | 24 |  | 52.174 |  | 52.174 |  | 52.174 |  |
|  |  | towards animals |  | 0 |  | 0.000 |  | 0.000 |  | 52.174 |  |
|  |  | towards humans (non-observers) |  | 0 |  | 0.000 |  | 0.000 |  | 52.174 |  |
|  |  | towards observers |  | 22 |  | 47.826 |  | 47.826 |  | 100.000 |  |
|  |  | towards other orangutans |  | 0 |  | 0.000 |  | 0.000 |  | 100.000 |  |
|  |  | Missing |  | 0 |  | 0.000 |  |  |  |  |  |
|  |  | Total |  | 46 |  | 100.000 |  |  |  |  |  |
| Bibi |  | no apparent danger |  | 0 |  | 0.000 |  | 0.000 |  | 0.000 |  |
|  |  | towards animals |  | 0 |  | 0.000 |  | 0.000 |  | 0.000 |  |
|  |  | towards humans (non-observers) |  | 0 |  | 0.000 |  | 0.000 |  | 0.000 |  |
|  |  | towards observers |  | 13 |  | 100.000 |  | 100.000 |  | 100.000 |  |
|  |  | towards other orangutans |  | 0 |  | 0.000 |  | 0.000 |  | 100.000 |  |
|  |  | Missing |  | 0 |  | 0.000 |  |  |  |  |  |
|  |  | Total |  | 13 |  | 100.000 |  |  |  |  |  |
| Bintang |  | no apparent danger |  | 0 |  | 0.000 |  | 0.000 |  | 0.000 |  |
|  |  | towards animals |  | 0 |  | 0.000 |  | 0.000 |  | 0.000 |  |
|  |  | towards humans (non-observers) |  | 0 |  | 0.000 |  | 0.000 |  | 0.000 |  |
|  |  | towards observers |  | 93 |  | 100.000 |  | 100.000 |  | 100.000 |  |
|  |  | towards other orangutans |  | 0 |  | 0.000 |  | 0.000 |  | 100.000 |  |
|  |  | Missing |  | 0 |  | 0.000 |  |  |  |  |  |
|  |  | Total |  | 93 |  | 100.000 |  |  |  |  |  |
| Brutus |  | no apparent danger |  | 0 |  | 0.000 |  | 0.000 |  | 0.000 |  |
|  |  | towards animals |  | 6 |  | 3.279 |  | 3.279 |  | 3.279 |  |
|  |  | towards humans (non-observers) |  | 0 |  | 0.000 |  | 0.000 |  | 3.279 |  |
|  |  | towards observers |  | 177 |  | 96.721 |  | 96.721 |  | 100.000 |  |
|  |  | towards other orangutans |  | 0 |  | 0.000 |  | 0.000 |  | 100.000 |  |
|  |  | Missing |  | 0 |  | 0.000 |  |  |  |  |  |
|  |  | Total |  | 183 |  | 100.000 |  |  |  |  |  |
| Chindy |  | no apparent danger |  | 0 |  | 0.000 |  | 0.000 |  | 0.000 |  |
|  |  | towards animals |  | 9 |  | 40.909 |  | 40.909 |  | 40.909 |  |
|  |  | towards humans (non-observers) |  | 0 |  | 0.000 |  | 0.000 |  | 40.909 |  |
|  |  | towards observers |  | 13 |  | 59.091 |  | 59.091 |  | 100.000 |  |
|  |  | towards other orangutans |  | 0 |  | 0.000 |  | 0.000 |  | 100.000 |  |
|  |  | Missing |  | 0 |  | 0.000 |  |  |  |  |  |
|  |  | Total |  | 22 |  | 100.000 |  |  |  |  |  |
| Codet |  | no apparent danger |  | 0 |  | 0.000 |  | 0.000 |  | 0.000 |  |
|  |  | towards animals |  | 0 |  | 0.000 |  | 0.000 |  | 0.000 |  |
|  |  | towards humans (non-observers) |  | 0 |  | 0.000 |  | 0.000 |  | 0.000 |  |
|  |  | towards observers |  | 32 |  | 100.000 |  | 100.000 |  | 100.000 |  |
|  |  | towards other orangutans |  | 0 |  | 0.000 |  | 0.000 |  | 100.000 |  |
|  |  | Missing |  | 0 |  | 0.000 |  |  |  |  |  |
|  |  | Total |  | 32 |  | 100.000 |  |  |  |  |  |
| Elly |  | no apparent danger |  | 0 |  | 0.000 |  | 0.000 |  | 0.000 |  |
|  |  | towards animals |  | 1 |  | 1.961 |  | 1.961 |  | 1.961 |  |
|  |  | towards humans (non-observers) |  | 0 |  | 0.000 |  | 0.000 |  | 1.961 |  |
|  |  | towards observers |  | 46 |  | 90.196 |  | 90.196 |  | 92.157 |  |
|  |  | towards other orangutans |  | 4 |  | 7.843 |  | 7.843 |  | 100.000 |  |
|  |  | Missing |  | 0 |  | 0.000 |  |  |  |  |  |
|  |  | Total |  | 51 |  | 100.000 |  |  |  |  |  |
| Fajar |  | no apparent danger |  | 0 |  | 0.000 |  | 0.000 |  | 0.000 |  |
|  |  | towards animals |  | 0 |  | 0.000 |  | 0.000 |  | 0.000 |  |
|  |  | towards humans (non-observers) |  | 0 |  | 0.000 |  | 0.000 |  | 0.000 |  |
|  |  | towards observers |  | 246 |  | 100.000 |  | 100.000 |  | 100.000 |  |
|  |  | towards other orangutans |  | 0 |  | 0.000 |  | 0.000 |  | 100.000 |  |
|  |  | Missing |  | 0 |  | 0.000 |  |  |  |  |  |
|  |  | Total |  | 246 |  | 100.000 |  |  |  |  |  |
| Feb |  | no apparent danger |  | 0 |  | 0.000 |  | 0.000 |  | 0.000 |  |
|  |  | towards animals |  | 0 |  | 0.000 |  | 0.000 |  | 0.000 |  |
|  |  | towards humans (non-observers) |  | 0 |  | 0.000 |  | 0.000 |  | 0.000 |  |
|  |  | towards observers |  | 1 |  | 25.000 |  | 25.000 |  | 25.000 |  |
|  |  | towards other orangutans |  | 3 |  | 75.000 |  | 75.000 |  | 100.000 |  |
|  |  | Missing |  | 0 |  | 0.000 |  |  |  |  |  |
|  |  | Total |  | 4 |  | 100.000 |  |  |  |  |  |
| Female |  | no apparent danger |  | 0 |  | 0.000 |  | 0.000 |  | 0.000 |  |
|  |  | towards animals |  | 0 |  | 0.000 |  | 0.000 |  | 0.000 |  |
|  |  | towards humans (non-observers) |  | 0 |  | 0.000 |  | 0.000 |  | 0.000 |  |
|  |  | towards observers |  | 16 |  | 84.211 |  | 84.211 |  | 84.211 |  |
|  |  | towards other orangutans |  | 3 |  | 15.789 |  | 15.789 |  | 100.000 |  |
|  |  | Missing |  | 0 |  | 0.000 |  |  |  |  |  |
|  |  | Total |  | 19 |  | 100.000 |  |  |  |  |  |
| Fio |  | no apparent danger |  | 0 |  | 0.000 |  | 0.000 |  | 0.000 |  |
|  |  | towards animals |  | 1 |  | 11.111 |  | 11.111 |  | 11.111 |  |
|  |  | towards humans (non-observers) |  | 0 |  | 0.000 |  | 0.000 |  | 11.111 |  |
|  |  | towards observers |  | 5 |  | 55.556 |  | 55.556 |  | 66.667 |  |
|  |  | towards other orangutans |  | 3 |  | 33.333 |  | 33.333 |  | 100.000 |  |
|  |  | Missing |  | 0 |  | 0.000 |  |  |  |  |  |
|  |  | Total |  | 9 |  | 100.000 |  |  |  |  |  |
| Flanged male |  | no apparent danger |  | 0 |  | 0.000 |  | 0.000 |  | 0.000 |  |
|  |  | towards animals |  | 0 |  | 0.000 |  | 0.000 |  | 0.000 |  |
|  |  | towards humans (non-observers) |  | 0 |  | 0.000 |  | 0.000 |  | 0.000 |  |
|  |  | towards observers |  | 6 |  | 100.000 |  | 100.000 |  | 100.000 |  |
|  |  | towards other orangutans |  | 0 |  | 0.000 |  | 0.000 |  | 100.000 |  |
|  |  | Missing |  | 0 |  | 0.000 |  |  |  |  |  |
|  |  | Total |  | 6 |  | 100.000 |  |  |  |  |  |
| Freddy |  | no apparent danger |  | 0 |  | 0.000 |  | 0.000 |  | 0.000 |  |
|  |  | towards animals |  | 0 |  | 0.000 |  | 0.000 |  | 0.000 |  |
|  |  | towards humans (non-observers) |  | 0 |  | 0.000 |  | 0.000 |  | 0.000 |  |
|  |  | towards observers |  | 1 |  | 100.000 |  | 100.000 |  | 100.000 |  |
|  |  | towards other orangutans |  | 0 |  | 0.000 |  | 0.000 |  | 100.000 |  |
|  |  | Missing |  | 0 |  | 0.000 |  |  |  |  |  |
|  |  | Total |  | 1 |  | 100.000 |  |  |  |  |  |
| Friska |  | no apparent danger |  | 0 |  | 0.000 |  | 0.000 |  | 0.000 |  |
|  |  | towards animals |  | 0 |  | 0.000 |  | 0.000 |  | 0.000 |  |
|  |  | towards humans (non-observers) |  | 0 |  | 0.000 |  | 0.000 |  | 0.000 |  |
|  |  | towards observers |  | 0 |  | 0.000 |  | 0.000 |  | 0.000 |  |
|  |  | towards other orangutans |  | 1 |  | 100.000 |  | 100.000 |  | 100.000 |  |
|  |  | Missing |  | 0 |  | 0.000 |  |  |  |  |  |
|  |  | Total |  | 1 |  | 100.000 |  |  |  |  |  |
| Fugit |  | no apparent danger |  | 0 |  | 0.000 |  | 0.000 |  | 0.000 |  |
|  |  | towards animals |  | 0 |  | 0.000 |  | 0.000 |  | 0.000 |  |
|  |  | towards humans (non-observers) |  | 0 |  | 0.000 |  | 0.000 |  | 0.000 |  |
|  |  | towards observers |  | 0 |  | 0.000 |  | 0.000 |  | 0.000 |  |
|  |  | towards other orangutans |  | 126 |  | 100.000 |  | 100.000 |  | 100.000 |  |
|  |  | Missing |  | 0 |  | 0.000 |  |  |  |  |  |
|  |  | Total |  | 126 |  | 100.000 |  |  |  |  |  |
| Gangstah |  | no apparent danger |  | 0 |  | 0.000 |  | 0.000 |  | 0.000 |  |
|  |  | towards animals |  | 0 |  | 0.000 |  | 0.000 |  | 0.000 |  |
|  |  | towards humans (non-observers) |  | 0 |  | 0.000 |  | 0.000 |  | 0.000 |  |
|  |  | towards observers |  | 17 |  | 100.000 |  | 100.000 |  | 100.000 |  |
|  |  | towards other orangutans |  | 0 |  | 0.000 |  | 0.000 |  | 100.000 |  |
|  |  | Missing |  | 0 |  | 0.000 |  |  |  |  |  |
|  |  | Total |  | 17 |  | 100.000 |  |  |  |  |  |
| Gordon |  | no apparent danger |  | 0 |  | 0.000 |  | 0.000 |  | 0.000 |  |
|  |  | towards animals |  | 0 |  | 0.000 |  | 0.000 |  | 0.000 |  |
|  |  | towards humans (non-observers) |  | 0 |  | 0.000 |  | 0.000 |  | 0.000 |  |
|  |  | towards observers |  | 52 |  | 100.000 |  | 100.000 |  | 100.000 |  |
|  |  | towards other orangutans |  | 0 |  | 0.000 |  | 0.000 |  | 100.000 |  |
|  |  | Missing |  | 0 |  | 0.000 |  |  |  |  |  |
|  |  | Total |  | 52 |  | 100.000 |  |  |  |  |  |
| Gracia |  | no apparent danger |  | 0 |  | 0.000 |  | 0.000 |  | 0.000 |  |
|  |  | towards animals |  | 1 |  | 1.852 |  | 1.852 |  | 1.852 |  |
|  |  | towards humans (non-observers) |  | 0 |  | 0.000 |  | 0.000 |  | 1.852 |  |
|  |  | towards observers |  | 36 |  | 66.667 |  | 66.667 |  | 68.519 |  |
|  |  | towards other orangutans |  | 17 |  | 31.481 |  | 31.481 |  | 100.000 |  |
|  |  | Missing |  | 0 |  | 0.000 |  |  |  |  |  |
|  |  | Total |  | 54 |  | 100.000 |  |  |  |  |  |
| Gretel |  | no apparent danger |  | 0 |  | 0.000 |  | 0.000 |  | 0.000 |  |
|  |  | towards animals |  | 0 |  | 0.000 |  | 0.000 |  | 0.000 |  |
|  |  | towards humans (non-observers) |  | 0 |  | 0.000 |  | 0.000 |  | 0.000 |  |
|  |  | towards observers |  | 33 |  | 91.667 |  | 91.667 |  | 91.667 |  |
|  |  | towards other orangutans |  | 3 |  | 8.333 |  | 8.333 |  | 100.000 |  |
|  |  | Missing |  | 0 |  | 0.000 |  |  |  |  |  |
|  |  | Total |  | 36 |  | 100.000 |  |  |  |  |  |
| Henk |  | no apparent danger |  | 0 |  | 0.000 |  | 0.000 |  | 0.000 |  |
|  |  | towards animals |  | 0 |  | 0.000 |  | 0.000 |  | 0.000 |  |
|  |  | towards humans (non-observers) |  | 0 |  | 0.000 |  | 0.000 |  | 0.000 |  |
|  |  | towards observers |  | 4 |  | 100.000 |  | 100.000 |  | 100.000 |  |
|  |  | towards other orangutans |  | 0 |  | 0.000 |  | 0.000 |  | 100.000 |  |
|  |  | Missing |  | 0 |  | 0.000 |  |  |  |  |  |
|  |  | Total |  | 4 |  | 100.000 |  |  |  |  |  |
| Icarus |  | no apparent danger |  | 0 |  | 0.000 |  | 0.000 |  | 0.000 |  |
|  |  | towards animals |  | 0 |  | 0.000 |  | 0.000 |  | 0.000 |  |
|  |  | towards humans (non-observers) |  | 0 |  | 0.000 |  | 0.000 |  | 0.000 |  |
|  |  | towards observers |  | 1 |  | 20.000 |  | 20.000 |  | 20.000 |  |
|  |  | towards other orangutans |  | 4 |  | 80.000 |  | 80.000 |  | 100.000 |  |
|  |  | Missing |  | 0 |  | 0.000 |  |  |  |  |  |
|  |  | Total |  | 5 |  | 100.000 |  |  |  |  |  |
| Imp |  | no apparent danger |  | 0 |  | 0.000 |  | 0.000 |  | 0.000 |  |
|  |  | towards animals |  | 0 |  | 0.000 |  | 0.000 |  | 0.000 |  |
|  |  | towards humans (non-observers) |  | 0 |  | 0.000 |  | 0.000 |  | 0.000 |  |
|  |  | towards observers |  | 49 |  | 100.000 |  | 100.000 |  | 100.000 |  |
|  |  | towards other orangutans |  | 0 |  | 0.000 |  | 0.000 |  | 100.000 |  |
|  |  | Missing |  | 0 |  | 0.000 |  |  |  |  |  |
|  |  | Total |  | 49 |  | 100.000 |  |  |  |  |  |
| Indah |  | no apparent danger |  | 0 |  | 0.000 |  | 0.000 |  | 0.000 |  |
|  |  | towards animals |  | 0 |  | 0.000 |  | 0.000 |  | 0.000 |  |
|  |  | towards humans (non-observers) |  | 0 |  | 0.000 |  | 0.000 |  | 0.000 |  |
|  |  | towards observers |  | 17 |  | 100.000 |  | 100.000 |  | 100.000 |  |
|  |  | towards other orangutans |  | 0 |  | 0.000 |  | 0.000 |  | 100.000 |  |
|  |  | Missing |  | 0 |  | 0.000 |  |  |  |  |  |
|  |  | Total |  | 17 |  | 100.000 |  |  |  |  |  |
| Indi |  | no apparent danger |  | 5 |  | 25.000 |  | 25.000 |  | 25.000 |  |
|  |  | towards animals |  | 0 |  | 0.000 |  | 0.000 |  | 25.000 |  |
|  |  | towards humans (non-observers) |  | 0 |  | 0.000 |  | 0.000 |  | 25.000 |  |
|  |  | towards observers |  | 15 |  | 75.000 |  | 75.000 |  | 100.000 |  |
|  |  | towards other orangutans |  | 0 |  | 0.000 |  | 0.000 |  | 100.000 |  |
|  |  | Missing |  | 0 |  | 0.000 |  |  |  |  |  |
|  |  | Total |  | 20 |  | 100.000 |  |  |  |  |  |
| Irma |  | no apparent danger |  | 0 |  | 0.000 |  | 0.000 |  | 0.000 |  |
|  |  | towards animals |  | 7 |  | 0.440 |  | 0.440 |  | 0.440 |  |
|  |  | towards humans (non-observers) |  | 22 |  | 1.384 |  | 1.384 |  | 1.824 |  |
|  |  | towards observers |  | 1561 |  | 98.176 |  | 98.176 |  | 100.000 |  |
|  |  | towards other orangutans |  | 0 |  | 0.000 |  | 0.000 |  | 100.000 |  |
|  |  | Missing |  | 0 |  | 0.000 |  |  |  |  |  |
|  |  | Total |  | 1590 |  | 100.000 |  |  |  |  |  |
| James |  | no apparent danger |  | 0 |  | 0.000 |  | 0.000 |  | 0.000 |  |
|  |  | towards animals |  | 0 |  | 0.000 |  | 0.000 |  | 0.000 |  |
|  |  | towards humans (non-observers) |  | 0 |  | 0.000 |  | 0.000 |  | 0.000 |  |
|  |  | towards observers |  | 7 |  | 100.000 |  | 100.000 |  | 100.000 |  |
|  |  | towards other orangutans |  | 0 |  | 0.000 |  | 0.000 |  | 100.000 |  |
|  |  | Missing |  | 0 |  | 0.000 |  |  |  |  |  |
|  |  | Total |  | 7 |  | 100.000 |  |  |  |  |  |
| Janda Tua |  | no apparent danger |  | 0 |  | 0.000 |  | 0.000 |  | 0.000 |  |
|  |  | towards animals |  | 0 |  | 0.000 |  | 0.000 |  | 0.000 |  |
|  |  | towards humans (non-observers) |  | 0 |  | 0.000 |  | 0.000 |  | 0.000 |  |
|  |  | towards observers |  | 0 |  | 0.000 |  | 0.000 |  | 0.000 |  |
|  |  | towards other orangutans |  | 3 |  | 100.000 |  | 100.000 |  | 100.000 |  |
|  |  | Missing |  | 0 |  | 0.000 |  |  |  |  |  |
|  |  | Total |  | 3 |  | 100.000 |  |  |  |  |  |
| Jinak |  | no apparent danger |  | 0 |  | 0.000 |  | 0.000 |  | 0.000 |  |
|  |  | towards animals |  | 0 |  | 0.000 |  | 0.000 |  | 0.000 |  |
|  |  | towards humans (non-observers) |  | 0 |  | 0.000 |  | 0.000 |  | 0.000 |  |
|  |  | towards observers |  | 41 |  | 100.000 |  | 100.000 |  | 100.000 |  |
|  |  | towards other orangutans |  | 0 |  | 0.000 |  | 0.000 |  | 100.000 |  |
|  |  | Missing |  | 0 |  | 0.000 |  |  |  |  |  |
|  |  | Total |  | 41 |  | 100.000 |  |  |  |  |  |
| Joy |  | no apparent danger |  | 0 |  | 0.000 |  | 0.000 |  | 0.000 |  |
|  |  | towards animals |  | 0 |  | 0.000 |  | 0.000 |  | 0.000 |  |
|  |  | towards humans (non-observers) |  | 0 |  | 0.000 |  | 0.000 |  | 0.000 |  |
|  |  | towards observers |  | 1 |  | 50.000 |  | 50.000 |  | 50.000 |  |
|  |  | towards other orangutans |  | 1 |  | 50.000 |  | 50.000 |  | 100.000 |  |
|  |  | Missing |  | 0 |  | 0.000 |  |  |  |  |  |
|  |  | Total |  | 2 |  | 100.000 |  |  |  |  |  |
| Juni |  | no apparent danger |  | 0 |  | 0.000 |  | 0.000 |  | 0.000 |  |
|  |  | towards animals |  | 4 |  | 100.000 |  | 100.000 |  | 100.000 |  |
|  |  | towards humans (non-observers) |  | 0 |  | 0.000 |  | 0.000 |  | 100.000 |  |
|  |  | towards observers |  | 0 |  | 0.000 |  | 0.000 |  | 100.000 |  |
|  |  | towards other orangutans |  | 0 |  | 0.000 |  | 0.000 |  | 100.000 |  |
|  |  | Missing |  | 0 |  | 0.000 |  |  |  |  |  |
|  |  | Total |  | 4 |  | 100.000 |  |  |  |  |  |
| Juno |  | no apparent danger |  | 0 |  | 0.000 |  | 0.000 |  | 0.000 |  |
|  |  | towards animals |  | 0 |  | 0.000 |  | 0.000 |  | 0.000 |  |
|  |  | towards humans (non-observers) |  | 0 |  | 0.000 |  | 0.000 |  | 0.000 |  |
|  |  | towards observers |  | 14 |  | 100.000 |  | 100.000 |  | 100.000 |  |
|  |  | towards other orangutans |  | 0 |  | 0.000 |  | 0.000 |  | 100.000 |  |
|  |  | Missing |  | 0 |  | 0.000 |  |  |  |  |  |
|  |  | Total |  | 14 |  | 100.000 |  |  |  |  |  |
| Kacil |  | no apparent danger |  | 0 |  | 0.000 |  | 0.000 |  | 0.000 |  |
|  |  | towards animals |  | 0 |  | 0.000 |  | 0.000 |  | 0.000 |  |
|  |  | towards humans (non-observers) |  | 0 |  | 0.000 |  | 0.000 |  | 0.000 |  |
|  |  | towards observers |  | 5 |  | 100.000 |  | 100.000 |  | 100.000 |  |
|  |  | towards other orangutans |  | 0 |  | 0.000 |  | 0.000 |  | 100.000 |  |
|  |  | Missing |  | 0 |  | 0.000 |  |  |  |  |  |
|  |  | Total |  | 5 |  | 100.000 |  |  |  |  |  |
| Kan |  | no apparent danger |  | 0 |  | 0.000 |  | 0.000 |  | 0.000 |  |
|  |  | towards animals |  | 0 |  | 0.000 |  | 0.000 |  | 0.000 |  |
|  |  | towards humans (non-observers) |  | 0 |  | 0.000 |  | 0.000 |  | 0.000 |  |
|  |  | towards observers |  | 2 |  | 100.000 |  | 100.000 |  | 100.000 |  |
|  |  | towards other orangutans |  | 0 |  | 0.000 |  | 0.000 |  | 100.000 |  |
|  |  | Missing |  | 0 |  | 0.000 |  |  |  |  |  |
|  |  | Total |  | 2 |  | 100.000 |  |  |  |  |  |
| Kasi |  | no apparent danger |  | 0 |  | 0.000 |  | 0.000 |  | 0.000 |  |
|  |  | towards animals |  | 0 |  | 0.000 |  | 0.000 |  | 0.000 |  |
|  |  | towards humans (non-observers) |  | 0 |  | 0.000 |  | 0.000 |  | 0.000 |  |
|  |  | towards observers |  | 159 |  | 100.000 |  | 100.000 |  | 100.000 |  |
|  |  | towards other orangutans |  | 0 |  | 0.000 |  | 0.000 |  | 100.000 |  |
|  |  | Missing |  | 0 |  | 0.000 |  |  |  |  |  |
|  |  | Total |  | 159 |  | 100.000 |  |  |  |  |  |
| Kay |  | no apparent danger |  | 0 |  | 0.000 |  | 0.000 |  | 0.000 |  |
|  |  | towards animals |  | 0 |  | 0.000 |  | 0.000 |  | 0.000 |  |
|  |  | towards humans (non-observers) |  | 0 |  | 0.000 |  | 0.000 |  | 0.000 |  |
|  |  | towards observers |  | 107 |  | 96.396 |  | 100.000 |  | 100.000 |  |
|  |  | towards other orangutans |  | 0 |  | 0.000 |  | 0.000 |  | 100.000 |  |
|  |  | Missing |  | 4 |  | 3.604 |  |  |  |  |  |
|  |  | Total |  | 111 |  | 100.000 |  |  |  |  |  |
| Keri |  | no apparent danger |  | 0 |  | 0.000 |  | 0.000 |  | 0.000 |  |
|  |  | towards animals |  | 0 |  | 0.000 |  | 0.000 |  | 0.000 |  |
|  |  | towards humans (non-observers) |  | 0 |  | 0.000 |  | 0.000 |  | 0.000 |  |
|  |  | towards observers |  | 6 |  | 100.000 |  | 100.000 |  | 100.000 |  |
|  |  | towards other orangutans |  | 0 |  | 0.000 |  | 0.000 |  | 100.000 |  |
|  |  | Missing |  | 0 |  | 0.000 |  |  |  |  |  |
|  |  | Total |  | 6 |  | 100.000 |  |  |  |  |  |
| Keto |  | no apparent danger |  | 0 |  | 0.000 |  | 0.000 |  | 0.000 |  |
|  |  | towards animals |  | 0 |  | 0.000 |  | 0.000 |  | 0.000 |  |
|  |  | towards humans (non-observers) |  | 0 |  | 0.000 |  | 0.000 |  | 0.000 |  |
|  |  | towards observers |  | 1 |  | 100.000 |  | 100.000 |  | 100.000 |  |
|  |  | towards other orangutans |  | 0 |  | 0.000 |  | 0.000 |  | 100.000 |  |
|  |  | Missing |  | 0 |  | 0.000 |  |  |  |  |  |
|  |  | Total |  | 1 |  | 100.000 |  |  |  |  |  |
| Kondor |  | no apparent danger |  | 0 |  | 0.000 |  | 0.000 |  | 0.000 |  |
|  |  | towards animals |  | 0 |  | 0.000 |  | 0.000 |  | 0.000 |  |
|  |  | towards humans (non-observers) |  | 0 |  | 0.000 |  | 0.000 |  | 0.000 |  |
|  |  | towards observers |  | 38 |  | 100.000 |  | 100.000 |  | 100.000 |  |
|  |  | towards other orangutans |  | 0 |  | 0.000 |  | 0.000 |  | 100.000 |  |
|  |  | Missing |  | 0 |  | 0.000 |  |  |  |  |  |
|  |  | Total |  | 38 |  | 100.000 |  |  |  |  |  |
| Kundur |  | no apparent danger |  | 0 |  | 0.000 |  | 0.000 |  | 0.000 |  |
|  |  | towards animals |  | 0 |  | 0.000 |  | 0.000 |  | 0.000 |  |
|  |  | towards humans (non-observers) |  | 0 |  | 0.000 |  | 0.000 |  | 0.000 |  |
|  |  | towards observers |  | 42 |  | 100.000 |  | 100.000 |  | 100.000 |  |
|  |  | towards other orangutans |  | 0 |  | 0.000 |  | 0.000 |  | 100.000 |  |
|  |  | Missing |  | 0 |  | 0.000 |  |  |  |  |  |
|  |  | Total |  | 42 |  | 100.000 |  |  |  |  |  |
| Madalena |  | no apparent danger |  | 0 |  | 0.000 |  | 0.000 |  | 0.000 |  |
|  |  | towards animals |  | 15 |  | 100.000 |  | 100.000 |  | 100.000 |  |
|  |  | towards humans (non-observers) |  | 0 |  | 0.000 |  | 0.000 |  | 100.000 |  |
|  |  | towards observers |  | 0 |  | 0.000 |  | 0.000 |  | 100.000 |  |
|  |  | towards other orangutans |  | 0 |  | 0.000 |  | 0.000 |  | 100.000 |  |
|  |  | Missing |  | 0 |  | 0.000 |  |  |  |  |  |
|  |  | Total |  | 15 |  | 100.000 |  |  |  |  |  |
| Malé |  | no apparent danger |  | 0 |  | 0.000 |  | 0.000 |  | 0.000 |  |
|  |  | towards animals |  | 0 |  | 0.000 |  | 0.000 |  | 0.000 |  |
|  |  | towards humans (non-observers) |  | 0 |  | 0.000 |  | 0.000 |  | 0.000 |  |
|  |  | towards observers |  | 104 |  | 100.000 |  | 100.000 |  | 100.000 |  |
|  |  | towards other orangutans |  | 0 |  | 0.000 |  | 0.000 |  | 100.000 |  |
|  |  | Missing |  | 0 |  | 0.000 |  |  |  |  |  |
|  |  | Total |  | 104 |  | 100.000 |  |  |  |  |  |
| Mindi |  | no apparent danger |  | 0 |  | 0.000 |  | 0.000 |  | 0.000 |  |
|  |  | towards animals |  | 17 |  | 100.000 |  | 100.000 |  | 100.000 |  |
|  |  | towards humans (non-observers) |  | 0 |  | 0.000 |  | 0.000 |  | 100.000 |  |
|  |  | towards observers |  | 0 |  | 0.000 |  | 0.000 |  | 100.000 |  |
|  |  | towards other orangutans |  | 0 |  | 0.000 |  | 0.000 |  | 100.000 |  |
|  |  | Missing |  | 0 |  | 0.000 |  |  |  |  |  |
|  |  | Total |  | 17 |  | 100.000 |  |  |  |  |  |
| Ompung |  | no apparent danger |  | 0 |  | 0.000 |  | 0.000 |  | 0.000 |  |
|  |  | towards animals |  | 3 |  | 1.141 |  | 1.141 |  | 1.141 |  |
|  |  | towards humans (non-observers) |  | 0 |  | 0.000 |  | 0.000 |  | 1.141 |  |
|  |  | towards observers |  | 260 |  | 98.859 |  | 98.859 |  | 100.000 |  |
|  |  | towards other orangutans |  | 0 |  | 0.000 |  | 0.000 |  | 100.000 |  |
|  |  | Missing |  | 0 |  | 0.000 |  |  |  |  |  |
|  |  | Total |  | 263 |  | 100.000 |  |  |  |  |  |
| Pensi |  | no apparent danger |  | 0 |  | 0.000 |  | 0.000 |  | 0.000 |  |
|  |  | towards animals |  | 0 |  | 0.000 |  | 0.000 |  | 0.000 |  |
|  |  | towards humans (non-observers) |  | 0 |  | 0.000 |  | 0.000 |  | 0.000 |  |
|  |  | towards observers |  | 15 |  | 100.000 |  | 100.000 |  | 100.000 |  |
|  |  | towards other orangutans |  | 0 |  | 0.000 |  | 0.000 |  | 100.000 |  |
|  |  | Missing |  | 0 |  | 0.000 |  |  |  |  |  |
|  |  | Total |  | 15 |  | 100.000 |  |  |  |  |  |
| Peot |  | no apparent danger |  | 0 |  | 0.000 |  | 0.000 |  | 0.000 |  |
|  |  | towards animals |  | 0 |  | 0.000 |  | 0.000 |  | 0.000 |  |
|  |  | towards humans (non-observers) |  | 0 |  | 0.000 |  | 0.000 |  | 0.000 |  |
|  |  | towards observers |  | 8 |  | 100.000 |  | 100.000 |  | 100.000 |  |
|  |  | towards other orangutans |  | 0 |  | 0.000 |  | 0.000 |  | 100.000 |  |
|  |  | Missing |  | 0 |  | 0.000 |  |  |  |  |  |
|  |  | Total |  | 8 |  | 100.000 |  |  |  |  |  |
| Prabu |  | no apparent danger |  | 0 |  | 0.000 |  | 0.000 |  | 0.000 |  |
|  |  | towards animals |  | 0 |  | 0.000 |  | 0.000 |  | 0.000 |  |
|  |  | towards humans (non-observers) |  | 0 |  | 0.000 |  | 0.000 |  | 0.000 |  |
|  |  | towards observers |  | 80 |  | 100.000 |  | 100.000 |  | 100.000 |  |
|  |  | towards other orangutans |  | 0 |  | 0.000 |  | 0.000 |  | 100.000 |  |
|  |  | Missing |  | 0 |  | 0.000 |  |  |  |  |  |
|  |  | Total |  | 80 |  | 100.000 |  |  |  |  |  |
| Raffi |  | no apparent danger |  | 0 |  | 0.000 |  | 0.000 |  | 0.000 |  |
|  |  | towards animals |  | 0 |  | 0.000 |  | 0.000 |  | 0.000 |  |
|  |  | towards humans (non-observers) |  | 0 |  | 0.000 |  | 0.000 |  | 0.000 |  |
|  |  | towards observers |  | 61 |  | 100.000 |  | 100.000 |  | 100.000 |  |
|  |  | towards other orangutans |  | 0 |  | 0.000 |  | 0.000 |  | 100.000 |  |
|  |  | Missing |  | 0 |  | 0.000 |  |  |  |  |  |
|  |  | Total |  | 61 |  | 100.000 |  |  |  |  |  |
| Rambo |  | no apparent danger |  | 0 |  | 0.000 |  | 0.000 |  | 0.000 |  |
|  |  | towards animals |  | 0 |  | 0.000 |  | 0.000 |  | 0.000 |  |
|  |  | towards humans (non-observers) |  | 0 |  | 0.000 |  | 0.000 |  | 0.000 |  |
|  |  | towards observers |  | 29 |  | 100.000 |  | 100.000 |  | 100.000 |  |
|  |  | towards other orangutans |  | 0 |  | 0.000 |  | 0.000 |  | 100.000 |  |
|  |  | Missing |  | 0 |  | 0.000 |  |  |  |  |  |
|  |  | Total |  | 29 |  | 100.000 |  |  |  |  |  |
| Ronaldo |  | no apparent danger |  | 0 |  | 0.000 |  | 0.000 |  | 0.000 |  |
|  |  | towards animals |  | 0 |  | 0.000 |  | 0.000 |  | 0.000 |  |
|  |  | towards humans (non-observers) |  | 0 |  | 0.000 |  | 0.000 |  | 0.000 |  |
|  |  | towards observers |  | 1 |  | 100.000 |  | 100.000 |  | 100.000 |  |
|  |  | towards other orangutans |  | 0 |  | 0.000 |  | 0.000 |  | 100.000 |  |
|  |  | Missing |  | 0 |  | 0.000 |  |  |  |  |  |
|  |  | Total |  | 1 |  | 100.000 |  |  |  |  |  |
| Salvador |  | no apparent danger |  | 0 |  | 0.000 |  | 0.000 |  | 0.000 |  |
|  |  | towards animals |  | 1 |  | 2.174 |  | 2.174 |  | 2.174 |  |
|  |  | towards humans (non-observers) |  | 0 |  | 0.000 |  | 0.000 |  | 2.174 |  |
|  |  | towards observers |  | 34 |  | 73.913 |  | 73.913 |  | 76.087 |  |
|  |  | towards other orangutans |  | 11 |  | 23.913 |  | 23.913 |  | 100.000 |  |
|  |  | Missing |  | 0 |  | 0.000 |  |  |  |  |  |
|  |  | Total |  | 46 |  | 100.000 |  |  |  |  |  |
| Suci |  | no apparent danger |  | 0 |  | 0.000 |  | 0.000 |  | 0.000 |  |
|  |  | towards animals |  | 4 |  | 80.000 |  | 80.000 |  | 80.000 |  |
|  |  | towards humans (non-observers) |  | 0 |  | 0.000 |  | 0.000 |  | 80.000 |  |
|  |  | towards observers |  | 1 |  | 20.000 |  | 20.000 |  | 100.000 |  |
|  |  | towards other orangutans |  | 0 |  | 0.000 |  | 0.000 |  | 100.000 |  |
|  |  | Missing |  | 0 |  | 0.000 |  |  |  |  |  |
|  |  | Total |  | 5 |  | 100.000 |  |  |  |  |  |
| Sultan |  | no apparent danger |  | 0 |  | 0.000 |  | 0.000 |  | 0.000 |  |
|  |  | towards animals |  | 0 |  | 0.000 |  | 0.000 |  | 0.000 |  |
|  |  | towards humans (non-observers) |  | 0 |  | 0.000 |  | 0.000 |  | 0.000 |  |
|  |  | towards observers |  | 106 |  | 100.000 |  | 100.000 |  | 100.000 |  |
|  |  | towards other orangutans |  | 0 |  | 0.000 |  | 0.000 |  | 100.000 |  |
|  |  | Missing |  | 0 |  | 0.000 |  |  |  |  |  |
|  |  | Total |  | 106 |  | 100.000 |  |  |  |  |  |
| Sumi |  | no apparent danger |  | 0 |  | 0.000 |  | 0.000 |  | 0.000 |  |
|  |  | towards animals |  | 24 |  | 75.000 |  | 75.000 |  | 75.000 |  |
|  |  | towards humans (non-observers) |  | 0 |  | 0.000 |  | 0.000 |  | 75.000 |  |
|  |  | towards observers |  | 8 |  | 25.000 |  | 25.000 |  | 100.000 |  |
|  |  | towards other orangutans |  | 0 |  | 0.000 |  | 0.000 |  | 100.000 |  |
|  |  | Missing |  | 0 |  | 0.000 |  |  |  |  |  |
|  |  | Total |  | 32 |  | 100.000 |  |  |  |  |  |
| Teju |  | no apparent danger |  | 0 |  | 0.000 |  | 0.000 |  | 0.000 |  |
|  |  | towards animals |  | 0 |  | 0.000 |  | 0.000 |  | 0.000 |  |
|  |  | towards humans (non-observers) |  | 0 |  | 0.000 |  | 0.000 |  | 0.000 |  |
|  |  | towards observers |  | 22 |  | 100.000 |  | 100.000 |  | 100.000 |  |
|  |  | towards other orangutans |  | 0 |  | 0.000 |  | 0.000 |  | 100.000 |  |
|  |  | Missing |  | 0 |  | 0.000 |  |  |  |  |  |
|  |  | Total |  | 22 |  | 100.000 |  |  |  |  |  |
| Teresia |  | no apparent danger |  | 0 |  | 0.000 |  | 0.000 |  | 0.000 |  |
|  |  | towards animals |  | 4 |  | 14.286 |  | 14.286 |  | 14.286 |  |
|  |  | towards humans (non-observers) |  | 0 |  | 0.000 |  | 0.000 |  | 14.286 |  |
|  |  | towards observers |  | 15 |  | 53.571 |  | 53.571 |  | 67.857 |  |
|  |  | towards other orangutans |  | 9 |  | 32.143 |  | 32.143 |  | 100.000 |  |
|  |  | Missing |  | 0 |  | 0.000 |  |  |  |  |  |
|  |  | Total |  | 28 |  | 100.000 |  |  |  |  |  |
| Timi |  | no apparent danger |  | 0 |  | 0.000 |  | 0.000 |  | 0.000 |  |
|  |  | towards animals |  | 0 |  | 0.000 |  | 0.000 |  | 0.000 |  |
|  |  | towards humans (non-observers) |  | 0 |  | 0.000 |  | 0.000 |  | 0.000 |  |
|  |  | towards observers |  | 2 |  | 66.667 |  | 66.667 |  | 66.667 |  |
|  |  | towards other orangutans |  | 1 |  | 33.333 |  | 33.333 |  | 100.000 |  |
|  |  | Missing |  | 0 |  | 0.000 |  |  |  |  |  |
|  |  | Total |  | 3 |  | 100.000 |  |  |  |  |  |
| Tina |  | no apparent danger |  | 0 |  | 0.000 |  | 0.000 |  | 0.000 |  |
|  |  | towards animals |  | 0 |  | 0.000 |  | 0.000 |  | 0.000 |  |
|  |  | towards humans (non-observers) |  | 0 |  | 0.000 |  | 0.000 |  | 0.000 |  |
|  |  | towards observers |  | 316 |  | 100.000 |  | 100.000 |  | 100.000 |  |
|  |  | towards other orangutans |  | 0 |  | 0.000 |  | 0.000 |  | 100.000 |  |
|  |  | Missing |  | 0 |  | 0.000 |  |  |  |  |  |
|  |  | Total |  | 316 |  | 100.000 |  |  |  |  |  |
| Travor |  | no apparent danger |  | 0 |  | 0.000 |  | 0.000 |  | 0.000 |  |
|  |  | towards animals |  | 0 |  | 0.000 |  | 0.000 |  | 0.000 |  |
|  |  | towards humans (non-observers) |  | 0 |  | 0.000 |  | 0.000 |  | 0.000 |  |
|  |  | towards observers |  | 1 |  | 100.000 |  | 100.000 |  | 100.000 |  |
|  |  | towards other orangutans |  | 0 |  | 0.000 |  | 0.000 |  | 100.000 |  |
|  |  | Missing |  | 0 |  | 0.000 |  |  |  |  |  |
|  |  | Total |  | 1 |  | 100.000 |  |  |  |  |  |
| Umi |  | no apparent danger |  | 0 |  | 0.000 |  | 0.000 |  | 0.000 |  |
|  |  | towards animals |  | 0 |  | 0.000 |  | 0.000 |  | 0.000 |  |
|  |  | towards humans (non-observers) |  | 0 |  | 0.000 |  | 0.000 |  | 0.000 |  |
|  |  | towards observers |  | 2 |  | 100.000 |  | 100.000 |  | 100.000 |  |
|  |  | towards other orangutans |  | 0 |  | 0.000 |  | 0.000 |  | 100.000 |  |
|  |  | Missing |  | 0 |  | 0.000 |  |  |  |  |  |
|  |  | Total |  | 2 |  | 100.000 |  |  |  |  |  |
| Unflm |  | no apparent danger |  | 0 |  | 0.000 |  | 0.000 |  | 0.000 |  |
|  |  | towards animals |  | 0 |  | 0.000 |  | 0.000 |  | 0.000 |  |
|  |  | towards humans (non-observers) |  | 0 |  | 0.000 |  | 0.000 |  | 0.000 |  |
|  |  | towards observers |  | 22 |  | 68.750 |  | 68.750 |  | 68.750 |  |
|  |  | towards other orangutans |  | 10 |  | 31.250 |  | 31.250 |  | 100.000 |  |
|  |  | Missing |  | 0 |  | 0.000 |  |  |  |  |  |
|  |  | Total |  | 32 |  | 100.000 |  |  |  |  |  |
| Uok |  | no apparent danger |  | 2 |  | 4.082 |  | 4.082 |  | 4.082 |  |
|  |  | towards animals |  | 0 |  | 0.000 |  | 0.000 |  | 4.082 |  |
|  |  | towards humans (non-observers) |  | 0 |  | 0.000 |  | 0.000 |  | 4.082 |  |
|  |  | towards observers |  | 47 |  | 95.918 |  | 95.918 |  | 100.000 |  |
|  |  | towards other orangutans |  | 0 |  | 0.000 |  | 0.000 |  | 100.000 |  |
|  |  | Missing |  | 0 |  | 0.000 |  |  |  |  |  |
|  |  | Total |  | 49 |  | 100.000 |  |  |  |  |  |
| Vulcan |  | no apparent danger |  | 0 |  | 0.000 |  | 0.000 |  | 0.000 |  |
|  |  | towards animals |  | 0 |  | 0.000 |  | 0.000 |  | 0.000 |  |
|  |  | towards humans (non-observers) |  | 0 |  | 0.000 |  | 0.000 |  | 0.000 |  |
|  |  | towards observers |  | 2 |  | 100.000 |  | 100.000 |  | 100.000 |  |
|  |  | towards other orangutans |  | 0 |  | 0.000 |  | 0.000 |  | 100.000 |  |
|  |  | Missing |  | 0 |  | 0.000 |  |  |  |  |  |
|  |  | Total |  | 2 |  | 100.000 |  |  |  |  |  |
| Walimah |  | no apparent danger |  | 0 |  | 0.000 |  | 0.000 |  | 0.000 |  |
|  |  | towards animals |  | 0 |  | 0.000 |  | 0.000 |  | 0.000 |  |
|  |  | towards humans (non-observers) |  | 0 |  | 0.000 |  | 0.000 |  | 0.000 |  |
|  |  | towards observers |  | 100 |  | 100.000 |  | 100.000 |  | 100.000 |  |
|  |  | towards other orangutans |  | 0 |  | 0.000 |  | 0.000 |  | 100.000 |  |
|  |  | Missing |  | 0 |  | 0.000 |  |  |  |  |  |
|  |  | Total |  | 100 |  | 100.000 |  |  |  |  |  |
| Wulan |  | no apparent danger |  | 0 |  | 0.000 |  | 0.000 |  | 0.000 |  |
|  |  | towards animals |  | 0 |  | 0.000 |  | 0.000 |  | 0.000 |  |
|  |  | towards humans (non-observers) |  | 0 |  | 0.000 |  | 0.000 |  | 0.000 |  |
|  |  | towards observers |  | 69 |  | 100.000 |  | 100.000 |  | 100.000 |  |
|  |  | towards other orangutans |  | 0 |  | 0.000 |  | 0.000 |  | 100.000 |  |
|  |  | Missing |  | 0 |  | 0.000 |  |  |  |  |  |
|  |  | Total |  | 69 |  | 100.000 |  |  |  |  |  |
| XL |  | no apparent danger |  | 0 |  | 0.000 |  | 0.000 |  | 0.000 |  |
|  |  | towards animals |  | 0 |  | 0.000 |  | 0.000 |  | 0.000 |  |
|  |  | towards humans (non-observers) |  | 0 |  | 0.000 |  | 0.000 |  | 0.000 |  |
|  |  | towards observers |  | 41 |  | 100.000 |  | 100.000 |  | 100.000 |  |
|  |  | towards other orangutans |  | 0 |  | 0.000 |  | 0.000 |  | 100.000 |  |
|  |  | Missing |  | 0 |  | 0.000 |  |  |  |  |  |
|  |  | Total |  | 41 |  | 100.000 |  |  |  |  |  |
| Xenix |  | no apparent danger |  | 0 |  | 0.000 |  | 0.000 |  | 0.000 |  |
|  |  | towards animals |  | 0 |  | 0.000 |  | 0.000 |  | 0.000 |  |
|  |  | towards humans (non-observers) |  | 0 |  | 0.000 |  | 0.000 |  | 0.000 |  |
|  |  | towards observers |  | 8 |  | 100.000 |  | 100.000 |  | 100.000 |  |
|  |  | towards other orangutans |  | 0 |  | 0.000 |  | 0.000 |  | 100.000 |  |
|  |  | Missing |  | 0 |  | 0.000 |  |  |  |  |  |
|  |  | Total |  | 8 |  | 100.000 |  |  |  |  |  |
| Yanti |  | no apparent danger |  | 0 |  | 0.000 |  | 0.000 |  | 0.000 |  |
|  |  | towards animals |  | 0 |  | 0.000 |  | 0.000 |  | 0.000 |  |
|  |  | towards humans (non-observers) |  | 0 |  | 0.000 |  | 0.000 |  | 0.000 |  |
|  |  | towards observers |  | 414 |  | 100.000 |  | 100.000 |  | 100.000 |  |
|  |  | towards other orangutans |  | 0 |  | 0.000 |  | 0.000 |  | 100.000 |  |
|  |  | Missing |  | 0 |  | 0.000 |  |  |  |  |  |
|  |  | Total |  | 414 |  | 100.000 |  |  |  |  |  |
| Zeus |  | no apparent danger |  | 0 |  | 0.000 |  | 0.000 |  | 0.000 |  |
|  |  | towards animals |  | 0 |  | 0.000 |  | 0.000 |  | 0.000 |  |
|  |  | towards humans (non-observers) |  | 0 |  | 0.000 |  | 0.000 |  | 0.000 |  |
|  |  | towards observers |  | 0 |  | 0.000 |  | 0.000 |  | 0.000 |  |
|  |  | towards other orangutans |  | 1 |  | 100.000 |  | 100.000 |  | 100.000 |  |
|  |  | Missing |  | 0 |  | 0.000 |  |  |  |  |  |
|  |  | Total |  | 1 |  | 100.000 |  |  |  |  |  |
| Zorro |  | no apparent danger |  | 0 |  | 0.000 |  | 0.000 |  | 0.000 |  |
|  |  | towards animals |  | 0 |  | 0.000 |  | 0.000 |  | 0.000 |  |
|  |  | towards humans (non-observers) |  | 0 |  | 0.000 |  | 0.000 |  | 0.000 |  |
|  |  | towards observers |  | 11 |  | 100.000 |  | 100.000 |  | 100.000 |  |
|  |  | towards other orangutans |  | 0 |  | 0.000 |  | 0.000 |  | 100.000 |  |
|  |  | Missing |  | 0 |  | 0.000 |  |  |  |  |  |
|  |  | Total |  | 11 |  | 100.000 |  |  |  |  |  |
|  | | | | | | | | | | | |
