# Supplementary material for: Sociality predicts orangutan vocal phenotype
Source: Nat Ecol Evol. 2022 Mar 21;6(5):644–52. doi: 10.1038/s41559-022-01689-z (PMC9085614; doi:10.1038/s41559-022-01689-z)
Supplement: Supplementary file 4 — Descriptive statistics for entropic data before exclusions. [file 41559_2022_1689_MOESM4_ESM.html]

JASP 


# Results

## Grand Descriptive Statistics\_Dataset before exclusions

| Descriptive Statistics | | | | | | | | | | | | | | | | | | | | | |
| --- | --- | --- | --- | --- | --- | --- | --- | --- | --- | --- | --- | --- | --- | --- | --- | --- | --- | --- | --- | --- | --- |
|  | | E.frequency | | S.frequency | | C.frequency | | E.duration | | S.duration | | C.duration | | Sex | | AgeSex class | | Context | | Individual | |
| Valid |  | 106 |  | 106 |  | 106 |  | 106 |  | 106 |  | 106 |  | 106 |  | 106 |  | 105 |  | 106 |  |
| Missing |  | 0 |  | 0 |  | 0 |  | 0 |  | 0 |  | 0 |  | 0 |  | 0 |  | 1 |  | 0 |  |
| Mean |  | 0.536 |  | 0.464 |  | 0.645 |  | 0.572 |  | 0.428 |  | 0.588 |  |  |  |  |  |  |  |  |  |
| Std. Deviation |  | 0.297 |  | 0.297 |  | 0.342 |  | 0.314 |  | 0.314 |  | 0.334 |  |  |  |  |  |  |  |  |  |
| Minimum |  | 0.000 |  | 0.064 |  | 0.000 |  | 0.000 |  | 0.054 |  | 0.000 |  |  |  |  |  |  |  |  |  |
| Maximum |  | 0.936 |  | 1.000 |  | 0.998 |  | 0.946 |  | 1.000 |  | 1.000 |  |  |  |  |  |  |  |  |  |
|  | | | | | | | | | | | | | | | | | | | | | |
|  |  |  |  |  |  |  |  |  |  |  |  |  |  |  |  |  |  |  |  |  |  |
| --- | --- | --- | --- | --- | --- | --- | --- | --- | --- | --- | --- | --- | --- | --- | --- | --- | --- | --- | --- | --- | --- |
| *Note.*  Not all values are available for *Nominal Text* variables | | | | | | | | | | | | | | | | | | | | | |

### Frequency Tables

| Frequencies for Sex | | | | | | | | | |
| --- | --- | --- | --- | --- | --- | --- | --- | --- | --- |
| Sex | | Frequency | | Percent | | Valid Percent | | Cumulative Percent | |
| female |  | 61 |  | 57.547 |  | 57.547 |  | 57.547 |  |
| male |  | 45 |  | 42.453 |  | 42.453 |  | 100.000 |  |
| Missing |  | 0 |  | 0.000 |  |  |  |  |  |
| Total |  | 106 |  | 100.000 |  |  |  |  |  |
|  | | | | | | | | | |

| Frequencies for AgeSex class | | | | | | | | | |
| --- | --- | --- | --- | --- | --- | --- | --- | --- | --- |
| AgeSex class | | Frequency | | Percent | | Valid Percent | | Cumulative Percent | |
| adolescent |  | 19 |  | 17.925 |  | 17.925 |  | 17.925 |  |
| female with infant |  | 36 |  | 33.962 |  | 33.962 |  | 51.887 |  |
| flanged male |  | 22 |  | 20.755 |  | 20.755 |  | 72.642 |  |
| infant |  | 17 |  | 16.038 |  | 16.038 |  | 88.679 |  |
| unflanged male |  | 12 |  | 11.321 |  | 11.321 |  | 100.000 |  |
| Missing |  | 0 |  | 0.000 |  |  |  |  |  |
| Total |  | 106 |  | 100.000 |  |  |  |  |  |
|  | | | | | | | | | |

| Frequencies for Context | | | | | | | | | |
| --- | --- | --- | --- | --- | --- | --- | --- | --- | --- |
| Context | | Frequency | | Percent | | Valid Percent | | Cumulative Percent | |
| no apparent danger |  | 4 |  | 3.774 |  | 3.810 |  | 3.810 |  |
| towards animals |  | 14 |  | 13.208 |  | 13.333 |  | 17.143 |  |
| towards humans (non-observers) |  | 1 |  | 0.943 |  | 0.952 |  | 18.095 |  |
| towards observers |  | 69 |  | 65.094 |  | 65.714 |  | 83.810 |  |
| towards other orangutans |  | 17 |  | 16.038 |  | 16.190 |  | 100.000 |  |
| Missing |  | 1 |  | 0.943 |  |  |  |  |  |
| Total |  | 106 |  | 100.000 |  |  |  |  |  |
|  | | | | | | | | | |

| Frequencies for Individual | | | | | | | | | |
| --- | --- | --- | --- | --- | --- | --- | --- | --- | --- |
| Individual | | Frequency | | Percent | | Valid Percent | | Cumulative Percent | |
| Alice |  | 2 |  | 1.887 |  | 1.887 |  | 1.887 |  |
| Aminah |  | 1 |  | 0.943 |  | 0.943 |  | 2.830 |  |
| Anto |  | 1 |  | 0.943 |  | 0.943 |  | 3.774 |  |
| Asny |  | 1 |  | 0.943 |  | 0.943 |  | 4.717 |  |
| Bagong |  | 1 |  | 0.943 |  | 0.943 |  | 5.660 |  |
| Bendot |  | 1 |  | 0.943 |  | 0.943 |  | 6.604 |  |
| Berani |  | 1 |  | 0.943 |  | 0.943 |  | 7.547 |  |
| Beth |  | 2 |  | 1.887 |  | 1.887 |  | 9.434 |  |
| Bibi |  | 1 |  | 0.943 |  | 0.943 |  | 10.377 |  |
| Bintang |  | 1 |  | 0.943 |  | 0.943 |  | 11.321 |  |
| Brutus |  | 2 |  | 1.887 |  | 1.887 |  | 13.208 |  |
| Chindy |  | 2 |  | 1.887 |  | 1.887 |  | 15.094 |  |
| Codet |  | 1 |  | 0.943 |  | 0.943 |  | 16.038 |  |
| Elly |  | 3 |  | 2.830 |  | 2.830 |  | 18.868 |  |
| Fajar |  | 1 |  | 0.943 |  | 0.943 |  | 19.811 |  |
| Feb |  | 2 |  | 1.887 |  | 1.887 |  | 21.698 |  |
| Female |  | 2 |  | 1.887 |  | 1.887 |  | 23.585 |  |
| Fio |  | 3 |  | 2.830 |  | 2.830 |  | 26.415 |  |
| Flanged male |  | 1 |  | 0.943 |  | 0.943 |  | 27.358 |  |
| Freddy |  | 1 |  | 0.943 |  | 0.943 |  | 28.302 |  |
| Friska |  | 1 |  | 0.943 |  | 0.943 |  | 29.245 |  |
| Fugit |  | 1 |  | 0.943 |  | 0.943 |  | 30.189 |  |
| Gangstah |  | 1 |  | 0.943 |  | 0.943 |  | 31.132 |  |
| Gordon |  | 1 |  | 0.943 |  | 0.943 |  | 32.075 |  |
| Gracia |  | 3 |  | 2.830 |  | 2.830 |  | 34.906 |  |
| Gretel |  | 2 |  | 1.887 |  | 1.887 |  | 36.792 |  |
| Henk |  | 1 |  | 0.943 |  | 0.943 |  | 37.736 |  |
| Icarus |  | 2 |  | 1.887 |  | 1.887 |  | 39.623 |  |
| Imp |  | 1 |  | 0.943 |  | 0.943 |  | 40.566 |  |
| Indah |  | 1 |  | 0.943 |  | 0.943 |  | 41.509 |  |
| Indi |  | 2 |  | 1.887 |  | 1.887 |  | 43.396 |  |
| Irma |  | 3 |  | 2.830 |  | 2.830 |  | 46.226 |  |
| James |  | 1 |  | 0.943 |  | 0.943 |  | 47.170 |  |
| Janda Tua |  | 1 |  | 0.943 |  | 0.943 |  | 48.113 |  |
| Jinak |  | 1 |  | 0.943 |  | 0.943 |  | 49.057 |  |
| Joy |  | 2 |  | 1.887 |  | 1.887 |  | 50.943 |  |
| Juni |  | 1 |  | 0.943 |  | 0.943 |  | 51.887 |  |
| Juno |  | 1 |  | 0.943 |  | 0.943 |  | 52.830 |  |
| Kacil |  | 1 |  | 0.943 |  | 0.943 |  | 53.774 |  |
| Kan |  | 1 |  | 0.943 |  | 0.943 |  | 54.717 |  |
| Kasi |  | 1 |  | 0.943 |  | 0.943 |  | 55.660 |  |
| Kay |  | 2 |  | 1.887 |  | 1.887 |  | 57.547 |  |
| Keri |  | 1 |  | 0.943 |  | 0.943 |  | 58.491 |  |
| Keto |  | 1 |  | 0.943 |  | 0.943 |  | 59.434 |  |
| Kondor |  | 1 |  | 0.943 |  | 0.943 |  | 60.377 |  |
| Kundur |  | 1 |  | 0.943 |  | 0.943 |  | 61.321 |  |
| Madalena |  | 1 |  | 0.943 |  | 0.943 |  | 62.264 |  |
| Male |  | 1 |  | 0.943 |  | 0.943 |  | 63.208 |  |
| Mindi |  | 1 |  | 0.943 |  | 0.943 |  | 64.151 |  |
| Ompung |  | 2 |  | 1.887 |  | 1.887 |  | 66.038 |  |
| Pensi |  | 1 |  | 0.943 |  | 0.943 |  | 66.981 |  |
| Peot |  | 1 |  | 0.943 |  | 0.943 |  | 67.925 |  |
| Prabu |  | 1 |  | 0.943 |  | 0.943 |  | 68.868 |  |
| Raffi |  | 1 |  | 0.943 |  | 0.943 |  | 69.811 |  |
| Rambo |  | 1 |  | 0.943 |  | 0.943 |  | 70.755 |  |
| Ronaldo |  | 1 |  | 0.943 |  | 0.943 |  | 71.698 |  |
| Salvador |  | 3 |  | 2.830 |  | 2.830 |  | 74.528 |  |
| Suci |  | 2 |  | 1.887 |  | 1.887 |  | 76.415 |  |
| Sultan |  | 1 |  | 0.943 |  | 0.943 |  | 77.358 |  |
| Sumi |  | 2 |  | 1.887 |  | 1.887 |  | 79.245 |  |
| Teju |  | 1 |  | 0.943 |  | 0.943 |  | 80.189 |  |
| Teresia |  | 3 |  | 2.830 |  | 2.830 |  | 83.019 |  |
| Timi |  | 2 |  | 1.887 |  | 1.887 |  | 84.906 |  |
| Tina |  | 2 |  | 1.887 |  | 1.887 |  | 86.792 |  |
| Travor |  | 1 |  | 0.943 |  | 0.943 |  | 87.736 |  |
| Umi |  | 1 |  | 0.943 |  | 0.943 |  | 88.679 |  |
| Unflm |  | 2 |  | 1.887 |  | 1.887 |  | 90.566 |  |
| Uok |  | 2 |  | 1.887 |  | 1.887 |  | 92.453 |  |
| Vulcan |  | 1 |  | 0.943 |  | 0.943 |  | 93.396 |  |
| Walimah |  | 1 |  | 0.943 |  | 0.943 |  | 94.340 |  |
| Wulan |  | 1 |  | 0.943 |  | 0.943 |  | 95.283 |  |
| XL |  | 1 |  | 0.943 |  | 0.943 |  | 96.226 |  |
| Xenix |  | 1 |  | 0.943 |  | 0.943 |  | 97.170 |  |
| Yanti |  | 1 |  | 0.943 |  | 0.943 |  | 98.113 |  |
| Zeus |  | 1 |  | 0.943 |  | 0.943 |  | 99.057 |  |
| Zorro |  | 1 |  | 0.943 |  | 0.943 |  | 100.000 |  |
| Missing |  | 0 |  | 0.000 |  |  |  |  |  |
| Total |  | 106 |  | 100.000 |  |  |  |  |  |
|  | | | | | | | | | |

## Descriptive Statistics, split by Population\_Dataset before exclusions

| Descriptive Statistics | | | | | | | | | | | | | | | | | | | | | | | | | | | | | | | | | | | | | | | | | | | | | | | | | | | | | | | | | | | | | | | | | | | | | | | | | | | | | | | | | | | | | | | | | | | | | | | | | | | | | | | | | | | | | | | | | | | | | | | | | |
| --- | --- | --- | --- | --- | --- | --- | --- | --- | --- | --- | --- | --- | --- | --- | --- | --- | --- | --- | --- | --- | --- | --- | --- | --- | --- | --- | --- | --- | --- | --- | --- | --- | --- | --- | --- | --- | --- | --- | --- | --- | --- | --- | --- | --- | --- | --- | --- | --- | --- | --- | --- | --- | --- | --- | --- | --- | --- | --- | --- | --- | --- | --- | --- | --- | --- | --- | --- | --- | --- | --- | --- | --- | --- | --- | --- | --- | --- | --- | --- | --- | --- | --- | --- | --- | --- | --- | --- | --- | --- | --- | --- | --- | --- | --- | --- | --- | --- | --- | --- | --- | --- | --- | --- | --- | --- | --- | --- | --- | --- | --- | --- | --- | --- | --- | --- | --- | --- | --- | --- | --- | --- |
|  | | E.frequency | | | | | | | | | | | | S.frequency | | | | | | | | | | | | C.frequency | | | | | | | | | | | | E.duration | | | | | | | | | | | | S.duration | | | | | | | | | | | | C.duration | | | | | | | | | | | | Sex | | | | | | | | | | | | AgeSex class | | | | | | | | | | | | Context | | | | | | | | | | | | Individual | | | | | | | | | | | |
|  | | Gunung Palung | | Sabangau | | Sampan Getek | | Sikundur | | Suaq | | Tuanan | | Gunung Palung | | Sabangau | | Sampan Getek | | Sikundur | | Suaq | | Tuanan | | Gunung Palung | | Sabangau | | Sampan Getek | | Sikundur | | Suaq | | Tuanan | | Gunung Palung | | Sabangau | | Sampan Getek | | Sikundur | | Suaq | | Tuanan | | Gunung Palung | | Sabangau | | Sampan Getek | | Sikundur | | Suaq | | Tuanan | | Gunung Palung | | Sabangau | | Sampan Getek | | Sikundur | | Suaq | | Tuanan | | Gunung Palung | | Sabangau | | Sampan Getek | | Sikundur | | Suaq | | Tuanan | | Gunung Palung | | Sabangau | | Sampan Getek | | Sikundur | | Suaq | | Tuanan | | Gunung Palung | | Sabangau | | Sampan Getek | | Sikundur | | Suaq | | Tuanan | | Gunung Palung | | Sabangau | | Sampan Getek | | Sikundur | | Suaq | | Tuanan | |
| Valid |  | 22 |  | 31 |  | 7 |  | 15 |  | 16 |  | 15 |  | 22 |  | 31 |  | 7 |  | 15 |  | 16 |  | 15 |  | 22 |  | 31 |  | 7 |  | 15 |  | 16 |  | 15 |  | 22 |  | 31 |  | 7 |  | 15 |  | 16 |  | 15 |  | 22 |  | 31 |  | 7 |  | 15 |  | 16 |  | 15 |  | 22 |  | 31 |  | 7 |  | 15 |  | 16 |  | 15 |  | 22 |  | 31 |  | 7 |  | 15 |  | 16 |  | 15 |  | 22 |  | 31 |  | 7 |  | 15 |  | 16 |  | 15 |  | 22 |  | 31 |  | 7 |  | 15 |  | 16 |  | 14 |  | 22 |  | 31 |  | 7 |  | 15 |  | 16 |  | 15 |  |
| Missing |  | 0 |  | 0 |  | 0 |  | 0 |  | 0 |  | 0 |  | 0 |  | 0 |  | 0 |  | 0 |  | 0 |  | 0 |  | 0 |  | 0 |  | 0 |  | 0 |  | 0 |  | 0 |  | 0 |  | 0 |  | 0 |  | 0 |  | 0 |  | 0 |  | 0 |  | 0 |  | 0 |  | 0 |  | 0 |  | 0 |  | 0 |  | 0 |  | 0 |  | 0 |  | 0 |  | 0 |  | 0 |  | 0 |  | 0 |  | 0 |  | 0 |  | 0 |  | 0 |  | 0 |  | 0 |  | 0 |  | 0 |  | 0 |  | 0 |  | 0 |  | 0 |  | 0 |  | 0 |  | 1 |  | 0 |  | 0 |  | 0 |  | 0 |  | 0 |  | 0 |  |
| Mean |  | 0.605 |  | 0.358 |  | 0.698 |  | 0.624 |  | 0.533 |  | 0.645 |  | 0.395 |  | 0.642 |  | 0.302 |  | 0.376 |  | 0.467 |  | 0.355 |  | 0.794 |  | 0.534 |  | 0.787 |  | 0.741 |  | 0.409 |  | 0.742 |  | 0.708 |  | 0.344 |  | 0.756 |  | 0.644 |  | 0.522 |  | 0.736 |  | 0.292 |  | 0.656 |  | 0.244 |  | 0.356 |  | 0.478 |  | 0.264 |  | 0.636 |  | 0.521 |  | 0.653 |  | 0.686 |  | 0.427 |  | 0.698 |  |  |  |  |  |  |  |  |  |  |  |  |  |  |  |  |  |  |  |  |  |  |  |  |  |  |  |  |  |  |  |  |  |  |  |  |  |  |  |  |  |  |  |  |  |  |  |  |  |
| Std. Deviation |  | 0.206 |  | 0.315 |  | 0.128 |  | 0.230 |  | 0.396 |  | 0.216 |  | 0.206 |  | 0.315 |  | 0.128 |  | 0.230 |  | 0.396 |  | 0.216 |  | 0.151 |  | 0.419 |  | 0.229 |  | 0.301 |  | 0.358 |  | 0.243 |  | 0.223 |  | 0.314 |  | 0.157 |  | 0.249 |  | 0.390 |  | 0.146 |  | 0.223 |  | 0.314 |  | 0.157 |  | 0.249 |  | 0.390 |  | 0.146 |  | 0.203 |  | 0.421 |  | 0.244 |  | 0.281 |  | 0.377 |  | 0.263 |  |  |  |  |  |  |  |  |  |  |  |  |  |  |  |  |  |  |  |  |  |  |  |  |  |  |  |  |  |  |  |  |  |  |  |  |  |  |  |  |  |  |  |  |  |  |  |  |  |
| Minimum |  | 0.292 |  | 0.000 |  | 0.570 |  | 0.000 |  | 0.000 |  | 0.164 |  | 0.134 |  | 0.176 |  | 0.112 |  | 0.064 |  | 0.071 |  | 0.084 |  | 0.464 |  | 0.000 |  | 0.399 |  | 0.000 |  | 0.000 |  | 0.308 |  | 0.276 |  | 0.000 |  | 0.439 |  | 0.000 |  | 0.000 |  | 0.452 |  | 0.060 |  | 0.160 |  | 0.054 |  | 0.095 |  | 0.082 |  | 0.063 |  | 0.226 |  | 0.000 |  | 0.205 |  | 0.000 |  | 0.000 |  | 0.237 |  |  |  |  |  |  |  |  |  |  |  |  |  |  |  |  |  |  |  |  |  |  |  |  |  |  |  |  |  |  |  |  |  |  |  |  |  |  |  |  |  |  |  |  |  |  |  |  |  |
| Maximum |  | 0.866 |  | 0.824 |  | 0.888 |  | 0.936 |  | 0.929 |  | 0.916 |  | 0.708 |  | 1.000 |  | 0.430 |  | 1.000 |  | 1.000 |  | 0.836 |  | 0.998 |  | 0.998 |  | 0.980 |  | 0.998 |  | 0.998 |  | 0.997 |  | 0.940 |  | 0.840 |  | 0.946 |  | 0.905 |  | 0.918 |  | 0.937 |  | 0.724 |  | 1.000 |  | 0.561 |  | 1.000 |  | 1.000 |  | 0.548 |  | 0.993 |  | 0.998 |  | 0.985 |  | 1.000 |  | 0.998 |  | 0.991 |  |  |  |  |  |  |  |  |  |  |  |  |  |  |  |  |  |  |  |  |  |  |  |  |  |  |  |  |  |  |  |  |  |  |  |  |  |  |  |  |  |  |  |  |  |  |  |  |  |
|  | | | | | | | | | | | | | | | | | | | | | | | | | | | | | | | | | | | | | | | | | | | | | | | | | | | | | | | | | | | | | | | | | | | | | | | | | | | | | | | | | | | | | | | | | | | | | | | | | | | | | | | | | | | | | | | | | | | | | | | | | |
|  |  |  |  |  |  |  |  |  |  |  |  |  |  |  |  |  |  |  |  |  |  |  |  |  |  |  |  |  |  |  |  |  |  |  |  |  |  |  |  |  |  |  |  |  |  |  |  |  |  |  |  |  |  |  |  |  |  |  |  |  |  |  |  |  |  |  |  |  |  |  |  |  |  |  |  |  |  |  |  |  |  |  |  |  |  |  |  |  |  |  |  |  |  |  |  |  |  |  |  |  |  |  |  |  |  |  |  |  |  |  |  |  |  |  |  |  |  |  |  |  |  |
| --- | --- | --- | --- | --- | --- | --- | --- | --- | --- | --- | --- | --- | --- | --- | --- | --- | --- | --- | --- | --- | --- | --- | --- | --- | --- | --- | --- | --- | --- | --- | --- | --- | --- | --- | --- | --- | --- | --- | --- | --- | --- | --- | --- | --- | --- | --- | --- | --- | --- | --- | --- | --- | --- | --- | --- | --- | --- | --- | --- | --- | --- | --- | --- | --- | --- | --- | --- | --- | --- | --- | --- | --- | --- | --- | --- | --- | --- | --- | --- | --- | --- | --- | --- | --- | --- | --- | --- | --- | --- | --- | --- | --- | --- | --- | --- | --- | --- | --- | --- | --- | --- | --- | --- | --- | --- | --- | --- | --- | --- | --- | --- | --- | --- | --- | --- | --- | --- | --- | --- | --- | --- |
| *Note.*  Not all values are available for *Nominal Text* variables | | | | | | | | | | | | | | | | | | | | | | | | | | | | | | | | | | | | | | | | | | | | | | | | | | | | | | | | | | | | | | | | | | | | | | | | | | | | | | | | | | | | | | | | | | | | | | | | | | | | | | | | | | | | | | | | | | | | | | | | | |

### Frequency Tables

| Frequencies for Sex | | | | | | | | | | | |
| --- | --- | --- | --- | --- | --- | --- | --- | --- | --- | --- | --- |
| Population | | Sex | | Frequency | | Percent | | Valid Percent | | Cumulative Percent | |
| Gunung Palung |  | female |  | 13 |  | 59.091 |  | 59.091 |  | 59.091 |  |
|  |  | male |  | 9 |  | 40.909 |  | 40.909 |  | 100.000 |  |
|  |  | Missing |  | 0 |  | 0.000 |  |  |  |  |  |
|  |  | Total |  | 22 |  | 100.000 |  |  |  |  |  |
| Sabangau |  | female |  | 17 |  | 54.839 |  | 54.839 |  | 54.839 |  |
|  |  | male |  | 14 |  | 45.161 |  | 45.161 |  | 100.000 |  |
|  |  | Missing |  | 0 |  | 0.000 |  |  |  |  |  |
|  |  | Total |  | 31 |  | 100.000 |  |  |  |  |  |
| Sampan Getek |  | female |  | 6 |  | 85.714 |  | 85.714 |  | 85.714 |  |
|  |  | male |  | 1 |  | 14.286 |  | 14.286 |  | 100.000 |  |
|  |  | Missing |  | 0 |  | 0.000 |  |  |  |  |  |
|  |  | Total |  | 7 |  | 100.000 |  |  |  |  |  |
| Sikundur |  | female |  | 7 |  | 46.667 |  | 46.667 |  | 46.667 |  |
|  |  | male |  | 8 |  | 53.333 |  | 53.333 |  | 100.000 |  |
|  |  | Missing |  | 0 |  | 0.000 |  |  |  |  |  |
|  |  | Total |  | 15 |  | 100.000 |  |  |  |  |  |
| Suaq |  | female |  | 11 |  | 68.750 |  | 68.750 |  | 68.750 |  |
|  |  | male |  | 5 |  | 31.250 |  | 31.250 |  | 100.000 |  |
|  |  | Missing |  | 0 |  | 0.000 |  |  |  |  |  |
|  |  | Total |  | 16 |  | 100.000 |  |  |  |  |  |
| Tuanan |  | female |  | 7 |  | 46.667 |  | 46.667 |  | 46.667 |  |
|  |  | male |  | 8 |  | 53.333 |  | 53.333 |  | 100.000 |  |
|  |  | Missing |  | 0 |  | 0.000 |  |  |  |  |  |
|  |  | Total |  | 15 |  | 100.000 |  |  |  |  |  |
|  | | | | | | | | | | | |

| Frequencies for AgeSex class | | | | | | | | | | | |
| --- | --- | --- | --- | --- | --- | --- | --- | --- | --- | --- | --- |
| Population | | AgeSex class | | Frequency | | Percent | | Valid Percent | | Cumulative Percent | |
| Gunung Palung |  | adolescent |  | 3 |  | 13.636 |  | 13.636 |  | 13.636 |  |
|  |  | female with infant |  | 8 |  | 36.364 |  | 36.364 |  | 50.000 |  |
|  |  | flanged male |  | 4 |  | 18.182 |  | 18.182 |  | 68.182 |  |
|  |  | infant |  | 5 |  | 22.727 |  | 22.727 |  | 90.909 |  |
|  |  | unflanged male |  | 2 |  | 9.091 |  | 9.091 |  | 100.000 |  |
|  |  | Missing |  | 0 |  | 0.000 |  |  |  |  |  |
|  |  | Total |  | 22 |  | 100.000 |  |  |  |  |  |
| Sabangau |  | adolescent |  | 7 |  | 22.581 |  | 22.581 |  | 22.581 |  |
|  |  | female with infant |  | 8 |  | 25.806 |  | 25.806 |  | 48.387 |  |
|  |  | flanged male |  | 5 |  | 16.129 |  | 16.129 |  | 64.516 |  |
|  |  | infant |  | 8 |  | 25.806 |  | 25.806 |  | 90.323 |  |
|  |  | unflanged male |  | 3 |  | 9.677 |  | 9.677 |  | 100.000 |  |
|  |  | Missing |  | 0 |  | 0.000 |  |  |  |  |  |
|  |  | Total |  | 31 |  | 100.000 |  |  |  |  |  |
| Sampan Getek |  | adolescent |  | 1 |  | 14.286 |  | 14.286 |  | 14.286 |  |
|  |  | female with infant |  | 4 |  | 57.143 |  | 57.143 |  | 71.429 |  |
|  |  | flanged male |  | 1 |  | 14.286 |  | 14.286 |  | 85.714 |  |
|  |  | infant |  | 1 |  | 14.286 |  | 14.286 |  | 100.000 |  |
|  |  | unflanged male |  | 0 |  | 0.000 |  | 0.000 |  | 100.000 |  |
|  |  | Missing |  | 0 |  | 0.000 |  |  |  |  |  |
|  |  | Total |  | 7 |  | 100.000 |  |  |  |  |  |
| Sikundur |  | adolescent |  | 0 |  | 0.000 |  | 0.000 |  | 0.000 |  |
|  |  | female with infant |  | 7 |  | 46.667 |  | 46.667 |  | 46.667 |  |
|  |  | flanged male |  | 3 |  | 20.000 |  | 20.000 |  | 66.667 |  |
|  |  | infant |  | 0 |  | 0.000 |  | 0.000 |  | 66.667 |  |
|  |  | unflanged male |  | 5 |  | 33.333 |  | 33.333 |  | 100.000 |  |
|  |  | Missing |  | 0 |  | 0.000 |  |  |  |  |  |
|  |  | Total |  | 15 |  | 100.000 |  |  |  |  |  |
| Suaq |  | adolescent |  | 7 |  | 43.750 |  | 43.750 |  | 43.750 |  |
|  |  | female with infant |  | 4 |  | 25.000 |  | 25.000 |  | 68.750 |  |
|  |  | flanged male |  | 1 |  | 6.250 |  | 6.250 |  | 75.000 |  |
|  |  | infant |  | 2 |  | 12.500 |  | 12.500 |  | 87.500 |  |
|  |  | unflanged male |  | 2 |  | 12.500 |  | 12.500 |  | 100.000 |  |
|  |  | Missing |  | 0 |  | 0.000 |  |  |  |  |  |
|  |  | Total |  | 16 |  | 100.000 |  |  |  |  |  |
| Tuanan |  | adolescent |  | 1 |  | 6.667 |  | 6.667 |  | 6.667 |  |
|  |  | female with infant |  | 5 |  | 33.333 |  | 33.333 |  | 40.000 |  |
|  |  | flanged male |  | 8 |  | 53.333 |  | 53.333 |  | 93.333 |  |
|  |  | infant |  | 1 |  | 6.667 |  | 6.667 |  | 100.000 |  |
|  |  | unflanged male |  | 0 |  | 0.000 |  | 0.000 |  | 100.000 |  |
|  |  | Missing |  | 0 |  | 0.000 |  |  |  |  |  |
|  |  | Total |  | 15 |  | 100.000 |  |  |  |  |  |
|  | | | | | | | | | | | |

| Frequencies for Context | | | | | | | | | | | |
| --- | --- | --- | --- | --- | --- | --- | --- | --- | --- | --- | --- |
| Population | | Context | | Frequency | | Percent | | Valid Percent | | Cumulative Percent | |
| Gunung Palung |  | no apparent danger |  | 3 |  | 13.636 |  | 13.636 |  | 13.636 |  |
|  |  | towards animals |  | 0 |  | 0.000 |  | 0.000 |  | 13.636 |  |
|  |  | towards humans (non-observers) |  | 0 |  | 0.000 |  | 0.000 |  | 13.636 |  |
|  |  | towards observers |  | 18 |  | 81.818 |  | 81.818 |  | 95.455 |  |
|  |  | towards other orangutans |  | 1 |  | 4.545 |  | 4.545 |  | 100.000 |  |
|  |  | Missing |  | 0 |  | 0.000 |  |  |  |  |  |
|  |  | Total |  | 22 |  | 100.000 |  |  |  |  |  |
| Sabangau |  | no apparent danger |  | 0 |  | 0.000 |  | 0.000 |  | 0.000 |  |
|  |  | towards animals |  | 4 |  | 12.903 |  | 12.903 |  | 12.903 |  |
|  |  | towards humans (non-observers) |  | 0 |  | 0.000 |  | 0.000 |  | 12.903 |  |
|  |  | towards observers |  | 15 |  | 48.387 |  | 48.387 |  | 61.290 |  |
|  |  | towards other orangutans |  | 12 |  | 38.710 |  | 38.710 |  | 100.000 |  |
|  |  | Missing |  | 0 |  | 0.000 |  |  |  |  |  |
|  |  | Total |  | 31 |  | 100.000 |  |  |  |  |  |
| Sampan Getek |  | no apparent danger |  | 0 |  | 0.000 |  | 0.000 |  | 0.000 |  |
|  |  | towards animals |  | 0 |  | 0.000 |  | 0.000 |  | 0.000 |  |
|  |  | towards humans (non-observers) |  | 0 |  | 0.000 |  | 0.000 |  | 0.000 |  |
|  |  | towards observers |  | 7 |  | 100.000 |  | 100.000 |  | 100.000 |  |
|  |  | towards other orangutans |  | 0 |  | 0.000 |  | 0.000 |  | 100.000 |  |
|  |  | Missing |  | 0 |  | 0.000 |  |  |  |  |  |
|  |  | Total |  | 7 |  | 100.000 |  |  |  |  |  |
| Sikundur |  | no apparent danger |  | 0 |  | 0.000 |  | 0.000 |  | 0.000 |  |
|  |  | towards animals |  | 5 |  | 33.333 |  | 33.333 |  | 33.333 |  |
|  |  | towards humans (non-observers) |  | 1 |  | 6.667 |  | 6.667 |  | 40.000 |  |
|  |  | towards observers |  | 9 |  | 60.000 |  | 60.000 |  | 100.000 |  |
|  |  | towards other orangutans |  | 0 |  | 0.000 |  | 0.000 |  | 100.000 |  |
|  |  | Missing |  | 0 |  | 0.000 |  |  |  |  |  |
|  |  | Total |  | 15 |  | 100.000 |  |  |  |  |  |
| Suaq |  | no apparent danger |  | 1 |  | 6.250 |  | 6.250 |  | 6.250 |  |
|  |  | towards animals |  | 2 |  | 12.500 |  | 12.500 |  | 18.750 |  |
|  |  | towards humans (non-observers) |  | 0 |  | 0.000 |  | 0.000 |  | 18.750 |  |
|  |  | towards observers |  | 10 |  | 62.500 |  | 62.500 |  | 81.250 |  |
|  |  | towards other orangutans |  | 3 |  | 18.750 |  | 18.750 |  | 100.000 |  |
|  |  | Missing |  | 0 |  | 0.000 |  |  |  |  |  |
|  |  | Total |  | 16 |  | 100.000 |  |  |  |  |  |
| Tuanan |  | no apparent danger |  | 0 |  | 0.000 |  | 0.000 |  | 0.000 |  |
|  |  | towards animals |  | 3 |  | 20.000 |  | 21.429 |  | 21.429 |  |
|  |  | towards humans (non-observers) |  | 0 |  | 0.000 |  | 0.000 |  | 21.429 |  |
|  |  | towards observers |  | 10 |  | 66.667 |  | 71.429 |  | 92.857 |  |
|  |  | towards other orangutans |  | 1 |  | 6.667 |  | 7.143 |  | 100.000 |  |
|  |  | Missing |  | 1 |  | 6.667 |  |  |  |  |  |
|  |  | Total |  | 15 |  | 100.000 |  |  |  |  |  |
|  | | | | | | | | | | | |

| Frequencies for Individual | | | | | | | | | | | |
| --- | --- | --- | --- | --- | --- | --- | --- | --- | --- | --- | --- |
| Population | | Individual | | Frequency | | Percent | | Valid Percent | | Cumulative Percent | |
| Gunung Palung |  | Alice |  | 0 |  | 0.000 |  | 0.000 |  | 0.000 |  |
|  |  | Aminah |  | 1 |  | 4.545 |  | 4.545 |  | 4.545 |  |
|  |  | Anto |  | 0 |  | 0.000 |  | 0.000 |  | 4.545 |  |
|  |  | Asny |  | 1 |  | 4.545 |  | 4.545 |  | 9.091 |  |
|  |  | Bagong |  | 0 |  | 0.000 |  | 0.000 |  | 9.091 |  |
|  |  | Bendot |  | 0 |  | 0.000 |  | 0.000 |  | 9.091 |  |
|  |  | Berani |  | 1 |  | 4.545 |  | 4.545 |  | 13.636 |  |
|  |  | Beth |  | 2 |  | 9.091 |  | 9.091 |  | 22.727 |  |
|  |  | Bibi |  | 1 |  | 4.545 |  | 4.545 |  | 27.273 |  |
|  |  | Bintang |  | 0 |  | 0.000 |  | 0.000 |  | 27.273 |  |
|  |  | Brutus |  | 0 |  | 0.000 |  | 0.000 |  | 27.273 |  |
|  |  | Chindy |  | 0 |  | 0.000 |  | 0.000 |  | 27.273 |  |
|  |  | Codet |  | 1 |  | 4.545 |  | 4.545 |  | 31.818 |  |
|  |  | Elly |  | 0 |  | 0.000 |  | 0.000 |  | 31.818 |  |
|  |  | Fajar |  | 1 |  | 4.545 |  | 4.545 |  | 36.364 |  |
|  |  | Feb |  | 0 |  | 0.000 |  | 0.000 |  | 36.364 |  |
|  |  | Female |  | 0 |  | 0.000 |  | 0.000 |  | 36.364 |  |
|  |  | Fio |  | 0 |  | 0.000 |  | 0.000 |  | 36.364 |  |
|  |  | Flanged male |  | 0 |  | 0.000 |  | 0.000 |  | 36.364 |  |
|  |  | Freddy |  | 0 |  | 0.000 |  | 0.000 |  | 36.364 |  |
|  |  | Friska |  | 0 |  | 0.000 |  | 0.000 |  | 36.364 |  |
|  |  | Fugit |  | 0 |  | 0.000 |  | 0.000 |  | 36.364 |  |
|  |  | Gangstah |  | 0 |  | 0.000 |  | 0.000 |  | 36.364 |  |
|  |  | Gordon |  | 1 |  | 4.545 |  | 4.545 |  | 40.909 |  |
|  |  | Gracia |  | 0 |  | 0.000 |  | 0.000 |  | 40.909 |  |
|  |  | Gretel |  | 0 |  | 0.000 |  | 0.000 |  | 40.909 |  |
|  |  | Henk |  | 0 |  | 0.000 |  | 0.000 |  | 40.909 |  |
|  |  | Icarus |  | 0 |  | 0.000 |  | 0.000 |  | 40.909 |  |
|  |  | Imp |  | 0 |  | 0.000 |  | 0.000 |  | 40.909 |  |
|  |  | Indah |  | 1 |  | 4.545 |  | 4.545 |  | 45.455 |  |
|  |  | Indi |  | 2 |  | 9.091 |  | 9.091 |  | 54.545 |  |
|  |  | Irma |  | 0 |  | 0.000 |  | 0.000 |  | 54.545 |  |
|  |  | James |  | 0 |  | 0.000 |  | 0.000 |  | 54.545 |  |
|  |  | Janda Tua |  | 1 |  | 4.545 |  | 4.545 |  | 59.091 |  |
|  |  | Jinak |  | 0 |  | 0.000 |  | 0.000 |  | 59.091 |  |
|  |  | Joy |  | 0 |  | 0.000 |  | 0.000 |  | 59.091 |  |
|  |  | Juni |  | 0 |  | 0.000 |  | 0.000 |  | 59.091 |  |
|  |  | Juno |  | 0 |  | 0.000 |  | 0.000 |  | 59.091 |  |
|  |  | Kacil |  | 0 |  | 0.000 |  | 0.000 |  | 59.091 |  |
|  |  | Kan |  | 1 |  | 4.545 |  | 4.545 |  | 63.636 |  |
|  |  | Kasi |  | 0 |  | 0.000 |  | 0.000 |  | 63.636 |  |
|  |  | Kay |  | 0 |  | 0.000 |  | 0.000 |  | 63.636 |  |
|  |  | Keri |  | 0 |  | 0.000 |  | 0.000 |  | 63.636 |  |
|  |  | Keto |  | 0 |  | 0.000 |  | 0.000 |  | 63.636 |  |
|  |  | Kondor |  | 0 |  | 0.000 |  | 0.000 |  | 63.636 |  |
|  |  | Kundur |  | 0 |  | 0.000 |  | 0.000 |  | 63.636 |  |
|  |  | Madalena |  | 0 |  | 0.000 |  | 0.000 |  | 63.636 |  |
|  |  | Male |  | 0 |  | 0.000 |  | 0.000 |  | 63.636 |  |
|  |  | Mindi |  | 0 |  | 0.000 |  | 0.000 |  | 63.636 |  |
|  |  | Ompung |  | 0 |  | 0.000 |  | 0.000 |  | 63.636 |  |
|  |  | Pensi |  | 0 |  | 0.000 |  | 0.000 |  | 63.636 |  |
|  |  | Peot |  | 1 |  | 4.545 |  | 4.545 |  | 68.182 |  |
|  |  | Prabu |  | 1 |  | 4.545 |  | 4.545 |  | 72.727 |  |
|  |  | Raffi |  | 0 |  | 0.000 |  | 0.000 |  | 72.727 |  |
|  |  | Rambo |  | 0 |  | 0.000 |  | 0.000 |  | 72.727 |  |
|  |  | Ronaldo |  | 0 |  | 0.000 |  | 0.000 |  | 72.727 |  |
|  |  | Salvador |  | 0 |  | 0.000 |  | 0.000 |  | 72.727 |  |
|  |  | Suci |  | 0 |  | 0.000 |  | 0.000 |  | 72.727 |  |
|  |  | Sultan |  | 0 |  | 0.000 |  | 0.000 |  | 72.727 |  |
|  |  | Sumi |  | 0 |  | 0.000 |  | 0.000 |  | 72.727 |  |
|  |  | Teju |  | 0 |  | 0.000 |  | 0.000 |  | 72.727 |  |
|  |  | Teresia |  | 0 |  | 0.000 |  | 0.000 |  | 72.727 |  |
|  |  | Timi |  | 0 |  | 0.000 |  | 0.000 |  | 72.727 |  |
|  |  | Tina |  | 0 |  | 0.000 |  | 0.000 |  | 72.727 |  |
|  |  | Travor |  | 0 |  | 0.000 |  | 0.000 |  | 72.727 |  |
|  |  | Umi |  | 1 |  | 4.545 |  | 4.545 |  | 77.273 |  |
|  |  | Unflm |  | 0 |  | 0.000 |  | 0.000 |  | 77.273 |  |
|  |  | Uok |  | 2 |  | 9.091 |  | 9.091 |  | 86.364 |  |
|  |  | Vulcan |  | 0 |  | 0.000 |  | 0.000 |  | 86.364 |  |
|  |  | Walimah |  | 1 |  | 4.545 |  | 4.545 |  | 90.909 |  |
|  |  | Wulan |  | 0 |  | 0.000 |  | 0.000 |  | 90.909 |  |
|  |  | XL |  | 1 |  | 4.545 |  | 4.545 |  | 95.455 |  |
|  |  | Xenix |  | 0 |  | 0.000 |  | 0.000 |  | 95.455 |  |
|  |  | Yanti |  | 0 |  | 0.000 |  | 0.000 |  | 95.455 |  |
|  |  | Zeus |  | 0 |  | 0.000 |  | 0.000 |  | 95.455 |  |
|  |  | Zorro |  | 1 |  | 4.545 |  | 4.545 |  | 100.000 |  |
|  |  | Missing |  | 0 |  | 0.000 |  |  |  |  |  |
|  |  | Total |  | 22 |  | 100.000 |  |  |  |  |  |
| Sabangau |  | Alice |  | 0 |  | 0.000 |  | 0.000 |  | 0.000 |  |
|  |  | Aminah |  | 0 |  | 0.000 |  | 0.000 |  | 0.000 |  |
|  |  | Anto |  | 0 |  | 0.000 |  | 0.000 |  | 0.000 |  |
|  |  | Asny |  | 0 |  | 0.000 |  | 0.000 |  | 0.000 |  |
|  |  | Bagong |  | 0 |  | 0.000 |  | 0.000 |  | 0.000 |  |
|  |  | Bendot |  | 0 |  | 0.000 |  | 0.000 |  | 0.000 |  |
|  |  | Berani |  | 0 |  | 0.000 |  | 0.000 |  | 0.000 |  |
|  |  | Beth |  | 0 |  | 0.000 |  | 0.000 |  | 0.000 |  |
|  |  | Bibi |  | 0 |  | 0.000 |  | 0.000 |  | 0.000 |  |
|  |  | Bintang |  | 0 |  | 0.000 |  | 0.000 |  | 0.000 |  |
|  |  | Brutus |  | 0 |  | 0.000 |  | 0.000 |  | 0.000 |  |
|  |  | Chindy |  | 0 |  | 0.000 |  | 0.000 |  | 0.000 |  |
|  |  | Codet |  | 0 |  | 0.000 |  | 0.000 |  | 0.000 |  |
|  |  | Elly |  | 0 |  | 0.000 |  | 0.000 |  | 0.000 |  |
|  |  | Fajar |  | 0 |  | 0.000 |  | 0.000 |  | 0.000 |  |
|  |  | Feb |  | 2 |  | 6.452 |  | 6.452 |  | 6.452 |  |
|  |  | Female |  | 2 |  | 6.452 |  | 6.452 |  | 12.903 |  |
|  |  | Fio |  | 3 |  | 9.677 |  | 9.677 |  | 22.581 |  |
|  |  | Flanged male |  | 0 |  | 0.000 |  | 0.000 |  | 22.581 |  |
|  |  | Freddy |  | 0 |  | 0.000 |  | 0.000 |  | 22.581 |  |
|  |  | Friska |  | 0 |  | 0.000 |  | 0.000 |  | 22.581 |  |
|  |  | Fugit |  | 0 |  | 0.000 |  | 0.000 |  | 22.581 |  |
|  |  | Gangstah |  | 0 |  | 0.000 |  | 0.000 |  | 22.581 |  |
|  |  | Gordon |  | 0 |  | 0.000 |  | 0.000 |  | 22.581 |  |
|  |  | Gracia |  | 3 |  | 9.677 |  | 9.677 |  | 32.258 |  |
|  |  | Gretel |  | 2 |  | 6.452 |  | 6.452 |  | 38.710 |  |
|  |  | Henk |  | 0 |  | 0.000 |  | 0.000 |  | 38.710 |  |
|  |  | Icarus |  | 2 |  | 6.452 |  | 6.452 |  | 45.161 |  |
|  |  | Imp |  | 0 |  | 0.000 |  | 0.000 |  | 45.161 |  |
|  |  | Indah |  | 0 |  | 0.000 |  | 0.000 |  | 45.161 |  |
|  |  | Indi |  | 0 |  | 0.000 |  | 0.000 |  | 45.161 |  |
|  |  | Irma |  | 0 |  | 0.000 |  | 0.000 |  | 45.161 |  |
|  |  | James |  | 0 |  | 0.000 |  | 0.000 |  | 45.161 |  |
|  |  | Janda Tua |  | 0 |  | 0.000 |  | 0.000 |  | 45.161 |  |
|  |  | Jinak |  | 0 |  | 0.000 |  | 0.000 |  | 45.161 |  |
|  |  | Joy |  | 2 |  | 6.452 |  | 6.452 |  | 51.613 |  |
|  |  | Juni |  | 0 |  | 0.000 |  | 0.000 |  | 51.613 |  |
|  |  | Juno |  | 1 |  | 3.226 |  | 3.226 |  | 54.839 |  |
|  |  | Kacil |  | 0 |  | 0.000 |  | 0.000 |  | 54.839 |  |
|  |  | Kan |  | 0 |  | 0.000 |  | 0.000 |  | 54.839 |  |
|  |  | Kasi |  | 0 |  | 0.000 |  | 0.000 |  | 54.839 |  |
|  |  | Kay |  | 0 |  | 0.000 |  | 0.000 |  | 54.839 |  |
|  |  | Keri |  | 0 |  | 0.000 |  | 0.000 |  | 54.839 |  |
|  |  | Keto |  | 1 |  | 3.226 |  | 3.226 |  | 58.065 |  |
|  |  | Kondor |  | 0 |  | 0.000 |  | 0.000 |  | 58.065 |  |
|  |  | Kundur |  | 0 |  | 0.000 |  | 0.000 |  | 58.065 |  |
|  |  | Madalena |  | 0 |  | 0.000 |  | 0.000 |  | 58.065 |  |
|  |  | Male |  | 0 |  | 0.000 |  | 0.000 |  | 58.065 |  |
|  |  | Mindi |  | 0 |  | 0.000 |  | 0.000 |  | 58.065 |  |
|  |  | Ompung |  | 0 |  | 0.000 |  | 0.000 |  | 58.065 |  |
|  |  | Pensi |  | 0 |  | 0.000 |  | 0.000 |  | 58.065 |  |
|  |  | Peot |  | 0 |  | 0.000 |  | 0.000 |  | 58.065 |  |
|  |  | Prabu |  | 0 |  | 0.000 |  | 0.000 |  | 58.065 |  |
|  |  | Raffi |  | 0 |  | 0.000 |  | 0.000 |  | 58.065 |  |
|  |  | Rambo |  | 0 |  | 0.000 |  | 0.000 |  | 58.065 |  |
|  |  | Ronaldo |  | 0 |  | 0.000 |  | 0.000 |  | 58.065 |  |
|  |  | Salvador |  | 3 |  | 9.677 |  | 9.677 |  | 67.742 |  |
|  |  | Suci |  | 0 |  | 0.000 |  | 0.000 |  | 67.742 |  |
|  |  | Sultan |  | 0 |  | 0.000 |  | 0.000 |  | 67.742 |  |
|  |  | Sumi |  | 0 |  | 0.000 |  | 0.000 |  | 67.742 |  |
|  |  | Teju |  | 0 |  | 0.000 |  | 0.000 |  | 67.742 |  |
|  |  | Teresia |  | 3 |  | 9.677 |  | 9.677 |  | 77.419 |  |
|  |  | Timi |  | 2 |  | 6.452 |  | 6.452 |  | 83.871 |  |
|  |  | Tina |  | 0 |  | 0.000 |  | 0.000 |  | 83.871 |  |
|  |  | Travor |  | 1 |  | 3.226 |  | 3.226 |  | 87.097 |  |
|  |  | Umi |  | 0 |  | 0.000 |  | 0.000 |  | 87.097 |  |
|  |  | Unflm |  | 2 |  | 6.452 |  | 6.452 |  | 93.548 |  |
|  |  | Uok |  | 0 |  | 0.000 |  | 0.000 |  | 93.548 |  |
|  |  | Vulcan |  | 1 |  | 3.226 |  | 3.226 |  | 96.774 |  |
|  |  | Walimah |  | 0 |  | 0.000 |  | 0.000 |  | 96.774 |  |
|  |  | Wulan |  | 0 |  | 0.000 |  | 0.000 |  | 96.774 |  |
|  |  | XL |  | 0 |  | 0.000 |  | 0.000 |  | 96.774 |  |
|  |  | Xenix |  | 0 |  | 0.000 |  | 0.000 |  | 96.774 |  |
|  |  | Yanti |  | 0 |  | 0.000 |  | 0.000 |  | 96.774 |  |
|  |  | Zeus |  | 1 |  | 3.226 |  | 3.226 |  | 100.000 |  |
|  |  | Zorro |  | 0 |  | 0.000 |  | 0.000 |  | 100.000 |  |
|  |  | Missing |  | 0 |  | 0.000 |  |  |  |  |  |
|  |  | Total |  | 31 |  | 100.000 |  |  |  |  |  |
| Sampan Getek |  | Alice |  | 0 |  | 0.000 |  | 0.000 |  | 0.000 |  |
|  |  | Aminah |  | 0 |  | 0.000 |  | 0.000 |  | 0.000 |  |
|  |  | Anto |  | 0 |  | 0.000 |  | 0.000 |  | 0.000 |  |
|  |  | Asny |  | 0 |  | 0.000 |  | 0.000 |  | 0.000 |  |
|  |  | Bagong |  | 1 |  | 14.286 |  | 14.286 |  | 14.286 |  |
|  |  | Bendot |  | 0 |  | 0.000 |  | 0.000 |  | 14.286 |  |
|  |  | Berani |  | 0 |  | 0.000 |  | 0.000 |  | 14.286 |  |
|  |  | Beth |  | 0 |  | 0.000 |  | 0.000 |  | 14.286 |  |
|  |  | Bibi |  | 0 |  | 0.000 |  | 0.000 |  | 14.286 |  |
|  |  | Bintang |  | 1 |  | 14.286 |  | 14.286 |  | 28.571 |  |
|  |  | Brutus |  | 0 |  | 0.000 |  | 0.000 |  | 28.571 |  |
|  |  | Chindy |  | 0 |  | 0.000 |  | 0.000 |  | 28.571 |  |
|  |  | Codet |  | 0 |  | 0.000 |  | 0.000 |  | 28.571 |  |
|  |  | Elly |  | 0 |  | 0.000 |  | 0.000 |  | 28.571 |  |
|  |  | Fajar |  | 0 |  | 0.000 |  | 0.000 |  | 28.571 |  |
|  |  | Feb |  | 0 |  | 0.000 |  | 0.000 |  | 28.571 |  |
|  |  | Female |  | 0 |  | 0.000 |  | 0.000 |  | 28.571 |  |
|  |  | Fio |  | 0 |  | 0.000 |  | 0.000 |  | 28.571 |  |
|  |  | Flanged male |  | 0 |  | 0.000 |  | 0.000 |  | 28.571 |  |
|  |  | Freddy |  | 0 |  | 0.000 |  | 0.000 |  | 28.571 |  |
|  |  | Friska |  | 0 |  | 0.000 |  | 0.000 |  | 28.571 |  |
|  |  | Fugit |  | 0 |  | 0.000 |  | 0.000 |  | 28.571 |  |
|  |  | Gangstah |  | 0 |  | 0.000 |  | 0.000 |  | 28.571 |  |
|  |  | Gordon |  | 0 |  | 0.000 |  | 0.000 |  | 28.571 |  |
|  |  | Gracia |  | 0 |  | 0.000 |  | 0.000 |  | 28.571 |  |
|  |  | Gretel |  | 0 |  | 0.000 |  | 0.000 |  | 28.571 |  |
|  |  | Henk |  | 0 |  | 0.000 |  | 0.000 |  | 28.571 |  |
|  |  | Icarus |  | 0 |  | 0.000 |  | 0.000 |  | 28.571 |  |
|  |  | Imp |  | 0 |  | 0.000 |  | 0.000 |  | 28.571 |  |
|  |  | Indah |  | 0 |  | 0.000 |  | 0.000 |  | 28.571 |  |
|  |  | Indi |  | 0 |  | 0.000 |  | 0.000 |  | 28.571 |  |
|  |  | Irma |  | 0 |  | 0.000 |  | 0.000 |  | 28.571 |  |
|  |  | James |  | 0 |  | 0.000 |  | 0.000 |  | 28.571 |  |
|  |  | Janda Tua |  | 0 |  | 0.000 |  | 0.000 |  | 28.571 |  |
|  |  | Jinak |  | 0 |  | 0.000 |  | 0.000 |  | 28.571 |  |
|  |  | Joy |  | 0 |  | 0.000 |  | 0.000 |  | 28.571 |  |
|  |  | Juni |  | 0 |  | 0.000 |  | 0.000 |  | 28.571 |  |
|  |  | Juno |  | 0 |  | 0.000 |  | 0.000 |  | 28.571 |  |
|  |  | Kacil |  | 1 |  | 14.286 |  | 14.286 |  | 42.857 |  |
|  |  | Kan |  | 0 |  | 0.000 |  | 0.000 |  | 42.857 |  |
|  |  | Kasi |  | 1 |  | 14.286 |  | 14.286 |  | 57.143 |  |
|  |  | Kay |  | 0 |  | 0.000 |  | 0.000 |  | 57.143 |  |
|  |  | Keri |  | 0 |  | 0.000 |  | 0.000 |  | 57.143 |  |
|  |  | Keto |  | 0 |  | 0.000 |  | 0.000 |  | 57.143 |  |
|  |  | Kondor |  | 0 |  | 0.000 |  | 0.000 |  | 57.143 |  |
|  |  | Kundur |  | 0 |  | 0.000 |  | 0.000 |  | 57.143 |  |
|  |  | Madalena |  | 0 |  | 0.000 |  | 0.000 |  | 57.143 |  |
|  |  | Male |  | 1 |  | 14.286 |  | 14.286 |  | 71.429 |  |
|  |  | Mindi |  | 0 |  | 0.000 |  | 0.000 |  | 71.429 |  |
|  |  | Ompung |  | 0 |  | 0.000 |  | 0.000 |  | 71.429 |  |
|  |  | Pensi |  | 1 |  | 14.286 |  | 14.286 |  | 85.714 |  |
|  |  | Peot |  | 0 |  | 0.000 |  | 0.000 |  | 85.714 |  |
|  |  | Prabu |  | 0 |  | 0.000 |  | 0.000 |  | 85.714 |  |
|  |  | Raffi |  | 0 |  | 0.000 |  | 0.000 |  | 85.714 |  |
|  |  | Rambo |  | 0 |  | 0.000 |  | 0.000 |  | 85.714 |  |
|  |  | Ronaldo |  | 0 |  | 0.000 |  | 0.000 |  | 85.714 |  |
|  |  | Salvador |  | 0 |  | 0.000 |  | 0.000 |  | 85.714 |  |
|  |  | Suci |  | 0 |  | 0.000 |  | 0.000 |  | 85.714 |  |
|  |  | Sultan |  | 0 |  | 0.000 |  | 0.000 |  | 85.714 |  |
|  |  | Sumi |  | 0 |  | 0.000 |  | 0.000 |  | 85.714 |  |
|  |  | Teju |  | 0 |  | 0.000 |  | 0.000 |  | 85.714 |  |
|  |  | Teresia |  | 0 |  | 0.000 |  | 0.000 |  | 85.714 |  |
|  |  | Timi |  | 0 |  | 0.000 |  | 0.000 |  | 85.714 |  |
|  |  | Tina |  | 0 |  | 0.000 |  | 0.000 |  | 85.714 |  |
|  |  | Travor |  | 0 |  | 0.000 |  | 0.000 |  | 85.714 |  |
|  |  | Umi |  | 0 |  | 0.000 |  | 0.000 |  | 85.714 |  |
|  |  | Unflm |  | 0 |  | 0.000 |  | 0.000 |  | 85.714 |  |
|  |  | Uok |  | 0 |  | 0.000 |  | 0.000 |  | 85.714 |  |
|  |  | Vulcan |  | 0 |  | 0.000 |  | 0.000 |  | 85.714 |  |
|  |  | Walimah |  | 0 |  | 0.000 |  | 0.000 |  | 85.714 |  |
|  |  | Wulan |  | 1 |  | 14.286 |  | 14.286 |  | 100.000 |  |
|  |  | XL |  | 0 |  | 0.000 |  | 0.000 |  | 100.000 |  |
|  |  | Xenix |  | 0 |  | 0.000 |  | 0.000 |  | 100.000 |  |
|  |  | Yanti |  | 0 |  | 0.000 |  | 0.000 |  | 100.000 |  |
|  |  | Zeus |  | 0 |  | 0.000 |  | 0.000 |  | 100.000 |  |
|  |  | Zorro |  | 0 |  | 0.000 |  | 0.000 |  | 100.000 |  |
|  |  | Missing |  | 0 |  | 0.000 |  |  |  |  |  |
|  |  | Total |  | 7 |  | 100.000 |  |  |  |  |  |
| Sikundur |  | Alice |  | 0 |  | 0.000 |  | 0.000 |  | 0.000 |  |
|  |  | Aminah |  | 0 |  | 0.000 |  | 0.000 |  | 0.000 |  |
|  |  | Anto |  | 1 |  | 6.667 |  | 6.667 |  | 6.667 |  |
|  |  | Asny |  | 0 |  | 0.000 |  | 0.000 |  | 6.667 |  |
|  |  | Bagong |  | 0 |  | 0.000 |  | 0.000 |  | 6.667 |  |
|  |  | Bendot |  | 1 |  | 6.667 |  | 6.667 |  | 13.333 |  |
|  |  | Berani |  | 0 |  | 0.000 |  | 0.000 |  | 13.333 |  |
|  |  | Beth |  | 0 |  | 0.000 |  | 0.000 |  | 13.333 |  |
|  |  | Bibi |  | 0 |  | 0.000 |  | 0.000 |  | 13.333 |  |
|  |  | Bintang |  | 0 |  | 0.000 |  | 0.000 |  | 13.333 |  |
|  |  | Brutus |  | 2 |  | 13.333 |  | 13.333 |  | 26.667 |  |
|  |  | Chindy |  | 0 |  | 0.000 |  | 0.000 |  | 26.667 |  |
|  |  | Codet |  | 0 |  | 0.000 |  | 0.000 |  | 26.667 |  |
|  |  | Elly |  | 0 |  | 0.000 |  | 0.000 |  | 26.667 |  |
|  |  | Fajar |  | 0 |  | 0.000 |  | 0.000 |  | 26.667 |  |
|  |  | Feb |  | 0 |  | 0.000 |  | 0.000 |  | 26.667 |  |
|  |  | Female |  | 0 |  | 0.000 |  | 0.000 |  | 26.667 |  |
|  |  | Fio |  | 0 |  | 0.000 |  | 0.000 |  | 26.667 |  |
|  |  | Flanged male |  | 0 |  | 0.000 |  | 0.000 |  | 26.667 |  |
|  |  | Freddy |  | 0 |  | 0.000 |  | 0.000 |  | 26.667 |  |
|  |  | Friska |  | 0 |  | 0.000 |  | 0.000 |  | 26.667 |  |
|  |  | Fugit |  | 0 |  | 0.000 |  | 0.000 |  | 26.667 |  |
|  |  | Gangstah |  | 0 |  | 0.000 |  | 0.000 |  | 26.667 |  |
|  |  | Gordon |  | 0 |  | 0.000 |  | 0.000 |  | 26.667 |  |
|  |  | Gracia |  | 0 |  | 0.000 |  | 0.000 |  | 26.667 |  |
|  |  | Gretel |  | 0 |  | 0.000 |  | 0.000 |  | 26.667 |  |
|  |  | Henk |  | 0 |  | 0.000 |  | 0.000 |  | 26.667 |  |
|  |  | Icarus |  | 0 |  | 0.000 |  | 0.000 |  | 26.667 |  |
|  |  | Imp |  | 0 |  | 0.000 |  | 0.000 |  | 26.667 |  |
|  |  | Indah |  | 0 |  | 0.000 |  | 0.000 |  | 26.667 |  |
|  |  | Indi |  | 0 |  | 0.000 |  | 0.000 |  | 26.667 |  |
|  |  | Irma |  | 3 |  | 20.000 |  | 20.000 |  | 46.667 |  |
|  |  | James |  | 1 |  | 6.667 |  | 6.667 |  | 53.333 |  |
|  |  | Janda Tua |  | 0 |  | 0.000 |  | 0.000 |  | 53.333 |  |
|  |  | Jinak |  | 0 |  | 0.000 |  | 0.000 |  | 53.333 |  |
|  |  | Joy |  | 0 |  | 0.000 |  | 0.000 |  | 53.333 |  |
|  |  | Juni |  | 0 |  | 0.000 |  | 0.000 |  | 53.333 |  |
|  |  | Juno |  | 0 |  | 0.000 |  | 0.000 |  | 53.333 |  |
|  |  | Kacil |  | 0 |  | 0.000 |  | 0.000 |  | 53.333 |  |
|  |  | Kan |  | 0 |  | 0.000 |  | 0.000 |  | 53.333 |  |
|  |  | Kasi |  | 0 |  | 0.000 |  | 0.000 |  | 53.333 |  |
|  |  | Kay |  | 0 |  | 0.000 |  | 0.000 |  | 53.333 |  |
|  |  | Keri |  | 0 |  | 0.000 |  | 0.000 |  | 53.333 |  |
|  |  | Keto |  | 0 |  | 0.000 |  | 0.000 |  | 53.333 |  |
|  |  | Kondor |  | 0 |  | 0.000 |  | 0.000 |  | 53.333 |  |
|  |  | Kundur |  | 1 |  | 6.667 |  | 6.667 |  | 60.000 |  |
|  |  | Madalena |  | 1 |  | 6.667 |  | 6.667 |  | 66.667 |  |
|  |  | Male |  | 0 |  | 0.000 |  | 0.000 |  | 66.667 |  |
|  |  | Mindi |  | 0 |  | 0.000 |  | 0.000 |  | 66.667 |  |
|  |  | Ompung |  | 2 |  | 13.333 |  | 13.333 |  | 80.000 |  |
|  |  | Pensi |  | 0 |  | 0.000 |  | 0.000 |  | 80.000 |  |
|  |  | Peot |  | 0 |  | 0.000 |  | 0.000 |  | 80.000 |  |
|  |  | Prabu |  | 0 |  | 0.000 |  | 0.000 |  | 80.000 |  |
|  |  | Raffi |  | 0 |  | 0.000 |  | 0.000 |  | 80.000 |  |
|  |  | Rambo |  | 0 |  | 0.000 |  | 0.000 |  | 80.000 |  |
|  |  | Ronaldo |  | 0 |  | 0.000 |  | 0.000 |  | 80.000 |  |
|  |  | Salvador |  | 0 |  | 0.000 |  | 0.000 |  | 80.000 |  |
|  |  | Suci |  | 2 |  | 13.333 |  | 13.333 |  | 93.333 |  |
|  |  | Sultan |  | 0 |  | 0.000 |  | 0.000 |  | 93.333 |  |
|  |  | Sumi |  | 0 |  | 0.000 |  | 0.000 |  | 93.333 |  |
|  |  | Teju |  | 0 |  | 0.000 |  | 0.000 |  | 93.333 |  |
|  |  | Teresia |  | 0 |  | 0.000 |  | 0.000 |  | 93.333 |  |
|  |  | Timi |  | 0 |  | 0.000 |  | 0.000 |  | 93.333 |  |
|  |  | Tina |  | 0 |  | 0.000 |  | 0.000 |  | 93.333 |  |
|  |  | Travor |  | 0 |  | 0.000 |  | 0.000 |  | 93.333 |  |
|  |  | Umi |  | 0 |  | 0.000 |  | 0.000 |  | 93.333 |  |
|  |  | Unflm |  | 0 |  | 0.000 |  | 0.000 |  | 93.333 |  |
|  |  | Uok |  | 0 |  | 0.000 |  | 0.000 |  | 93.333 |  |
|  |  | Vulcan |  | 0 |  | 0.000 |  | 0.000 |  | 93.333 |  |
|  |  | Walimah |  | 0 |  | 0.000 |  | 0.000 |  | 93.333 |  |
|  |  | Wulan |  | 0 |  | 0.000 |  | 0.000 |  | 93.333 |  |
|  |  | XL |  | 0 |  | 0.000 |  | 0.000 |  | 93.333 |  |
|  |  | Xenix |  | 0 |  | 0.000 |  | 0.000 |  | 93.333 |  |
|  |  | Yanti |  | 1 |  | 6.667 |  | 6.667 |  | 100.000 |  |
|  |  | Zeus |  | 0 |  | 0.000 |  | 0.000 |  | 100.000 |  |
|  |  | Zorro |  | 0 |  | 0.000 |  | 0.000 |  | 100.000 |  |
|  |  | Missing |  | 0 |  | 0.000 |  |  |  |  |  |
|  |  | Total |  | 15 |  | 100.000 |  |  |  |  |  |
| Suaq |  | Alice |  | 2 |  | 12.500 |  | 12.500 |  | 12.500 |  |
|  |  | Aminah |  | 0 |  | 0.000 |  | 0.000 |  | 12.500 |  |
|  |  | Anto |  | 0 |  | 0.000 |  | 0.000 |  | 12.500 |  |
|  |  | Asny |  | 0 |  | 0.000 |  | 0.000 |  | 12.500 |  |
|  |  | Bagong |  | 0 |  | 0.000 |  | 0.000 |  | 12.500 |  |
|  |  | Bendot |  | 0 |  | 0.000 |  | 0.000 |  | 12.500 |  |
|  |  | Berani |  | 0 |  | 0.000 |  | 0.000 |  | 12.500 |  |
|  |  | Beth |  | 0 |  | 0.000 |  | 0.000 |  | 12.500 |  |
|  |  | Bibi |  | 0 |  | 0.000 |  | 0.000 |  | 12.500 |  |
|  |  | Bintang |  | 0 |  | 0.000 |  | 0.000 |  | 12.500 |  |
|  |  | Brutus |  | 0 |  | 0.000 |  | 0.000 |  | 12.500 |  |
|  |  | Chindy |  | 2 |  | 12.500 |  | 12.500 |  | 25.000 |  |
|  |  | Codet |  | 0 |  | 0.000 |  | 0.000 |  | 25.000 |  |
|  |  | Elly |  | 3 |  | 18.750 |  | 18.750 |  | 43.750 |  |
|  |  | Fajar |  | 0 |  | 0.000 |  | 0.000 |  | 43.750 |  |
|  |  | Feb |  | 0 |  | 0.000 |  | 0.000 |  | 43.750 |  |
|  |  | Female |  | 0 |  | 0.000 |  | 0.000 |  | 43.750 |  |
|  |  | Fio |  | 0 |  | 0.000 |  | 0.000 |  | 43.750 |  |
|  |  | Flanged male |  | 0 |  | 0.000 |  | 0.000 |  | 43.750 |  |
|  |  | Freddy |  | 1 |  | 6.250 |  | 6.250 |  | 50.000 |  |
|  |  | Friska |  | 1 |  | 6.250 |  | 6.250 |  | 56.250 |  |
|  |  | Fugit |  | 0 |  | 0.000 |  | 0.000 |  | 56.250 |  |
|  |  | Gangstah |  | 1 |  | 6.250 |  | 6.250 |  | 62.500 |  |
|  |  | Gordon |  | 0 |  | 0.000 |  | 0.000 |  | 62.500 |  |
|  |  | Gracia |  | 0 |  | 0.000 |  | 0.000 |  | 62.500 |  |
|  |  | Gretel |  | 0 |  | 0.000 |  | 0.000 |  | 62.500 |  |
|  |  | Henk |  | 0 |  | 0.000 |  | 0.000 |  | 62.500 |  |
|  |  | Icarus |  | 0 |  | 0.000 |  | 0.000 |  | 62.500 |  |
|  |  | Imp |  | 1 |  | 6.250 |  | 6.250 |  | 68.750 |  |
|  |  | Indah |  | 0 |  | 0.000 |  | 0.000 |  | 68.750 |  |
|  |  | Indi |  | 0 |  | 0.000 |  | 0.000 |  | 68.750 |  |
|  |  | Irma |  | 0 |  | 0.000 |  | 0.000 |  | 68.750 |  |
|  |  | James |  | 0 |  | 0.000 |  | 0.000 |  | 68.750 |  |
|  |  | Janda Tua |  | 0 |  | 0.000 |  | 0.000 |  | 68.750 |  |
|  |  | Jinak |  | 0 |  | 0.000 |  | 0.000 |  | 68.750 |  |
|  |  | Joy |  | 0 |  | 0.000 |  | 0.000 |  | 68.750 |  |
|  |  | Juni |  | 0 |  | 0.000 |  | 0.000 |  | 68.750 |  |
|  |  | Juno |  | 0 |  | 0.000 |  | 0.000 |  | 68.750 |  |
|  |  | Kacil |  | 0 |  | 0.000 |  | 0.000 |  | 68.750 |  |
|  |  | Kan |  | 0 |  | 0.000 |  | 0.000 |  | 68.750 |  |
|  |  | Kasi |  | 0 |  | 0.000 |  | 0.000 |  | 68.750 |  |
|  |  | Kay |  | 0 |  | 0.000 |  | 0.000 |  | 68.750 |  |
|  |  | Keri |  | 0 |  | 0.000 |  | 0.000 |  | 68.750 |  |
|  |  | Keto |  | 0 |  | 0.000 |  | 0.000 |  | 68.750 |  |
|  |  | Kondor |  | 0 |  | 0.000 |  | 0.000 |  | 68.750 |  |
|  |  | Kundur |  | 0 |  | 0.000 |  | 0.000 |  | 68.750 |  |
|  |  | Madalena |  | 0 |  | 0.000 |  | 0.000 |  | 68.750 |  |
|  |  | Male |  | 0 |  | 0.000 |  | 0.000 |  | 68.750 |  |
|  |  | Mindi |  | 0 |  | 0.000 |  | 0.000 |  | 68.750 |  |
|  |  | Ompung |  | 0 |  | 0.000 |  | 0.000 |  | 68.750 |  |
|  |  | Pensi |  | 0 |  | 0.000 |  | 0.000 |  | 68.750 |  |
|  |  | Peot |  | 0 |  | 0.000 |  | 0.000 |  | 68.750 |  |
|  |  | Prabu |  | 0 |  | 0.000 |  | 0.000 |  | 68.750 |  |
|  |  | Raffi |  | 1 |  | 6.250 |  | 6.250 |  | 75.000 |  |
|  |  | Rambo |  | 0 |  | 0.000 |  | 0.000 |  | 75.000 |  |
|  |  | Ronaldo |  | 1 |  | 6.250 |  | 6.250 |  | 81.250 |  |
|  |  | Salvador |  | 0 |  | 0.000 |  | 0.000 |  | 81.250 |  |
|  |  | Suci |  | 0 |  | 0.000 |  | 0.000 |  | 81.250 |  |
|  |  | Sultan |  | 0 |  | 0.000 |  | 0.000 |  | 81.250 |  |
|  |  | Sumi |  | 0 |  | 0.000 |  | 0.000 |  | 81.250 |  |
|  |  | Teju |  | 0 |  | 0.000 |  | 0.000 |  | 81.250 |  |
|  |  | Teresia |  | 0 |  | 0.000 |  | 0.000 |  | 81.250 |  |
|  |  | Timi |  | 0 |  | 0.000 |  | 0.000 |  | 81.250 |  |
|  |  | Tina |  | 2 |  | 12.500 |  | 12.500 |  | 93.750 |  |
|  |  | Travor |  | 0 |  | 0.000 |  | 0.000 |  | 93.750 |  |
|  |  | Umi |  | 0 |  | 0.000 |  | 0.000 |  | 93.750 |  |
|  |  | Unflm |  | 0 |  | 0.000 |  | 0.000 |  | 93.750 |  |
|  |  | Uok |  | 0 |  | 0.000 |  | 0.000 |  | 93.750 |  |
|  |  | Vulcan |  | 0 |  | 0.000 |  | 0.000 |  | 93.750 |  |
|  |  | Walimah |  | 0 |  | 0.000 |  | 0.000 |  | 93.750 |  |
|  |  | Wulan |  | 0 |  | 0.000 |  | 0.000 |  | 93.750 |  |
|  |  | XL |  | 0 |  | 0.000 |  | 0.000 |  | 93.750 |  |
|  |  | Xenix |  | 1 |  | 6.250 |  | 6.250 |  | 100.000 |  |
|  |  | Yanti |  | 0 |  | 0.000 |  | 0.000 |  | 100.000 |  |
|  |  | Zeus |  | 0 |  | 0.000 |  | 0.000 |  | 100.000 |  |
|  |  | Zorro |  | 0 |  | 0.000 |  | 0.000 |  | 100.000 |  |
|  |  | Missing |  | 0 |  | 0.000 |  |  |  |  |  |
|  |  | Total |  | 16 |  | 100.000 |  |  |  |  |  |
| Tuanan |  | Alice |  | 0 |  | 0.000 |  | 0.000 |  | 0.000 |  |
|  |  | Aminah |  | 0 |  | 0.000 |  | 0.000 |  | 0.000 |  |
|  |  | Anto |  | 0 |  | 0.000 |  | 0.000 |  | 0.000 |  |
|  |  | Asny |  | 0 |  | 0.000 |  | 0.000 |  | 0.000 |  |
|  |  | Bagong |  | 0 |  | 0.000 |  | 0.000 |  | 0.000 |  |
|  |  | Bendot |  | 0 |  | 0.000 |  | 0.000 |  | 0.000 |  |
|  |  | Berani |  | 0 |  | 0.000 |  | 0.000 |  | 0.000 |  |
|  |  | Beth |  | 0 |  | 0.000 |  | 0.000 |  | 0.000 |  |
|  |  | Bibi |  | 0 |  | 0.000 |  | 0.000 |  | 0.000 |  |
|  |  | Bintang |  | 0 |  | 0.000 |  | 0.000 |  | 0.000 |  |
|  |  | Brutus |  | 0 |  | 0.000 |  | 0.000 |  | 0.000 |  |
|  |  | Chindy |  | 0 |  | 0.000 |  | 0.000 |  | 0.000 |  |
|  |  | Codet |  | 0 |  | 0.000 |  | 0.000 |  | 0.000 |  |
|  |  | Elly |  | 0 |  | 0.000 |  | 0.000 |  | 0.000 |  |
|  |  | Fajar |  | 0 |  | 0.000 |  | 0.000 |  | 0.000 |  |
|  |  | Feb |  | 0 |  | 0.000 |  | 0.000 |  | 0.000 |  |
|  |  | Female |  | 0 |  | 0.000 |  | 0.000 |  | 0.000 |  |
|  |  | Fio |  | 0 |  | 0.000 |  | 0.000 |  | 0.000 |  |
|  |  | Flanged male |  | 1 |  | 6.667 |  | 6.667 |  | 6.667 |  |
|  |  | Freddy |  | 0 |  | 0.000 |  | 0.000 |  | 6.667 |  |
|  |  | Friska |  | 0 |  | 0.000 |  | 0.000 |  | 6.667 |  |
|  |  | Fugit |  | 1 |  | 6.667 |  | 6.667 |  | 13.333 |  |
|  |  | Gangstah |  | 0 |  | 0.000 |  | 0.000 |  | 13.333 |  |
|  |  | Gordon |  | 0 |  | 0.000 |  | 0.000 |  | 13.333 |  |
|  |  | Gracia |  | 0 |  | 0.000 |  | 0.000 |  | 13.333 |  |
|  |  | Gretel |  | 0 |  | 0.000 |  | 0.000 |  | 13.333 |  |
|  |  | Henk |  | 1 |  | 6.667 |  | 6.667 |  | 20.000 |  |
|  |  | Icarus |  | 0 |  | 0.000 |  | 0.000 |  | 20.000 |  |
|  |  | Imp |  | 0 |  | 0.000 |  | 0.000 |  | 20.000 |  |
|  |  | Indah |  | 0 |  | 0.000 |  | 0.000 |  | 20.000 |  |
|  |  | Indi |  | 0 |  | 0.000 |  | 0.000 |  | 20.000 |  |
|  |  | Irma |  | 0 |  | 0.000 |  | 0.000 |  | 20.000 |  |
|  |  | James |  | 0 |  | 0.000 |  | 0.000 |  | 20.000 |  |
|  |  | Janda Tua |  | 0 |  | 0.000 |  | 0.000 |  | 20.000 |  |
|  |  | Jinak |  | 1 |  | 6.667 |  | 6.667 |  | 26.667 |  |
|  |  | Joy |  | 0 |  | 0.000 |  | 0.000 |  | 26.667 |  |
|  |  | Juni |  | 1 |  | 6.667 |  | 6.667 |  | 33.333 |  |
|  |  | Juno |  | 0 |  | 0.000 |  | 0.000 |  | 33.333 |  |
|  |  | Kacil |  | 0 |  | 0.000 |  | 0.000 |  | 33.333 |  |
|  |  | Kan |  | 0 |  | 0.000 |  | 0.000 |  | 33.333 |  |
|  |  | Kasi |  | 0 |  | 0.000 |  | 0.000 |  | 33.333 |  |
|  |  | Kay |  | 2 |  | 13.333 |  | 13.333 |  | 46.667 |  |
|  |  | Keri |  | 1 |  | 6.667 |  | 6.667 |  | 53.333 |  |
|  |  | Keto |  | 0 |  | 0.000 |  | 0.000 |  | 53.333 |  |
|  |  | Kondor |  | 1 |  | 6.667 |  | 6.667 |  | 60.000 |  |
|  |  | Kundur |  | 0 |  | 0.000 |  | 0.000 |  | 60.000 |  |
|  |  | Madalena |  | 0 |  | 0.000 |  | 0.000 |  | 60.000 |  |
|  |  | Male |  | 0 |  | 0.000 |  | 0.000 |  | 60.000 |  |
|  |  | Mindi |  | 1 |  | 6.667 |  | 6.667 |  | 66.667 |  |
|  |  | Ompung |  | 0 |  | 0.000 |  | 0.000 |  | 66.667 |  |
|  |  | Pensi |  | 0 |  | 0.000 |  | 0.000 |  | 66.667 |  |
|  |  | Peot |  | 0 |  | 0.000 |  | 0.000 |  | 66.667 |  |
|  |  | Prabu |  | 0 |  | 0.000 |  | 0.000 |  | 66.667 |  |
|  |  | Raffi |  | 0 |  | 0.000 |  | 0.000 |  | 66.667 |  |
|  |  | Rambo |  | 1 |  | 6.667 |  | 6.667 |  | 73.333 |  |
|  |  | Ronaldo |  | 0 |  | 0.000 |  | 0.000 |  | 73.333 |  |
|  |  | Salvador |  | 0 |  | 0.000 |  | 0.000 |  | 73.333 |  |
|  |  | Suci |  | 0 |  | 0.000 |  | 0.000 |  | 73.333 |  |
|  |  | Sultan |  | 1 |  | 6.667 |  | 6.667 |  | 80.000 |  |
|  |  | Sumi |  | 2 |  | 13.333 |  | 13.333 |  | 93.333 |  |
|  |  | Teju |  | 1 |  | 6.667 |  | 6.667 |  | 100.000 |  |
|  |  | Teresia |  | 0 |  | 0.000 |  | 0.000 |  | 100.000 |  |
|  |  | Timi |  | 0 |  | 0.000 |  | 0.000 |  | 100.000 |  |
|  |  | Tina |  | 0 |  | 0.000 |  | 0.000 |  | 100.000 |  |
|  |  | Travor |  | 0 |  | 0.000 |  | 0.000 |  | 100.000 |  |
|  |  | Umi |  | 0 |  | 0.000 |  | 0.000 |  | 100.000 |  |
|  |  | Unflm |  | 0 |  | 0.000 |  | 0.000 |  | 100.000 |  |
|  |  | Uok |  | 0 |  | 0.000 |  | 0.000 |  | 100.000 |  |
|  |  | Vulcan |  | 0 |  | 0.000 |  | 0.000 |  | 100.000 |  |
|  |  | Walimah |  | 0 |  | 0.000 |  | 0.000 |  | 100.000 |  |
|  |  | Wulan |  | 0 |  | 0.000 |  | 0.000 |  | 100.000 |  |
|  |  | XL |  | 0 |  | 0.000 |  | 0.000 |  | 100.000 |  |
|  |  | Xenix |  | 0 |  | 0.000 |  | 0.000 |  | 100.000 |  |
|  |  | Yanti |  | 0 |  | 0.000 |  | 0.000 |  | 100.000 |  |
|  |  | Zeus |  | 0 |  | 0.000 |  | 0.000 |  | 100.000 |  |
|  |  | Zorro |  | 0 |  | 0.000 |  | 0.000 |  | 100.000 |  |
|  |  | Missing |  | 0 |  | 0.000 |  |  |  |  |  |
|  |  | Total |  | 15 |  | 100.000 |  |  |  |  |  |
|  | | | | | | | | | | | |
