# Supplementary material for: Sociality predicts orangutan vocal phenotype
Source: Nat Ecol Evol. 2022 Mar 21;6(5):644–52. doi: 10.1038/s41559-022-01689-z (PMC9085614; doi:10.1038/s41559-022-01689-z)
Supplement: Supplementary file 5 — Descriptive statistics for entropic data, final. [file 41559_2022_1689_MOESM5_ESM.html]

JASP 


# Results

## Grand Descriptive Statistics\_Final dataset

| Descriptive Statistics | | | | | | | | | | | |
| --- | --- | --- | --- | --- | --- | --- | --- | --- | --- | --- | --- |
|  | E.duration | S.duration | C.duration | E.frequency | S.frequency | C.frequency | Sex | AgeSex class | Context | Species | Individual |
| Valid | 69 | 69 | 69 | 69 | 69 | 69 | 69 | 69 | 69 | 69 | 69 |
| Missing | 0 | 0 | 0 | 0 | 0 | 0 | 0 | 0 | 0 | 0 | 0 |
| Mean | 0.733 | 0.267 | 0.662 | 0.658 | 0.342 | 0.794 |  |  |  |  |  |
| Std. Deviation | 0.175 | 0.175 | 0.230 | 0.164 | 0.164 | 0.189 |  |  |  |  |  |
| Minimum | 0.118 | 0.054 | 0.205 | 0.164 | 0.064 | 0.239 |  |  |  |  |  |
| Maximum | 0.946 | 0.882 | 1.000 | 0.936 | 0.836 | 0.998 |  |  |  |  |  |
|  | | | | | | | | | | | |

### Frequency Tables

| Frequencies for Sex | | | | |
| --- | --- | --- | --- | --- |
| Sex | Frequency | Percent | Valid Percent | Cumulative Percent |
| female | 41 | 59.4 | 59.4 | 59.4 |
| male | 28 | 40.6 | 40.6 | 100.0 |
| Missing | 0 | 0.0 |  |  |
| Total | 69 | 100.0 |  |  |
|  | | | | |
|  |  |  |  |  |
| --- | --- | --- | --- | --- |
| *Note.*  The following variables have more than 10 distinct values and are omitted: E.duration, S.duration, C.duration, E.frequency, S.frequency, C.frequency, Individual. | | | | |

| Frequencies for AgeSex class | | | | |
| --- | --- | --- | --- | --- |
| AgeSex class | Frequency | Percent | Valid Percent | Cumulative Percent |
| adolescent | 12 | 17.4 | 17.4 | 17.4 |
| female with infant | 25 | 36.2 | 36.2 | 53.6 |
| flanged male | 16 | 23.2 | 23.2 | 76.8 |
| infant | 6 | 8.7 | 8.7 | 85.5 |
| unflanged male | 10 | 14.5 | 14.5 | 100.0 |
| Missing | 0 | 0.0 |  |  |
| Total | 69 | 100.0 |  |  |
|  | | | | |

| Frequencies for Context | | | | |
| --- | --- | --- | --- | --- |
| Context | Frequency | Percent | Valid Percent | Cumulative Percent |
| no apparent danger | 3 | 4.3 | 4.3 | 4.3 |
| towards animals | 10 | 14.5 | 14.5 | 18.8 |
| towards humans (non-observers) | 1 | 1.4 | 1.4 | 20.3 |
| towards observers | 55 | 79.7 | 79.7 | 100.0 |
| Missing | 0 | 0.0 |  |  |
| Total | 69 | 100.0 |  |  |
|  | | | | |

| Frequencies for Species | | | | |
| --- | --- | --- | --- | --- |
| Species | Frequency | Percent | Valid Percent | Cumulative Percent |
| Bornean | 41 | 59.4 | 59.4 | 59.4 |
| Sumatran | 28 | 40.6 | 40.6 | 100.0 |
| Missing | 0 | 0.0 |  |  |
| Total | 69 | 100.0 |  |  |
|  | | | | |

## Descriptive Statistics, split by Population\_Final dataset

| Descriptive Statistics | | | | | | | | | | | | | | | | | | | | | | | | | | | | | | | | | | | | | | | | | | | | | | | | | | | | | | | | | | | | | | | | | | |
| --- | --- | --- | --- | --- | --- | --- | --- | --- | --- | --- | --- | --- | --- | --- | --- | --- | --- | --- | --- | --- | --- | --- | --- | --- | --- | --- | --- | --- | --- | --- | --- | --- | --- | --- | --- | --- | --- | --- | --- | --- | --- | --- | --- | --- | --- | --- | --- | --- | --- | --- | --- | --- | --- | --- | --- | --- | --- | --- | --- | --- | --- | --- | --- | --- | --- | --- |
|  | E.duration | | | | | | S.duration | | | | | | C.duration | | | | | | E.frequency | | | | | | S.frequency | | | | | | C.frequency | | | | | | Sex | | | | | | AgeSex class | | | | | | Context | | | | | | Species | | | | | | Individual | | | | | |
|  | Gunung Palung | Sabangau | Sampan Getek | Sikundur | Suaq | Tuanan | Gunung Palung | Sabangau | Sampan Getek | Sikundur | Suaq | Tuanan | Gunung Palung | Sabangau | Sampan Getek | Sikundur | Suaq | Tuanan | Gunung Palung | Sabangau | Sampan Getek | Sikundur | Suaq | Tuanan | Gunung Palung | Sabangau | Sampan Getek | Sikundur | Suaq | Tuanan | Gunung Palung | Sabangau | Sampan Getek | Sikundur | Suaq | Tuanan | Gunung Palung | Sabangau | Sampan Getek | Sikundur | Suaq | Tuanan | Gunung Palung | Sabangau | Sampan Getek | Sikundur | Suaq | Tuanan | Gunung Palung | Sabangau | Sampan Getek | Sikundur | Suaq | Tuanan | Gunung Palung | Sabangau | Sampan Getek | Sikundur | Suaq | Tuanan | Gunung Palung | Sabangau | Sampan Getek | Sikundur | Suaq | Tuanan |
| Valid | 18 | 9 | 7 | 13 | 8 | 14 | 18 | 9 | 7 | 13 | 8 | 14 | 18 | 9 | 7 | 13 | 8 | 14 | 18 | 9 | 7 | 13 | 8 | 14 | 18 | 9 | 7 | 13 | 8 | 14 | 18 | 9 | 7 | 13 | 8 | 14 | 18 | 9 | 7 | 13 | 8 | 14 | 18 | 9 | 7 | 13 | 8 | 14 | 18 | 9 | 7 | 13 | 8 | 14 | 18 | 9 | 7 | 13 | 8 | 14 | 18 | 9 | 7 | 13 | 8 | 14 |
| Missing | 0 | 0 | 0 | 0 | 0 | 0 | 0 | 0 | 0 | 0 | 0 | 0 | 0 | 0 | 0 | 0 | 0 | 0 | 0 | 0 | 0 | 0 | 0 | 0 | 0 | 0 | 0 | 0 | 0 | 0 | 0 | 0 | 0 | 0 | 0 | 0 | 0 | 0 | 0 | 0 | 0 | 0 | 0 | 0 | 0 | 0 | 0 | 0 | 0 | 0 | 0 | 0 | 0 | 0 | 0 | 0 | 0 | 0 | 0 | 0 | 0 | 0 | 0 | 0 | 0 | 0 |
| Mean | 0.800 | 0.580 | 0.756 | 0.677 | 0.814 | 0.738 | 0.200 | 0.420 | 0.244 | 0.323 | 0.186 | 0.262 | 0.592 | 0.725 | 0.653 | 0.754 | 0.562 | 0.688 | 0.663 | 0.685 | 0.698 | 0.656 | 0.640 | 0.626 | 0.337 | 0.315 | 0.302 | 0.344 | 0.360 | 0.374 | 0.774 | 0.802 | 0.787 | 0.811 | 0.848 | 0.770 |  |  |  |  |  |  |  |  |  |  |  |  |  |  |  |  |  |  |  |  |  |  |  |  |  |  |  |  |  |  |
| Std. Deviation | 0.111 | 0.265 | 0.157 | 0.180 | 0.111 | 0.151 | 0.111 | 0.265 | 0.157 | 0.180 | 0.111 | 0.151 | 0.199 | 0.207 | 0.244 | 0.210 | 0.247 | 0.270 | 0.178 | 0.131 | 0.128 | 0.157 | 0.145 | 0.211 | 0.178 | 0.131 | 0.128 | 0.157 | 0.145 | 0.211 | 0.158 | 0.176 | 0.229 | 0.228 | 0.124 | 0.225 |  |  |  |  |  |  |  |  |  |  |  |  |  |  |  |  |  |  |  |  |  |  |  |  |  |  |  |  |  |  |
| Minimum | 0.458 | 0.118 | 0.439 | 0.276 | 0.636 | 0.452 | 0.060 | 0.160 | 0.054 | 0.095 | 0.082 | 0.063 | 0.226 | 0.415 | 0.205 | 0.344 | 0.301 | 0.237 | 0.292 | 0.452 | 0.570 | 0.413 | 0.344 | 0.164 | 0.134 | 0.176 | 0.112 | 0.064 | 0.186 | 0.084 | 0.464 | 0.580 | 0.399 | 0.239 | 0.605 | 0.308 |  |  |  |  |  |  |  |  |  |  |  |  |  |  |  |  |  |  |  |  |  |  |  |  |  |  |  |  |  |  |
| Maximum | 0.940 | 0.840 | 0.946 | 0.905 | 0.918 | 0.937 | 0.542 | 0.882 | 0.561 | 0.724 | 0.364 | 0.548 | 0.993 | 0.975 | 0.985 | 1.000 | 0.926 | 0.991 | 0.866 | 0.824 | 0.888 | 0.936 | 0.814 | 0.916 | 0.708 | 0.548 | 0.430 | 0.587 | 0.656 | 0.836 | 0.998 | 0.991 | 0.980 | 0.998 | 0.992 | 0.997 |  |  |  |  |  |  |  |  |  |  |  |  |  |  |  |  |  |  |  |  |  |  |  |  |  |  |  |  |  |  |
|  | | | | | | | | | | | | | | | | | | | | | | | | | | | | | | | | | | | | | | | | | | | | | | | | | | | | | | | | | | | | | | | | | | |

### Frequency Tables

| Frequencies for Sex | | | | | |
| --- | --- | --- | --- | --- | --- |
| Population | Sex | Frequency | Percent | Valid Percent | Cumulative Percent |
| Gunung Palung | female | 11 | 61.1 | 61.1 | 61.1 |
|  | male | 7 | 38.9 | 38.9 | 100.0 |
|  | Missing | 0 | 0.0 |  |  |
|  | Total | 18 | 100.0 |  |  |
| Sabangau | female | 6 | 66.7 | 66.7 | 66.7 |
|  | male | 3 | 33.3 | 33.3 | 100.0 |
|  | Missing | 0 | 0.0 |  |  |
|  | Total | 9 | 100.0 |  |  |
| Sampan Getek | female | 6 | 85.7 | 85.7 | 85.7 |
|  | male | 1 | 14.3 | 14.3 | 100.0 |
|  | Missing | 0 | 0.0 |  |  |
|  | Total | 7 | 100.0 |  |  |
| Sikundur | female | 6 | 46.2 | 46.2 | 46.2 |
|  | male | 7 | 53.8 | 53.8 | 100.0 |
|  | Missing | 0 | 0.0 |  |  |
|  | Total | 13 | 100.0 |  |  |
| Suaq | female | 5 | 62.5 | 62.5 | 62.5 |
|  | male | 3 | 37.5 | 37.5 | 100.0 |
|  | Missing | 0 | 0.0 |  |  |
|  | Total | 8 | 100.0 |  |  |
| Tuanan | female | 7 | 50.0 | 50.0 | 50.0 |
|  | male | 7 | 50.0 | 50.0 | 100.0 |
|  | Missing | 0 | 0.0 |  |  |
|  | Total | 14 | 100.0 |  |  |
|  | | | | | |
|  |  |  |  |  |  |
| --- | --- | --- | --- | --- | --- |
| *Note.*  The following variables have more than 10 distinct values and are omitted: E.duration, S.duration, C.duration, E.frequency, S.frequency, C.frequency, Individual. | | | | | |

| Frequencies for AgeSex class | | | | | |
| --- | --- | --- | --- | --- | --- |
| Population | AgeSex class | Frequency | Percent | Valid Percent | Cumulative Percent |
| Gunung Palung | adolescent | 3 | 16.7 | 16.7 | 16.7 |
|  | female with infant | 6 | 33.3 | 33.3 | 50.0 |
|  | flanged male | 4 | 22.2 | 22.2 | 72.2 |
|  | infant | 3 | 16.7 | 16.7 | 88.9 |
|  | unflanged male | 2 | 11.1 | 11.1 | 100.0 |
|  | Missing | 0 | 0.0 |  |  |
|  | Total | 18 | 100.0 |  |  |
| Sabangau | adolescent | 3 | 33.3 | 33.3 | 33.3 |
|  | female with infant | 3 | 33.3 | 33.3 | 66.7 |
|  | flanged male | 1 | 11.1 | 11.1 | 77.8 |
|  | infant | 1 | 11.1 | 11.1 | 88.9 |
|  | unflanged male | 1 | 11.1 | 11.1 | 100.0 |
|  | Missing | 0 | 0.0 |  |  |
|  | Total | 9 | 100.0 |  |  |
| Sampan Getek | adolescent | 1 | 14.3 | 14.3 | 14.3 |
|  | female with infant | 4 | 57.1 | 57.1 | 71.4 |
|  | flanged male | 1 | 14.3 | 14.3 | 85.7 |
|  | infant | 1 | 14.3 | 14.3 | 100.0 |
|  | unflanged male | 0 | 0.0 | 0.0 | 100.0 |
|  | Missing | 0 | 0.0 |  |  |
|  | Total | 7 | 100.0 |  |  |
| Sikundur | adolescent | 0 | 0.0 | 0.0 | 0.0 |
|  | female with infant | 6 | 46.2 | 46.2 | 46.2 |
|  | flanged male | 2 | 15.4 | 15.4 | 61.5 |
|  | infant | 0 | 0.0 | 0.0 | 61.5 |
|  | unflanged male | 5 | 38.5 | 38.5 | 100.0 |
|  | Missing | 0 | 0.0 |  |  |
|  | Total | 13 | 100.0 |  |  |
| Suaq | adolescent | 4 | 50.0 | 50.0 | 50.0 |
|  | female with infant | 1 | 12.5 | 12.5 | 62.5 |
|  | flanged male | 1 | 12.5 | 12.5 | 75.0 |
|  | infant | 0 | 0.0 | 0.0 | 75.0 |
|  | unflanged male | 2 | 25.0 | 25.0 | 100.0 |
|  | Missing | 0 | 0.0 |  |  |
|  | Total | 8 | 100.0 |  |  |
| Tuanan | adolescent | 1 | 7.1 | 7.1 | 7.1 |
|  | female with infant | 5 | 35.7 | 35.7 | 42.9 |
|  | flanged male | 7 | 50.0 | 50.0 | 92.9 |
|  | infant | 1 | 7.1 | 7.1 | 100.0 |
|  | unflanged male | 0 | 0.0 | 0.0 | 100.0 |
|  | Missing | 0 | 0.0 |  |  |
|  | Total | 14 | 100.0 |  |  |
|  | | | | | |

| Frequencies for Context | | | | | |
| --- | --- | --- | --- | --- | --- |
| Population | Context | Frequency | Percent | Valid Percent | Cumulative Percent |
| Gunung Palung | no apparent danger | 2 | 11.1 | 11.1 | 11.1 |
|  | towards animals | 0 | 0.0 | 0.0 | 11.1 |
|  | towards humans (non-observers) | 0 | 0.0 | 0.0 | 11.1 |
|  | towards observers | 16 | 88.9 | 88.9 | 100.0 |
|  | Missing | 0 | 0.0 |  |  |
|  | Total | 18 | 100.0 |  |  |
| Sabangau | no apparent danger | 0 | 0.0 | 0.0 | 0.0 |
|  | towards animals | 1 | 11.1 | 11.1 | 11.1 |
|  | towards humans (non-observers) | 0 | 0.0 | 0.0 | 11.1 |
|  | towards observers | 8 | 88.9 | 88.9 | 100.0 |
|  | Missing | 0 | 0.0 |  |  |
|  | Total | 9 | 100.0 |  |  |
| Sampan Getek | no apparent danger | 0 | 0.0 | 0.0 | 0.0 |
|  | towards animals | 0 | 0.0 | 0.0 | 0.0 |
|  | towards humans (non-observers) | 0 | 0.0 | 0.0 | 0.0 |
|  | towards observers | 7 | 100.0 | 100.0 | 100.0 |
|  | Missing | 0 | 0.0 |  |  |
|  | Total | 7 | 100.0 |  |  |
| Sikundur | no apparent danger | 0 | 0.0 | 0.0 | 0.0 |
|  | towards animals | 5 | 38.5 | 38.5 | 38.5 |
|  | towards humans (non-observers) | 1 | 7.7 | 7.7 | 46.2 |
|  | towards observers | 7 | 53.8 | 53.8 | 100.0 |
|  | Missing | 0 | 0.0 |  |  |
|  | Total | 13 | 100.0 |  |  |
| Suaq | no apparent danger | 0 | 0.0 | 0.0 | 0.0 |
|  | towards animals | 1 | 12.5 | 12.5 | 12.5 |
|  | towards humans (non-observers) | 0 | 0.0 | 0.0 | 12.5 |
|  | towards observers | 7 | 87.5 | 87.5 | 100.0 |
|  | Missing | 0 | 0.0 |  |  |
|  | Total | 8 | 100.0 |  |  |
| Tuanan | no apparent danger | 1 | 7.1 | 7.1 | 7.1 |
|  | towards animals | 3 | 21.4 | 21.4 | 28.6 |
|  | towards humans (non-observers) | 0 | 0.0 | 0.0 | 28.6 |
|  | towards observers | 10 | 71.4 | 71.4 | 100.0 |
|  | Missing | 0 | 0.0 |  |  |
|  | Total | 14 | 100.0 |  |  |
|  | | | | | |

| Frequencies for Species | | | | | |
| --- | --- | --- | --- | --- | --- |
| Population | Species | Frequency | Percent | Valid Percent | Cumulative Percent |
| Gunung Palung | Bornean | 18 | 100.0 | 100.0 | 100.0 |
|  | Sumatran | 0 | 0.0 | 0.0 | 100.0 |
|  | Missing | 0 | 0.0 |  |  |
|  | Total | 18 | 100.0 |  |  |
| Sabangau | Bornean | 9 | 100.0 | 100.0 | 100.0 |
|  | Sumatran | 0 | 0.0 | 0.0 | 100.0 |
|  | Missing | 0 | 0.0 |  |  |
|  | Total | 9 | 100.0 |  |  |
| Sampan Getek | Bornean | 0 | 0.0 | 0.0 | 0.0 |
|  | Sumatran | 7 | 100.0 | 100.0 | 100.0 |
|  | Missing | 0 | 0.0 |  |  |
|  | Total | 7 | 100.0 |  |  |
| Sikundur | Bornean | 0 | 0.0 | 0.0 | 0.0 |
|  | Sumatran | 13 | 100.0 | 100.0 | 100.0 |
|  | Missing | 0 | 0.0 |  |  |
|  | Total | 13 | 100.0 |  |  |
| Suaq | Bornean | 0 | 0.0 | 0.0 | 0.0 |
|  | Sumatran | 8 | 100.0 | 100.0 | 100.0 |
|  | Missing | 0 | 0.0 |  |  |
|  | Total | 8 | 100.0 |  |  |
| Tuanan | Bornean | 14 | 100.0 | 100.0 | 100.0 |
|  | Sumatran | 0 | 0.0 | 0.0 | 100.0 |
|  | Missing | 0 | 0.0 |  |  |
|  | Total | 14 | 100.0 |  |  |
|  | | | | | |
