# Supplementary material for: Sociality predicts orangutan vocal phenotype
Source: Nat Ecol Evol. 2022 Mar 21;6(5):644–52. doi: 10.1038/s41559-022-01689-z (PMC9085614; doi:10.1038/s41559-022-01689-z)
Supplement: Supplementary file 6 — Results of linear mixed models. [file 41559_2022_1689_MOESM6_ESM.html]

JASP 


# Results

## Linear Mixed Models, Emergence\_Duration

| ANOVA Summary | | | |
| --- | --- | --- | --- |
| Effect | df | F | p |
| Sex | 1, 52.69 | 0.209 | .649 |
| AgeSex class | 4, 50.15 | 0.986 | .424 |
| Context | 3, 7.46 | 3.013 | .099 |
| Species | 1, 46.80 | 1.621 | .209 |
| Population density | 1, 47.69 | 8.472 | .005 |
|  | | | |
|  |  |  |  |
| --- | --- | --- | --- |
| *Note.*  Model terms tested with Satterthwaite testMethod. | | | |
| *Note.*  The following variable is used as a random effects grouping factor: 'Individual'. | | | |
| *Note.*  Type III Sum of Squares | | | |

### Model summary

| Fit statistics | | | | |
| --- | --- | --- | --- | --- |
| Deviance (REML) | log Lik. | df | AIC | BIC |
| -11.57 | 5.785 | 13 | 14.43 | 43.47 |
|  | | | | |
|  |  |  |  |  |
| --- | --- | --- | --- | --- |
| *Note.*  The model was fitted using restricted maximum likelihood. Please note that models with different fixed effects cannot be compared when REML is used. To use ML, switch 'Test method' to 'Likelihood ratio tests'. | | | | |

| Sample sizes | |
| --- | --- |
|  | Levels of RE grouping factors |
| Observations | Individual |
| 69 | 60 |
|  | |

| Fixed Effects Estimates | | | | | |
| --- | --- | --- | --- | --- | --- |
| Term | Estimate | SE | df | t | p |
| Intercept | 0.557 | 0.071 | 39.748 | 7.868 | < .001 |
| Sex (1) | 0.034 | 0.074 | 52.692 | 0.458 | .649 |
| AgeSex class (1) | -0.107 | 0.083 | 51.736 | -1.284 | .205 |
| AgeSex class (2) | 0.015 | 0.079 | 51.517 | 0.194 | .847 |
| AgeSex class (3) | 0.044 | 0.089 | 52.029 | 0.494 | .623 |
| AgeSex class (4) | 0.026 | 0.063 | 52.748 | 0.415 | .680 |
| Context (1) | -0.150 | 0.076 | 7.447 | -1.985 | .085 |
| Context (2) | -0.057 | 0.053 | 7.869 | -1.080 | .312 |
| Context (3) | 0.170 | 0.107 | 5.280 | 1.588 | .170 |
| Species (1) | -0.033 | 0.026 | 46.796 | -1.273 | .209 |
| Population density | 0.067 | 0.023 | 47.690 | 2.911 | .005 |
|  | | | | | |
|  |  |  |  |  |  |
| --- | --- | --- | --- | --- | --- |
| *Note.*  The intercept corresponds to the (unweighted) grand mean; for each factor with k levels, k - 1 parameters are estimated with sum contrast coding. Consequently, the estimates cannot be directly mapped to factor levels. Use estimated marginal means for obtaining estimates for each factor level/design cell or their differences. | | | | | |

| Estimated Marginal Means | | | | |
| --- | --- | --- | --- | --- |
|  | | | 95% CI | |
| Species | Estimate | SE | Lower | Upper |
| Bornean | 0.679 | 0.053 | 0.575 | 0.783 |
| Sumatran | 0.745 | 0.057 | 0.633 | 0.856 |
|  | | | | |
|  |  |  |  |  |
| --- | --- | --- | --- | --- |
| *Note.*  Results are averaged over the levels of: Sex, AgeSex class, Context. | | | | |

## Linear Mixed Models, Self-organization\_Duration

| ANOVA Summary | | | |
| --- | --- | --- | --- |
| Effect | df | F | p |
| Sex | 1, 52.69 | 0.209 | .649 |
| AgeSex class | 4, 50.15 | 0.986 | .424 |
| Context | 3, 7.46 | 3.013 | .099 |
| Species | 1, 46.80 | 1.621 | .209 |
| Population density | 1, 47.69 | 8.472 | .005 |
|  | | | |
|  |  |  |  |
| --- | --- | --- | --- |
| Note: All random slopes involving ‘'Sex', 'AgeSex class', 'Species', 'Population density'’ have been removed for the random effects grouping factor ‘Individual’. -- Factors 'Sex', 'AgeSex class', 'Species', 'Population density' do not vary within the levels of random effects grouping factor 'Individual'. | | | |
| *Note.*  Model terms tested with Satterthwaite testMethod. | | | |
| Note: Random slope of ‘'Context'’ for the random effects grouping factor ‘Individual’ removed -- Too few observations to estimate random slope of ''Context'' for random effects grouping factor 'Individual'. | | | |
| *Note.*  The following variable is used as a random effects grouping factor: 'Individual'. | | | |
| *Note.*  Type III Sum of Squares | | | |

### Model summary

| Fit statistics | | | | |
| --- | --- | --- | --- | --- |
| Deviance (REML) | log Lik. | df | AIC | BIC |
| -11.57 | 5.785 | 13 | 14.43 | 43.47 |
|  | | | | |
|  |  |  |  |  |
| --- | --- | --- | --- | --- |
| *Note.*  The model was fitted using restricted maximum likelihood. Please note that models with different fixed effects cannot be compared when REML is used. To use ML, switch 'Test method' to 'Likelihood ratio tests'. | | | | |

| Sample sizes | |
| --- | --- |
|  | Levels of RE grouping factors |
| Observations | Individual |
| 69 | 60 |
|  | |

| Fixed Effects Estimates | | | | | |
| --- | --- | --- | --- | --- | --- |
| Term | Estimate | SE | df | t | p |
| Intercept | 0.443 | 0.071 | 39.748 | 6.251 | < .001 |
| Sex (1) | -0.034 | 0.074 | 52.692 | -0.458 | .649 |
| AgeSex class (1) | 0.107 | 0.083 | 51.736 | 1.284 | .205 |
| AgeSex class (2) | -0.015 | 0.079 | 51.517 | -0.194 | .847 |
| AgeSex class (3) | -0.044 | 0.089 | 52.029 | -0.494 | .623 |
| AgeSex class (4) | -0.026 | 0.063 | 52.748 | -0.415 | .680 |
| Context (1) | 0.150 | 0.076 | 7.447 | 1.985 | .085 |
| Context (2) | 0.057 | 0.053 | 7.869 | 1.080 | .312 |
| Context (3) | -0.170 | 0.107 | 5.280 | -1.588 | .170 |
| Species (1) | 0.033 | 0.026 | 46.796 | 1.273 | .209 |
| Population density | -0.067 | 0.023 | 47.690 | -2.911 | .005 |
|  | | | | | |
|  |  |  |  |  |  |
| --- | --- | --- | --- | --- | --- |
| *Note.*  The intercept corresponds to the (unweighted) grand mean; for each factor with k levels, k - 1 parameters are estimated with sum contrast coding. Consequently, the estimates cannot be directly mapped to factor levels. Use estimated marginal means for obtaining estimates for each factor level/design cell or their differences. | | | | | |

| Estimated Marginal Means | | | | |
| --- | --- | --- | --- | --- |
|  | | | 95% CI | |
| Species | Estimate | SE | Lower | Upper |
| Bornean | 0.321 | 0.053 | 0.217 | 0.425 |
| Sumatran | 0.255 | 0.057 | 0.144 | 0.367 |
|  | | | | |
|  |  |  |  |  |
| --- | --- | --- | --- | --- |
| *Note.*  Results are averaged over the levels of: Sex, AgeSex class, Context. | | | | |

## Linear Mixed Models, Complexity\_Duration

| ANOVA Summary | | | |
| --- | --- | --- | --- |
| Effect | df | F | p |
| Sex | 1, 56.87 | 1.586 | .213 |
| AgeSex class | 4, 52.10 | 1.161 | .339 |
| Context | 3, 20.62 | 3.117 | .048 |
| Species | 1, 45.37 | 0.548 | .463 |
| Population density | 1, 47.60 | 4.989 | .030 |
|  | | | |
|  |  |  |  |
| --- | --- | --- | --- |
| *Note.*  Model terms tested with Satterthwaite testMethod. | | | |
| *Note.*  The following variable is used as a random effects grouping factor: 'Individual'. | | | |
| *Note.*  Type III Sum of Squares | | | |

### Model summary

| Fit statistics | | | | |
| --- | --- | --- | --- | --- |
| Deviance (REML) | log Lik. | df | AIC | BIC |
| 19.18 | -9.590 | 13 | 45.18 | 74.22 |
|  | | | | |
|  |  |  |  |  |
| --- | --- | --- | --- | --- |
| *Note.*  The model was fitted using restricted maximum likelihood. Please note that models with different fixed effects cannot be compared when REML is used. To use ML, switch 'Test method' to 'Likelihood ratio tests'. | | | | |

| Sample sizes | |
| --- | --- |
|  | Levels of RE grouping factors |
| Observations | Individual |
| 69 | 60 |
|  | |

| Fixed Effects Estimates | | | | | |
| --- | --- | --- | --- | --- | --- |
| Term | Estimate | SE | df | t | p |
| Intercept | 0.811 | 0.096 | 54.27 | 8.458 | < .001 |
| Sex (1) | -0.119 | 0.095 | 56.87 | -1.259 | .213 |
| AgeSex class (1) | 0.179 | 0.107 | 55.60 | 1.676 | .099 |
| AgeSex class (2) | 0.108 | 0.101 | 54.83 | 1.069 | .290 |
| AgeSex class (3) | -0.175 | 0.114 | 56.02 | -1.538 | .130 |
| AgeSex class (4) | -0.044 | 0.081 | 56.74 | -0.534 | .595 |
| Context (1) | 0.245 | 0.113 | 23.99 | 2.174 | .040 |
| Context (2) | 0.105 | 0.080 | 18.42 | 1.319 | .203 |
| Context (3) | -0.325 | 0.166 | 14.45 | -1.960 | .070 |
| Species (1) | 0.024 | 0.032 | 45.37 | 0.740 | .463 |
| Population density | -0.065 | 0.029 | 47.60 | -2.234 | .030 |
|  | | | | | |
|  |  |  |  |  |  |
| --- | --- | --- | --- | --- | --- |
| *Note.*  The intercept corresponds to the (unweighted) grand mean; for each factor with k levels, k - 1 parameters are estimated with sum contrast coding. Consequently, the estimates cannot be directly mapped to factor levels. Use estimated marginal means for obtaining estimates for each factor level/design cell or their differences. | | | | | |

| Estimated Marginal Means | | | | |
| --- | --- | --- | --- | --- |
|  | | | 95% CI | |
| Species | Estimate | SE | Lower | Upper |
| Bornean | 0.685 | 0.076 | 0.536 | 0.835 |
| Sumatran | 0.637 | 0.079 | 0.482 | 0.793 |
|  | | | | |
|  |  |  |  |  |
| --- | --- | --- | --- | --- |
| *Note.*  Results are averaged over the levels of: Sex, AgeSex class, Context. | | | | |

## Linear Mixed Models, Emergence\_Max Frequency

| ANOVA Summary | | | |
| --- | --- | --- | --- |
| Effect | df | F | p |
| Sex | 1, 58 | 3.014 | .088 |
| AgeSex class | 4, 58 | 0.702 | .594 |
| Context | 3, 58 | 0.765 | .518 |
| Species | 1, 58 | 0.215 | .644 |
| Population density | 1, 58 | 0.321 | .573 |
|  | | | |
|  |  |  |  |
| --- | --- | --- | --- |
| Warning: Model fit is singular. Specified random effects parameters (random intercepts and random slopes) cannot be estimated from the available data. Carefully reduce the random effects structure, but this practice might inflate the reported p-value, and invalidates the analysis. | | | |
| *Note.*  Model terms tested with Satterthwaite testMethod. | | | |
| *Note.*  The following variable is used as a random effects grouping factor: 'Individual'. | | | |
| *Note.*  Type III Sum of Squares | | | |

### Model summary

| Fit statistics | | | | |
| --- | --- | --- | --- | --- |
| Deviance (REML) | log Lik. | df | AIC | BIC |
| -10.31 | 5.153 | 13 | 15.69 | 44.74 |
|  | | | | |
|  |  |  |  |  |
| --- | --- | --- | --- | --- |
| *Note.*  The model was fitted using restricted maximum likelihood. Please note that models with different fixed effects cannot be compared when REML is used. To use ML, switch 'Test method' to 'Likelihood ratio tests'. | | | | |

| Sample sizes | |
| --- | --- |
|  | Levels of RE grouping factors |
| Observations | Individual |
| 69 | 60 |
|  | |

| Fixed Effects Estimates | | | | | |
| --- | --- | --- | --- | --- | --- |
| Term | Estimate | SE | df | t | p |
| Intercept | 0.674 | 0.074 | 58.00 | 9.051 | < .001 |
| Sex (1) | 0.127 | 0.073 | 58.00 | 1.736 | .088 |
| AgeSex class (1) | -0.102 | 0.082 | 58.00 | -1.236 | .221 |
| AgeSex class (2) | -0.095 | 0.078 | 58.00 | -1.223 | .226 |
| AgeSex class (3) | 0.125 | 0.088 | 58.00 | 1.420 | .161 |
| AgeSex class (4) | -0.072 | 0.063 | 58.00 | -1.149 | .255 |
| Context (1) | -0.089 | 0.089 | 58.00 | -1.000 | .322 |
| Context (2) | -0.050 | 0.064 | 58.00 | -0.784 | .436 |
| Context (3) | 0.133 | 0.133 | 58.00 | 0.998 | .323 |
| Species (1) | 0.011 | 0.025 | 58.00 | 0.464 | .644 |
| Population density | -0.013 | 0.022 | 58.00 | -0.566 | .573 |
|  | | | | | |
|  |  |  |  |  |  |
| --- | --- | --- | --- | --- | --- |
| *Note.*  The intercept corresponds to the (unweighted) grand mean; for each factor with k levels, k - 1 parameters are estimated with sum contrast coding. Consequently, the estimates cannot be directly mapped to factor levels. Use estimated marginal means for obtaining estimates for each factor level/design cell or their differences. | | | | | |

| Estimated Marginal Means | | | | |
| --- | --- | --- | --- | --- |
|  | | | 95% CI | |
| Species | Estimate | SE | Lower | Upper |
| Bornean | 0.656 | 0.060 | 0.538 | 0.774 |
| Sumatran | 0.633 | 0.062 | 0.512 | 0.754 |
|  | | | | |
|  |  |  |  |  |
| --- | --- | --- | --- | --- |
| *Note.*  Results are averaged over the levels of: Sex, AgeSex class, Context. | | | | |

## Linear Mixed Models, Self-organization\_Max Frequency

| ANOVA Summary | | | |
| --- | --- | --- | --- |
| Effect | df | F | p |
| Sex | 1, 58 | 3.014 | .088 |
| AgeSex class | 4, 58 | 0.702 | .594 |
| Context | 3, 58 | 0.765 | .518 |
| Species | 1, 58 | 0.215 | .644 |
| Population density | 1, 58 | 0.321 | .573 |
|  | | | |
|  |  |  |  |
| --- | --- | --- | --- |
| Note: All random slopes involving ‘'Sex', 'AgeSex class', 'Species', 'Population density'’ have been removed for the random effects grouping factor ‘Individual’. -- Factors 'Sex', 'AgeSex class', 'Species', 'Population density' do not vary within the levels of random effects grouping factor 'Individual'. | | | |
| Warning: Model fit is singular. Specified random effects parameters (random intercepts and random slopes) cannot be estimated from the available data. Carefully reduce the random effects structure, but this practice might inflate the reported p-value, and invalidates the analysis. | | | |
| *Note.*  Model terms tested with Satterthwaite testMethod. | | | |
| Note: Random slope of ‘'Context'’ for the random effects grouping factor ‘Individual’ removed -- Too few observations to estimate random slope of ''Context'' for random effects grouping factor 'Individual'. | | | |
| *Note.*  The following variable is used as a random effects grouping factor: 'Individual'. | | | |
| *Note.*  Type III Sum of Squares | | | |

### Model summary

| Fit statistics | | | | |
| --- | --- | --- | --- | --- |
| Deviance (REML) | log Lik. | df | AIC | BIC |
| -10.31 | 5.153 | 13 | 15.69 | 44.74 |
|  | | | | |
|  |  |  |  |  |
| --- | --- | --- | --- | --- |
| *Note.*  The model was fitted using restricted maximum likelihood. Please note that models with different fixed effects cannot be compared when REML is used. To use ML, switch 'Test method' to 'Likelihood ratio tests'. | | | | |

| Sample sizes | |
| --- | --- |
|  | Levels of RE grouping factors |
| Observations | Individual |
| 69 | 60 |
|  | |

| Fixed Effects Estimates | | | | | |
| --- | --- | --- | --- | --- | --- |
| Term | Estimate | SE | df | t | p |
| Intercept | 0.326 | 0.074 | 58.00 | 4.386 | < .001 |
| Sex (1) | -0.127 | 0.073 | 58.00 | -1.736 | .088 |
| AgeSex class (1) | 0.102 | 0.082 | 58.00 | 1.236 | .221 |
| AgeSex class (2) | 0.095 | 0.078 | 58.00 | 1.223 | .226 |
| AgeSex class (3) | -0.125 | 0.088 | 58.00 | -1.420 | .161 |
| AgeSex class (4) | 0.072 | 0.063 | 58.00 | 1.149 | .255 |
| Context (1) | 0.089 | 0.089 | 58.00 | 1.000 | .322 |
| Context (2) | 0.050 | 0.064 | 58.00 | 0.784 | .436 |
| Context (3) | -0.133 | 0.133 | 58.00 | -0.998 | .323 |
| Species (1) | -0.011 | 0.025 | 58.00 | -0.464 | .644 |
| Population density | 0.013 | 0.022 | 58.00 | 0.566 | .573 |
|  | | | | | |
|  |  |  |  |  |  |
| --- | --- | --- | --- | --- | --- |
| *Note.*  The intercept corresponds to the (unweighted) grand mean; for each factor with k levels, k - 1 parameters are estimated with sum contrast coding. Consequently, the estimates cannot be directly mapped to factor levels. Use estimated marginal means for obtaining estimates for each factor level/design cell or their differences. | | | | | |

| Estimated Marginal Means | | | | |
| --- | --- | --- | --- | --- |
|  | | | 95% CI | |
| Species | Estimate | SE | Lower | Upper |
| Bornean | 0.344 | 0.060 | 0.226 | 0.462 |
| Sumatran | 0.367 | 0.062 | 0.246 | 0.488 |
|  | | | | |
|  |  |  |  |  |
| --- | --- | --- | --- | --- |
| *Note.*  Results are averaged over the levels of: Sex, AgeSex class, Context. | | | | |

## Linear Mixed Models, Complexity\_Max Frequency

| ANOVA Summary | | | |
| --- | --- | --- | --- |
| Effect | df | F | p |
| Sex | 1, 55.65 | 1.049 | .310 |
| AgeSex class | 4, 54.37 | 0.971 | .431 |
| Context | 3, 15.30 | 1.597 | .231 |
| Species | 1, 52.56 | 0.501 | .482 |
| Population density | 1, 53.06 | 0.009 | .927 |
|  | | | |
|  |  |  |  |
| --- | --- | --- | --- |
| *Note.*  Model terms tested with Satterthwaite testMethod. | | | |
| *Note.*  The following variable is used as a random effects grouping factor: 'Individual'. | | | |
| *Note.*  Type III Sum of Squares | | | |

### Model summary

| Fit statistics | | | | |
| --- | --- | --- | --- | --- |
| Deviance (REML) | log Lik. | df | AIC | BIC |
| 1.579 | -0.790 | 13 | 27.58 | 56.62 |
|  | | | | |
|  |  |  |  |  |
| --- | --- | --- | --- | --- |
| *Note.*  The model was fitted using restricted maximum likelihood. Please note that models with different fixed effects cannot be compared when REML is used. To use ML, switch 'Test method' to 'Likelihood ratio tests'. | | | | |

| Sample sizes | |
| --- | --- |
|  | Levels of RE grouping factors |
| Observations | Individual |
| 69 | 60 |
|  | |

| Fixed Effects Estimates | | | | | |
| --- | --- | --- | --- | --- | --- |
| Term | Estimate | SE | df | t | p |
| Intercept | 0.759 | 0.079 | 48.41 | 9.554 | < .001 |
| Sex (1) | -0.085 | 0.083 | 55.65 | -1.024 | .310 |
| AgeSex class (1) | 0.113 | 0.093 | 55.18 | 1.210 | .231 |
| AgeSex class (2) | -0.009 | 0.089 | 55.06 | -0.106 | .916 |
| AgeSex class (3) | -0.087 | 0.099 | 55.33 | -0.874 | .386 |
| AgeSex class (4) | 0.061 | 0.071 | 55.68 | 0.855 | .396 |
| Context (1) | 0.077 | 0.085 | 15.08 | 0.904 | .380 |
| Context (2) | 0.107 | 0.060 | 15.73 | 1.787 | .093 |
| Context (3) | -0.232 | 0.121 | 11.11 | -1.926 | .080 |
| Species (1) | -0.020 | 0.029 | 52.56 | -0.708 | .482 |
| Population density | 0.002 | 0.026 | 53.06 | 0.093 | .927 |
|  | | | | | |
|  |  |  |  |  |  |
| --- | --- | --- | --- | --- | --- |
| *Note.*  The intercept corresponds to the (unweighted) grand mean; for each factor with k levels, k - 1 parameters are estimated with sum contrast coding. Consequently, the estimates cannot be directly mapped to factor levels. Use estimated marginal means for obtaining estimates for each factor level/design cell or their differences. | | | | | |

| Estimated Marginal Means | | | | |
| --- | --- | --- | --- | --- |
|  | | | 95% CI | |
| Species | Estimate | SE | Lower | Upper |
| Bornean | 0.744 | 0.060 | 0.628 | 0.861 |
| Sumatran | 0.785 | 0.064 | 0.659 | 0.911 |
|  | | | | |
|  |  |  |  |  |
| --- | --- | --- | --- | --- |
| *Note.*  Results are averaged over the levels of: Sex, AgeSex class, Context. | | | | |
