# Supplementary material for: Sociality predicts orangutan vocal phenotype
Source: Nat Ecol Evol. 2022 Mar 21;6(5):644–52. doi: 10.1038/s41559-022-01689-z (PMC9085614; doi:10.1038/s41559-022-01689-z)
Supplement: Supplementary file 7 — R code notebook for entropic calculation. [file 41559_2022_1689_MOESM7_ESM.html]

Sociality predicts orangutan vocal phenotype


# Sociality predicts orangutan vocal phenotype

#### G. Santamaria-Bonfil

# 1 Libraries required

First, the required libraries must be loaded. These are standard
`R` packages such as `data.table`,
`dplyr`, `ggplot1`, `reshape2`, and so
on.

```
## 
## Attaching package: 'dplyr'
```

```
## The following objects are masked from 'package:data.table':
## 
##     between, first, last
```

```
## The following objects are masked from 'package:stats':
## 
##     filter, lag
```

```
## The following objects are masked from 'package:base':
## 
##     intersect, setdiff, setequal, union
```

```
## 
## Attaching package: 'reshape2'
```

```
## The following objects are masked from 'package:data.table':
## 
##     dcast, melt
```

# 2 Entropy-based Emergence, Self-Organization, and Complexity measures

Afterwards `source` in the `R` environment the
entropy-based **complexity** measures, namely *Emergence
(E)*, *Self-Organization (S)*, and *Complexity (C)*
(in short *ESC*). As part of this notebook, the corresponding
*ESC* measures are provided.

To fully grasp the idea behind the *ESC* measures, lets show
them with an example. Consider a phenomenon (such as a coin) that can be
described by the Binomial distribution, that is defined as follows:

\[ Pr(k;n,p) = Pr(X = H) = {{n}\choose{k}}
\cdot p^kq^{n-k},\] where \(k\)
successes occur with probability \(p^k\) and \(n −
k\) failures occur with probability \(q
= (1 − p)^{n − k}\); the \(k\)
successes can occur anywhere among the \(n\) trials, and there are \({{n}\choose{k}}\) different ways of
distributing \(k\) successes in a
sequence of \(n\) trials.

## 2.1 E = 1

Suppose a non-biased coin, where the probability of getting a HEAD
(H) or a TAIL (T) is the same, i.e. Pr(H=T)=0.5. In this case, the
distribution that describes the coin behavior corresponds to a uniform
distribution. Consequently, the emergence of the system is maximal, with
zero self-organization and complexity.

```
set.seed(123)
tmp.dist <- rbinom(n = 1000000,size = 1,prob = 0.5)
tmp.dist.df <- data.frame(p.event = tmp.dist)
tmp.dist.df$Event <- ifelse(tmp.dist.df$p.event ==1, 'blue','red')
ggplot(tmp.dist.df, aes(x=p.event, fill=Event))+geom_bar()+
  scale_fill_discrete(name = "Head or Tail?", labels = c("H", "T"))+theme_bw()
```

```
complexity.discrete(tmp.dist,no_states = 2)
```

## 2.2 S = 1

Suppose a biased coin, where the probability of getting a HEAD (H) is
Pr(H)=1 and for TAIL (T) Pr(T) = 1-Pr(H)=0. In this case, the
distribution that describes the coin behavior corresponds to a Dirac
delta distribution. Consequently, the self-organization of the system is
maximal, with zero emergence and complexity.

```
set.seed(123)
tmp.dist <- rbinom(n = 1000000,size = 1,prob = 0)
tmp.dist.df <- data.frame(p.event = tmp.dist)
tmp.dist.df$Event <- ifelse(tmp.dist.df$p.event ==1, 'blue','red')
ggplot(tmp.dist.df, aes(x=p.event, fill=Event))+geom_bar()+
  scale_fill_discrete(name = "Head or Tail?", labels = c("H", "T"))+theme_bw()
```

```
complexity.discrete(tmp.dist,no_states = 2)
```

## 2.3 A balance between E and S: C

Now suppose a biased coin which is being manipulated such that, it is
getting biased from displaying always \(H\) (i.e. \(p(H)=1; p(T)=0\)) towards displaying always
\(T\) (i.e. \(p(H)=0; p(T)=1\)) by increments of \(0.001\) (e.g. \(p(H)=0.999; p(T)=0.001\), \(p(H)=0.998; p(T)=0.002\), \(p(H)=0.997; p(T)=0.003\), and so on). The
resulting \(E, S, C\) measures for such
distributions will be depicted by the following figure.

In this sense the complexity is maximal, i.e. \(C=1\), when one of the states (either H or
T) has a higher probability of occurrence, still the probability of
occurrence for the opposite event is not zero.

```
no_states <- 2
p.event   <- seq(0.001,0.999,by=0.001)
kConst    <- 1/log2(no_states)

esc.binDist           <- data.frame(p_x = p.event, 
                                    p_y=1-p.event)

for(i in 1:dim(esc.binDist)[1])
{
  entrop <- Entropy(esc.binDist[i,],nonNAidxes = 1:2)
  
  emergence         <- kConst*entrop
  selfOrganization  <- 1 - emergence
  complexity        <- 4 * emergence * selfOrganization
  
  esc.binDist$E[i] <- emergence
  esc.binDist$S[i] <- selfOrganization
  esc.binDist$C[i] <- complexity
}

esc.binDist.mlt <- melt(esc.binDist,id.vars=c('p_x','p_y'))


ggplot(esc.binDist.mlt, aes(x=p_x, y = value,color = variable))+
  geom_line(size = 1.2)+theme_bw()+labs(color = 'Measures')
```

```
## Warning: Using `size` aesthetic for lines was deprecated in ggplot2 3.4.0.
## ℹ Please use `linewidth` instead.
## This warning is displayed once every 8 hours.
## Call `lifecycle::last_lifecycle_warnings()` to see where this warning was
## generated.
```

# 3 Load orangutan vocal production data

# 4 Estimate ESC measures for the vocal production of each orangutan without considering context

```
orangutans.ids <- sort(unique(orangutan.data$individual), na.last = T) %>% 
                  as.character()
orangutans.ctx <- sort(unique(orangutan.data$context), na.last = T) %>% 
                  as.character()

orangutans.maxF  <- data.frame(E = numeric(), 
                            S = numeric(), 
                            C = numeric(), 
                            mky.id = numeric(),
                            k.id = numeric(),
                            AgeSex = character(),
                            gender  = character(),
                            pop = character(),
                            context = character())

orangutans.duration<- data.frame(E = numeric(), 
                              S = numeric(), 
                              C = numeric(), 
                              mky.id = numeric(),
                              k.id = numeric(),
                              AgeSex = character(),
                              gender  = character(),
                              pop = character(),
                              context = character())

alph.size<- 10

for(j in 1:length(orangutans.ids))
{
  if(is.na(orangutans.ids[j]))
  {
    # indv.data <- filter(orangutan.data, is.na(context) )
    indv.data <- filter(orangutan.data, is.na(individual) )
  }else
  {
    indv.data <- filter(orangutan.data, 
                  # context == orangutans.ids[j])
                  individual == orangutans.ids[j])
  }
  
  res.maxF  <- data.frame(E = numeric(), 
                          S = numeric(), 
                          C = numeric(), 
                          mky.id = numeric(),
                          k.id = numeric(),
                          AgeSex = character(),
                          gender  = character(),
                          pop = character(),
                          context = character())
  
  res.duration<- data.frame(E = numeric(), 
                            S = numeric(), 
                            C = numeric(), 
                            mky.id = numeric(),
                            k.id = numeric(),
                            AgeSex = character(),
                            gender  = character(),
                            pop = character(),
                            context = character())
  
  res.maxF <- rbindlist(list(res.maxF, 
                             as.list(complexity.discrete(indv.data$maxFreq, 
                                                         alph.size)[1:3])),
                        use.names = T, fill = T)
  
  res.duration <- rbindlist(list(res.duration, 
                                 as.list(complexity.discrete(indv.data$duration, 
                                                             alph.size)[1:3])),
                            use.names = T, fill = T)
  
  res.maxF$mky.id <- as.character(orangutans.ids[j])
  res.maxF$k.id   <- alph.size
  res.maxF$AgeSex <- indv.data$`age-sex class`[1]
  res.maxF$gender <- indv.data$gender[1]
  res.maxF$pop    <- indv.data$population[1]
  res.maxF$context<- indv.data$context[1]
  
  res.duration$mky.id <- as.character(orangutans.ids[j])
  res.duration$k.id   <- alph.size
  res.duration$AgeSex <- indv.data$`age-sex class`[1]
  res.duration$gender <- indv.data$gender[1]
  res.duration$pop    <- indv.data$population[1]
  res.duration$context<- indv.data$context[1]
  
  orangutans.maxF    <- rbindlist(list(orangutans.maxF,
                                       as.list(res.maxF)))

  orangutans.duration  <- rbindlist(list(orangutans.duration,
                                         as.list(res.duration)))
  
}
```

## 4.1 Max frequency

### 4.1.1 Example for max. frequency of the individual `Brutus`

```
no_states   <- 10 
tmpIndv.data<- filter(orangutan.data, individual=='Brutus')
pmf.Len     <-dim(tmpIndv.data)[1]

margSttProb <- table(cut(tmpIndv.data$maxFreq, breaks = no_states))/pmf.Len
```

```
complexity.discrete(tmpIndv.data$maxFreq,no_states = 10)[1:3]
```

## 4.2 Duration

```
dur.melt <- melt(orangutans.duration, 
                  id.vars = c('mky.id', 'pop'), 
                  measure.vars = c('E', 'S', 'C'))
dur.melt <- dur.melt %>%
  mutate(mky.id = factor(mky.id),
         pop = factor(pop))

print(
  ggplot(dur.melt, 
         aes(x= variable, y = value, color = pop))+
    ggrepel::geom_text_repel(aes(label = mky.id), 
                             color = "black", force = 1,
                             size = 3, max.overlaps = Inf,
                             segment.color = "grey")+
    geom_point(aes(shape = pop)) + 
    guides(#color = 'none', fill = "none",  
           shape = guide_legend("Population", override.aes = list(size=4)))+
    theme_bw()+
    theme(axis.text=element_text(size=12, face = 'bold'),
          title = element_text(size=18))+
    scale_y_continuous(breaks = seq(0,1,by=0.1), labels =paste(seq(0,1,by=0.1)))+
    labs(title = "Duration by Orangutan",
         color  = "Population", linetype = "Population", shape = "Population",
         x = "Complexity Measures",
         y = "")
)
```

### 4.2.1 Example for duration of the individual `Brutus`

```
no_states   <- 10 
tmpIndv.data<- filter(orangutan.data, individual=='Brutus')
pmf.Len     <-dim(tmpIndv.data)[1]

margSttProb <- table(cut(tmpIndv.data$duration, breaks = no_states))/pmf.Len
```

```
complexity.discrete(tmpIndv.data$maxFreq,no_states = 10)[1:3]
```

```
margStt.melt <- melt(margSttProb)
print(
ggplot(margStt.melt, aes(x=value)) + 
      geom_histogram(color="black", alpha=0.5, fill="red", bins = no_states)+
      geom_density(alpha=.5, fill="#FF6666") + theme_classic()
)
```

## 4.3 ESC distributions for the whole orangutans sample

### 4.3.1 By max. frequency

```
ggplot(orangutans.maxF, aes(x=E)) + 
  geom_histogram(color="black", alpha=0.5, fill="red")+
  geom_density(alpha=.5, fill="#FF6666") + theme_classic()
```

```
## `stat_bin()` using `bins = 30`. Pick better value with `binwidth`.
```

```
ggplot(orangutans.maxF, aes(x=S)) + 
  geom_histogram(color="black", alpha=0.5, fill="blue")+
  geom_density(alpha=.5, fill="#008080") + theme_classic()
```

```
## `stat_bin()` using `bins = 30`. Pick better value with `binwidth`.
```

```
ggplot(orangutans.maxF, aes(x=C)) + 
  geom_histogram(color="black", alpha=0.5, fill="green")+
  geom_density(alpha=.5, fill="#00FFFF") + theme_classic()
```

```
## `stat_bin()` using `bins = 30`. Pick better value with `binwidth`.
```

### 4.3.2 By calls duration

```
ggplot(orangutans.duration, aes(x=E)) + 
  geom_histogram(color="black", fill="red")+
  geom_density(alpha=.5, fill="#FF6666") + theme_classic()
```

```
## `stat_bin()` using `bins = 30`. Pick better value with `binwidth`.
```

```
ggplot(orangutans.duration, aes(x=S)) + 
  geom_histogram(color="black", fill="blue")+
  geom_density(alpha=.5, fill="#008080") + theme_classic()
```

```
## `stat_bin()` using `bins = 30`. Pick better value with `binwidth`.
```

```
ggplot(orangutans.duration, aes(x=C)) + 
  geom_histogram(color="black", fill="green")+
  geom_density(alpha=.5, fill="#00FFFF") + theme_classic()
```

```
## `stat_bin()` using `bins = 30`. Pick better value with `binwidth`.
```

# 5 Estimate ESC measures for the vocal production of each orangutan considering context

Process for estimating ESC measures

```
orangutans.ids <- sort(unique(orangutan.data$individual), na.last = T) %>% 
                  as.character()
orangutans.ctx <- sort(unique(orangutan.data$context), na.last = T) %>% 
                  as.character()

orangutans.maxF  <- data.frame(E = numeric(), 
                            S = numeric(), 
                            C = numeric(), 
                            mky.id = numeric(),
                            k.id = numeric(),
                            AgeSex = character(),
                            gender  = character(),
                            pop = character(),
                            context = character())

orangutans.duration<- data.frame(E = numeric(), 
                              S = numeric(), 
                              C = numeric(), 
                              mky.id = numeric(),
                              k.id = numeric(),
                              AgeSex = character(),
                              gender  = character(),
                              pop = character(),
                              context = character())

alph.size<- 10

orangutan.grid <- expand.grid(orangutans.ids,orangutans.ctx)
colnames(orangutan.grid) <- c('id','context')

for(j in 1:dim(orangutan.grid)[1])
{
  if(is.na(orangutan.grid$id[j])||is.na(orangutan.grid$context[j]))
  {
    if(is.na(orangutan.grid$id[j])&&is.na(orangutan.grid$context[j]))
    {
      indv.data <- filter(orangutan.data, 
                          is.na(individual), is.na(context))
    }
    else if(is.na(orangutan.grid$id[j]))
    {
      indv.data <- filter(orangutan.data, is.na(individual), 
                          context == orangutan.grid$context[j])
    }
    else
    {
      indv.data <- filter(orangutan.data, 
                          individual == orangutan.grid$id[j], 
                          is.na(context))
    }
  }else
  {
    indv.data <- filter(orangutan.data, 
                        individual == orangutan.grid$id[j],
                        context == orangutan.grid$context[j])
  }
  
  if(dim(indv.data)[1]==0)
  {
    next
  }
  res.maxF  <- data.frame(E = numeric(), 
                          S = numeric(), 
                          C = numeric(), 
                          mky.id = numeric(),
                          k.id = numeric(),
                          AgeSex = character(),
                          gender  = character(),
                          pop = character(),
                          context = character())
  
  res.duration<- data.frame(E = numeric(), 
                            S = numeric(), 
                            C = numeric(), 
                            mky.id = numeric(),
                            k.id = numeric(),
                            AgeSex = character(),
                            gender  = character(),
                            pop = character(),
                            context = character())
  
  res.maxF <- rbindlist(list(res.maxF, 
                             as.list(complexity.discrete(indv.data$maxFreq, 
                                                         alph.size)[1:3])),
                        use.names = T, fill = T)
  
  res.duration <- rbindlist(list(res.duration, 
                                 as.list(complexity.discrete(indv.data$duration, 
                                                             alph.size)[1:3])),
                            use.names = T, fill = T)
  
  res.maxF$mky.id <- as.character(orangutan.grid$id[j])
  res.maxF$k.id   <- alph.size
  res.maxF$AgeSex <- indv.data$`age-sex class`[1]
  res.maxF$gender <- indv.data$gender[1]
  res.maxF$pop    <- indv.data$population[1]
  res.maxF$context<- indv.data$context[1]
  
  res.duration$mky.id <- as.character(orangutan.grid$id[j])
  res.duration$k.id   <- alph.size
  res.duration$AgeSex <- indv.data$`age-sex class`[1]
  res.duration$gender <- indv.data$gender[1]
  res.duration$pop    <- indv.data$population[1]
  res.duration$context<- indv.data$context[1]
  
  orangutans.maxF    <- rbindlist(list(orangutans.maxF,
                                       as.list(res.maxF)))

  orangutans.duration  <- rbindlist(list(orangutans.duration,
                                         as.list(res.duration)))
  
}
```

## 5.1 Max frequency


### 5.1.1 Example for max. frequency of the individual `Brutus`

#### 5.1.1.1 `context == 1`

```
no_states   <- 10 
tmpIndv.data<- filter(orangutan.data, individual=='Brutus',  context=='1')
pmf.Len     <- dim(tmpIndv.data)[1]

margSttProb <- table(cut(tmpIndv.data$maxFreq, breaks = no_states))/pmf.Len
```

```
complexity.discrete(tmpIndv.data$maxFreq,no_states = 10)[1:3]
```

#### 5.1.1.2 `context == 2`

```
no_states   <- 10 
tmpIndv.data<- filter(orangutan.data, individual=='Brutus',  context=='2')
pmf.Len     <- dim(tmpIndv.data)[1]

margSttProb <- table(cut(tmpIndv.data$maxFreq, breaks = no_states))/pmf.Len
```

```
complexity.discrete(tmpIndv.data$maxFreq,no_states = 10)[1:3]
```

## 5.2 Duration

```
dur.melt <- melt(orangutans.duration, 
                  id.vars = c('mky.id', 'pop'), 
                  measure.vars = c('E', 'S', 'C'))
dur.melt <- dur.melt %>%
  mutate(mky.id = factor(mky.id),
         pop = factor(pop))

print(
  ggplot(dur.melt, 
         aes(x= variable, y = value, color = pop))+
    ggrepel::geom_text_repel(aes(label = mky.id), 
                             color = "black", force = 1,
                             size = 3, max.overlaps = Inf,
                             segment.color = "grey")+
    geom_point(aes(shape = pop)) + 
    guides(#color = 'none', fill = "none",  
           shape = guide_legend("Population", override.aes = list(size=4)))+
    theme_bw()+
    theme(axis.text=element_text(size=12, face = 'bold'),
          title = element_text(size=18))+
    scale_y_continuous(breaks = seq(0,1,by=0.1), labels =paste(seq(0,1,by=0.1)))+
    labs(title = "Duration by Orangutan",
         color  = "Population", linetype = "Population", shape = "Population",
         x = "Complexity Measures",
         y = "")
)
```


### 5.2.1 Example for duration of the individual `Brutus`

#### 5.2.1.1 `Context == '1'`

```
no_states   <- 10 
tmpIndv.data<- filter(orangutan.data, individual=='Brutus', context=='1')
pmf.Len     <-dim(tmpIndv.data)[1]

margSttProb <- table(cut(tmpIndv.data$duration, breaks = no_states))/pmf.Len
```

```
complexity.discrete(tmpIndv.data$duration,no_states = 10)[1:3]
```

```
margStt.melt <- melt(margSttProb)
print(
ggplot(margStt.melt, aes(x=value)) + 
      geom_histogram(color="black", alpha=0.5, fill="red", bins = no_states)+
      geom_density(alpha=.5, fill="#FF6666") + theme_classic()
)
```

#### 5.2.1.2 `Context == '2'`

```
no_states   <- 10 
tmpIndv.data<- filter(orangutan.data, individual=='Brutus', context=='2')
pmf.Len     <-dim(tmpIndv.data)[1]

margSttProb <- table(cut(tmpIndv.data$duration, breaks = no_states))/pmf.Len
```

```
complexity.discrete(tmpIndv.data$duration,no_states = 10)[1:3]
```

```
margStt.melt <- melt(margSttProb)
print(
ggplot(margStt.melt, aes(x=value)) + 
      geom_histogram(color="black", alpha=0.5, fill="red", bins = no_states)+
      geom_density(alpha=.5, fill="#FF6666") + theme_classic()
)
```

## 5.3 ESC distributions for the whole orangutans sample

### 5.3.1 By max. frequency

```
ggplot(orangutans.maxF, aes(x=E)) + 
  geom_histogram(color="black", alpha=0.5, fill="red")+
  geom_density(alpha=.5, fill="#FF6666") + theme_classic()
```

```
## `stat_bin()` using `bins = 30`. Pick better value with `binwidth`.
```

```
ggplot(orangutans.maxF, aes(x=S)) + 
  geom_histogram(color="black", alpha=0.5, fill="blue")+
  geom_density(alpha=.5, fill="#008080") + theme_classic()
```

```
## `stat_bin()` using `bins = 30`. Pick better value with `binwidth`.
```

```
ggplot(orangutans.maxF, aes(x=C)) + 
  geom_histogram(color="black", alpha=0.5, fill="green")+
  geom_density(alpha=.5, fill="#00FFFF") + theme_classic()
```

```
## `stat_bin()` using `bins = 30`. Pick better value with `binwidth`.
```

### 5.3.2 By calls duration

```
ggplot(orangutans.duration, aes(x=E)) + 
  geom_histogram(color="black", fill="red")+
  geom_density(alpha=.5, fill="#FF6666") + theme_classic()
```

```
## `stat_bin()` using `bins = 30`. Pick better value with `binwidth`.
```

```
ggplot(orangutans.duration, aes(x=S)) + 
  geom_histogram(color="black", fill="blue")+
  geom_density(alpha=.5, fill="#008080") + theme_classic()
```

```
## `stat_bin()` using `bins = 30`. Pick better value with `binwidth`.
```

```
ggplot(orangutans.duration, aes(x=C)) + 
  geom_histogram(color="black", fill="green")+
  geom_density(alpha=.5, fill="#00FFFF") + theme_classic()
```

```
## `stat_bin()` using `bins = 30`. Pick better value with `binwidth`.
```
