# Supplementary material for: Sociality predicts orangutan vocal phenotype
Source: Nat Ecol Evol. 2022 Mar 21;6(5):644–52. doi: 10.1038/s41559-022-01689-z (PMC9085614; doi:10.1038/s41559-022-01689-z)
Supplement: Supplementary file 2 — Peer Review Information [file 41559_2022_1689_MOESM2_ESM.pdf]

Peer Review Information

Journal: Nature Ecology & Evolution
Manuscript Title: Sociality predicts orangutan vocal phenotype
Corresponding author name(s): Adriano R. Lameira

Editorial Notes:

Reviewer Comments & Decisions:

Decision Letter, initial version:
20th September 2021

*Please ensure you delete the link to your author homepage in this e-mail if you wish to forward it to your co-authors.

Dear Dr Lameira,

Your manuscript entitled "Social template (mis)matching in wild orangutan vocal production" has now been seen by three reviewers, whose comments are attached. The reviewers have raised a number of concerns which will need to be addressed before we can offer publication in Nature Ecology & Evolution. We will therefore need to see your responses to the criticisms raised and to some editorial concerns, along with a revised manuscript, before we can reach a final decision regarding publication.

Most importantly, please ensure that the revised version includes links to access all data and code necessary to reproduce the findings of the manuscript--while we strongly encourage accessible data at publication, it is absolutely essential during review so that referees can properly assess the manuscript.

We therefore invite you to revise your manuscript taking into account all reviewer and editor comments. Please highlight all changes in the manuscript text file [OPTIONAL: in Microsoft Word format].

We are committed to providing a fair and constructive peer-review process. Do not hesitate to contact us if there are specific requests from the reviewers that you believe are technically impossible or unlikely to yield a meaningful outcome.

When revising your manuscript:

* Include a “Response to reviewers” document detailing, point-by-point, how you addressed each reviewer comment. If no action was taken to address a point, you must provide a compelling argument. This response will be sent back to the reviewers along with the revised manuscript.

* If you have not done so already please begin to revise your manuscript so that it conforms to our Article format instructions at http://www.nature.com/natecolevol/info/final-submission. Refer also to any guidelines provided in this letter.

* Include a revised version of any required reporting checklist. It will be available to referees (and, potentially, statisticians) to aid in their evaluation if the manuscript goes back for peer review. A revised checklist is essential for re-review of the paper.

Please use the link below to submit your revised manuscript and related files:

[REDACTED]

Note: This URL links to your confidential home page and associated information about manuscripts you may have submitted, or that you are reviewing for us. If you wish to forward this email to co-authors, please delete the link to your homepage.

We hope to receive your revised manuscript within four to eight weeks. If you cannot send it within this time, please let us know. We will be happy to consider your revision so long as nothing similar has been accepted for publication at Nature Ecology & Evolution or published elsewhere.

Nature Ecology & Evolution is committed to improving transparency in authorship. As part of our efforts in this direction, we are now requesting that all authors identified as ‘corresponding author’ on published papers create and link their Open Researcher and Contributor Identifier (ORCID) with their account on the Manuscript Tracking System (MTS), prior to acceptance. ORCID helps the scientific community achieve unambiguous attribution of all scholarly contributions. You can create and link your ORCID from the home page of the MTS by clicking on ‘Modify my Springer Nature account’. For more information please visit please visit www.springernature.com/orcid.

Please do not hesitate to contact me if you have any questions or would like to discuss these revisions further.

We look forward to seeing the revised manuscript and thank you for the opportunity to review your work.

[REDACTED]

Reviewer expertise:

Reviewer #1: vocal learning in songbirds

Open Access This file is licensed under a Creative Commons Attribution 4.0 International License, which permits use, sharing, adaptation, distribution and reproduction in any medium or format, as long as you give appropriate credit to the original author(s) and the source, provide a link to the Creative Commons license, and indicate if changes were made. In the cases where the authors are anonymous, such as is the case for the reports of anonymous peer reviewers, author attribution should be to 'Anonymous Referee' followed by a clear attribution to the source work. The images or other third party material in this file are included in the article’s Creative Commons license, unless indicated otherwise in a credit line to the material. If material is not included in the article’s Creative Commons license and your intended use is not permitted by statutory regulation or exceeds the permitted use, you will need to obtain permission directly from the copyright holder. To view a copy of this license, visit http://creativecommons.org/licenses/by/4.0/.

Reviewer #2: vocal learning in marmosets

Reviewer #3: great ape behavioural flexibility --note that we were not aware that this reviewer had previously reviewed the manuscript elsewhere, as they divulge in their report. We don't see this fact as problematic, given that the reviewer has clearly assessed the current submitted version rather than any previous version for their report here.

Reviewers' comments:

Reviewer #1 (Remarks to the Author):

This is a potentially important study that provides, for the first time, a large-scale cross-populations analysis of vocal behavior in wild populations of orangutans. The study discovered that vocal repertoire varies with population size, individual age and sex, and perhaps also with social context (see comments below). Authors developed new measures to describe the vocal behavior of these apes, and performed statistical analysis, which might need adjustments (but see comments below). Overall, this study is of broad interest, but before it can be published, several issues must be addressed:

1. Some of the conclusions presented here are not supported by the data, and should be reduced or revised until further evidence are available. In particular, authors cannot conclude that there is any vocal learning or even vocal plasticity given their data. The fact that population density is correlated with acoustic features is not a conclusive evidence. Take for example the vocalization of dogs: In higher density, for example is a dog park, one can observe many more vocalization compared to home environment. This is simply because the social environment includes more states, such as when two dogs attack each other, or play with each other. Each behavioral state results with different vocalization in the dog. But dogs are not known to have any vocal learning or vocal plasticity – these are all innate vocalization as much as we know. The current study cannot solve this issue unless local dialects can be included. However, authors did not include what they considered to be ‘location specific’ vocalization. This is unfortunate because such vocalization could support their claims. See minor comments for specific issues with conclusions and interpretation below.

2. Authors do not present a single sonogram in this MS, which is unbelievable. Several examples should be presented for each of the study parameters, to give the reader some intuition of how different those vocalizations are. This is very frustrating for me as a reviewer to try to evaluate a study where no raw data are presented. Further, authors do not show how they derive acoustic measures from the sonograms, and do not show how those raw measures are transformed to the entropy measures. Therefore, I could not interpret the robustness of the procedure. For example, the definition of emergence is the rate at which new signal states appear in an individual’s repertoire. I could not figure out what this means? What is a new signal? Looking at the vector of peak frequency for example – what is new? Is the data segmented? How? What sort of decisions are made during signal processing? None of this is specified.

Open Access This file is licensed under a Creative Commons Attribution 4.0 International License, which permits use, sharing, adaptation, distribution and reproduction in any medium or format, as long as you give appropriate credit to the original author(s) and the source, provide a link to the Creative Commons license, and indicate if changes were made. In the cases where the authors are anonymous, such as is the case for the reports of anonymous peer reviewers, author attribution should be to 'Anonymous Referee' followed by a clear attribution to the source work. The images or other third party material in this file are included in the article’s Creative Commons license, unless indicated otherwise in a credit line to the material. If material is not included in the article’s Creative Commons license and your intended use is not permitted by statutory regulation or exceeds the permitted use, you will need to obtain permission directly from the copyright holder. To view a copy of this license, visit http://creativecommons.org/licenses/by/4.0/.

3. I am a bit worried about the statistical model used. I would like to see the entire ML model including the outcome of comparing the null model to the experiment model. Please provide the actual R code and raw results in the supplement so this can be properly assessed. I am not sure that the models are correctly set and more importantly, that p values are properly adjusted to multiplicity of measurements and approaches taken here.

Specific comments:

1. Abstract last statements are problematic: "populations. In denser populations, individuals are vocally more innovative but new vocal variants are short-lived, leading to more confirmative, consistent and also complex vocal repertoires in dispersed populations. Findings provide first non-invasive evidence that degree in social contact predicts vocal phenotype and repertoire structure in a wild great ape.": First, your data do not show anything about vocal innovation, what you measured in entropy. As stated above, this entropy could change due to different social interaction associated with density, sex, age, etc. These are not vocal phenotypes and you should avoid this term here unless more analysis is done to support it.

2. "…hypothesis that great apes are incapable of vocal (production) learning and poor models of language evolution research, individuals should operate as independent agents and their vocal development should take course autonomously, with individuals producing calls equally variable to one another, regardless of social setting or population": This statement is false and must be revised. The lack of vocal learning does not mean at all that you will not observe variability in vocalization. Such variability may simply stem from different behavioral states in different conditions. You will find such variation even in the buzzing of house flies depending on the condition there are in!

3. "Methods). We conducted six generalized linear models, each with one of the entropic parameters as response variable.". Since you conduced 6 models, you may need to adjust your p values for multiplicity. This is of course after each model is tested against a null model – all this should be presented in the supplement.

4. "168 Results reject the traditional hypothesis that great ape vocal development is impervious to social input, according to which vocal variability and repertoire structure per individual ought to be identical within and between populations,": this is incorrect and besides, you did not measure vocal development at all here. I would suggest removing this statement.

5. Figure 1 is substandard in quality: please improve it. The gray foreground and the white grid below the gray markers makes the graph difficult to look at. Each axis must have units and legend.

Reviewer #2 (Remarks to the Author):

The authors study the relationship between orangutan densities and some acoustic characteristics of

Open Access This file is licensed under a Creative Commons Attribution 4.0 International License, which permits use, sharing, adaptation, distribution and reproduction in any medium or format, as long as you give appropriate credit to the original author(s) and the source, provide a link to the Creative Commons license, and indicate if changes were made. In the cases where the authors are anonymous, such as is the case for the reports of anonymous peer reviewers, author attribution should be to 'Anonymous Referee' followed by a clear attribution to the source work. The images or other third party material in this file are included in the article's Creative Commons license, unless indicated otherwise in a credit line to the material. If material is not included in the article's Creative Commons license and your intended use is not permitted by statutory regulation or exceeds the permitted use, you will need to obtain permission directly from the copyright holder. To view a copy of this license, visit http://creativecommons.org/licenses/by/4.0/.

kiss-squeak calls. The central claim is that the degree of social contact predicts vocal phenotype and repertoire structure in orangutans. In particular, denser populations exhibit larger vocal variability. I find the article very interesting. The general aim of demonstrating that (non-human) great apes show vocal plasticity beyond what has been traditionally acknowledged is relevant. I agree with the authors that the lack of studies of vocal plasticity in apes has been used as evidence of the absence of plasticity, which is logically incorrect. Given the heated debate around this issue within the scientific community and in the popular press, this study is relevant to the field and the general scientific community.

My main comments for improvement are that the authors overinterpret some of the results and should better discuss the study's caveats. Moreover, I have some concerns about the entropic measures used in the study that should be easily addressable. I hope the following comments help the authors.

Major comments
1) The authors use the subject density as a proxy for the degree of social contact. This sounds reasonable, but subject density is also correlated with several other factors like resource availability, type of food, average subject size, the proportion of age/sex groups, logging activities, the density of predators, foraging strategies, etc. Some of these relationships are strongly supported in references 57-58 cited in the manuscript (also chapter 8 of the same book). Hence, it is equally or maybe even more parsimonious to relate those characteristics with changes in vocal characteristics than the degree of social contact (for which the association is weaker). The authors might want to consider these possibilities and change the conclusion accordingly. Doing so does not exclude the possibility that the orangutans exhibit vocal plasticity but makes the conclusion more nuanced and precise.

2) Related to previous comments, the authors did not measure the degree of social contact in the study, hence again, the conclusion should take this fact into account.

3) The authors should describe during which months/seasons of the year data were collected. It is also not clear whether the data were collected during several years. Could those factors affect the result because the call variability during more extended periods implies more significant variability?

4) If I understood correctly, the authors reported that the density data was taken from another reference (reference 57) and not directly measured during the recording period. Is it possible that the density changed significantly, and therefore, the results are invalid?

5) The discussion about learning is imprecise. For example, in line 168, the claim that "Results reject the traditional hypothesis that great ape vocal development is impervious to social input ..." seems to be a stretch given that it is a cross-sectional and observational study. The manuscript show evidence of change in the characteristics of a single call type, which correlates with group density. This is not evidence of template (social) learning in the traditional sense. The reported evidence suggests vocal plasticity, but there is no evidence that the change is "learned" or "learned to be different" from one individual of the same group to the other. The difference of call characteristics in flanged males is interesting, but it is reasonable to think it is related to hormonal and morphological changes.

Open Access This file is licensed under a Creative Commons Attribution 4.0 International License, which permits use, sharing, adaptation, distribution and reproduction in any medium or format, as long as you give appropriate credit to the original author(s) and the source, provide a link to the Creative Commons license, and indicate if changes were made. In the cases where the authors are anonymous, such as is the case for the reports of anonymous peer reviewers, author attribution should be to 'Anonymous Referee' followed by a clear attribution to the source work. The images or other third party material in this file are included in the article's Creative Commons license, unless indicated otherwise in a credit line to the material. If material is not included in the article's Creative Commons license and your intended use is not permitted by statutory regulation or exceeds the permitted use, you will need to obtain permission directly from the copyright holder. To view a copy of this license, visit http://creativecommons.org/licenses/by/4.0/.

6) What is the proportion of flanged and non-flanged males in each population? Given that flanged males have a major effect on the entropic measures, I wonder if there is a relevant interaction between population density and Age.

7) More generally, the authors should test the model in which population density interacts with Age, Sex, Island, and Context.

8) The authors should discuss better why they chose to analyze kiss-squeaks. Why would it make sense that kiss-squeaks change their acoustic characteristics depending on population density? What is the social context of the call usage? Do the authors think that other calls are less plastic? It would also be didactic to show the spectrogram of some exemplars of the kiss-squeaks.

9) Self-organization is just 1-Emergence, so I don't see a point in doing a separate analysis for self-organization. All the stats should be identical to both, just reversing the signal and adding a constant. We can clearly see that in table 1 for Duration. Furthermore, the result for Max frequency seems wrong because the p-values don't match for Emergence and Self-organization, unless the authors used different data.

10) The discussion of the reported results should include the literature on vocal divergence. It is more directly related to the findings of the work. This comment also applied to the title. There is no evidence of a template in the results.

11) The authors should describe whether the data is available on a repository or not.

Minor comments:

1) Line 191 is incorrect. Marmosets are cooperative breeders, and they likely receive input from other members of the group. Songbirds in the wild can learn from different tutors.

2) The authors should be more precise on the songbird species they are referring to as different species have different characteristics—especially when discussing zebra finches in the laboratory environment, which is a special case.

Reviewer #3 (Remarks to the Author):

The authors present a cross-population study investigating whether variation in population density (taken as a proxy for varying degrees of sociality) may effect the degree of acoustic variability in orangutan kiss squeaks. I find this study incredibly interesting and a worthwhile contribution to Nature Ecology and Evolution if the authors can clarify some important theoretical and quantitative concerns I have with the manuscript.

Open Access This file is licensed under a Creative Commons Attribution 4.0 International License, which permits use, sharing, adaptation, distribution and reproduction in any medium or format, as long as you give appropriate credit to the original author(s) and the source, provide a link to the Creative Commons license, and indicate if changes were made. In the cases where the authors are anonymous, such as is the case for the reports of anonymous peer reviewers, author attribution should be to 'Anonymous Referee' followed by a clear attribution to the source work. The images or other third party material in this file are included in the article's Creative Commons license, unless indicated otherwise in a credit line to the material. If material is not included in the article's Creative Commons license and your intended use is not permitted by statutory regulation or exceeds the permitted use, you will need to obtain permission directly from the copyright holder. To view a copy of this license, visit http://creativecommons.org/licenses/by/4.0/.

I was pleased to find that some of my comments from a previous review (a different journal) were incorporated in this new version of the manuscript, but some aspects are still not convincing/clear enough in my opinion. That said, I really like the study and would be happy to see a revised version if the authors can address these major concerns.

Major concerns:
1. Statistics issues: I can appreciate that the authors have included individual ID in their models, but I am missing population ID as a random effect considering that if the authors want to attribute their results to variation in population density per se then they must control for stochastic variation associated with sampling different populations. I am also unable to find sample size anywhere in this manuscript (apologies if I've simply overlooked it somewhere), which makes understanding the models and the data points used for each hard to grasp. Please clarify/fix these aspects of the analysis and provide N throughout.

2. It is difficult to understand and appreciate how variation in maximum frequency (or duration) of a single call type is considered innovative behaviour and/or a new vocal variant. If I am understanding this correctly, that is what the authors argue after having measured various ‘entropic variation regimes’ for call duration and max frequency of kiss squeaks. I think a problem lies in my inability to truly grasp what ‘entropic variation regimes’ really translates to and I think the authors need to do a better job of explaining this in a way that is more easily understood to a non-specialist reader. This issue is critical to their paper, since variation in max frequency or duration within a single call type would not normally be considered particularly innovative or more complex in animal communication but rather normal intra-call variation, especially for a graded vocal repertoire characteristic of great apes.

Part of my confusion also lies in the terminology, where the authors argue that high density populations had ‘more complex vocal repertoires’ again, a single call type (kiss squeaks) being used to represent the entire orangutan repertoire is not very convincing. The term usually refers to the whole manner of vocalizations produced by an animal. In general the acoustic measures need to be made more clear as to why they are evidence for what the authors claim. One gets the feeling that even the authors themselves might find it difficult to describe what they measured here and why it is representative of vocal innovativeness or complexity. It would help tremendously if the terminology used in the results and discussion with respect to these entropic measures were made more explicit rather than using broad, over-generalized terms like repertoire, innovative or complex.

3. I can accept that population density translates to a greater degree of sociality and thank the authors for adding the additional lines of evidence, but I think this relationship is still somewhat weak. Critics will argue that population density is insufficient to truly capture sociality (similar to how group size was often criticized as a poor proxy for social complexity). The authors’ claim would be substantially strengthened if they could provide data on variation in social interactions/size of sub-units observed on average, etc. for each population, or something similar.

Minor comment: Line 112- remove the for in ‘and for’

Open Access This file is licensed under a Creative Commons Attribution 4.0 International License, which permits use, sharing, adaptation, distribution and reproduction in any medium or format, as long as you give appropriate credit to the original author(s) and the source, provide a link to the Creative Commons license, and indicate if changes were made. In the cases where the authors are anonymous, such as is the case for the reports of anonymous peer reviewers, author attribution should be to 'Anonymous Referee' followed by a clear attribution to the source work. The images or other third party material in this file are included in the article’s Creative Commons license, unless indicated otherwise in a credit line to the material. If material is not included in the article’s Creative Commons license and your intended use is not permitted by statutory regulation or exceeds the permitted use, you will need to obtain permission directly from the copyright holder. To view a copy of this license, visit http://creativecommons.org/licenses/by/4.0/.

********************END********************

Author Rebuttal to Initial comments

Reviews

Reviewers' comments:

Reviewer #1 (Remarks to the Author):

This is a potentially important study that provides, for the first time, a large-scale cross-populations analysis of vocal behavior in wild populations of orangutans. The study discovered that vocal repertoire varies with population size, individual age and sex, and perhaps also with social context (see comments below). Authors developed new measures to describe the vocal behavior of these apes, and performed statistical analysis, which might need adjustments (but see comments below). Overall, this study is of broad interest, but before it can be published, several issues must be addressed:

1. Some of the conclusions presented here are not supported by the data, and should be reduced or revised until further evidence are available. In particular, authors cannot conclude that there is any vocal learning or even vocal plasticity given their data. The fact that population density is correlated with acoustic features is not a conclusive evidence. Take for example the vocalization of dogs: In higher density, for example is a dog park, one can observe many more vocalization compared to home environment. This is simply because the social environment includes more states, such as when two dogs attack each other, or play with each other. Each behavioral state results with different vocalization in the dog. But dogs are not known to have any vocal learning or vocal plasticity – these are all innate vocalization as much as we know. The current study cannot solve this issue unless local dialects can be included. However, authors did not include what they considered to be ‘location specific’ vocalization. This is unfortunate because such vocalization could support their claims. See minor comments for specific issues with conclusions and interpretation below.

We thank the reviewer for raising here a very pertinent issue and for drawing our attention to the important point of potential ecological and social confounds. We apologise we did not explicitly

Open Access This file is licensed under a Creative Commons Attribution 4.0 International License, which permits use, sharing, adaptation, distribution and reproduction in any medium or format, as long as you give appropriate credit to the original author(s) and the source, provide a link to the Creative Commons license, and indicate if changes were made. In the cases where the authors are anonymous, such as is the case for the reports of anonymous peer reviewers, author attribution should be to 'Anonymous Referee' followed by a clear attribution to the source work. The images or other third party material in this file are included in the article’s Creative Commons license, unless indicated otherwise in a credit line to the material. If material is not included in the article’s Creative Commons license and your intended use is not permitted by statutory regulation or exceeds the permitted use, you will need to obtain permission directly from the copyright holder. To view a copy of this license, visit http://creativecommons.org/licenses/by/4.0/.

address this issue in our original version. Following the reviewer's comment, we have now added sub-headings in the introduction (lines 104-201) explaining extensively our methodological decisions, particularly with regards to how we excluded any potential biases and confounding effects due to ecological or social differences between populations.

We believe these new subheadings also provide some important clarifications (though not exclusively) about the calls that were measured and analysed, helping to address the concerns raised by the reviewer's next point. Please see next point for further revision steps taken.

2. Authors do not present a single sonogram in this MS, which is unbelievable. Several examples should be presented for each of the study parameters, to give the reader some intuition of how different those vocalizations are. This is very frustrating for me as a reviewer to try to evaluate a study where no raw data are presented. Further, authors do not show how they derive acoustic measures from the sonograms, and do not show how those raw measures are transformed to the entropy measures. Therefore, I could not interpret the robustness of the procedure. For example, the definition of emergence is the rate at which new signal states appear in an individual's repertoire. I could not figure out what this means? What is a new signal? Looking at the vector of peak frequency for example – what is new? Is the data segmented? How? What sort of decisions are made during signal processing? None of this is specified.

We agree with the reviewer and the points raised are fully germane. Following the reviewer's advice, we have new included a new figure that displays call sonograms and illustrates how acoustic parameters where extracted (new Fig. 1). In addition to the transformation formulas provided in the methods, the new figure also visually depicts these transformations for a more readily and intuitive interpretation of our protocol and results. For full clarity, transparency and accessibility, we now also include as supplementary data the descriptive stats of the acoustic data and the R code notebook necessary to transform the acoustic data into the entropic data.

3. I am a bit worried about the statistical model used. I would like to see the entire ML model including the outcome of comparing the null model to the experiment model. Please provide the actual R code and raw results in the supplement so this can be properly assessed. I am not sure that the models are correctly set and more importantly, that p values are properly adjusted to multiplicity of measurements and approaches taken here.

To address this point raised by the reviewer, we have now re-run our models using JASP, a non-code-based open-source statistic package with intuitive use that allows directly exporting model results in table form (as well as a R-bridge for advanced users) and we now include both data

Open Access This file is licensed under a Creative Commons Attribution 4.0 International License, which permits use, sharing, adaptation, distribution and reproduction in any medium or format, as long as you give appropriate credit to the original author(s) and the source, provide a link to the Creative Commons license, and indicate if changes were made. In the cases where the authors are anonymous, such as is the case for the reports of anonymous peer reviewers, author attribution should be to 'Anonymous Referee' followed by a clear attribution to the source work. The images or other third party material in this file are included in the article's Creative Commons license, unless indicated otherwise in a credit line to the material. If material is not included in the article's Creative Commons license and your intended use is not permitted by statutory regulation or exceeds the permitted use, you will need to obtain permission directly from the copyright holder. To view a copy of this license, visit http://creativecommons.org/licenses/by/4.0/.

descriptive stats, as well as model tables (including, fit statistics, samples sizes, fixed effects estimates and estimated marginal means) as supplementary materials to facilitate readers' assessment and interpretation.

We have now also ensured that the description of the models and their respective factors are comprehensively explained and clearly indicated in the text, both in the Introduction (line 225) and Methods (line 427). We hope this will clarify the readers that each model relied on different sets of data, and therefore, that no p-correction applies.

Specific comments:

1. Abstract last statements are problematic: "populations. In denser populations, individuals are vocally more innovative but new vocal variants are short-lived, leading to more confirmative, consistent and also complex vocal repertoires in dispersed populations. Findings provide first non-invasive evidence that degree in social contact predicts vocal phenotype and repertoire structure in a wild great ape.": First, your data do not show anything about vocal innovation, what you measured in entropy. As stated above, this entropy could change due to different social interaction associated with density, sex, age, etc. These are not vocal phenotypes and you should avoid this term here unless more analysis is done to support it.

It seems we have created some confusion and tried to clarify this in the new version. We did not measure "acoustic entropy" as suggested. This confusion may have been in part also because acoustic entropy has been the parameter used to track call variation in past lab studies. As the reviewer correctly points out, acoustic entropy does not characterize vocal innovation or vocal phenotype. Conversely, we used "repertoire entropy" measures that chart the probability distributions of calls within an individual's call set, and thus, that characterise that individual's repertoire variation regime. Indeed, the main function of these parameters is to measure the degree/rate of novel or conserved states within a system.

*To fully address the reviewer's concerns and pre-empt future misunderstanding by the readers, we made several edits: **First**, we have now inserted new sub-section in the Introduction (line 104-201) laying out in more detail that our entropic measures are calculated from an individual's repertoire, not from an individual's single calls (as done with acoustic entropy). These differences were mentioned in the original version, but only in the discussion section. We hope to have made this point clearer by bringing an extensive explanation much earlier in the article (together with a brief mention in the Abstract). **Second**, a new figure (mentioned in comment above, new Fig. 1) provides now a graphic aid to visually understand how our entropic measures were calculated. Data transformations and respective formulas were provided in the original version, but this format is admittedly difficult and requires advanced algebra knowledge. We hope the new figure unpacks*

Open Access This file is licensed under a Creative Commons Attribution 4.0 International License, which permits use, sharing, adaptation, distribution and reproduction in any medium or format, as long as you give appropriate credit to the original author(s) and the source, provide a link to the Creative Commons license, and indicate if changes were made. In the cases where the authors are anonymous, such as is the case for the reports of anonymous peer reviewers, author attribution should be to 'Anonymous Referee' followed by a clear attribution to the source work. The images or other third party material in this file are included in the article's Creative Commons license, unless indicated otherwise in a credit line to the material. If material is not included in the article's Creative Commons license and your intended use is not permitted by statutory regulation or exceeds the permitted use, you will need to obtain permission directly from the copyright holder. To view a copy of this license, visit http://creativecommons.org/licenses/by/4.0/.

these transformations in a much more intuitive and readily accessible format to the reader. We also provide the R code notebook to calculate the entropic measures as supplementary material. ***Third****, we have avoided the use of the term "innovation". As hinted by the reviewer, the term carries a very specific connotation in the animal social learning literature. We believe our original wording choice could have been confusing and we hope this correction will help the reader understand more promptly our results and their interpretation.*

2. "…hypothesis that great apes are incapable of vocal (production) learning and poor models of language evolution research, individuals should operate as independent agents and their vocal development should take course autonomously, with individuals producing calls equally variable to one another, regardless of social setting or population": This statement is false and must be revised. The lack of vocal learning does not mean at all that you will not observe variability in vocalization. Such variability may simply stem from different behavioral states in different conditions. You will find such variation even in the buzzing of house flies depending on the condition there are in!

We have now amended the target sentence according to the reviewer's advice. Indeed, as the reviewer correctly points out, our previous passage could be interpreted too generally, creating ambiguity about the effects that we are referring to and mainlys interested in. We have now clarified that the target prediction pertains strictly to the degree of sociality, whose effects we set out to assess specifically in the paper. Together with new sub-sections of the Introduction (line 104-201), we hope the text now clarifies this.

3. "Methods). We conducted six generalized linear models, each with one of the entropic parameters as response variable.". Since you conduced 6 models, you may need to adjust your p values for multiplicity. This is of course after each model is tested against a null model – all this should be presented in the supplement.

As indicated in the target sentence, each model is based on a distinct data set (i.e. entropic measures calculated from different acoustic measures). Our models do therefore not represent, repeated/serial tests of the same dataset. Therefore, we think that no p inflation applies to this analytical set up. To curtail any possible misunderstanding by the readers, we now explain in detail the structure of our models in the Introduction, in addition to the Methods section, together with model fit statistics, samples sizes, fixed effects estimates and estimated marginal means as ESM.

4. "168 Results reject the traditional hypothesis that great ape vocal development is impervious to social input, according to which vocal variability and repertoire structure per individual ought to be

Open Access This file is licensed under a Creative Commons Attribution 4.0 International License, which permits use, sharing, adaptation, distribution and reproduction in any medium or format, as long as you give appropriate credit to the original author(s) and the source, provide a link to the Creative Commons license, and indicate if changes were made. In the cases where the authors are anonymous, such as is the case for the reports of anonymous peer reviewers, author attribution should be to 'Anonymous Referee' followed by a clear attribution to the source work. The images or other third party material in this file are included in the article's Creative Commons license, unless indicated otherwise in a credit line to the material. If material is not included in the article's Creative Commons license and your intended use is not permitted by statutory regulation or exceeds the permitted use, you will need to obtain permission directly from the copyright holder. To view a copy of this license, visit http://creativecommons.org/licenses/by/4.0/.

identical within and between populations,": this is incorrect and besides, you did not measure vocal development at all here. I would suggest removing this statement.

The reviewer is correct in indicating that we did not directly measure vocal development. For absolute clarity, we have now added new Introduction sub-sections dedicated to explaining how we adapted lab-based longitudinal studies into wild-based cross-sectional study. We have now ensured explicitly that our study is cross-sectional and have removed any passage that could imply or appear to suggest that our study was developmental in nature. The target sentence has now been fully rephrased (line 233).

5. Figure 1 is substandard in quality: please improve it. The gray foreground and the white grid below the gray markers makes the graph difficult to look at. Each axis must have units and legend.

Our submission included a high-resolution image, so we suspect the reviewer may be referring here to the figure embedded in the text, which does indeed tend to lose resolution when pdf's are generated from Word. We have now removed this figure from the text to avoid any confusion and have submitted all our new figures as separate files only to prevent quality loss or the use of low-quality copies as reference. We are unclear regarding what the reviewer refers to as markers, but we have now modified the colour theme of all graphs for added contrast. The entropic measures used are unitless, hence, they have now units. Axis legends have been added, as requested.

Reviewer #2 (Remarks to the Author):

The authors study the relationship between orangutan densities and some acoustic characteristics of kiss-squeak calls. The central claim is that the degree of social contact predicts vocal phenotype and repertoire structure in orangutans. In particular, denser populations exhibit larger vocal variability. I find the article very interesting. The general aim of demonstrating that (non-human) great apes show vocal plasticity beyond what has been traditionally acknowledged is relevant. I agree with the authors that the lack of studies of vocal plasticity in apes has been used as evidence of the absence of plasticity, which is logically incorrect. Given the heated debate around this issue within the scientific community and in the popular press, this study is relevant to the field and the general scientific community.

My main comments for improvement are that the authors overinterpret some of the results and should better discuss the study's caveats. Moreover, I have some concerns about the entropic measures used in the study that should be easily addressable. I hope the following comments help the authors.

Open Access This file is licensed under a Creative Commons Attribution 4.0 International License, which permits use, sharing, adaptation, distribution and reproduction in any medium or format, as long as you give appropriate credit to the original author(s) and the source, provide a link to the Creative Commons license, and indicate if changes were made. In the cases where the authors are anonymous, such as is the case for the reports of anonymous peer reviewers, author attribution should be to 'Anonymous Referee' followed by a clear attribution to the source work. The images or other third party material in this file are included in the article's Creative Commons license, unless indicated otherwise in a credit line to the material. If material is not included in the article's Creative Commons license and your intended use is not permitted by statutory regulation or exceeds the permitted use, you will need to obtain permission directly from the copyright holder. To view a copy of this license, visit http://creativecommons.org/licenses/by/4.0/.

Major comments

1) The authors use the subject density as a proxy for the degree of social contact. This sounds reasonable, but subject density is also correlated with several other factors like resource availability, type of food, average subject size, the proportion of age/sex groups, logging activities, the density of predators, foraging strategies, etc. Some of these relationships are strongly supported in references 57-58 cited in the manuscript (also chapter 8 of the same book). Hence, it is equally or maybe even more parsimonious to relate those characteristics with changes in vocal characteristics than the degree of social contact (for which the association is weaker). The authors might want to consider these possibilities and change the conclusion accordingly. Doing so does not exclude the possibility that the orangutans exhibit vocal plasticity but makes the conclusion more nuanced and precise.

The reviewer raises here pertinent points relatively to potential ecological and social confounds. Our previous version wasn't sufficiently clear about this important aspect of the study's empirical paradigm. Following the reviewer's comment, we have now added new Introduction sub-sections that extensively explain and motivate how and why these effects were controlled for in our methodological set up and statistical analyses (line 104-201). Among other points, we clarify that analyses are focused on alarm calls as these allow to bypass potential social and ecological confounds, such as those touched upon by the reviewer. We now also provide new detail, code and a new figure about our entropic measures and analyses. We wish this will also provide added clarify about the fact that we are characterizing the "regimes" of repertoire structure and dynamics, not "raw" call acoustics, making the probability of potential social or ecological confounding effects further unlikely. This point ties with a further pointed raised by the reviewer, and further elaborated, below.

2) Related to previous comments, the authors did not measure the degree of social contact in the study, hence again, the conclusion should take this fact into account.

In light of the reviewer's input, we have now removed any passage that could be interpreted as an indication that we directly measured degree of social contact. We now also further clarify early in the Introduction that we are using populational density as a surrogate measure of sociality and why this is an adequate and operational variable with wild orangutans (line 109-121). We now also provide further details about transposing lab studies into the wild in the new sub-sections of the Introduction.

3) The authors should describe during which months/seasons of the year data were collected. It is

Open Access This file is licensed under a Creative Commons Attribution 4.0 International License, which permits use, sharing, adaptation, distribution and reproduction in any medium or format, as long as you give appropriate credit to the original author(s) and the source, provide a link to the Creative Commons license, and indicate if changes were made. In the cases where the authors are anonymous, such as is the case for the reports of anonymous peer reviewers, author attribution should be to 'Anonymous Referee' followed by a clear attribution to the source work. The images or other third party material in this file are included in the article's Creative Commons license, unless indicated otherwise in a credit line to the material. If material is not included in the article's Creative Commons license and your intended use is not permitted by statutory regulation or exceeds the permitted use, you will need to obtain permission directly from the copyright holder. To view a copy of this license, visit http://creativecommons.org/licenses/by/4.0/.

also not clear whether the data were collected during several years. Could those factors affect the result because the call variability during more extended periods implies more significant variability?

This is an interesting point raised by the reviewer. We now provide new detailed explanation of why seasonal effects are not expected to have biased data collection (line 330).

4) If I understood correctly, the authors reported that the density data was taken from another reference (reference 57) and not directly measured during the recording period. Is it possible that the density changed significantly, and therefore, the results are invalid?

The reviewer raises an interesting point here, however, directly measuring population density is not possible with wild orangutans given the dispersed nature of their social organization, particularly with regards to the roving character of males. Density estimates rely on long-term and intense surveys conducted to determine nest density and nest decomposition across areas. We now indicate the years during which recordings were collected (line 335).

5) The discussion about learning is imprecise. For example, in line 168, the claim that "Results reject the traditional hypothesis that great ape vocal development is impervious to social input ..." seems to be a stretch given that it is a cross-sectional and observational study. The manuscript show evidence of change in the characteristics of a single call type, which correlates with group density. This is not evidence of template (social) learning in the traditional sense.

We have now corrected the target sentence and have rephrased the title for added accuracy.

The reported evidence suggests vocal plasticity, but there is no evidence that the change is "learned" or "learned to be different" from one individual of the same group to the other. The difference of call characteristics in flanged males is interesting, but it is reasonable to think it is related to hormonal and morphological changes.

We have removed any instance indicating that our results demonstrate learning, as correctly indicated by the reviewer.

6) What is the proportion of flanged and non-flanged males in each population? Given that flanged

Open Access This file is licensed under a Creative Commons Attribution 4.0 International License, which permits use, sharing, adaptation, distribution and reproduction in any medium or format, as long as you give appropriate credit to the original author(s) and the source, provide a link to the Creative Commons license, and indicate if changes were made. In the cases where the authors are anonymous, such as is the case for the reports of anonymous peer reviewers, author attribution should be to 'Anonymous Referee' followed by a clear attribution to the source work. The images or other third party material in this file are included in the article's Creative Commons license, unless indicated otherwise in a credit line to the material. If material is not included in the article's Creative Commons license and your intended use is not permitted by statutory regulation or exceeds the permitted use, you will need to obtain permission directly from the copyright holder. To view a copy of this license, visit http://creativecommons.org/licenses/by/4.0/.

males have a major effect on the entropic measures, I wonder if there is a relevant interaction between population density and Age.

Descriptive statistics are now provided as supplementary materials to allow the reader to explore any detail of interest, anticipating similar questions such as those raised by the reviewer. The examples provided by the reviewer here are certainly of interest and could be leaned over in future studies of explorative nature. Our analyses, however, were set out to test a very specific relationship and prediction – between population density and repertoire entropy. Sex, species, age-sex classes and contexts are control variables. Any interaction would render uninterpretable the effects of the single factors involved. Including interactions would also exceed the number of factors allowed in a model with a sample size such as ours.

7) More generally, the authors should test the model in which population density interacts with Age, Sex, Island, and Context.

We agree with the reviewer that there are many possible interesting interactions between the fixed factors used. However, we prevented ourselves from conducting these analyses for two main reasons, one heuristic and one technical. Heuristically, our working hypothesis does not predict any specific relationship/interaction between specific fixed factors, and therefore shouldn't be arbitrarily included. Such interaction(s) could be included in explorative studies, but technically, conducting multiple additional interactional analyses starkly decreases statistical power. ***Critically****, and of vital important to our study, including any interaction between two variables would render unviable their separate interpretation, particularly in relation to population density. For instance, including density*age interaction renders the interpretation of density and age separately inappropriate. This would defeat the purpose of our analysis, which seeks to specifically assess the effects of population density on repertoire entropy.*

8) The authors should discuss better why they chose to analyze kiss-squeaks. Why would it make sense that kiss-squeaks change their acoustic characteristics depending on population density? What is the social context of the call usage? Do the authors think that other calls are less plastic? It would also be didactic to show the spectrogram of some exemplars of the kiss-squeaks.

We thank the reviewer for this suggestion. This is now thoroughly addressed in new sub-sections of the Introduction. Following the reviewer's request, we now also include a spectrogram of exemplar

Open Access This file is licensed under a Creative Commons Attribution 4.0 International License, which permits use, sharing, adaptation, distribution and reproduction in any medium or format, as long as you give appropriate credit to the original author(s) and the source, provide a link to the Creative Commons license, and indicate if changes were made. In the cases where the authors are anonymous, such as is the case for the reports of anonymous peer reviewers, author attribution should be to 'Anonymous Referee' followed by a clear attribution to the source work. The images or other third party material in this file are included in the article's Creative Commons license, unless indicated otherwise in a credit line to the material. If material is not included in the article's Creative Commons license and your intended use is not permitted by statutory regulation or exceeds the permitted use, you will need to obtain permission directly from the copyright holder. To view a copy of this license, visit http://creativecommons.org/licenses/by/4.0/.

kiss-squeaks, together with a visual diagram to facilitate a better and more accurate intuitive understanding of the nature of our entropic measures and analyses (new Fig. 1).

9) Self-organization is just 1-Emergence, so I don't see a point in doing a separate analysis for self-organization. All the stats should be identical to both, just reversing the signal and adding a constant. We can clearly see that in table 1 for Duration. Furthermore, the result for Max frequency seems wrong because the p-values don't match for Emergence and Self-organization, unless the authors used different data.

The reviewer is correct. The relationship between Self-organization and Emergence is indicated in the Introduction and Methods. The legend of the figures clearly states that the presentation of Self-organization and Emergence is done solely to facilitate interpretation by the reader. For added clarity, in the new Fig. 2, we now also include "rotating" arrows between Emergence and Self-organization and present the results for Emergence and Self-organization as mirrored panels, to make it explicit that the two variables are inter-convertible. Following the reviewer's comment, we have now re-run all stats and provide new result tables, including LMM fit statistics, samples sizes, fixed effects estimates and estimated marginal means, as ESM. We have now also reformatted Table 1 to bring visual ease for the presentation and interpretation of results for both Emergence and Self-organization, while simultaneously making it clear that both measures and inter-convertible.

10) The discussion of the reported results should include the literature on vocal divergence. It is more directly related to the findings of the work. This comment also applied to the title. There is no evidence of a template in the results.

This is a legitimate observation by the reviewer, and we believe that we may have misguided the reviewer on the point raised here on vocal divergence(/convergence) due to insufficient information in our original version. The original title of the paper may have also partly contributed to this. Title and introduction have now been revised and extended, respectively, for absolute clarity. Our analyses focus of "repertoire entropy", not vocal divergence/convergence; The former characterises variation regimes, the latter characterises precise acoustics. For example, two individuals may exhibit distinct or similar sets of calls (acoustically divergent or convergent, respectively) and show the same level of self-organization in either case, namely, when calls of similar/different acoustics within individual occur at similar rates. To help avoid any future mis-interpretation by our readers, we now directly address this point in the Methods (line 417) and provide new added explanations of our entropic measures, how they are calculate and what they characterise.

Open Access This file is licensed under a Creative Commons Attribution 4.0 International License, which permits use, sharing, adaptation, distribution and reproduction in any medium or format, as long as you give appropriate credit to the original author(s) and the source, provide a link to the Creative Commons license, and indicate if changes were made. In the cases where the authors are anonymous, such as is the case for the reports of anonymous peer reviewers, author attribution should be to 'Anonymous Referee' followed by a clear attribution to the source work. The images or other third party material in this file are included in the article's Creative Commons license, unless indicated otherwise in a credit line to the material. If material is not included in the article's Creative Commons license and your intended use is not permitted by statutory regulation or exceeds the permitted use, you will need to obtain permission directly from the copyright holder. To view a copy of this license, visit http://creativecommons.org/licenses/by/4.0/.

11) The authors should describe whether the data is available on a repository or not.

Thank you for drawing our attention to this point. Descriptive stats for the acoustic data are now provided directly in the paper as ESM, together with descriptive stats for the entropy data (i.e. data input to LMM's) and R code notebook used to transform acoustic into entropic data.

Minor comments:

1) Line 191 is incorrect. Marmosets are cooperative breeders, and they likely receive input from other members of the group. Songbirds in the wild can learn from different tutors.

The reviewer is correct. However, regarding the marmoset breeding system, vocal learning effects have been observed specifically with regards to parental vocal input (e.g. Takahashi et al. 2015 in Science, e.g. Gultekin & Hage 2018 in Science Advances). To avoid mis-describing the various possible mechanisms through which songbirds learn their songs from tutors in the wild, we have now rephrased the target sentence.

2) The authors should be more precise on the songbird species they are referring to as different species have different characteristics—especially when discussing zebra finches in the laboratory environment, which is a special case.

We thank the reviewer for bringing up this point and we have now corrected this passage as indicated above.

Reviewer #3 (Remarks to the Author):

The authors present a cross-population study investigating whether variation in population density (taken as a proxy for varying degrees of sociality) may effect the degree of acoustic variability in orangutan kiss squeaks. I find this study incredibly interesting and a worthwhile contribution to Nature Ecology and Evolution if the authors can clarify some important theoretical and quantitative concerns I have with the manuscript.

I was pleased to find that some of my comments from a previous review (a different journal) were

Open Access This file is licensed under a Creative Commons Attribution 4.0 International License, which permits use, sharing, adaptation, distribution and reproduction in any medium or format, as long as you give appropriate credit to the original author(s) and the source, provide a link to the Creative Commons license, and indicate if changes were made. In the cases where the authors are anonymous, such as is the case for the reports of anonymous peer reviewers, author attribution should be to 'Anonymous Referee' followed by a clear attribution to the source work. The images or other third party material in this file are included in the article's Creative Commons license, unless indicated otherwise in a credit line to the material. If material is not included in the article's Creative Commons license and your intended use is not permitted by statutory regulation or exceeds the permitted use, you will need to obtain permission directly from the copyright holder. To view a copy of this license, visit http://creativecommons.org/licenses/by/4.0/.

incorporated in this new version of the manuscript, but some aspects are still not convincing/clear enough in my opinion. That said, I really like the study and would be happy to see a revised version if the authors can address these major concerns.

Major concerns:

1. Statistics issues: I can appreciate that the authors have included individual ID in their models, but I am missing population ID as a random effect considering that if the authors want to attribute their results to variation in population density per se then they must control for stochastic variation associated with sampling different populations. I am also unable to find sample size anywhere in this manuscript (apologies if I've simply overlooked it somewhere), which makes understanding the models and the data points used for each hard to grasp. Please clarify/fix these aspects of the analysis and provide N throughout.

We are thankful to the reviewer for drawing our attention to these points (as well as others important points made in the previous review round for a previous journal, which we made sure to incorporate). We now indicate sample sizes (lines 220 + 223), as advised, and provide additional descriptive statistics as supplementary materials, where the composition of our data is fully unpacked to facilitate readers' interpretation, both for the acoustic and entropic data.

The question regarding the insertion of population ID as random effect is a pertinent one. We now explain in a new paragraph (line 437) why the variable was not included in our model, either as a random effect (as suggested by the reviewer) or a fixed effect. We appreciate that this request by the reviewer may have derived in part from insufficient clarity on our side regarding model implementation and model results. For the sake of ease of replicability and transparency, we now have re-run our models using JASP, a non-code-based open-source statistic package with intuitive use that allows directly exporting model results in table form. (The program also features a R-bridge for advanced users). We now include R code notebook and these LMM result tables (including fit statistics, samples sizes, fixed effects estimates and estimated marginal means) as supplementary materials to facilitate readers' interpretation.

2. It is difficult to understand and appreciate how variation in maximum frequency (or duration) of a single call type is considered innovative behaviour and/or a new vocal variant. If I am understanding this correctly, that is what the authors argue after having measured various 'entropic variation regimes' for call duration and max frequency of kiss squeaks. I think a problem lies in my inability to truly grasp what 'entropic variation regimes' really translates to and I think the authors need to do a better job of explaining this in a way that is more easily understood to a non-specialist reader. This

Open Access This file is licensed under a Creative Commons Attribution 4.0 International License, which permits use, sharing, adaptation, distribution and reproduction in any medium or format, as long as you give appropriate credit to the original author(s) and the source, provide a link to the Creative Commons license, and indicate if changes were made. In the cases where the authors are anonymous, such as is the case for the reports of anonymous peer reviewers, author attribution should be to 'Anonymous Referee' followed by a clear attribution to the source work. The images or other third party material in this file are included in the article's Creative Commons license, unless indicated otherwise in a credit line to the material. If material is not included in the article's Creative Commons license and your intended use is not permitted by statutory regulation or exceeds the permitted use, you will need to obtain permission directly from the copyright holder. To view a copy of this license, visit http://creativecommons.org/licenses/by/4.0/.

issue is critical to their paper, since variation in max frequency or duration within a single call type would not normally be considered particularly innovative or more complex in animal communication but rather normal intra-call variation, especially for a graded vocal repertoire characteristic of great apes.

Part of my confusion also lies in the terminology, where the authors argue that high density populations had 'more complex vocal repertoires' again, a single call type (kiss squeaks) being used to represent the entire orangutan repertoire is not very convincing. The term usually refers to the whole manner of vocalizations produced by an animal. In general the acoustic measures need to be made more clear as to why they are evidence for what the authors claim. One gets the feeling that even the authors themselves might find it difficult to describe what they measured here and why it is representative of vocal innovativeness or complexity. It would help tremendously if the terminology used in the results and discussion with respect to these entropic measures were made more explicit rather than using broad, over-generalized terms like repertoire, innovative or complex.

This is an important point raised by the reviewer. Although we previously included all formulas used for data transformation and entropic calculations, we admit these are not readily interpretable without advanced algebra knowledge. Therefore, we have now dedicated various new edits specifically with regards to this point highlighted by the reviewer. In order to fully allow the reader to understand what exactly our entropy measures are capturing, and how these measures differ to others typically used in the field, we provide (i) a new figure (Fig. 1), (ii) new Introduction sub-sections (iii) and new supplementary materials (R code notebook).

3. I can accept that population density translates to a greater degree of sociality and thank the authors for adding the additional lines of evidence, but I think this relationship is still somewhat weak. Critics will argue that population density is insufficient to truly capture sociality (similar to how group size was often criticized as a poor proxy for social complexity). The authors' claim would be substantially strengthened if they could provide data on variation in social interactions/size of sub-units observed on average, etc. for each population, or something similar.

This is a valid concern that would apply to social calls exchanged between individuals. We avoided these potential confounding effects by assessing variation regimes of alarm calls. We now address this point (and overall potential social and ecological confounds) extensively in new sub-sections of the Introduction.

Minor comment: Line 112- remove the for in 'and for'

Open Access This file is licensed under a Creative Commons Attribution 4.0 International License, which permits use, sharing, adaptation, distribution and reproduction in any medium or format, as long as you give appropriate credit to the original author(s) and the source, provide a link to the Creative Commons license, and indicate if changes were made. In the cases where the authors are anonymous, such as is the case for the reports of anonymous peer reviewers, author attribution should be to 'Anonymous Referee' followed by a clear attribution to the source work. The images or other third party material in this file are included in the article's Creative Commons license, unless indicated otherwise in a credit line to the material. If material is not included in the article's Creative Commons license and your intended use is not permitted by statutory regulation or exceeds the permitted use, you will need to obtain permission directly from the copyright holder. To view a copy of this license, visit http://creativecommons.org/licenses/by/4.0/.

Corrected accordingly.

Decision Letter, first revision:

9th December 2021

*Please ensure you delete the link to your author homepage in this e-mail if you wish to forward it to your co-authors.

Dear Adriano,

Your manuscript entitled "Sociality predicts orangutan vocal phenotype" has now been seen by the same three reviewers, whose comments are attached. You will see that although reviewers 2 and 3 more or less sign off, there is consensus that the emphasis in the manuscript should be moved to vocal plasticity rather than vocal learning. Additionally, reviewer 1 remains concerned that it is necessary to adjust the statistical analysis for multiple measurements. We will therefore need to see your responses to the criticisms raised, along with a revised manuscript, before we can reach a final decision regarding publication.

We therefore invite you to revise your manuscript taking into account all reviewer and editor comments. Please highlight all changes in the manuscript text file [OPTIONAL: in Microsoft Word format].

We are committed to providing a fair and constructive peer-review process. Do not hesitate to contact us if there are specific requests from the reviewers that you believe are technically impossible or unlikely to yield a meaningful outcome.

When revising your manuscript:

* Include a “Response to reviewers” document detailing, point-by-point, how you addressed each reviewer comment. If no action was taken to address a point, you must provide a compelling argument. This response will be sent back to the reviewers along with the revised manuscript.

* If you have not done so already please begin to revise your manuscript so that it conforms to our Article format instructions at http://www.nature.com/natecolevol/info/final-submission. Refer also to any guidelines provided in this letter.

Open Access This file is licensed under a Creative Commons Attribution 4.0 International License, which permits use, sharing, adaptation, distribution and reproduction in any medium or format, as long as you give appropriate credit to the original author(s) and the source, provide a link to the Creative Commons license, and indicate if changes were made. In the cases where the authors are anonymous, such as is the case for the reports of anonymous peer reviewers, author attribution should be to 'Anonymous Referee' followed by a clear attribution to the source work. The images or other third party material in this file are included in the article’s Creative Commons license, unless indicated otherwise in a credit line to the material. If material is not included in the article’s Creative Commons license and your intended use is not permitted by statutory regulation or exceeds the permitted use, you will need to obtain permission directly from the copyright holder. To view a copy of this license, visit http://creativecommons.org/licenses/by/4.0/.

* Include a revised version of any required reporting checklist. It will be available to referees (and, potentially, statisticians) to aid in their evaluation if the manuscript goes back for peer review. A revised checklist is essential for re-review of the paper.

Please use the link below to submit your revised manuscript and related files:

[REDACTED]

Note: This URL links to your confidential home page and associated information about manuscripts you may have submitted, or that you are reviewing for us. If you wish to forward this email to co-authors, please delete the link to your homepage.

We hope to receive your revised manuscript within four to eight weeks. If you cannot send it within this time, please let us know. We will be happy to consider your revision so long as nothing similar has been accepted for publication at Nature Ecology & Evolution or published elsewhere.

Nature Ecology & Evolution is committed to improving transparency in authorship. As part of our efforts in this direction, we are now requesting that all authors identified as 'corresponding author' on published papers create and link their Open Researcher and Contributor Identifier (ORCID) with their account on the Manuscript Tracking System (MTS), prior to acceptance. ORCID helps the scientific community achieve unambiguous attribution of all scholarly contributions. You can create and link your ORCID from the home page of the MTS by clicking on 'Modify my Springer Nature account'. For more information please visit please visit www.springernature.com/orcid.

Please do not hesitate to contact me if you have any questions or would like to discuss these revisions further.

We look forward to seeing the revised manuscript and thank you for the opportunity to review your work.

[REDACTED]

Reviewer expertise:

as before

Reviewers' comments:

Reviewer #1 (Remarks to the Author):

The authors did a good job explaining their approach, and I now understand their claims much better. Indeed, if the statistical results are correct (see comments below), this would indicate a social effect on vocal phenotype, which is an important claim. Still, suggesting that this is vocal learning is not

Open Access This file is licensed under a Creative Commons Attribution 4.0 International License, which permits use, sharing, adaptation, distribution and reproduction in any medium or format, as long as you give appropriate credit to the original author(s) and the source, provide a link to the Creative Commons license, and indicate if changes were made. In the cases where the authors are anonymous, such as is the case for the reports of anonymous peer reviewers, author attribution should be to 'Anonymous Referee' followed by a clear attribution to the source work. The images or other third party material in this file are included in the article's Creative Commons license, unless indicated otherwise in a credit line to the material. If material is not included in the article's Creative Commons license and your intended use is not permitted by statutory regulation or exceeds the permitted use, you will need to obtain permission directly from the copyright holder. To view a copy of this license, visit http://creativecommons.org/licenses/by/4.0/.

supported. There can be many mechanisms that would affect vocal repertoire in a manner that is not due to vocal learning. For example, it can be due to hormonal effects. This is something that future studies should figure out, and here, as long as authors remove the claim about vocal learning and present it as an hypothesis, this should be fine.

However, two issues still worries me:

1. I am now even more convinced that the statistical analysis requires adjustment for multiple measurements. Authors are claiming that each model is based on a distinct data set (i.e., entropic measures calculated from different acoustic measures). However, different acoustic measures were all calculated from the same acoustic data set, is this right? Even if these measures were completely orthogonal, if the same acoustic data was used to calculate them, it indicates that p values must be adjusted (this looks like a textbook case to me). So please consult further with your statistician. But I hope some statistical significance will survive. To increase power I suggest using the FDR approach (see: The control of the false discovery rate in multiple testing under dependency Y Benjamini, D Yekutieli Annals of statistics, 1165-1188)

2. As much as I tried, I am still having problems understanding your repertoire entropy measures. These are very complex measures that are based on assumptions that I cannot evaluate unless you elaborate further and show examples. For example, I don’t understand how you define a ‘call variant’. Are you using any threshold to detect such new variants? I think you should show an example of representative sonograms: say some in in high and others low population areas. I wonder if one can see the differences, given how noisy these calls are. Please highlight the new variants on these sonograms, so as to give the reader an intuitive feeling of what your measure actually mean.

Reviewer #2 (Remarks to the Author):

The authors did an extensive rewriting of the manuscript. I commend the authors for addressing most of my (and other reviewers') comments. I have only a couple of observations.

1) The authors should remove passages claiming that they demonstrate vocal learning or lines that suggest similar claims (e.g., lines 44-45).

2) lines 45-47 and 323. Too speculative. There is no evidence in the paper about vocal input playing any role on vocal output, let alone a "deterministic role." The authors should modify the claims to fit the evidence better.

Reviewer #3 (Remarks to the Author):

I was very happy to read this revised version of the manuscript. I found the new additions in response to all of the reviewer's comments to be immensely helpful. My own specific concerns were clearly addressed in this new version and I feel have provided much needed clarity. I no longer have

Open Access This file is licensed under a Creative Commons Attribution 4.0 International License, which permits use, sharing, adaptation, distribution and reproduction in any medium or format, as long as you give appropriate credit to the original author(s) and the source, provide a link to the Creative Commons license, and indicate if changes were made. In the cases where the authors are anonymous, such as is the case for the reports of anonymous peer reviewers, author attribution should be to 'Anonymous Referee' followed by a clear attribution to the source work. The images or other third party material in this file are included in the article’s Creative Commons license, unless indicated otherwise in a credit line to the material. If material is not included in the article’s Creative Commons license and your intended use is not permitted by statutory regulation or exceeds the permitted use, you will need to obtain permission directly from the copyright holder. To view a copy of this license, visit http://creativecommons.org/licenses/by/4.0/.

reservations about recommending this study for publication. Thank you for your efforts in taking the reviews seriously.

Only a couple minor things:

For the abstract, I would recommend to add the total number of orangutan individuals that contributed to this study as (N=106) on line 38 since that is really impressive! It could also be made more explicit in some parts of the results/methods too.

Also for abstract, the sentence starting on line 40 is rather long winded and a bit confusing. I'd suggest rephrasing to separate the two effects more clearly.

********************END********************

Author Rebuttal, first revision:

Reviewers' comments:

Reviewer #1 (Remarks to the Author):

The authors did a good job explaining their approach, and I now understand their claims much better. Indeed, if the statistical results are correct (see comments below), this would indicate a social effect on vocal phenotype, which is an important claim. Still, suggesting that this is vocal learning is not supported. There can be many mechanisms that would affect vocal repertoire in a manner that is not due to vocal learning. For example, it can be due to hormonal effects. This is something that future studies should figure out, and here, as long as authors remove the claim about vocal learning and present it as an hypothesis, this should be fine.

Thank you, we are very glad we have been able to address most of the reviewers' concerns in our new version. We are grateful to the reviewer for the important feedback provided in the previous review round.

The reviewer is correct regarding causal mechanisms. Following the reviewer's recommendation, we have now removed/resolved any passage that directly alluded to vocal learning.

Open Access This file is licensed under a Creative Commons Attribution 4.0 International License, which permits use, sharing, adaptation, distribution and reproduction in any medium or format, as long as you give appropriate credit to the original author(s) and the source, provide a link to the Creative Commons license, and indicate if changes were made. In the cases where the authors are anonymous, such as is the case for the reports of anonymous peer reviewers, author attribution should be to 'Anonymous Referee' followed by a clear attribution to the source work. The images or other third party material in this file are included in the article's Creative Commons license, unless indicated otherwise in a credit line to the material. If material is not included in the article's Creative Commons license and your intended use is not permitted by statutory regulation or exceeds the permitted use, you will need to obtain permission directly from the copyright holder. To view a copy of this license, visit http://creativecommons.org/licenses/by/4.0/.

However, two issues still worries me:

1. I am now even more convinced that the statistical analysis requires adjustment for multiple measurements. Authors are claiming that each model is based on a distinct data set (i.e., entropic measures calculated from different acoustic measures). However, different acoustic measures were all calculated from the same acoustic data set, is this right? Even if these measures were completely orthogonal, if the same acoustic data was used to calculate them, it indicates that p values must be adjusted (this looks like a textbook case to me). So please consult further with your statistician. But I hope some statistical significance will survive. To increase power I suggest using the FDR approach (see: The control of the false discovery rate in multiple testing under dependency Y Benjamini, D Yekutieli Annals of statistics, 1165-1188)

We thank the reviewer for highlighting this important point regarding our analyses. The reviewer is correct; we now confirm that max frequency and duration are indeed uncorrelated (or "orthogonal" as stated by the reviewer), however, given that max frequency and duration were nevertheless extracted from the same acoustic event (i.e. call), the two variables are not independent. As rightly indicated by the reviewer, our analyses require, therefore, p-value adjustment for controlling for false discovery rate (FRD). We are pleased that the reviewer brought the need for this correction procedure to our attention. We now apply a classic FRD correction procedure to our analyses, presented in the Table 1 of the Results section and explained in the Methods section (line 493). (Population density effects remained significant for both frequency-based and time-based repertoire entropy parameters after FRD p adjustment.) On a related aspect, following this reviewer's advice, and for added clarity, we have now removed terms related to statistical significance from the interpretation of control factors.

2. As much as I tried, I am still having problems understanding your repertoire entropy measures. These are very complex measures that are based on assumptions that I cannot evaluate unless you elaborate further and show examples. For example, I don't understand how you define a 'call variant'. Are you using any threshold to detect such new variants? I think you should show an example of representative sonograms: say some in in high and others low population areas. I wonder if one can see the differences, given how noisy these calls are. Please highlight the new variants on these sonograms, so as to give the reader an intuitive feeling of what your measure actually mean.

Following the reviewer's comment, we have now made 4 important edits. First, we now provide "lay(wo)man" examples involving a coin-flip, provided in the R code notebook in our supplementary materials. Second, we direct the reader to further "layperson" examples involving household electric consumption, solar flares and bike-sharing services (line 466). Third, we direct the reader to the open-access "white paper" dedicated to the description of the entropy measures used (line 469). Forth, we direct the reader to

Open Access This file is licensed under a Creative Commons Attribution 4.0 International License, which permits use, sharing, adaptation, distribution and reproduction in any medium or format, as long as you give appropriate credit to the original author(s) and the source, provide a link to the Creative Commons license, and indicate if changes were made. In the cases where the authors are anonymous, such as is the case for the reports of anonymous peer reviewers, author attribution should be to 'Anonymous Referee' followed by a clear attribution to the source work. The images or other third party material in this file are included in the article's Creative Commons license, unless indicated otherwise in a credit line to the material. If material is not included in the article's Creative Commons license and your intended use is not permitted by statutory regulation or exceeds the permitted use, you will need to obtain permission directly from the copyright holder. To view a copy of this license, visit http://creativecommons.org/licenses/by/4.0/.

the open-source package that allows the implementation of these measures across natural and artificial phenomena (in addition to our R code notebook as applied to our datasets).

Regarding the suggestion of using "representative" sonograms, the examples provided by the reviewer would apply to cases of vocal convergence/divergence. This is, however, different from our findings: Repertoire entropy measures characterise variation regimes, not "raw" acoustics as is the case for vocal convergence/divergence. For example, two different individuals may show the same level of entropic emergence in distinct and non-overlapping ranges of the acoustic space. Repertoire entropy profiles are, thus, unique to each individual. An individual's repertoire entropy can only be understood while considering its entire recorded repertoire, it does not apply to "exemplar" calls that could be used as a punctual visual illustration. To address this point raised by the reviewer, the manuscript version explicitly clarifies the difference between cases of vocal convergence/divergence and cases of repertoire entropy (line 457).

Reviewer #2 (Remarks to the Author):

The authors did an extensive rewriting of the manuscript. I commend the authors for addressing most of my (and other reviewers') comments. I have only a couple of observations.

Thank you, we are very glad we have been able to address the reviewer's concerns and adequately incorporate the important feedback and commendations received in the previous review round.

1) The authors should remove passages claiming that they demonstrate vocal learning or lines that suggest similar claims (e.g., lines 44-45).

This is a germane observation. The target sentence has now been rephrased following the reviewer's request.

2) lines 45-47 and 323. Too speculative. There is no evidence in the paper about vocal input playing any role on vocal output, let alone a "deterministic role." The authors should modify the claims to fit the evidence better.

This is a relevant observation. The target sentences have now been rephrased following the reviewer's request.

Open Access This file is licensed under a Creative Commons Attribution 4.0 International License, which permits use, sharing, adaptation, distribution and reproduction in any medium or format, as long as you give appropriate credit to the original author(s) and the source, provide a link to the Creative Commons license, and indicate if changes were made. In the cases where the authors are anonymous, such as is the case for the reports of anonymous peer reviewers, author attribution should be to 'Anonymous Referee' followed by a clear attribution to the source work. The images or other third party material in this file are included in the article's Creative Commons license, unless indicated otherwise in a credit line to the material. If material is not included in the article's Creative Commons license and your intended use is not permitted by statutory regulation or exceeds the permitted use, you will need to obtain permission directly from the copyright holder. To view a copy of this license, visit http://creativecommons.org/licenses/by/4.0/.

Reviewer #3 (Remarks to the Author):

I was very happy to read this revised version of the manuscript. I found the new additions in response to all of the reviewer's comments to be immensely helpful. My own specific concerns were clearly addressed in this new version and I feel have provided much needed clarity. I no longer have reservations about recommending this study for publication. Thank you for your efforts in taking the reviews seriously.

Thank you, we are thankful for the support and constructive input provided by the reviewer throughout, which we feel has greatly helped improve the manuscript.

Only a couple minor things:

For the abstract, I would recommend to add the total number of orangutan individuals that contributed to this study as (N=106) on line 38 since that is really impressive! It could also be made more explicit in some parts of the results/methods too.

We have now added sample sizes to the Abstract and Results sections following the reviewer's recommendation.

Also for abstract, the sentence starting on line 40 is rather long winded and a bit confusing. I'd suggest rephrasing to separate the two effects more clearly.

Corrected accordingly.

Decision Letter, second revision:

Open Access This file is licensed under a Creative Commons Attribution 4.0 International License, which permits use, sharing, adaptation, distribution and reproduction in any medium or format, as long as you give appropriate credit to the original author(s) and the source, provide a link to the Creative Commons license, and indicate if changes were made. In the cases where the authors are anonymous, such as is the case for the reports of anonymous peer reviewers, author attribution should be to 'Anonymous Referee' followed by a clear attribution to the source work. The images or other third party material in this file are included in the article's Creative Commons license, unless indicated otherwise in a credit line to the material. If material is not included in the article's Creative Commons license and your intended use is not permitted by statutory regulation or exceeds the permitted use, you will need to obtain permission directly from the copyright holder. To view a copy of this license, visit http://creativecommons.org/licenses/by/4.0/.

20th December 2021

Dear Dr. Lameira,

Thank you for submitting your revised manuscript "Sociality predicts orangutan vocal phenotype" (NATECOLEVOL-210814385B). It has now been seen again by the original reviewers and their comments are below. The reviewers find that the paper has improved in revision, and therefore we'll be happy in principle to publish it in Nature Ecology & Evolution, pending minor revisions to satisfy the reviewers' final requests and to comply with our editorial and formatting guidelines.

If the current version of your manuscript is in a PDF format, please email us a copy of the file in an editable format (Microsoft Word or LaTex)-- we can not proceed with PDFs at this stage.

We are now performing detailed checks on your paper and will send you a checklist detailing our editorial and formatting requirements in about a week (note--this is our usual target but given the time of year we are working with a skeleton staff so it may actually be January before we can send this to you). Please do not upload the final materials and make any revisions until you receive this additional information from us.

Thank you again for your interest in Nature Ecology & Evolution. Please do not hesitate to contact me if you have any questions.

[REDACTED]

Reviewer #1 (Remarks to the Author):

All my concerns are now fully addressed. I have no further comments. I am looking forward to see this study published.

Our ref: NATECOLEVOL-210814385B

12th January 2022

Dear Dr. Lameira,

Open Access This file is licensed under a Creative Commons Attribution 4.0 International License, which permits use, sharing, adaptation, distribution and reproduction in any medium or format, as long as you give appropriate credit to the original author(s) and the source, provide a link to the Creative Commons license, and indicate if changes were made. In the cases where the authors are anonymous, such as is the case for the reports of anonymous peer reviewers, author attribution should be to 'Anonymous Referee' followed by a clear attribution to the source work. The images or other third party material in this file are included in the article's Creative Commons license, unless indicated otherwise in a credit line to the material. If material is not included in the article's Creative Commons license and your intended use is not permitted by statutory regulation or exceeds the permitted use, you will need to obtain permission directly from the copyright holder. To view a copy of this license, visit http://creativecommons.org/licenses/by/4.0/.

Thank you for your patience as we've prepared the guidelines for final submission of your Nature Ecology & Evolution manuscript, "Sociality predicts orangutan vocal phenotype" (NATECOLEVOL-210814385B). Please carefully follow the step-by-step instructions provided in the attached file, and add a response in each row of the table to indicate the changes that you have made. Please also check and comment on any additional marked-up edits we have proposed within the text. Ensuring that each point is addressed will help to ensure that your revised manuscript can be swiftly handed over to our production team.

We would like to start working on your revised paper, with all of the requested files and forms, as soon as possible (preferably within two weeks). Please get in contact with us immediately if you anticipate it taking more than two weeks to submit these revised files.

When you upload your final materials, please include a point-by-point response to any remaining reviewer comments.

If you have not done so already, please alert us to any related manuscripts from your group that are under consideration or in press at other journals, or are being written up for submission to other journals (see: https://www.nature.com/nature-research/editorial-policies/plagiarism#policy-on-duplicate-publication for details).

In recognition of the time and expertise our reviewers provide to Nature Ecology & Evolution's editorial process, we would like to formally acknowledge their contribution to the external peer review of your manuscript entitled "Sociality predicts orangutan vocal phenotype". For those reviewers who give their assent, we will be publishing their names alongside the published article.

Nature Ecology & Evolution offers a Transparent Peer Review option for new original research manuscripts submitted after December 1st, 2019. As part of this initiative, we encourage our authors to support increased transparency into the peer review process by agreeing to have the reviewer comments, author rebuttal letters, and editorial decision letters published as a Supplementary item. When you submit your final files please clearly state in your cover letter whether or not you would like to participate in this initiative. Please note that failure to state your preference will result in delays in accepting your manuscript for publication.

Cover suggestions

As you prepare your final files we encourage you to consider whether you have any images or illustrations that may be appropriate for use on the cover of Nature Ecology & Evolution.

Covers should be both aesthetically appealing and scientifically relevant, and should be supplied at the best quality available. Due to the prominence of these images, we do not generally select images featuring faces, children, text, graphs, schematic drawings, or collages on our covers.

We accept TIFF, JPEG, PNG or PSD file formats (a layered PSD file would be ideal), and the image should be at least 300ppi resolution (preferably 600-1200 ppi), in CMYK colour mode.

Open Access This file is licensed under a Creative Commons Attribution 4.0 International License, which permits use, sharing, adaptation, distribution and reproduction in any medium or format, as long as you give appropriate credit to the original author(s) and the source, provide a link to the Creative Commons license, and indicate if changes were made. In the cases where the authors are anonymous, such as is the case for the reports of anonymous peer reviewers, author attribution should be to 'Anonymous Referee' followed by a clear attribution to the source work. The images or other third party material in this file are included in the article's Creative Commons license, unless indicated otherwise in a credit line to the material. If material is not included in the article's Creative Commons license and your intended use is not permitted by statutory regulation or exceeds the permitted use, you will need to obtain permission directly from the copyright holder. To view a copy of this license, visit http://creativecommons.org/licenses/by/4.0/.

If your image is selected, we may also use it on the journal website as a banner image, and may need to make artistic alterations to fit our journal style.

Please submit your suggestions, clearly labeled, along with your final files. We'll be in touch if more information is needed.

Nature Ecology & Evolution has now transitioned to a unified Rights Collection system which will allow our Author Services team to quickly and easily collect the rights and permissions required to publish your work. Approximately 10 days after your paper is formally accepted, you will receive an email in providing you with a link to complete the grant of rights. If your paper is eligible for Open Access, our Author Services team will also be in touch regarding any additional information that may be required to arrange payment for your article.

Please note that <i>Nature Ecology & Evolution</i> is a Transformative Journal (TJ). Authors may publish their research with us through the traditional subscription access route or make their paper immediately open access through payment of an article-processing charge (APC). Authors will not be required to make a final decision about access to their article until it has been accepted. Find out more about Transformative Journals

<B>Authors may need to take specific actions to achieve compliance with funder and institutional open access mandates. For submissions from January 2021, if your research is supported by a funder that requires immediate open access (e.g. according to Plan S principles) then you should select the gold OA route, and we will direct you to the compliant route where possible. For authors selecting the subscription publication route our standard licensing terms will need to be accepted, including our self-archiving policies. Those standard licensing terms will supersede any other terms that the author or any third party may assert apply to any version of the manuscript.

Please note that you will not receive your proofs until the publishing agreement has been received through our system.

For information regarding our different publishing models please see our Transformative Journals page. If you have any questions about costs, Open Access requirements, or our legal forms, please contact ASJournals@springernature.com.

Please use the following link for uploading these materials:
[REDACTED]

Open Access This file is licensed under a Creative Commons Attribution 4.0 International License, which permits use, sharing, adaptation, distribution and reproduction in any medium or format, as long as you give appropriate credit to the original author(s) and the source, provide a link to the Creative Commons license, and indicate if changes were made. In the cases where the authors are anonymous, such as is the case for the reports of anonymous peer reviewers, author attribution should be to 'Anonymous Referee' followed by a clear attribution to the source work. The images or other third party material in this file are included in the article's Creative Commons license, unless indicated otherwise in a credit line to the material. If material is not included in the article's Creative Commons license and your intended use is not permitted by statutory regulation or exceeds the permitted use, you will need to obtain permission directly from the copyright holder. To view a copy of this license, visit http://creativecommons.org/licenses/by/4.0/.

If you have any further questions, please feel free to contact me.

[REDACTED]

Reviewer #1:
Remarks to the Author:
All my concerns are now fully addressed. I have no further comments. I am looking forward to see this study published.

Final Decision Letter:

2nd February 2022

Dear Adriano,

We are pleased to inform you that your Article entitled "Sociality predicts orangutan vocal phenotype", has now been accepted for publication in Nature Ecology & Evolution.

Over the next few weeks, your paper will be copyedited to ensure that it conforms to Nature Ecology and Evolution style. Once your paper is typeset, you will receive an email with a link to choose the appropriate publishing options for your paper and our Author Services team will be in touch regarding any additional information that may be required

After the grant of rights is completed, you will receive a link to your electronic proof via email with a request to make any corrections within 48 hours. If, when you receive your proof, you cannot meet this deadline, please inform us at rjsproduction@springernature.com immediately.

You will not receive your proofs until the publishing agreement has been received through our system

Due to the importance of these deadlines, we ask you please us know now whether you will be difficult to contact over the next month. If this is the case, we ask you provide us with the contact information (email, phone and fax) of someone who will be able to check the proofs on your behalf, and who will be available to address any last-minute problems . Once your paper has been scheduled for online publication, the Nature press office will be in touch to confirm the details.

Acceptance of your manuscript is conditional on all authors' agreement with our publication policies (see www.nature.com/authors/policies/index.html). In particular your manuscript must not be published elsewhere and there must be no announcement of the work to any media outlet until the publication date (the day on which it is uploaded onto our web site).

Please note that <i>Nature Ecology & Evolution</i> is a Transformative Journal (TJ). Authors may

Open Access This file is licensed under a Creative Commons Attribution 4.0 International License, which permits use, sharing, adaptation, distribution and reproduction in any medium or format, as long as you give appropriate credit to the original author(s) and the source, provide a link to the Creative Commons license, and indicate if changes were made. In the cases where the authors are anonymous, such as is the case for the reports of anonymous peer reviewers, author attribution should be to 'Anonymous Referee' followed by a clear attribution to the source work. The images or other third party material in this file are included in the article's Creative Commons license, unless indicated otherwise in a credit line to the material. If material is not included in the article's Creative Commons license and your intended use is not permitted by statutory regulation or exceeds the permitted use, you will need to obtain permission directly from the copyright holder. To view a copy of this license, visit http://creativecommons.org/licenses/by/4.0/.

publish their research with us through the traditional subscription access route or make their paper immediately open access through payment of an article-processing charge (APC). Authors will not be required to make a final decision about access to their article until it has been accepted. Find out more about Transformative Journals

<B>Authors may need to take specific actions to achieve compliance with funder and institutional open access mandates. For submissions from January 2021, if your research is supported by a funder that requires immediate open access (e.g. according to Plan S principles) then you should select the gold OA route, and we will direct you to the compliant route where possible. For authors selecting the subscription publication route our standard licensing terms will need to be accepted, including our self-archiving policies. Those standard licensing terms will supersede any other terms that the author or any third party may assert apply to any version of the manuscript.

In approximately 10 business days you will receive an email with a link to choose the appropriate publishing options for your paper and our Author Services team will be in touch regarding any additional information that may be required.

You will not receive your proofs until the publishing agreement has been received through our system.

If you have any questions about our publishing options, costs, Open Access requirements, or our legal forms, please contact ASJournals@springernature.com

An online order form for reprints of your paper is available at https://www.nature.com/reprints/author-reprints.html. All co-authors, authors' institutions and authors' funding agencies can order reprints using the form appropriate to their geographical region.

We welcome the submission of potential cover material (including a short caption of around 40 words) related to your manuscript; suggestions should be sent to Nature Ecology & Evolution as electronic files (the image should be 300 dpi at 210 x 297 mm in either TIFF or JPEG format). Please note that such pictures should be selected more for their aesthetic appeal than for their scientific content, and that colour images work better than black and white or grayscale images. Please do not try to design a cover with the Nature Ecology & Evolution logo etc., and please do not submit composites of images related to your work. I am sure you will understand that we cannot make any promise as to whether any of your suggestions might be selected for the cover of the journal.

You can now use a single sign-on for all your accounts, view the status of all your manuscript submissions and reviews, access usage statistics for your published articles and download a record of your refereeing activity for the Nature journals.

Open Access This file is licensed under a Creative Commons Attribution 4.0 International License, which permits use, sharing, adaptation, distribution and reproduction in any medium or format, as long as you give appropriate credit to the original author(s) and the source, provide a link to the Creative Commons license, and indicate if changes were made. In the cases where the authors are anonymous, such as is the case for the reports of anonymous peer reviewers, author attribution should be to 'Anonymous Referee' followed by a clear attribution to the source work. The images or other third party material in this file are included in the article's Creative Commons license, unless indicated otherwise in a credit line to the material. If material is not included in the article's Creative Commons license and your intended use is not permitted by statutory regulation or exceeds the permitted use, you will need to obtain permission directly from the copyright holder. To view a copy of this license, visit http://creativecommons.org/licenses/by/4.0/.

To assist our authors in disseminating their research to the broader community, our SharedIt initiative provides you with a unique shareable link that will allow anyone (with or without a subscription) to read the published article. Recipients of the link with a subscription will also be able to download and print the PDF.

You can generate the link yourself when you receive your article DOI by entering it here: <a href="http://authors.springernature.com/share">http://authors.springernature.com/share<a>.

[REDACTED]

P.S. Click on the following link if you would like to recommend Nature Ecology & Evolution to your librarian http://www.nature.com/subscriptions/recommend.html#forms

** Visit the Springer Nature Editorial and Publishing website at www.springernature.com/editorial-and-publishing-jobs for more information about our career opportunities. If you have any questions please click here.**

Open Access This file is licensed under a Creative Commons Attribution 4.0 International License, which permits use, sharing, adaptation, distribution and reproduction in any medium or format, as long as you give appropriate credit to the original author(s) and the source, provide a link to the Creative Commons license, and indicate if changes were made. In the cases where the authors are anonymous, such as is the case for the reports of anonymous peer reviewers, author attribution should be to 'Anonymous Referee' followed by a clear attribution to the source work. The images or other third party material in this file are included in the article's Creative Commons license, unless indicated otherwise in a credit line to the material. If material is not included in the article's Creative Commons license and your intended use is not permitted by statutory regulation or exceeds the permitted use, you will need to obtain permission directly from the copyright holder. To view a copy of this license, visit http://creativecommons.org/licenses/by/4.0/.
