# Supplementary material for: Author Correction: Sociality predicts orangutan vocal phenotype
Source: Nat Ecol Evol. 2025 Dec 18;10(1):154–5. doi: 10.1038/s41559-025-02954-7 (PMC12789004; doi:10.1038/s41559-025-02954-7)
Supplement: Supplementary file 1 — Original, uncorrected article [file 41559_2025_2954_MOESM1_ESM.pdf]

nature ecology & evolution

Supplementary information

Author Correction: Sociality predicts orangutan vocal phenotype

In the format provided by the authors and unedited

Original uncorrected article

ARTICLES
https://doi.org/10.1038/s41559-022-01689-z
nature ecology & evolution

Check for updates

OPEN

Sociality predicts orangutan vocal phenotype

Adriano R. Lameira[1,2] ✉, Guillermo Santamaría-Bonfil[3], Deborah Galeone[4], Marco Gamba[4], Madeleine E. Hardus[5], Cheryl D. Knott[6], Helen Morrogh-Bernard[7,8], Matthew G. Nowak[9,10], Gail Campbell-Smith[11] and Serge A. Wich[12,13]

In humans, individuals' social setting determines which and how language is acquired. Social seclusion experiments show that sociality also guides vocal development in songbirds and marmoset monkeys, but absence of similar great ape data has been interpreted as support to saltational notions for language origin, even if such laboratorial protocols are unethical with great apes. Here we characterize the repertoire entropy of orangutan individuals and show that in the wild, different degrees of sociality across populations are associated with different 'vocal personalities' in the form of distinct regimes of alarm call variants. In high-density populations, individuals are vocally more original and acoustically unpredictable but new call variants are short lived, whereas individuals in low-density populations are more conformative and acoustically consistent but also exhibit more complex call repertoires. Findings provide non-invasive evidence that sociality predicts vocal phenotype in a wild great ape. They prove false hypotheses that discredit great apes as having hardwired vocal development programmes and non-plastic vocal behaviour. Social settings mould vocal output in hominids besides humans.

Humans acquire language from their individual linguistic communities. Experiments manipulating individuals' social setting—from solitary social isolation to social grouping—have demonstrated that the degree of sociality experienced by songbirds[1–6] and marmoset monkeys[7–11] also determines how their vocal repertoire develops and matures. These findings have made these species favoured lab models for the study of (spoken) language evolution[12,13]. However, evolution is a path-dependent process that builds upon a lineage's biology and behaviour, where homology is critical for the reconstruction of ancestral states and insight into their ensuing evolution. Given that songbirds and marmosets are distantly related to our own phylogenetic family, without similar data from our closest living relatives—the (nonhuman) great apes—our understanding of why language transpired in our own clade but none other in 525 million years of vertebrate evolution will probably remain imperfect.

Laboratorial protocols involving solitary social isolation as conducted with songbirds and marmosets are not, however, ethically permissible with great apes. Personhood rights may extend to these species[14–16], and their survival status in the wild is critical[17–22] (International Union for Conservation of Nature, Red List of Threatened Species, 2021). In the absence of evidence from social manipulation experiments, great ape vocal phenotype has been presumed siloed from social influence, and their vocal production and repertoire posited as innate, automatic and hardwired[23–25]. Enigmatically, these notions fundamentally contradict the role of shared ancestry in biological evolution and lead to notions of language emergence as a non-continuous process[23,24,26,27]. These traditional notions derive in part from historical great ape language projects[28–31], which reportedly failed to teach great apes to speak. Paradoxically, however, their study subjects lived in home labs with impoverished (if any) social contact with conspecifics[32,33]. While positive evidence from these individuals' capacities (that is, 'things they *can* do') can be instrumental for improved heuristics of human evolution[34–38], negative evidence (that is, 'things they *cannot* do') is not generalizable[33]. Indeed, several recent human–ape interactional experiments in accredited zoos have now demonstrated that great apes exert fine real-time voluntary control over all the necessary structures required for speech production, including laryngeal control[35–37,39], that their repertoire is composed by vowel-like and consonant-like calls[33,40–44] and that they can produce these calls with a speech-like rhythm[34,45]. A new framework for the *gradual* evolution of spoken language in the human clade from an ancestral hominid repertoire and vocal system is, therefore, gaining predominance[42,46–56].

The last limitation in this growing body of evidence and the view that great apes are highly desirable models for language evolution research is arguably the fact that most data for vocal (production) learning have thus far derived from captivity[35–37,39,52,53,57–59] (cf. [60,61]). Individuals' social setting in captivity is artificial and relatively monotonous and therefore limits the full expression of animals' natural predispositions and potential phenotypes, making data from the wild paramount. There is extensive evidence for social learning across behaviour domains and for different types of great ape culture in the wild[62–65]. Although most research effort has focused of material cultures, there is no theoretical reason to believe that social effects would operate in starkly different ways with vocal and communicative behaviour. Great ape vocal research in the wild is inherently difficult and time intensive, but evidence for local traditions in sound communication[66–69] and call cultures[46,60,61,70] is steadily accumulating across great ape genera, even if great ape behavioural richness is eroding with human impact, and multiple local traditions should be assumed already extinct[71,72].

[1]Department of Psychology, University of Warwick, Coventry, UK. [2]School of Psychology and Neuroscience, University of St Andrews, St Andrews, UK. [3]Instituto Nacional de Electricidad y Energías Limpias, Gerencia de Tecnologías de la Información, Cuernavaca, México. [4]Department of Life Sciences and Systems Biology, University of Torino, Turin, Italy. [5]Independent researcher, Warwick, UK. [6]Department of Anthropology, Boston University, Boston, MA, USA. [7]Borneo Nature Foundation, Palangka Raya, Indonesia. [8]College of Life and Environmental Sciences, University of Exeter, Penryn, UK. [9]The PanEco Foundation—Sumatran Orangutan Conservation Programme, Berg am Irchel, Switzerland. [10]Department of Anthropology, Southern Illinois University, Carbondale, IL, USA. [11]Yayasan Inisiasi Alam Rehabilitasi Indonesia, International Animal Rescue, Ketapang, Indonesia. [12]School of Natural Sciences and Psychology, Liverpool John Moores University, Liverpool, UK. [13]Faculty of Science, University of Amsterdam, Amsterdam, Netherlands. ✉e-mail: adriano.lameira@warwick.ac.uk

To assess the influence of sociality on great ape vocal phenotype and resolve the existing empirical deadlock in the field of language origin and evolution, here we transpose from the artificial setting of the laboratory to the natural social arena of the wild and embark on the largest cross-populational analyses conduced in great ape vocal research to date. We capitalize on 'natural experiments' that have exposed wild orangutans to different degrees of sociality as residents of populations with different orangutan densities. According to the traditional hypothesis that great apes are incapable of vocal (production) learning and poor models of language evolution research[23–25] (cf. [28,29]), individuals should operate as independent agents and their vocal phenotype should take course without influence of social and vocal input. If the traditional hypothesis is correct, one should expect that natural differences in sociality between wild great apes should show *no* correlation with the gamut and acoustic range of call variants produced by great ape individuals.

Rationale

Transposing experimentally from songbirds and marmosets in the lab to great apes in the wild requires accounting for three major issues: social proxies, study designs and socio-ecological confounds.

Social proxy: populational density. Orangutans exhibit a fission–fusion social system without permanent social groups (besides long-term mother–infant associations)[73,74] and instead tend to organize in loose female communities with roving adults males[75,76]. This type of social organization typically leads to the exclusion of orangutans from cross-species comparisons because social measures used with other primates simply do not apply[77]. Hence, the degree of sociality here—capturing the probability for social and vocal input—was measured by the number of individuals per unit of area (km^2) at each population (that is, orangutan density)[78]. Indeed, higher orangutan densities are associated with higher average percentage of time spent with other independent conspecifics[79]. At the same time, if the opposite were true (that is, higher density without higher social contact), one would predict diminishing home range sizes, which is not observed; higher population densities are associated with more females sharing larger expanses of their home ranges[80]. This confirms overall that density can be used as a surrogate and operable metric of sociality with wild orangutans.

Study design: from longitudinal to cross-sectional. In the lab, studies conducted with songbirds and marmosets have been longitudinal, where infants' vocal development is closely followed through time. In these studies, the effect of social vocal input as a catalyst of vocal changes has been assessed through the measure of a single call's acoustic entropy. This parameter gauges the level of disorder in a sound by analysing a call's energy distribution. Comparing acoustic entropy across time allows for tracking how an individual hones a call's mature/adult/tutored version. But this requires extensive and regular recordings of an individual's vocal behaviour, best achieved with a rapidly developing species in a fixed and predictable environment. Moreover, acoustic entropy is highly sensitive to ambient noise, which can tamper with measures of acoustic energy distribution by adding spurious energy bursts, peak or bands. This requires recordings to be collected in low and/or constant levels of background noise and unchanging acoustic settings.

Conversely, great apes exhibit the slowest development, reproduction rates and generational turnover among the extant primate species with orangutans' life history being slower than that of humans[81–83]. Very few long-term field sites have been able to operate uninterruptedly and follow the development of specific individuals as they age[84–86]. Alas, currently, there is no available audio database spanning years of observation at the same location for orangutans or any other great ape. In addition, great ape observation in the wild is not under human control in a similar way as experiments are and must adhere to strict guidelines to assure that individuals remain wild. For example, in orangutan habitat, noise levels and acoustic settings are constant, variable and unpredictable, rendering unreliable any analyses based on acoustic entropy. Moreover, to avoid human over-habituation, an orangutan focal individual can be followed for only 5–10 days, after which they cannot be followed for another month with no expectation of when or whether they will be encountered again. This inherently renders unviable any attempt to systematically and regularly follow individual vocal behaviour and development. The wild thus poses contrasting opportunities and conditions for audio recordings in comparison with captivity; data collection is noise laden, sporadic, opportunistic and cross-sectional.

As such, to surpass the limitations imposed by lab-based methods when applied to the wild, we characterize orangutan vocal phenotypes by measuring individuals' 'repertoire entropy'. Repertoire entropy was calculated across an individual's call repertoire (instead of individuals' single calls as for acoustic entropy) using three entropic parameters: emergence, self-organization and complexity[87,88]. Each of these parameters gauges the distribution probability of novel or conserved call variants within a given set of calls produced by an individual, expressing the variation regime within that repertoire. Accordingly, these parameters do not measure 'raw acoustics' (as in acoustic entropy), but the rate at which calls with similar/distinct acoustics occur. Emergence defines the rate at which new acoustic states (a call variant) appear in a system (an individual's call set/repertoire), with higher values expressing higher rates of original/generative vocal production and vice versa. Self-organization defines the rate at which similar acoustic states appear in an individual's repertoire, with higher values expressing higher rates of conserved/conformist vocal production and vice versa, where self-organization is inversely proportional to emergence. Complexity defines the balance level between emergence and self-organization in an individual's repertoire; when new acoustic states emerge and are subsequently preserved through repetition (that is, conserved vocal production), over time, that system raises its average number of different states and, hence, its complexity (Supplementary Data 5).

Socio-ecological confounds: ecological. In the lab, different populations can exist and survive in different demographic densities accompanied by virtually no variation in ecological setting. This is because individuals' nutritional and energetic requirements are met by human artificial food provisioning. Conversely, in the wild, high-density populations will probably emerge in ecological habitats inherently more productive. Accordingly, food calls could be potentially affected by or reflect ecological differences between populations instead of differences in sociality between individuals. Therefore, food calls should not be considered for analyses of repertoire entropy. Unlike other great apes[89–92], orangutans do not produce food calls[93], but flanged male orangutans can long call upon arrival at a food patch, and so long calls and, conservatively, other call types exclusive to flanged males should also be excluded.

It has also been experimentally demonstrated that forests with different levels of plant productivity (for example, Sumatra vs. Borneo[94]) and different structural architecture (for example, low mountain rainforest vs. peat swamp) affect sound and information propagation of different orangutan call types in *similar* fashion[47,95]. Effects due to ecological differences in habitat physical structure can, thus, also be assumed absent or negligible between different areas of orangutan territory.

Socio-ecological confounds: social. In the lab, individuals can be socially 'staged' so they can establish vocal contact with others *without* social contact. This assures that call variation reflects the degree of vocal input instead of the kind of social interaction. In the wild, vocal input and social contact are, however, often inseparable.

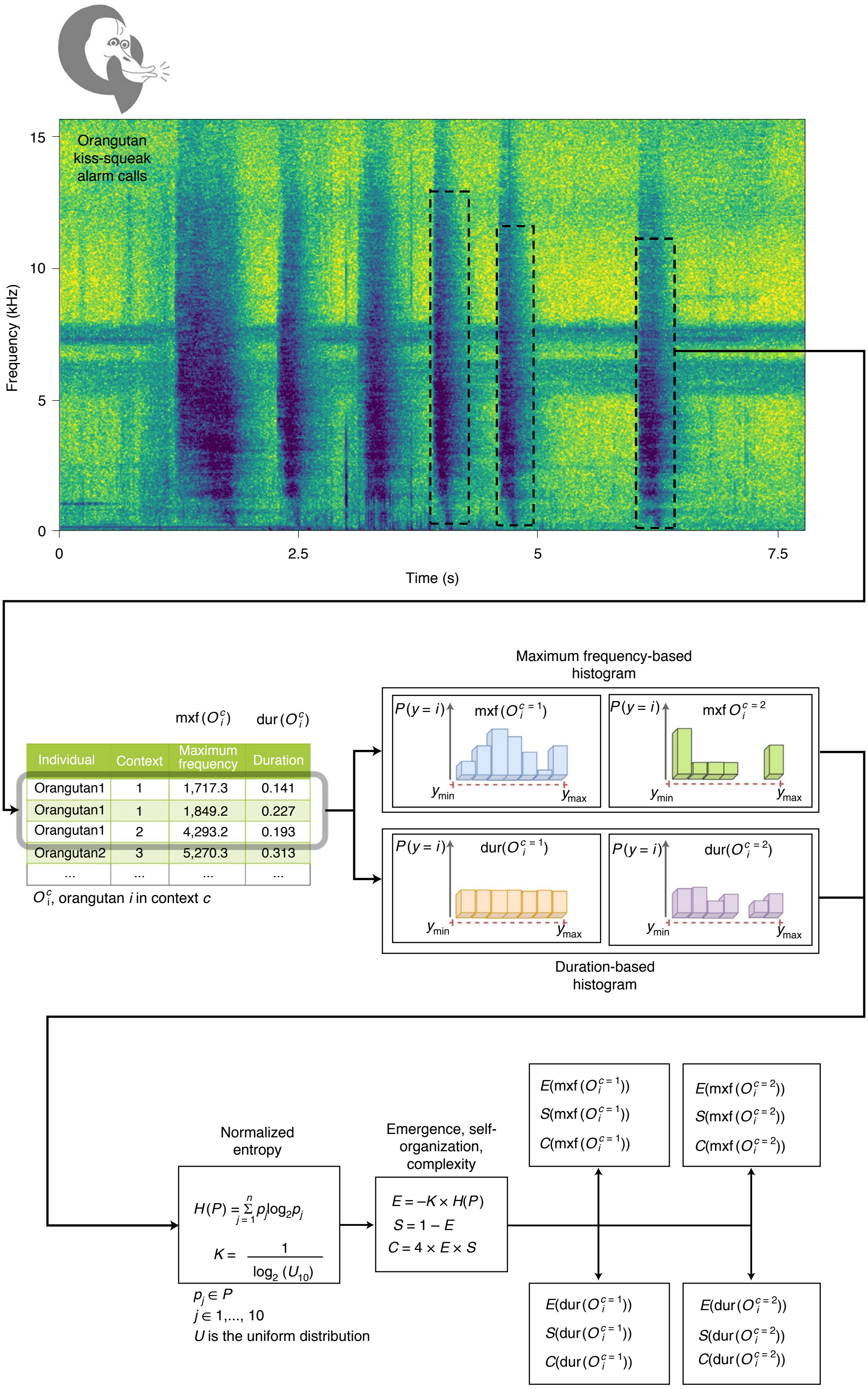

Fig. 1 | Spectrographic representation of orangutan kiss-squeak alarm calls and analytical flow chart. Spectrographic representation of six orangutan kiss-squeaks, where darker colours denote louder sound frequencies. Dashed lines indicate the manual selection from which kiss-squeak maximum frequency (mxf) and duration (dur) were extracted, and how the two acoustic parameters were them processed to calculcate their corresponding entropy parameters per individual per context, where *E* is emergence, *S* is self-organization and *C* is complexity. *P* and *p* are probabilities, *K* is a constraint that constrains *E*, *S* and *C*, *H* is normalized entropy and *y* represents a call variant. (Methods and Supplementary Data 5).

Table 1 | Analysis of variance (ANOVA) summary results for linear mixed models based on repertoire entropy parameters

	Maximum frequency						Duration					
	Emergence/self-organization			Complexity			Emergence/self-organization			Complexity		
Effect	d.f.	F	P	d.f.	F	P	d.f.	F	P	d.f.	F	P
sex	1, 58	2.685	0.107	1, 57.40	0.637	0.428	1, 52.69	0.209	0.649	1, 56.87	1.586	0.213
age–sex	4, 58	0.670	0.616	4, 55.64	1.018	0.406	4, 50.00	0.986	0.424	4, 51.54	1.161	0.339
context	3, 58	1.188	0.322	3, 31.20	1.504	0.233	3, 7.28	3.013	0.101	3, 20.21	3.117	0.049
species	1, 58	3.860	0.054	1, 53.32	6.288	0.015	1, 46.80	1.621	0.209	1, 45.37	0.548	0.463
density	**1, 58**	**4.469**	**0.039**[a]	**1, 54.15**	**11.766**	**0.001**[b]	**1, 47.69**	**8.472**	**0.005**[c]	**1, 47.60**	**4.989**	**0.030**[d]

Satterthwaite test model, type III sum of squares, two-sided. See fit statistics, samples sizes, fixed effects estimates and estimated marginal means in Supplementary Data 4. Adjusted *P* values for false discovery rate (Hochberg correction): [a]$P=0.039$, [b]$P=0.004$, [c]$P=0.015$, [d]$P=0.039$. **Bold** denotes significant effects after adjustment (applied to variable of interest only, that is, density).

Consequently, it is conceivable that living in high-density populations could lead individuals to engage in different types of social contact and, hence, different types of vocal interaction. Accordingly, social calls could potentially be affected by or reflect differences in social interaction between individuals instead of degree of vocal input. Therefore, social calls exchanged between conspecifics should not be considered for analyses of repertoire entropy.

Orangutans also exhibit call cultures in the wild[60,67,68,93]. These are not instances of geographic variation in the same call type[46] as reported across primates and other mammals[96]. Notably, some orangutan call types are exclusive to one population, whilst other populations exhibit an acoustically distinct 'synonym' call type produced in the same context and function, whereas other populations exhibit no vocal signal for that same context or function. Currently known cultural calls include (mother–infant) social contact calls and calls produced during nest construction[60]. Because these call types are local specific, they should also be excluded from analyses.

Final empirical setup

Accordingly, to prevent ecological and social confounding effects, we analysed orangutans' primary alarm call, the kiss-squeak[93]. This call type is universal across, and prevalent within, every wild population studied thus far. It is one of the most frequently produced calls by wild orangutans, providing relatively ample sampling, notably, towards human observers—a context virtually equal across populations and de-correlated from any orangutan social, ecological or demographic variables. Kiss-squeaks are predator-oriented alarm calls[49,67] and produced comparably by populations exposed to different predator guilds[97]. (Occasionally, they can be given towards other orangutans; thus, these cases should also be excluded from analyses (Methods).) Kiss-squeaks carry over dense forests up to 100 m without losing informational content[47] and thus can be detected, heard and monitored by conspecifics who are within earshot but not interacting socially with the senders. Kiss-squeaks provide, thus, a rare occasion in the wild where vocal input is neither socially motivated nor inextricable from social interaction, further liberating analyses from possible social confounds.

In sum, to study the effects of sociality on the expression of the orangutan vocal phenotypes in the wild, we used a two-island cross-populational cross-sectional study design. We assessed individual vocal phenotypes by calculating repertoire entropy for each individual's kiss-squeak repertoire ($N_{\text{individuals}}=76$; $N_{\text{calls}}=5{,}290$; $N_{\text{populations}}=6$; $N_{\text{observation hours}}>6{,}120$; Fig. 1, Supplementary Data 1). Namely, we calculated entropic emergence, self-organization and complexity (Fig. 1, Methods and Supplementary Data 5) based on maximum call frequency (Hz; that is, that of highest dB; $N=69$) and duration (s; $N=69$) separately for each individual per context (Fig. 1, Methods and Supplementary Data 2, 3 and 5). To quantify the effect of sociality on repertoire entropy, we conducted four linear mixed models, each with one of the entropic measures as a response variable (2 frequency-based + 2 time-based; 2 for emergence/self-organization + 2 for complexity). Each model included sex (two levels: female, male), age–sex class (five levels: infant, adolescent, adult female with infant, unflanged male, flanged male), species (two levels: Bornean, Sumatran), context (four levels: towards: observers, animals, humans (non-observers), no apparent danger) as control fixed factors and orangutan density as our main factor of interest. Individual ID was included as a random effect to weigh out individuals contributing several data points (Methods and Supplementary Data 4).

Results and discussion

Orangutan density—a surrogate measure for degree of sociality—had a preponderate effect on individuals' repertoire entropy (Table 1 and Fig. 2), rejecting the traditional hypothesis that great ape vocal phenotype is impervious to social settings. Frequency-based and time-based emergence (that is, 'rate of original calls') and self-organization (that is, 'rate of repetitive calls') were significantly correlated (positively and negatively, respectively) with orangutan density. That is, across six wild populations, individuals living in higher densities were vocally more original and acoustically more unpredictable than individuals living in lower densities, who instead were vocally more repetitive and acoustically more conformative. Additionally, frequency-based and time-based complexity were significantly correlated with orangutan density with individuals living in low densities exhibiting more complex call repertoires than those living in higher density populations (Table 1 and Fig. 2). It should be noted that these relationships were not an artifact of a smaller number of individuals or calls sampled in the low-density populations or vice versa but instead features of signal variation per individual per context (Methods and Supplementary Data 5).

For frequency-based repertoire entropy, species was the control factor with the strongest effect (Table 1 and Supplementary Data 4). Bornean orangutans were vocally more original and exhibited a more complex repertoire than Sumatran (Supplementary Data 4), which could reflect overall higher forest productivity in Sumatran (hence, higher orangutan densities) than in Borneo[94]. For time-based repertoire entropy, call context was the control factor with the strongest effect (Table 1); however, there were no substantial differences between contexts (Supplementary Data 4).

Results show that an orangutan's 'vocal personality'—being vocally original vs. vocally confirmative—was predicted by that individual's degree of sociality. This effect pertained to alarm calls directed to potential danger and excluded calls produced towards other orangutans. Strictly limiting our analyses to these calls allowed us to curtail possible socio-ecological confounds. Findings show that even in the absence of social interaction or direct vocal exchange, the weight of an individual's social and vocal landscape is sufficient to shape individuals' own vocal output type and variability regime.

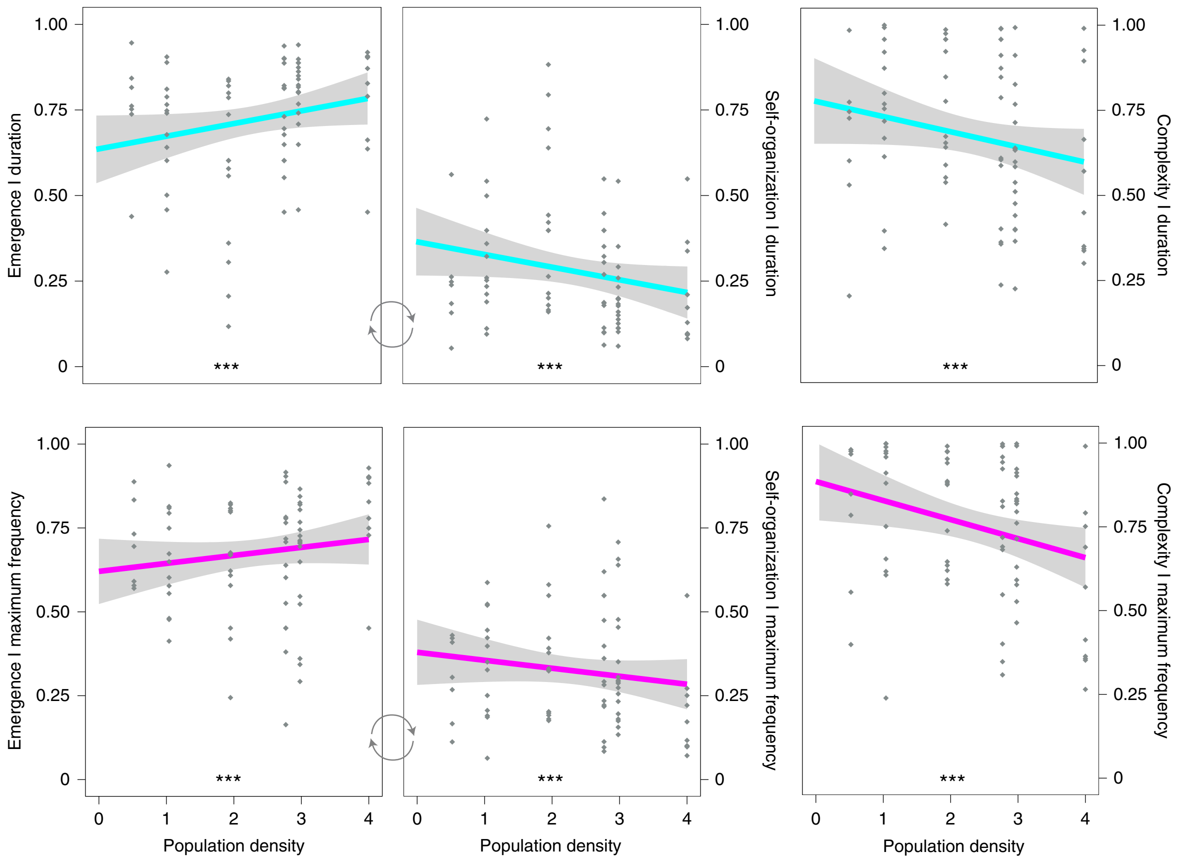

Fig. 2 | Effect of orangutan density on repertoire entropy of alarm calls. Frequency-based measures are shown in magenta and time-based measures in cyan. Shaded areas represent the 95% confidence interval around the mean, and small diamonds represent data points. Populations by order of increasing density (in number of individuals per km^2): Sampan Getek, Sikundur, Sebangau, Tuanan, Gunung Palung and Suaq. Emergence and self-organization are inversely proportional and depicted together for ease of interpretation only. *** denotes significant effects as calculated by linear mixed model analysis after *P* adjustment for false discovery rate (Table 1 and Supplementary Data 4). Graphic representations are based on raw data; differences between density levels are based on model estimates.

Individuals in populations with a lower density also exhibited more complex vocal repertoires. This is in line with population models of cumulative cultural evolution that show that the best breeding grounds for the accumulation of new traits through social learning are dispersed populations with intermittent contact[98]. This is a reminiscent demographic dynamic to the fission–fusion social organization of wild orangutans and that of ancient humans in the African continent[99]. Indeed, ecological changes towards drier habitats brought about by palaeo-climate change in the African continent[100,101] were unlikely to have sustained densely populated communities in the wake of human evolution[102]. Results agree, thus, with computational models, statistical analyses and phylogenetic reconstructions showing that ‘social intelligence' was not an evolutionary driver for human (brain) evolution as much as once believed[103–105].

Some of the vocal dynamics observed contrast with those of captive songbirds and marmoset monkeys: the latter show increased call consistency from young to adult age, whereas we observed the opposite pattern in wild orangutans. Several (non-mutually exclusive) factors may help explain these differences. First, number of tutors probably affects vocal dynamics of novices. For example, marmoset infants attend to one or two tutors during development, but young orangutans seek interaction with multiple adult conspecifics as they gradually become independent[85,106–110], becoming exposed to larger pools of ‘role models' for the acquisition of new behaviours and skills across domains[73,74]. Indeed, when songbirds were experimentally presented with an increased abundance of role models, similar results were obtained[4]. Second, the role of sociality on vocal development in songbirds and marmosets has been observed in transient call types, calls that play a role in supporting vocal development but that are not retained themselves in the mature repertoire[7]. This contrasts with the orangutan calls analysed here; once present in an individual's repertoire, kiss-squeaks are retained in the adult repertoire. Third, life in the wild presents stimuli that are otherwise absent in captivity. For example, by the time a captive infant matures, the range of possible situations that it might encounter in life has been greatly exhausted. This is known to lead to decreasing behavioural variability and potentially to (pathological) stereotypies in captivity. Conversely, the probability of new circumstances in the wild increases once an individual matures and gradually acquires independence, particularly in species with fission–fusion social organization who roam over extensive territories such as orangutans. Wild marmoset studies could help establish a comparison with lab marmoset studies and directly determine wild vs. captivity effects. Finally, acoustic entropy was used in lab studies whereas we used repertoire entropy in the wild. It will be important to determine in the future whether or how entropy at these two levels may be interrelated.

To date, all orangutan study sites have experienced some degree of human impact[17,111,112], particularly in recent decades[20,21], which has pushed populations into dire situations of human–orangutan conflict and survival in the wild (IUCN, Red List of Threatened

Species 2021). For example, our sample included a Sumatran population that lived in a human-dominated degraded landscape[113] that has now become locally extinct (Sampan Getek). The densities reported here have, therefore, not been shaped over millions of years of evolution. The observed correlation between vocal phenotype as a function of sociality corroborates, therefore, the view that the mechanisms at work here operate at a time scale within individual lifetimes, and thus do not reflect automatic, hardwired development programmes shaped by local adaptation over evolutionary time frames.

Concluding remarks

Our findings show that the degree of sociality experienced by individual orangutans in the wild moulds their vocal personality. Findings converge with evidence for active social learning in wild orangutans[109,110,114] that suggest that socially sourced information crosses over into the vocal and communicative domain. We confirm that like human learners exposed to different linguistic communities, social settings help modulate vocal output dynamics and structure in nonhuman hominids. Future models of language origin and human evolution must account for sociality effects on vocal phenotype expression. Extending at least as far back as the phylogenetic rise of the hominid family, low-density populations provided better breeding grounds for high vocal variant complexity.

Methods

Study sites. This study was conducted across six research stations: Tuanan, Gunung Palung and Sabangau in Borneo (*Pongo pygmaeus wurmbii*) and Sikundur, Sampan Getek and Suaq Balimbing in Sumatra (*P. abelii*). This study entailed 2,510 observation hours at Tuanan, 1,520 at Gunung Palung, 311 at Sabangau, 1,132 at Sikundur, 498 at Sampan Getek and 149 at Suaq with a grand total of 6,120 observation hours between 2005 and 2010 and a minimum of five months of uninterrupted orangutan follows and recordings at each site. All sites are laid across the Equator's vicinity and more than 3,000 km away from the Tibetan Plateau. Seasonality is therefore low and without pronounced raining/monsoon vs. dry seasons. No significant effects are hence expected to have arisen due to data having been collected during different overlapping periods/seasons of the year across sites, particularly for calls neither directly nor indirectly related to feeding contexts (for example, food calls and social calls at food patches, respectively). Population estimates were also calculated during these years. Orangutan generation length is typically longer than that of *Pan* and *Gorilla*[115], that is, >25 years; therefore, no significant differences in orangutan density should be expected to have arisen or been biologically possible to have arisen between year of census and year of data collection at each site.

Data recollection. All orangutan kiss-squeaks were opportunistically recorded while following subjects typically at 7 m to 30 m distance from the individuals. Only unaided variants of kiss-squeaks were addressed in the study because other variants are only present in some populations (that is, hand and leaf kiss-squeaks were not considered)[67,68,93]. Calls were recorded at Tuanan using a Marantz Analogue Recorder PMD222 (Marantz Corp.) in combination with a Sennheiser Microphone ME 64 (Sennheiser electronic GmbH & Co. KG) or a Sony Digital Recorder TCD-D100 in combination with a Sony Microphone ECM-M907 (Sony Corp.). In all remaining sites, calls were recorded using a Marantz Analogue Recorder PMD-660 or a ZOOM H4next Handy Recorder (ZOOM Corp.), both connected with a RODE NTG-2 directional microphone (RODE LLC). Audio data were recorded in 16-bit Wave format. No meaningful differences in audio input were expected to result from different professional directional microphones. Audio recordings were collected simultaneously with complete focal behavioural data on the focal animals and other conspecifics when in association. Data collection involved no interaction with or handling of the animals and strictly followed the Indonesian law and research station mandatory guidelines. Orangutan density values were extracted from Husson et al.[78].

Recordings were transferred to a computer with a sampling rate of 44.1 kHz. Duration (s) and maximum frequency (Hz; that of highest dB) were extracted using Raven interactive sound analysis software (version 1.5, Cornell Lab of Ornithology) using the spectrogram window (window type: Hann; 3 dB filter bandwidth: 124 Hz; grid frequency resolution: 2.69 Hz; grid time resolution: 256 samples). Both parameters were extracted directly from the spectrogram window by manually drawing a selection encompassing the complete call from onset to offset.

Data analyses: entropy-based parameters and calculations. Loosely speaking, a complex system can be understood as a dynamical system composed of many elements that display functional/spatial/temporal patterns that cannot be derived from its components by themselves[4,5]. Rather, these components and their future are partially determined by their interactions. There are several frameworks to characterize a system's complexity. From these, statistical Shannon-based complexity measures can be employed to determine the complexity of a system using its states' probability distribution. Particularly, the framework proposed by Santamaría-Bonfil and colleagues[88,116] characterizes a system's complexity, either discrete or continuous, as the trade-off between emergence (that is, the appearance of new systems states) and self-organization (that is, regular patterns in the form of highly probable system states). Here we limit the formal definition of complexity measures (emergence (E), self-organization (S) and complexity (C)) to its discrete form:

$$E = -K \sum_{i=1}^{N} p_i \log_2 p_i \quad (1)$$

$$S = 1 - E \quad (2)$$

$$C = 4 \times E \times S \quad (3)$$

where $p_i = P(X = x)$ is the probability of the element i. Moreover, K is a normalizing constant that constrains E, S and C within $0 \le E; S; C \le 1$ and is estimated as

$$K = \frac{1}{\log_2 b} \quad (4)$$

where b corresponds to the system's alphabet size, the number of states a system can exhibit. It is worth noting that C is only maximal (that is, $C = 1$) when E and S are equal (that is, $E = S = 0{:}5$) and becomes zero for equiprobable or Dirac delta distributions. In systems with more than two states, a high C implies that the system concentrates its dynamics into few highly probable states with many less frequent states (for example, a power-law distribution; Fig. 1 and Supplementary Data 5).

We organized orangutans' acoustic measures into sets per population, individual and context. Afterwards, for each set we calculated the respective entropy-based measures for call's duration (D) and maximal frequency (F) using openly available tools[4] as follows:

For each ith individual from the jth population under the kth ecological context (that is, $x_i^k \in P_j$), we obtained its corresponding E, S and C for duration (D) and maximal frequency (F) such as:

$$E\left(D_{x_i^k}\right),\ S\left(D_{x_i^k}\right) \text{ and } C\left(D_{x_i^k}\right) \quad (5)$$

$$E\left(F_{x_i^k}\right),\ S\left(F_{x_i^k}\right) \text{ and } C\left(F_{x_i^k}\right) \quad (6)$$

Although frequency and duration measurements are continuous, the number of calls per individual in many cases limited the approximation of the empirical probability distribution of these (by means of a kernel density estimation method), leading to spurious results for continuous complexity measurements. Therefore, first we approximated call duration and maximal frequency probability distribution through a histogram (Fig. 1 and Supplementary Data 5). Next, we employed discrete complexity measures as mentioned earlier.

As can be observed in the R code notebook (Supplementary Data 5), in general, orangutan individuals' calls range from low to very high complexity. In the case where individuals had only one record per context, these are regarded as completely self-organized, thus $E = 0$, $S = 1$ and $C = 0$, which can be observed by a group of individuals (for example, Ronaldo, Freddy, Tina and so on). These cases were excluded from subsequent analyses (reduction of $N = 106$ to $N = 89$); together, entropy measures were based on three or fewer calls, as these were expected to provide insufficient coverage of the possible acoustic states for an individual's call variation within a given context ($N = 89$ to $N = 77$). The entropy values that had been calculated for the context 'towards other orangutans' were also removed to avoid including any calls directly exchanged between conspecifics in our analyses to avoid any social confounds as explained in the Introduction ($N = 77$ to $N = 69$).

We should note that the function of these repertoire entropy parameters is to directly quantify the degree/rate of novel or conserved states within a system/call collection. This is not equivalent to detecting vocal convergence/divergence between individuals. For example, two individuals may exhibit *between them* distinct or similar sets of calls (acoustically divergent or convergent, respectively) and show the same level of self-organization in either case, namely, when calls of similar/different acoustics *within* individuals occur at similar rates. Vocal convergence/divergence (and acoustic entropy) is tied to raw acoustics of single calls, whereas repertoire entropy is tied to variation regimes of call collections.

For 'layperson' examples of how these entropic measures can be applied across systems, please see Supplementary Data 5 for flip-a-coin examples and see ref. [87] for examples pertaining to household electric spending, solar flares and bike-sharing services. To consult the open-access 'white paper' dedicated to the comprehensive description and technical explanation of these measures, please see ref. [88]. MATLAB/Octave functions are provided therein for the application of these

measures across natural and artificial systems (in addition to the R code notebook provided in Supplementary Data 5 as applied to our datasets).

Data analyses: linear mixed-effect models. After the entropy measures were estimated for each set, we studied the effect of sex (two levels: female, male), age–sex class (five levels: infant, adolescent, adult female with infant, unflanged male, flanged male), island (two levels: Bornean, Sumatran), context (five levels: towards: observers, animals, humans (non-observers), no apparent danger) on the three entropic measures for maximum frequency and duration (thus, six models in total), including them as fixed control factors. Orangutan density was included as our main fixed factor of interest in all models. We included individual identity as a random effect to control for repeated measures. We implemented our linear mixed models (LLMs) (test model terms: Satterthwaite; model type: III sum of squares) using open-source JASP[117] (v. 0.14.1). Results were plotted using R[118] and 'ggplot2'[119] and 'gridExtra'[120] packages.

Population was not included as a random effect because our design did not include repeated measures at the population level, because the complete resident population at each site was sampled (instead of partial pooling per population) and because the variable is categorical with few levels (that is, six), under which case the variable should be included as a fixed effect instead of random. However, population fully co-varies with orangutan density—the main variable of interest. Orangutan density does not vary within population. Including population would not contribute, therefore, (as random or fixed effect) to control for sampling bias, and its inclusion would spuriously reduce statistical power. (Force-inserting the variable as a fixed effect in our model leads JASP to produce error warnings and abort the operation.) It should be noted that under general statistical heuristics, there is a difference between clear hypothesis testing (X affects Y, hypothesized in advance)—as we do here— versus pure exploratory approaches. Hypothesis testing should seek to avoid model complexification, and this is also the reason why no interactions were included in our model; our working hypothesis did not rely on interactions between fixed factors for verification. Dosed and well-motivated addition of supplementary variables and interactions could be a helpful alternative to understand the phenomena under observation, but only in purely exploratory approaches.

Maximum frequency and duration constituted orthogonal, non-correlated variables (Spearman's rho = −0.017, P = 0.221); however, because they were extracted from the same call event, they should be treated as non-independent. Given that both entropic emergence/self-organization and complexity were in turn derived from both maximum frequency and duration, altogether, this required the results of our linear mixed models to be adjusted for false discovery rate (FDR). To this end, we applied the Hochberg correction procedure[121], 'arguably still the most widely used and cited method for controlling the FDR in practice'[122]. To compute adjusted P values using this correction, we used 'p.adjust {stats}' in R.

Reporting Summary. Further information on research design is available in the Nature Research Reporting Summary linked to this article.

Data and Code Availability

All data and code needed to evaluate the conclusions in the paper are present in the paper and/or the electronic supplementary materials (Supplementary Data 1–5). Additional data may be requested from the authors.

Received: 13 August 2021; Accepted: 2 February 2022;
Published online: 21 March 2022

References

1. Lipkind, D. et al. Stepwise acquisition of vocal combinatorial capacity in songbirds and human infants. *Nature* **498**, 104–108 (2013).
2. Goldstein, M., King, A. P. & West, M. J. Social interaction shapes babbling: testing parallels between birdsong and speech. *Proc. Natl Acad. Sci. USA* **100**, 8030–8035 (2003).
3. Fehér, O., Ljubičić, I., Suzuki, K., Okanoya, K. & Tchernichovski, O. Statistical learning in songbirds: from self-tutoring to song culture. *Phil. Trans. R. Soc. B* **372**, 20160053 (2017).
4. Tchernichovski, O., Lints, T., Mitra, P. P. & Nottebohm, F. Vocal imitation in zebra finches is inversely related to model abundance. *Proc. Natl Acad. Sci. USA* **96**, 12901–12904 (1999).
5. Tchernichovski, O. Dynamics of the vocal imitation process: how a zebra finch learns its song. *Science* **291**, 2564–2569 (2001).
6. Fehér, O., Wang, H., Saar, S., Mitra, P. P. & Tchernichovski, O. De novo establishment of wild-type song culture in the zebra finch. *Nature* **459**, 564–568 (2009).
7. Takahashi, D. et al. The developmental dynamics of marmoset monkey vocal production. *Science* **349**, 734–738 (2015).
8. Takahashi, D. Y., Liao, D. A. & Ghazanfar, A. A. Vocal learning via social reinforcement by infant marmoset monkeys. *Curr. Biol.* **27**, 1844–1852.E6 (2017).
9. Takahashi, D. Y., Fenley, A. R. & Ghazanfar, A. A. Early development of turn-taking with parents shapes vocal acoustics in infant marmoset monkeys. *Phil. Trans. R. Soc. B* **371**, 20150370 (2016).
10. Gultekin, Y. B. & Hage, S. R. Limiting parental interaction during vocal development affects acoustic call structure in marmoset monkeys. *Sci. Adv.* **4**, eaar4012 (2018).
11. Gultekin, Y. B. & Hage, S. R. Limiting parental feedback disrupts vocal development in marmoset monkeys. *Nat. Commun.* **8**, 14046 (2017).
12. Jarvis, E. D. Evolution of vocal learning and spoken language. *Science* **366**, 50–54 (2019).
13. Snowdon, C. T. Learning from monkey "talk". *Science* **355**, 1120–1122 (2017).
14. Malik, K. Rights and wrongs. *Nature* **406**, 675–676 (2000).
15. Wise, S. M. & Goodall, J. *Rattling the Cage: Toward Legal Rights for Animals* (Da Capo Press, 2017).
16. Grayson, L. *Animals in Research: For and Against* (British Library, 2000).
17. Nater, A. et al. Morphometric, behavioral, and genomic evidence for a new orangutan species. *Curr. Biol.* **27**, 3487–3498.E10 (2017).
18. Estrada, A. et al. Impending extinction crisis of the world's primates: why primates matter. *Sci. Adv.* **3**, e1600946 (2017).
19. Ross, S. et al. Inappropriate use and portrayal of chimpanzees. *Science* **319**, 1487 (2008).
20. Wich, S. A. et al. Land-cover changes predict steep declines for the Sumatran orangutan (*Pongo abelii*). *Sci. Adv.* **2**, e1500789 (2016).
21. Wich, S. A. et al. Understanding the impacts of land-use policies on a threatened species: is there a future for the Bornean orangutan? *PLoS ONE* **7**, e49142 (2012).
22. Wich, S. A. et al. Will oil palm's homecoming spell doom for Africa's great apes? *Curr. Biol.* https://doi.org/10.1016/j.cub.2014.05.077 (2014).
23. Fitch, T. W. Empirical approaches to the study of language evolution. *Psychon. Bull. Rev*. **24**, 3–33 (2017).
24. Hauser, M. D. et al. The mystery of language evolution. *Front. Psychol.* https://doi.org/10.3389/fpsyg.2014.00401 (2014)
25. Corballis, M. C. Crossing the Rubicon: behaviorism, language, and evolutionary continuity. *Front. Psychol.* **11**, 653 (2020).
26. Berwick, R. C. & Chomsky, N. All or nothing: no half-merge and the evolution of syntax. *PLoS Biol.* **17**, e3000539 (2019).
27. Bolhuis, J. J. & Wynne, C. D. Can evolution explain how minds work? *Nature* **458**, 832–833 (2009).
28. Hayes, K. J. & Hayes, C. The intellectual development of a home-raised chimpanzee. *Proc. Am. Phil. Soc.* **95**, 105–109 (1951).
29. Premack, D. Language in chimpanzee? *Science* **172**, 808–822 (1971).
30. Terrace, H., Petitto, L., Sanders, R. & Bever, T. Can an ape create a sentence? *Science* **206**, 891–902 (1979).
31. Patterson, F. & Linden, E. *The Education of Koko* (Holt, Rinehart and Winston, 1981).
32. Leavens, D. A., Bard, K. A. & Hopkins, W. D. BIZARRE chimpanzees do not represent "the chimpanzee". *Behav. Brain Sci.* **33**, 100–101 (2010).
33. Lameira, A. R. Bidding evidence for primate vocal learning and the cultural substrates for speech evolution. *Neurosci. Biobehav. Rev.* **83**, 429–439 (2017).
34. Lameira, A. R. et al. Speech-like rhythm in a voiced and voiceless orangutan call. *PLoS ONE* **10**, e116136 (2015).
35. Lameira, A. R. & Shumaker, R. W. Orangutans show active voicing through a membranophone. *Sci. Rep.* **9**, 12289 (2019).
36. Lameira, A. R., Hardus, M. E., Mielke, A., Wich, S. A. & Shumaker, R. W. Vocal fold control beyond the species-specific repertoire in an orangutan. *Sci. Rep.* **6**, 30315 (2016).
37. Lameira, A. R. et al. Orangutan (*Pongo* spp.) whistling and implications for the emergence of an open-ended call repertoire: a replication and extension. *J. Acoust. Soc. Am.* **134**, 2326–2335 (2013).
38. Perlman, M. & Clark, N. Learned vocal and breathing behavior in an enculturated gorilla. *Anim. Cogn.* **18**, 1165–1179 (2015).
39. Wich, S. et al. A case of spontaneous acquisition of a human sound by an orangutan. *Primates* **50**, 56–64 (2009).
40. Lameira, A. R., Maddieson, I. & Zuberbuhler, K. Primate feedstock for the evolution of consonants. *Trends Cogn. Sci.* **18**, 60–62 (2014).
41. Lameira, A. R. The forgotten role of consonant-like calls in theories of speech evolution. *Behav. Brain Sci.* **37**, 559–560 (2014).
42. Boë, L.-J. et al. Which way to the dawn of speech? Reanalyzing half a century of debates and data in light of speech science. *Sci. Adv.* **5**, eaaw3916 (2019).
43. Boë, L. J. et al. Evidence of a vocalic proto-system in the baboon (*Papio papio*) suggests pre-hominin speech precursors. *PLoS ONE* **12**, e0169321 (2017).
44. Fitch, T. W., Boer, B., Mathur, N. & Ghazanfar, A. A. Monkey vocal tracts are speech-ready. *Sci. Adv.* **2**, e1600723 (2016).
45. Pereira, A. S., Kavanagh, E., Hobaiter, C., Slocombe, K. E. & Lameira, A. R. Chimpanzee lip-smacks confirm primate continuity for speech-rhythm evolution. *Biol. Lett.* **16**, 20200232 (2020).

46. Lameira, A. R. et al. Proto-consonants were information-dense via identical bioacoustic tags to proto-vowels. *Nat. Hum. Behav.* **1**, 0044 (2017).
47. Lameira, A. R. et al. Orangutan information broadcast via consonant-like and vowel-like calls breaches mathematical models of linguistic evolution. *Biol. Lett.* **17**, 20210302 (2021).
48. Watson, S. K. et al. Nonadjacent dependency processing in monkeys, apes, and humans. *Sci. Adv.* **6**, eabb0725 (2020).
49. Lameira, A. R. & Call, J. Time-space–displaced responses in the orangutan vocal system. *Sci. Adv.* **4**, eaau3401 (2018).
50. Belyk, M. & Brown, S. The origins of the vocal brain in humans. *Neurosci. Biobehav. Rev.* **77**, 177–193 (2017).
51. Crockford, C., Wittig, R. M. & Zuberbuhler, K. Vocalizing in chimpanzees is influenced by social-cognitive processes. *Sci. Adv.* **3**, e1701742 (2017).
52. Taglialatela, J. P., Reamer, L., Schapiro, S. J. & Hopkins, W. D. Social learning of a communicative signal in captive chimpanzees. *Biol. Lett.* **8**, 498–501 (2012).
53. Russell, J. L., Joseph, M., Hopkins, W. D. & Taglialatela, J. P. Vocal learning of a communicative signal in captive chimpanzees, *Pan troglodytes*. *Brain Lang.* **127**, 520–525 (2013).
54. Hopkins, W. D. et al. Genetic factors and orofacial motor learning selectively influence variability in central sulcus morphology in chimpanzees (*Pan troglodytes*). *J. Neurosci.* **37**, 5475–5483 (2017).
55. Staes, N. et al. FOXP2 variation in great ape populations offers insight into the evolution of communication skills. *Sci. Rep.* **7**, 16866 (2017).
56. Martins, P. T. & Boeckx, C. Vocal learning: beyond the continuum. *PLoS Biol.* **18**, e3000672 (2020).
57. Watson, S. K. et al. Vocal learning in the functionally referential food grunts of chimpanzees. *Curr. Biol.* **25**, 495–499 (2015).
58. Hopkins, W. D., Taglialatela, J. P. & Leavens, D. A. Chimpanzees differentially produce novel vocalizations to capture the attention of a human. *Anim. Behav.* **73**, 281–286 (2007).
59. Bianchi, S., Reyes, L. D., Hopkins, W. D., Taglialatela, J. P. & Sherwood, C. C. Neocortical grey matter distribution underlying voluntary, flexible vocalizations in chimpanzees. *Sci. Rep.* **6**, 34733 (2016).
60. Wich, S. A. et al. Call cultures in orangutans? *PLoS ONE* **7**, e36180 (2012).
61. Crockford, C., Herbinger, I., Vigilant, L. & Boesch, C. Wild chimpanzees produce group-specific calls: a case for vocal learning? *Ethology* **110**, 221–243 (2004).
62. Whiten, A. et al. Cultures in chimpanzees. *Nature* **399**, 682–685 (1999).
63. van Schaik, C. P. et al. Orangutan cultures and the evolution of material culture. *Science* **299**, 102–105 (2003).
64. Whiten, A. Culture extends the scope of evolutionary biology in the great apes. *Proc. Natl Acad. Sci. USA* **114**, 7790–7797 (2017).
65. Koops, K., Visalberghi, E. & van Schaik, C. The ecology of primate material culture. *Biol. Lett.* **10**, 20140508 (2014).
66. Kalan, A. K. et al. Chimpanzees use tree species with a resonant timbre for accumulative stone throwing. *Biol. Lett.* **15**, 20190747 (2019).
67. Hardus, M., Lameira, A. R., Van Schaik, C. P. & Wich, S. A. Tool use in wild orangutans modifies sound production: a functionally deceptive innovation? *Proc. R. Soc. B* https://doi.org/10.1098/rspb.2009.1027 (2009).
68. Lameira, A. R. et al. Population-specific use of the same tool-assisted alarm call between two wild orangutan populations (*Pongo pygmaeus wurmbii*) indicates functional arbitrariness. *PLoS ONE* **8**, e69749 (2013).
69. Hohmann, G. & Fruth, B. Culture in bonobos? Between-species and within-species variation in behavior. *Curr. Anthropol.* **44**, 563–571 (2003).
70. Robbins, M. M. et al. Behavioral variation in gorillas: evidence of potential cultural traits. *PLoS ONE* **11**, e0160483 (2016).
71. Kühl, H. S. et al. Human impact erodes chimpanzee behavioral diversity. *Science* **363**, 1453–1455 (2019).
72. van Schaik, C. P. Fragility of Traditions: the disturbance hypothesis for the loss of local traditions in orangutans. *Int. J. Primatol.* **23**, 527–538 (2002).
73. Delgado, R. A. & van Schaik, C. P. The behavioral ecology and conservation of the orangutan (*Pongo pygmaeus*): a tale of two islands. *Evol. Anthropol.* **9**, 201–218 (2000).
74. van Schaik, C. The socioecology of fission–fusion sociality in orangutans. *Primates* **40**, 69–86 (1999).
75. Nater, A. et al. Sex-biased dispersal and volcanic activities shaped phylogeographic patterns of extant orangutans (genus: *Pongo*). *Mol. Biol.* **28**, 2275–2288 (2011).
76. Arora, N. et al. Parentage-based pedigree reconstruction reveals female matrilineal clusters and male-biased dispersal in nongregarious Asian great apes, the Bornean orangutans (*Pongo pygmaeus*). *Mol. Ecol.* **21**, 3352–3362 (2012).
77. Kavanagh, E. et al. Dominance style is a key predictor of vocal use and evolution across nonhuman primates. *R. Soc. Open Sci.* **8**, 210873 (2021).
78. Husson, S. et al. in *Orangutans: Geographic Variation in Behavioral Ecology and Conservation* (eds Wich, S. et al.) Ch. 6 (Oxford Univ. Press, 2009).
79. van Noordwijk, M. A. et al. in *Orangutans: Geographic Variation in Behavioral Ecology and Conservation* (eds Wich, S. et al.) Ch. 12 (Oxford Univ Press, 2009).
80. Singleton, I., Knott, C., Morrogh-Bernard, H., Wich, S. & van Schaik, C. P. in *Orangutans: Geographic Variation in Behavioral Ecology and Conservation* (eds Wich, S. et al.) Ch. 13 (Oxford Univ. Press, 2009).
81. Wich, S. et al. Life history of wild Sumatran orangutans (*Pongo abelii*). *J. Hum. Evol.* **47**, 385–398 (2004).
82. Wich, S. et al. in *Orangutans: Geographic Variation in Behavioral Ecology and Conservation* (eds Wich, S. et al.) Ch. 5 (Oxford Univ. Press, 2009).
83. Shumaker, R. W., Wich, S. A. & Perkins, L. Reproductive life history traits of female orangutans (*Pongo* spp.). *Primate Reprod. Aging* **36**, 147–161 (2008).
84. Freund, C., Rahman, E. & Knott, C. Ten years of orangutan-related wildlife crime investigation in West Kalimantan, Indonesia. *Am. J. Primatol.* **79**, 22620 (2016).
85. van Noordwijk, M. A. & van Schaik, C. P. Development of ecological competence in Sumatran orangutans. *Am. J. Phys. Anthropol.* **127**, 79–94 (2005).
86. Knot, C. D. et al. The Gunung Palung Orangutan Project: Twenty-five years at the intersection of research and conservation in a critical landscape in Indonesia. *Biol. Conserv.* **255**, 108856 (2021).
87. Guillermo, S.-B., Gershenson, C. & Fernández, N. A package for measuring emergence, self-organization, and complexity based on shannon entropy. *Front. Robot. AI* **4**, 174102 (2017).
88. Santamaría-Bonfil, G., Fernández, N. & Gershenson, C. Measuring the complexity of continuous distributions. *Entropy* **18**, 72 (2016).
89. Kalan, A. K., Mundry, R. & Boesch, C. Wild chimpanzees modify food call structure with respect to tree size for a particular fruit species. *Anim. Behav.* **101**, 1–9 (2015).
90. Fedurek, P. & Slocombe, K. E. The social function of food-associated calls in male chimpanzees. *Am. J. Primatol.* **75**, 726–739 (2013).
91. Luef, E., Breuer, T. & Pika, S. Food-associated calling in gorillas (*Gorilla g. gorilla*) in the wild. *PLoS ONE* **11**, e0144197 (2016).
92. Clay, Z. & Zuberbuhler, K. Food-associated calling sequences in bonobos. *Anim. Behav.* **77**, 1387–1396 (2009).
93. Hardus, M. E. et al. in *Orangutans: Geographic Variation in Behavioral Ecology and Conservation* (eds Wich, S. et al.) Ch. 4 (Oxford Univ. Press, 2009).
94. Wich, S. A. et al. Forest fruit production is higher on Sumatra than on Borneo. *PLoS ONE* **6**, e21278 (2011).
95. Lameira, A. R. & Wich, S. Orangutan long call degradation and individuality over distance: a playback approach. *Int. J. Primatol.* **29**, 615–625 (2008).
96. Lameira, A. R., Delgado, R. & Wich, S. Review of geographic variation in terrestrial mammalian acoustic signals: human speech variation in a comparative perspective. *J. Evolut. Psychol.* **8**, 309–332 (2010).
97. Lameira, A. R. et al. Predator guild does not influence orangutan alarm call rates and combinations. *Behav. Ecol. Sociobiol.* **67**, 519–528 (2013).
98. Derex, M. & Mesoudi, A. Cumulative cultural evolution within evolving population structures. *Trends Cogn. Sci.* **24**, 654–667 (2020).
99. Scerri, E. M. et al. Did our species evolve in subdivided populations across Africa, and why does it matter? *Trends Ecol. Evol.* **33**, 582–594 (2018).
100. Kaya, F. et al. The rise and fall of the Old World savannah fauna and the origins of the African savannah biome. *Nat. Ecol. Evol.* **2**, 241–246 (2018).
101. Bobe, R. The expansion of grassland ecosystems in Africa in relation to mammalian evolution and the origin of the genus *Homo*. *Palaeogeogr. Palaeoclimatol. Palaeoecol.* **207**, 399–420 (2004).
102. Zhu, D., Galbraith, E. D., Reyes-García, V. & Ciais, P. Global hunter-gatherer population densities constrained by influence of seasonality on diet composition. *Nat. Ecol. Evol.* **5**, 1536–1545 (2021).
103. DeCasien, A. R., Williams, S. A. & Higham, J. P. Primate brain size is predicted by diet but not sociality. *Nat. Ecol. Evol.* **1**, 0112 (2017).
104. Mauricio, G.-F. & Gardner, A. Inference of ecological and social drivers of human brain-size evolution. *Nature* **557**, 554–557 (2018).
105. Lindenfors, P., Wartel, A. & Lind, J. 'Dunbar's number' deconstructed. *Biol. Lett.* **17**, 20210158 (2021).
106. Schuppli, C., van Noordwijk, M., Atmoko, S. U. & van Schaik, C. Early sociability fosters later exploratory tendency in wild immature orangutans. *Sci. Adv.* **6**, eaaw2685 (2020).
107. Schuppli, C. et al. Observational social learning and socially induced practice of routine skills in immature wild orangutans. *Anim. Behav.* **119**, 87–98 (2016).
108. Jaeggi, A. V. et al. Social learning of diet and foraging skills by wild immature Bornean orangutans: implications for culture. *Am. J. Primatol.* **72**, 62–71 (2010).
109. Schuppli, C. et al. The effects of sociability on exploratory tendency and innovation repertoires in wild Sumatran and Bornean orangutans. *Sci. Rep.* **7**, 15464 (2017).

110. Ehmann, B. et al. Immature wild orangutans acquire relevant ecological knowledge through sex-specific attentional biases during social learning. *PLoS Biol.* **19**, e3001173 (2021).
111. Meijaard, E. et al. Declining orangutan encounter rates from Wallace to the present suggest the species was once more abundant. *PLoS ONE* **5**, e12042 (2010).
112. Marshall, A. J. et al. The blowgun is mightier than the chainsaw in determining population density of Bornean orangutans (*Pongo pygmaeus morio*) in the forests of East Kalimantan. *Biol. Conserv.* **129**, 566–578 (2006).
113. Gail, C.-S., Miran, C.-S., Singleton, I. & Linkie, M. Raiders of the lost bark: orangutan foraging strategies in a degraded landscape. *PLoS ONE* **6**, e20962 (2011).
114. Schuppli, C. & van Schaik, C. P. Animal cultures: how we've only seen the tip of the iceberg. *Evol. Hum. Sci.* **1**, e2 (2019).
115. Langergraber, K. E. et al. Vigilant, generation times in wild chimpanzees and gorillas suggest earlier divergence times in great ape and human evolution. *Proc. Natl Acad. Sci. USA* **109**, 15716–15721 (2012).
116. Fernández, N., Maldonado, C. & Gershenson, C. in *Guided Self-Organization: Inception* (ed Prokopenko, M.) 19–51 (Springer Berlin Heidelberg, 2014).
117. JAST Team, *JASP* (Univ. of Amsterdam, 2020).
118. R Core Team *R: A Language and Environment for Statistical Computing* (R Foundation for Statistical Computing, 2013).
119. Wickham, H. *ggplot2: Elegant Graphics for Data Analysis* (Springer-Verlag, 2009).
120. Auguie, B. gridExtra: Functions in grid graphics. R version 0.9.1 (2012).
121. Benjamini, Y. & Hochberg, Y. Controlling the false discovery rate: a practical and powerful approach to multiple testing. *J. R. Stat. Soc. B* **57**, 289–300 (1995).
122. Korthauer, K. et al. A practical guide to methods controlling false discoveries in computational biology. *Genome Biol.* **20**, 118 (2019).

Acknowledgements
We thank the Indonesian Ministry of Research and Technology, the Indonesian Ministry of Environment and Forestry, the Directorate General of Natural Resources and Ecosystem Conservation, the former Directorate General of Forest Protection and Nature Conservation, the Gunung Palung National Park, the Gunung Leuser National Park and the Leuser Ecosystem Management Authority for authorization to carry out research in Indonesia; the Universitas National, the Center for International Cooperation in Sustainable Management of Tropical Peatland, the University of Palangkaraya and the Tanjungpura University for supporting the project and acting as sponsors and counter-partners; the Sumatran Orangutan Conservation Programme—PanEco Foundation and C. van Schaik and M. van Noordwijk for logical support; the US Fish and Wildlife Service Great Apes Conservation Fund, ARCUS and the Borneo Nature Foundation for financial support; all field assistants and students for their help and hard work with data collection. We thank three anonymous reviewers for constructive commentary in previous versions of the manuscript. A.R.L. was supported by the European Union's Horizon 2020 Research and Innovation Program under the Marie Skłodowska-Curie grant agreement number 702137 and the UK Research and Innovation's Future Leaders Fellowship grant agreement number MR/T04229X/1.

Author contributions
A.R.L. conceived and designed the study. A.R.L., M.E.H. and D.G. collected data. A.R.L., M.G. and G.S.-B. analysed data. M.G., C.D.K., H.M.-B., M.G.N., G.C.-S. and S.A.W. provided materials/analysis tools and site-specific expertise. A.R.L., G.C.-S., M.E.H. and S.A.W. wrote the paper with the assistance and input of all co-authors.

Competing interests
The authors declare no competing interests.

Additional information
Supplementary information The online version contains supplementary material available at https://doi.org/10.1038/s41559-022-01689-z.

Correspondence and requests for materials should be addressed to Adriano R. Lameira.

Peer review information *Nature Ecology & Evolution* thanks Ofer Tchernichovski and the other, anonymous, reviewer(s) for their contribution to the peer review of this work.

Reprints and permissions information is available at www.nature.com/reprints.

Publisher's note Springer Nature remains neutral with regard to jurisdictional claims in published maps and institutional affiliations.

Open Access This article is licensed under a Creative Commons Attribution 4.0 International License, which permits use, sharing, adaptation, distribution and reproduction in any medium or format, as long as you give appropriate credit to the original author(s) and the source, provide a link to the Creative Commons license, and indicate if changes were made. The images or other third party material in this article are included in the article's Creative Commons license, unless indicated otherwise in a credit line to the material. If material is not included in the article's Creative Commons license and your intended use is not permitted by statutory regulation or exceeds the permitted use, you will need to obtain permission directly from the copyright holder. To view a copy of this license, visit http://creativecommons.org/licenses/by/4.0/.
© The Author(s) 2022
